# Antimicrobial Non-Susceptibility of *Escherichia coli* from Outpatients and Patients Visiting Emergency Rooms in Taiwan

**DOI:** 10.1371/journal.pone.0144103

**Published:** 2015-12-03

**Authors:** Jann-Tay Wang, Shan-Chwen Chang, Feng-Yee Chang, Chang-Phone Fung, Yin-Ching Chuang, Yao-Shen Chen, Yih-Ru Shiau, Mei-Chen Tan, Hui-Ying Wang, Jui-Fen Lai, I-Wen Huang, Tsai-Ling Yang Lauderdale

**Affiliations:** 1 Department of Internal Medicine, National Taiwan University Hospital, Taipei, Taiwan; 2 National Institute of Infectious Diseases and Vaccinology, National Health Research Institutes, Zhunan, Taiwan; 3 Graduate Institute of Clinical Pharmacy, College of Medicine, National Taiwan University, Taipei, Taiwan; 4 Division of Infectious Diseases and Tropical Medicine, Tri-Service General Hospital, Taipei, Taiwan; 5 Department of Internal Medicine, National Defense Medical Center, Taipei, Taiwan; 6 Division of Infectious Diseases, Department of Medicine, Taipei Veterans General Hospital, Taipei, Taiwan; 7 Department of Internal Medicine, Chi-Mei Medical Center, Tainan, Taiwan; 8 Department of Medicine, Kaohsiung Veterans General Hospital, Kaohsiung, Taiwan; 9 School of Medicine, National Yang Ming University, Taipei, Taiwan; Faculdade de Medicina de Lisboa, PORTUGAL

## Abstract

Longitudinal nationwide surveillance data on antimicrobial non-susceptibility and prevalence of extended-spectrum β-lactamases (ESBLs) as well as AmpC β-lactamases producers among *Escherichia coli* from different sources in the community settings are limited. Such data may impact treatment practice. The present study investigated *E*. *coli* from outpatients and patients visiting emergency rooms collected by the Taiwan Surveillance of Antimicrobial Resistance (TSAR) program. A total of 3481 *E*. *coli* isolates were studied, including 2153 (61.9%) from urine and 1125 (32.3%) from blood samples. These isolates were collected biennially between 2002 and 2012 from a total of 28 hospitals located in different geographic regions of Taiwan. Minimum inhibitory concentrations (MIC) were determined using methods recommended by the Clinical Laboratory Standards Institute (CLSI). The prevalence and factors associated with the presence of ESBL and AmpC β-lactamase-producers were determined. Significant increases in non-susceptibility to most β-lactams and ciprofloxacin occurred during the study period. By 2012, non-susceptibility to cefotaxime and ciprofloxacin reached 21.1% and 26.9%, respectively. The prevalence of ESBL- and AmpC- producers also increased from 4.0% and 5.3%, respectively, in 2002–2004, to 10.7% for both in 2010–2012 (*P* < 0.001). The predominant ESBL and AmpC β-lactamase genes were CTX-M and CMY-types, respectively. Non-susceptibility of urine isolates to nitrofurantoin remained at around 8% and to fosfomycin was low (0.7%) but to cefazolin (based on the 2014 CLSI urine criteria) increased from 11.5% in 2002–2004 to 23.9% in 2010–2012 (*P* <0.001). Non-susceptibility of isolates from different specimen types was generally similar, but isolates from elderly patients were significantly more resistant to most antimicrobial agents and associated with the presence of ESBL- and AmpC- β-lactamases. An additional concern is that decreased ciprofloxacin susceptibility (MIC 0.12–1 mg/L) was as high as 25% in isolates from all age groups, including those from pediatric patients. Our data indicated that there is a need to re-evaluate appropriate treatment selection for community-acquired infections in Taiwan. Identification of community reservoirs of multidrug-resistant *E*. *coli* is also warranted.

## Introduction

*Escherichia coli* is the most common Gram-negative bacteria to cause various infection syndromes in humans, including urinary tract infections (UTI), bacteremia, meningitis, and gastrointestinal illnesses [1]. It is the leading cause of UTI and accounts for 70–95% of community-onset UTI cases [2]. Timely administration of effective antibiotics plays a key role in resolution of bacterial infections [3, 4]. Fluoroquinolones and third generation cephalosporins are two groups of antibiotics usually recommended by therapeutic guidelines for treating infections caused by *Enterobacteriaceae*, including *E*. *coli* [5, 6], whereas nitrofurantoin, trimethoprim-sulfamethoxazole, and fosfomycin, may be considered for the treatment of uncomplicated and community-acquired UTI depending on the prevalence of resistance [5, 7].

Resistance to fluoroquinolones (FQ) and third-generation cephalosporins has increased significantly in clinical isolates of *E*. *coli* [8, 9]. A leading cause contributing to the increase of drug resistance has been dissemination of extended-spectrum β-lactamase (ESBL)—and/or AmpC β-lactamase-producers [10–13]. However, the prevalence of antimicrobial resistance can vary substantially between countries and regions. For example, ESBL-producers accounted for 5.9% of community-associated UTI *E*. *coli* isolated during 2009–2011 in the US [8], while it ranged 1.8% to 25.2% in *E*. *coli* isolated during 2004–2010 in Eastern European countries [14]. Information on the prevalence of AmpC-producers in clinical *E*. *coli* isolates is limited, but has been reported to be low, at 1.1% in Spain and 2% in China on isolates from 2010–2011 [15, 16]. A recent global surveillance study on gram-negative pathogens causing UTI isolated during 2009–2011 from different countries found FQ resistance to vary widely with a range of 6% in Estonia and 75% in India [17]. In Europe, the overall FQ non-susceptibility of *E*. *coli* isolates from 2013 also varied considerably between countries, from 11.7% in Norway to 51.9% in Cyprus [18]. Such variations highlight the need for surveillance in each country to define the extent of the problem and help identify unusual resistance problems that exist locally [3]. These data can also impact empirical therapy practices in each country.

Most studies, including some of the data mentioned above focused on isolates from hospitalized patients or do not differentiate inpatient and outpatient source. Longitudinal nationwide surveillance data on antimicrobial resistance of *E*. *coli* from community settings are limited and the isolates surveyed are mostly from urinary tract infections [19, 20]. In addition, the prevalence of ESBL and AmpC β-lactamases-producers in *E*. *coli* isolates from the community setting are scarce. The effect of the 2014 Clinical and Laboratory Standards Institute (CLSI) revised interpretive criteria on cefazolin for *E*. *coli* isolates from urine, on cefepime for Enterobacteriaceae [21] and on *E*. *coli* non-susceptibility from our region is also unknown. The Taiwan Surveillance of Antimicrobial Resistance (TSAR) is a biennial multicenter program conducted at the National Health Research Institutes [22, 23]. The present study analyzed the TSAR data from 2002 to 2012 on *E*. *coli* from different sources in the community setting to address the aforementioned issues.

## Methods

### Isolate collection

*E*. *coli* isolates were collected as part of the TSAR program conducted biennially between 2002 and 2012 (corresponding to TSAR III–VIII). Isolates were collected from medical centers and regional hospitals, including a total number of 28 hospitals located in the 4 geographic regions of Taiwan following previously described protocol [23]. All isolates were stored at −70°C. Only *E*. *coli* clinical isolates from outpatients and patients visiting emergency rooms were enrolled in the present study. The TSAR project was approved by the Research Ethics Committee of National Health Research Institutes, Taiwan (EC960205 and EC1010602-E). Written informed consent was not obtained because the study only used bacterial isolates recovered from clinical samples taken as part of standard care and patient information was anonymized and de-identified prior to analysis.

### Isolate identification

Clinical isolates reported to be *E*. *coli* by hospitals were first subcultured to blood agar and MacConkey agar plates for purity check. Species confirmation was based on colony morphology, positive spot indole and β-glucuronidase [24, 25]. For isolates not typical of *E*. *coli* colony morphology or if negative for either of the above biochemical reactions, Vitek II GN cards were used (bioMérieux, Marcy l’Etoile, France).

### Antimicrobial susceptibility testing (AST)

Minimum inhibitory concentrations (MICs) were determined using reference broth microdilution method following the guidelines of the manufacturer and CLSI [21]. Sensititre custom-designed plates were used from TSAR III (2002) to TSAR VI (2008), and the standard GNX2F plates were used in TSAR VII (2010) and TSAR VIII (2012) [ThermoFisher Scientific (formerly Trek Diagnostics), East Grinstead, UK]. All isolates were subcultured twice on sheep blood agar plate from -70°C before AST. Quality control was performed using *E*. *coli* ATCC 25922, *E*. *coli* ATCC 35218, *Klebsiella pneumoniae* ATCC 700603, and *Pseudomonas aeruginosa* ATCC 27853.

The following antimicrobial agents were tested on isolates from all study years: amikacin, ampicillin, aztreonam, cefazolin, cefepime, cefotaxime, cefoxitin, ceftazidime, cefuroxime, ciprofloxacin, gentamicin, and imipenem. Other agents not tested in all years included (years tested) amoxicillin/clavulanate (2002–2008), ertapenem (2012), nitrofurantoin (2002–2006, and 2012 on urine isolates), piperacillin (2002–2010), tetracycline (2002–2008), tigecycline (2010 and 2012), Fosfomycin was tested on 2012 isolates from urine only by the agar dilution method using Mueller-Hinton agar supplemented with 25 mg/L of glucose-6-phosphate. Interpretive criteria are based on the 2014 CLSI breakpoints [21]. Decreased ciprofloxacin susceptibility was defined as isolates having ciprofloxacin MIC in the 0.12–1 mg/L range. Susceptibility to cefazolin on urine isolates was determined using the urine and non-urine breakpoints. Susceptibility to tigecycline was interpreted using breakpoints proposed by the European Committee on Antimicrobial Susceptibilities Testing (EUCAST) (http://www.eucast.org/clinical_breakpoints/)

### Detections of ESBL, AmpC β-lactamase, and carbapenemase genes

The CLSI ESBL confirmatory test was performed on all isolates with aztreonam, ceftazidime, or cefotaxime MIC ≥ 2 mg/L using cefotaxime and ceftazidime disks with and without clavulanate [21]. These ESBL screening test-positive isolates were also subject to detection of ESBL and/or AmpC β-lactamases genes by multiplex PCR following previously described primers and protocols [26, 27]. Isolates non-susceptible to carbapenem were subject to carbapenemase PCR using published primers [28].

### Data analysis

Susceptibility interpretation analysis was made using the WHONET software [29]. Intermediate susceptibility and resistance were grouped together as “non-susceptibility”. Duplicate isolates were excluded before analysis. TSAR III and IV (2002–2004), TSAR V and VI (2006–2008), and TSAR VII and VIII (2010–2012) were grouped as Periods I, II, and III, respectively, in the related analysis. Categorical variables were compared using chi-square test or Fisher’s exact test (if the number was less than 10). If statistical difference was obtained when the tested categorical variables with three different levels were compared, post-hoc analysis was performed to identify which level was significantly different from the others. Trends were analyzed using chi-square for trend analysis. Multivariable logistic regression analysis was performed to assess the variables (including study year, specimen type, and patient age group) among ESBL or AmpC β-lactamase–producers vs.–non-producers. SAS 9.2 (SAS Institute, Cary, NC, USA) was used for the above analyses. A 2-tailed P value less than 0.05 was considered statistically significant.

## Results

### Isolates

A total of 3,481 non-duplicate *E*. *coli* isolates were enrolled, with 401, 485, 526, 612, 674, and 783 isolates from TSAR III (2002), IV (2004), V (2006), VI (2008), VII (2010), and VIII (2012), respectively. By geographic regions, a total of 1091, 979, 1004, and 407 isolates were from the northern, central, southern, and eastern parts of Taiwan, respectively. The most common specimen type was urine (2153, 61.9%), followed by blood (1125, 32.3%), and others (203, 5.8%). The age of the source patients was missing in 74 patients. The mean ± standard deviation age of the remaining 3407 patients was 57.4 ± 23.6 years (y), with 6.9% (234) being pediatric patients (≤ 18 y, mean 4.3 y), of whom the majority (195) were younger than 10 years of age, 45.9% being adult (19–64 y, mean 45.0 y), and 47.3% being elderly (≥ 65 y, mean 77 y).

### Non-susceptibility to different antimicrobial agents over the study period

**Fig 1:**
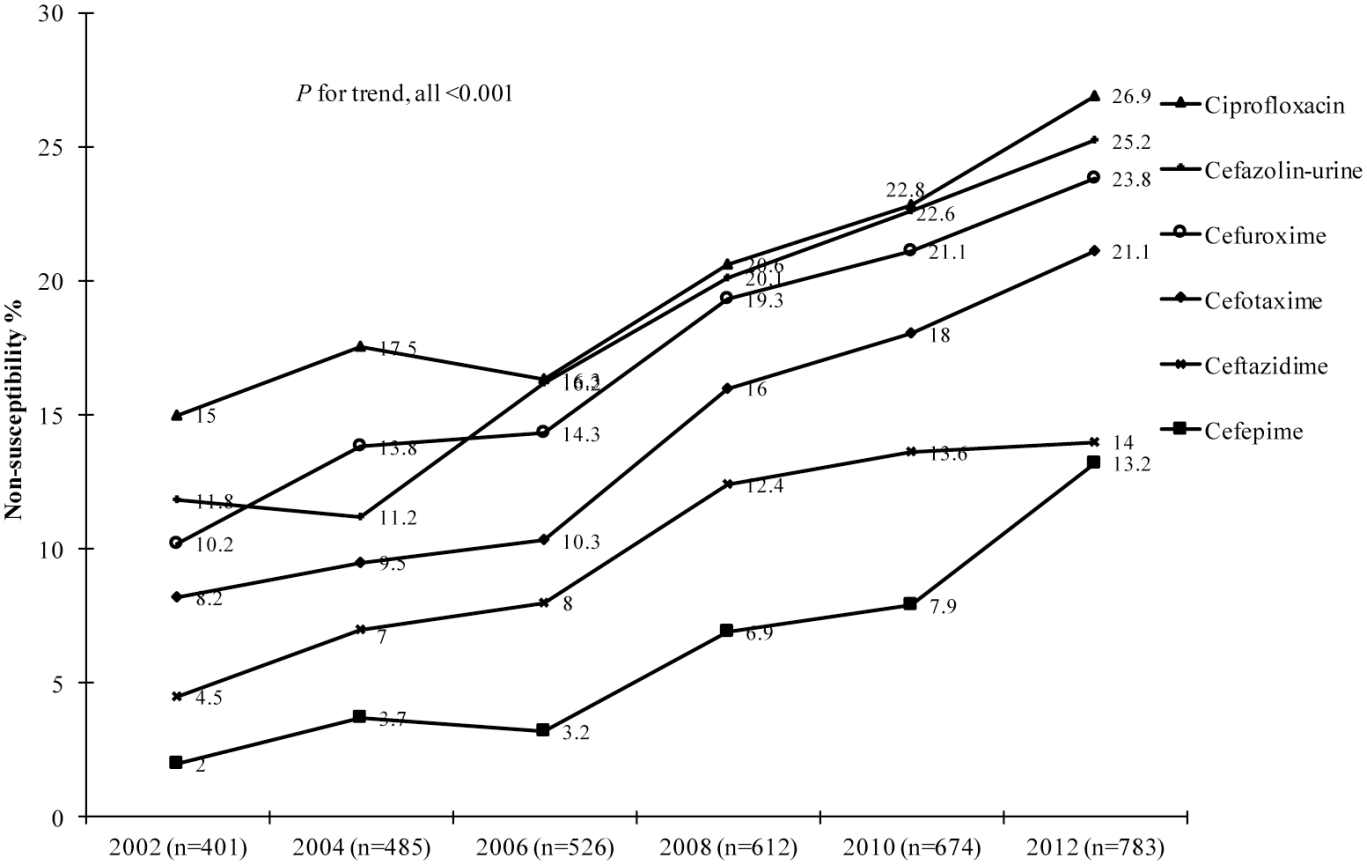
Increasing non-susceptibility (%) of *E*. *coli* to different agents from community setting, Taiwan, 2002–2012.

For ease of comparison, we grouped the 6 study years into 3 periods, with period I, II, III corresponding to 2002–2004, 2006–2008, and 2010–2012, respectively. The rates of overall non-susceptibility of the isolates to various antimicrobial agents from the 3 studied periods are listed in Table 1. Significant increases in non-susceptibility to ciprofloxacin and several β-lactams, including first to fourth generation cephalosporins, aztreonam, and cefoxitin, were noted. In contrast, non-susceptibility to tetracycline and trimethoprim/sulfamethoxazole (SXT) decreased but still remained high, at 59.1% (2006–2008) and 47.2% (2010–2012), respectively. Rates of non-susceptibility to all 4 generations of cephalosporins and ciprofloxacin by each study year are delineated in Fig 1, which highlights the increasing rates (all *P* < 0.001) of non-susceptibility over the study years. For example, the overall non-susceptibility to cefotaxime and ciprofloxacin increased from 8.2% and 15% in 2002 to 21.1% and 26.9% in 2012, respectively. Non-susceptibility to cefazolin of isolates from urine also increased from 11.5% in 2002 to 23.9% in 2012. The activities of the different antimicrobial agents against all 3481 *E*. *coli* isolates are presented in S1 File.

**Table 1:** Non-susceptibility to different agents and prevalence of ESBL and AmpC-β-lactamase producers in *E*. *coli* isolates from outpatient settings, 2002–2012^a^.

Antimicrobial agents^b^	Period I	Period II	Period III	*P* value^f^
	(2002–2004)	(2006–2008)	(2010–2012)	
	(n = 886)	(n = 1138)	(n = 1457)	
β-lactams:				
Amoxicillin/CA^c^	21.2	30.1	NT	<0.001
Ampicillin	71.2	70.6	69.6	NS
Aztreonam	6.4	9.9	15.7	<0.001
Cefazolin-Non-urine^d^	50.9	46.1	60.4	<0.001
Cefazolin-Urine^d^	11.5	18.1	23.9	<0.001
Cefuroxime	12.2	17.0	22.5	<0.001
Cefoxitin	10.2	14.8	17.0	<0.001
Cefotaxime	8.9	13.4	19.6	<0.001
Ceftazidime	6.1	10.4	13.9	<0.001
Cefepime– 2013	1.2	3.8	7.8	<0.001
Cefepime^e^	2.9	5.1	10.6	<0.001
Ertapenem^c^	NT^c^	NT	0.8	-
Imipenem	0.1	0.2	0.1	NS
Piperacillin^c^	69.4	69.6	65.4	0.038
Pip/tazo^b^,^c^	NT	4.6	3.9	NS
Non-β-lactams:				
Amikacin	1.6	1.1	0.8	NS
Gentamicin	26.6	25.8	24.1	NS
Ciprofloxacin	16.4	18.6	25.1	<0.001
Fosfomycin^c^	NT	NT	0.7	-
Nitrofurantoin^c^	8.3	4.8	7.8	NS
SXT^b^	55.1	50.1	47.2	0.001
Tetracycline^c^	64.8	59.1	NT	0.01
Tigecycline^c^	NT	NT	0	-
ESBL/AmpC β-lactamase positive:				
ESBL	4.0	4.5	10.7	<0.001
AmpC	5.3	9.1	10.7	<0.001

^a^ The study was conducted biennially between 2002 and 2012. We grouped the study years into 3 peroids for ease of comparison, with period 1 from 2002 and 2004, period II from 2006 and 2008, and period III from 2010 and 2012.

^b^ Based on the 2014 CLSI breakpoints unless indicated otherwise. CA, clavulanic acid; Pip/tazo, piperacillin/tazobactam; SXT, trimethoprim/sulfamethoxazole.

^c^ These agents were tested on years indicated only: amoxicillin/clavulanate (2002–2008), ertapenem (2012, n = 783), fosfomycin [2012 urine isolates (n = 461)], nitrofurantoin [urine isolates in 2002–2004 (n = 601), 2006 (n = 352), and 2012], piperacillin [2002–2008, and 2010 (n = 674)], tetracycline (2002–2008), tigecycline (2010 & 2012); NT, not tested.

^d^ Separate cefazolin breakpoints for non-urine and urine isolates were used.

^e^ Including the 2014 susceptible dose dependent category (SDD) for cefepime.

^f^ Chi square for trend analysis.

Isolates from different specimen types generally had similar rates of non-susceptibility, with except that isolates from blood had lower non-susceptibility than urine isolates to piperacillin (66.0% vs. 75.2%, *P* = 0.028), piperacillin/tazobactam (2.4% vs. 5.5%, *P* < 0.001), and ciprofloxacin (18.2% vs. 21.9%, *P* = 0.039) (Table 2). However, significant difference in rates of non-susceptibility to most tested agents existed in isolates from the three age groups, with isolates from elderly patients having the highest rates of non-susceptibility than those from adult and pediatric patients. These included (elderly vs. adult) all 4 generations of cephalosporins (using cefotaxime as example: 20.0% vs. 10.6%, P <0.001), cefoxitin (19.8% vs. 10.1%, P < 0.001), and non-β-lactams amikacin (1.6% vs. 0.6%, P = 0.002), gentamicin (29.6% vs. 21.2%, P < 0.001), and ciprofloxacin (27.6% vs. 15.2%, P < 0.001) (Table 2). Although isolates from elderly patients had the highest rates of non-susceptibility to most agents, the increase of non-susceptibility to extended-spectrum cephalosporins occurred in isolates from all age groups. Of note, rates of non-susceptibility of pediatric isolates were either higher than or similar to those from adult patients, except ciprofloxacin, which differed slightly (10.7% vs. 15.2%, *P* = 0.067). In addition, isolates from pediatric patients had significantly higher rate of non-susceptibility to amoxicillin/clavulanate than those from the adult patients (30.7% vs. 22.4%, *P* = 0.014).

**Table 2:** Non-susceptibility (%) of outpatient *E*. *coli* stratified by specimen types, age groups, and geographic regions (2002–2012 combined).

Antimicrobial agents^a^	Specimen types				Age groups				Geographic regions				
	Blood (n = 1125)	Urine (n = 2153)	Others (n = 203)	*P* ^c^	Pediatric (n = 234)	Adult (n = 1563)	Elderly (n = 1610)	*P* ^c^	North (n = 1091)	Central (n = 979)	South (n = 1004)	East (n = 407)	*P* ^c^
**β-lactams:**													
Amoxicillin/CA^b^	24.6 (586)	27.5 (1307)	20.6 (131)	NS	30.7 (150)	22.4 (913)	29.9 (892)	0.001	22.9 (625)	32.7 (556)	25.0 (601)	23.1 (242)	<0.001
Ampicillin	68.7	70.7	75.4	NS	73.5	68.3	72.2	NS	66.4	74.4	71.3	68.8	<0.001
Aztreonam	11.6	11.5	9.8	NS	6.0	8.1	15.7	<0.001	8.8	13.7	13.0	9.1	<0.001
Cefazolin	53.9	55.2	52.7	NS	55.1	49.5	60.1	<0.001	50.2	57.5	57.0	54.1	0.003
Cefazolin-urine		18.6		-	15.7 (209)	12.0 (1001)	26.5 (892)	<0.001	14.4 (716)	25.6 (570)	19.1 (643)	12.1 (224)	<0.001
Cefuroxime	18.5	18.0	16.7	NS	12.0	12.9	24.0	<0.001	14.5	22.1	20.1	13.0	<0.001
Cefoxitin	14.3	15.0	10.8	NS	8.5	10.1	19.8	<0.001	13.5	16.3	16.0	9.3	0.003
Cefotaxime	15.2	14.9	12.8	NS	8.5	10.6	20.0	<0.001	11.5	19.1	16.1	10.6	<0.001
Ceftazidime	11.2	10.7	8.9	NS	5.6	7.6	14.6	<0.001	8.5	13.1	12.4	7.1	<0.001
Cefepime	6.8	7.0	6.4	NS	3.8	5.2	9.1	<0.001	4.4	10.4	7.2	4.7	<0.001
Piperacillin	66.0	70.4	75.2	0.028	72	68.4	69.9	NS	66.1	73.2	69.0	67.2	0.04
Pip/tazo^b^	2.4	5.5	1.0	0.001	2.9	3.2	5.3	0.046	3.0	6.0	4.7	1.3	0.005
**Non-β-lactams:**													
Amikacin	1.0	1.2	0.5	NS	0	0.6	1.6	0.002	1.0	1.5	0.9	0.5	NS
Gentamicin	23.4	26.0	28.1	NS	23.6	21.2	29.6	<0.001	21.9	27.4	27.6	23.6	0.006
Ciprofloxacin	18.2	21.9	22.2	0.039	10.7	15.2	27.6	<0.001	17.7	23.0	23.2	17.4	<0.001
SXT^b^	49.1	50.4	53.2	NS	52.6	45.9	53.7	<0.001	46.8	52.6	52.4	47.4	0.016
**ESBL/AmpC prevalence:**													
ESBL	6.9	7.0	6.4	NS	3.9	4.9	9.4	<0.001	4.6	10.4	7.1	4.7	<0.001
AmpC	9.7	8.5	6.4	NS	3.9	5.9	12.4	<0.001	7.0	10.7	10.1	5.9	0.002

^a^ Not all agents were tested on all isolates in all years. For those agents not tested on all isolates, the numbers of isolates tested in each stratum are shown below the non-susceptibility percentages. Data on fosfomycin, nitrofurantoin, and tetracycline are not shown in this Table because there were no significant differences in rates of non-susceptibility between the variables in each stratum.

^b^ CA, clavulanic acid; Pip/tazo, piperacillin/tazobactam; SXT, trimethoprim/sulfamethoxazole.

^c^ P value of overall distribution among three groups; NS, Not significant (p >0.05). The post-hoc analysis results of specimen and age categories are presented in Results section.

Rates of non-susceptibility also differed significantly between isolates from the 4 geographic regions, with those from central and southern regions having the higher rates of non-susceptibility while those from the northern and eastern regions were lower (Table 2). For example, non-susceptibility to cefotaxime was 11.5%, 19.1%, 16.1%, and 10.6%, and to gentamicin was 21.9%, 27.4%, 27.6%, and 23.6% in isolates from the northern, central, southern, and eastern regions, respectively. These differences are also reflected in the prevalence of ESBL and AmpC–producers (Table 2).

**Fig 2:**
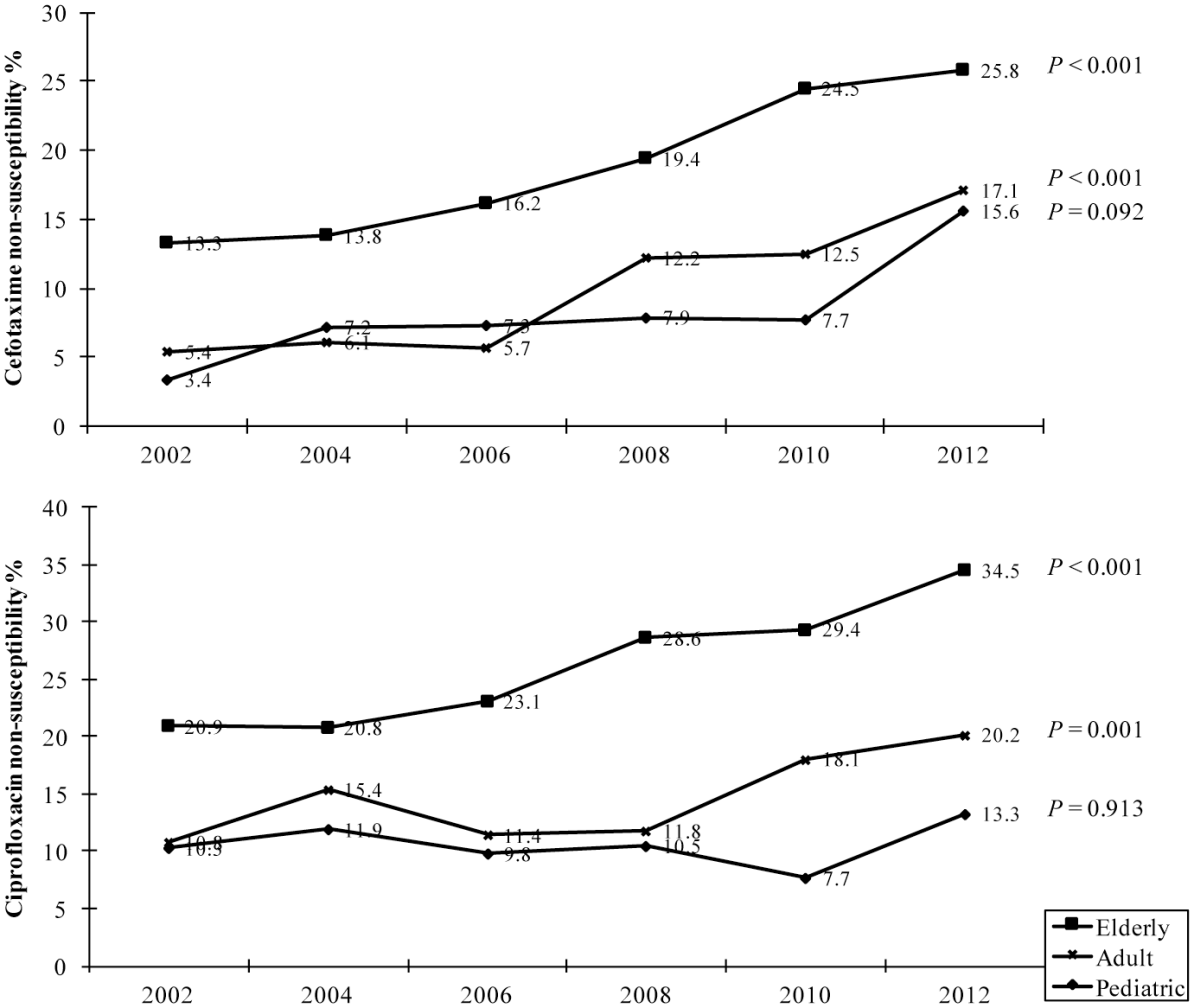
Non-susceptibility (%) of outpatient *E*. *coli* from different age groups to cefotaxime (top) and ciprofloxacin (bottom), Taiwan, 2002–2012. Pediatric, ≤18 y.o.; adult, 19–64 y.o.; elderly, ≥ 65 y.o. The number of isolates for each age group in 2002, 20024, 2006, 2008, 2010, and 2012 was 29, 42, 41, 38, 39, and 45, respectively for pediatric patients; 186, 228, 245, 254, 304, and 346, respectively for adult patients; and 158, 211, 229, 294, 326, and 392, respectively, for elderly patients.

**Fig 3:**
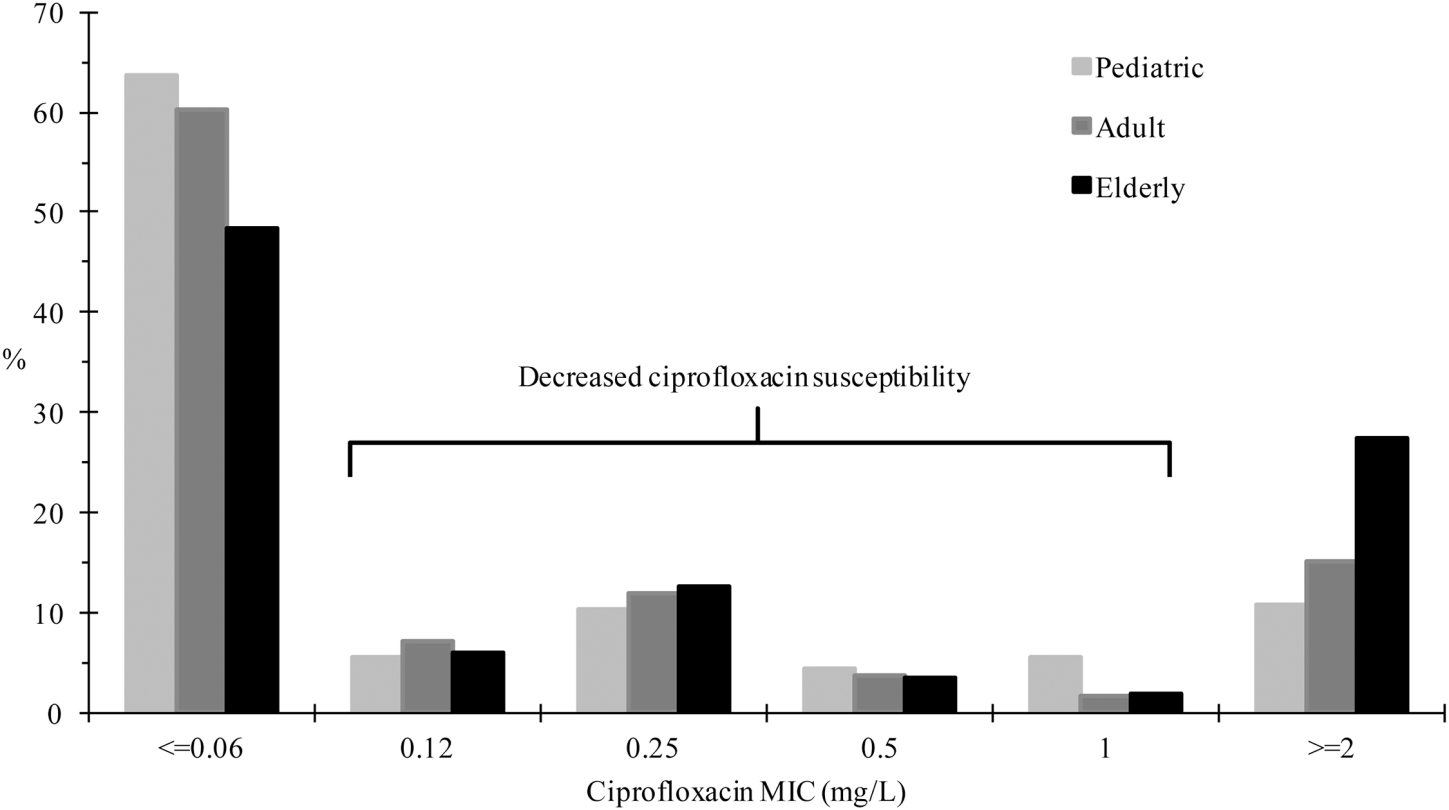
Ciprofloxacin MIC distribution of outpatient *E*. *coli* isolates from different age groups. Data were based on 234 isolates from pediatric, 1563 isolates from adult, and 1610 isolates from elderly patients.

Between 2002 and 2012, non-susceptibility to cefotaxime increased from 3.4 to 15.6% (P = 0.092), 5.4 to 17.1% (*P* < 0.001), and 13.3 to 25.8% (*P* < 0.001) in isolates from pediatric, adult, and elderly patients, respectively (Fig 2). Significant increase in ciprofloxacin non-susceptibility also occurred in isolates from adult (from 10.8% to 20.2%, *P* = 0.001) and elderly (from 20.9% to 34.5%, *P* < 0.001) patients (Fig 2). However, based on ciprofloxacin MIC distribution, decreased ciprofloxacin susceptibility (DCS) (considered those with ciprofloxacin MIC 0.12–1 mg/L) of isolates from pediatric patients was as high (25.8%) as those from adult (24.8%) and elderly (24.0%) patients (Fig 3).

Four isolates had low-level imipenem non-susceptibility [2 isolates had MIC 2 ug/mL (intermediate) and 2 isolates had MIC 4 ug/mL (resistant)]. Carbapenemase (IMP-type) was detected in only 1 of these isolates (data not shown). All isolates were susceptible to tigecycline. Non-susceptibility of the urine isolates to nitrofurantoin remained below 8% over the study years, while only 0.7% (3/461) was nonsusceptible to fosfomycin in the 2012 collection we studied. For the 2153 urine isolates, rates of non-susceptibility to cefazolin differed significantly using the 2014 non-urine (55.2%) and urine interpretive criteria (18.6%) (*P* <0.001). The new 2014 cefepime breakpoints had a small but significant effect on the overall non-susceptibility (old breakpoints: 4.8% vs. 2014 breakpoints: 6.9%, average of all 6 study years, P <0.001).

### Prevalence, types, and susceptibilities of ESBL and AmpC β-lactamases producers

There were 547 isolates with aztreonam, ceftazidime, and/or cefotaxime MIC ≥ 2 mg/L. These isolates were subject to ESBL phenotypic confirmatory test plus genotypic detection of ESBL and AmpC β-lactamase genes by PCR. Among these 547 isolates, 190 were positive for ESBL genes only (ESBL-pos/AmpC-neg), 254 were positive for AmpC genes only (ESBL-neg/AmpC-pos), and 52 were positive for both ESBL and AmpC genes (ESBL-pos/AmpC-pos) by PCR. Of note, 16 of the 52 PCR ESBL-pos/AmpC-pos isolates had negative ESBL phenotypic confirmatory test. Therefore, the overall false-negative ESBL phenotypic confirmatory test rate was 6.6% (16/242).

Based on PCR results, the prevalence of ESBL-producers remained similar in earlier years (4.0% in 2002–2004 and 4.5% in 2006–2008) but increased to 10.7% in 2010–2012 (P < 0.001), whereas AmpC-producers increased steadily from 5.3% in 2002–2004, to 9.1% in 2006–2008 and 10.7% in 2010–2012 (P < 0.001) (Table 1). Among the 242 ESBL-producers, 233 (96.3%) carried CTX-M-type genes, and 12 carried SHV-type genes including 3 that carried both CTX-M- and SHV -type genes. Of the 306 AmpC-producers, 295 (96.4%) carried CMY-type genes, 11 carried DHA-type genes, including 7 that carried both CMY- and DHA- type genes. Compared to isolates that were negative for both ESBL and AmpC by PCR (ESBL-neg/AmpC-neg), isolates positive for either ESBL and/or AmpC were significantly more non-susceptible to most β-lactam as well as non-β-lactam agents (Table 3).

**Table 3:** Non-susceptibility^a^ (%) to key agents among *E*. *coli* isolates with and without ESBL or AmpC β-lactamases genes.

Antimicrobial agents^b^	Combination of ESBL/AmpC gene status			
	+/+ (n = 52)	+/- (n = 190)	-/+ (n = 254)	-/- (n = 2985)
Amikacin	23.1	7.4	2.8	0.1
Aztreonam	98.1	73.7	74.4	0.6
Cefoxitin	100	29.5	99.2	4.9
Cefotaxime	100	100	97.2	0.9
Ceftazidime	100	35.3	94.5	0.5
Cefepime (R/SDD)^c^	76.9/15.4	64.7/25.8	0.8/5.5	0.03/0.1
Ciprofloxacin	94.2	73.7	63.0	12.5
Gentamicin	84.6	62.2	57.9	19.1

^a^ Non-susceptibility results are based on the 2014 CLSI interpretive criteria.

^b^ Data shown on selected agents that were tested on all isolates.

^c^ Cefepime results are shown in resistant and susceptible dose dependent (SDD) categories.

Using the current CLSI interpretive criteria, all 242 ESBL-positive isolates were non-susceptible to cefotaxime, and all but 7 of the 254 ESBL-neg/AmpC-pos isolates were also non-susceptible. Therefore, the sensitivity, specificity, positive predictive value (PPV), and negative predictive value (NPV) of cefotaxime non-susceptibility in predicting the presence of ESBL and/or AmpC -producers was 98.6%, 99.1%, 94.6%, and 99.8%, respectively. However, 137 (27.6%) of these 496 ESBL and/or AmpC-positive isolates remained susceptible to ceftazidime so the sensitivity, specificity, PPV, and NPV of ceftazidime non-susceptibility in predicting the presence of ESBL and/or AmpC–producers was 72.4%, 99.5%, 96.0%, and 95.6%, respectively, even with the revised lower CLSI breakpoints [21]. Of note, significant difference in cefepime non-susceptibility existed in ESBL-positive isolates (92.3% of ESBL-pos/AmpC-pos and 90.5% of ESBL-pos/AmpC-neg isolates) and ESBL-neg/AmpC-pos isolates (6.3%), which included 15.4%, 25.8%, and 5.5% of the ESBL-pos/AmpC-pos, ESBL-pos/AmpC-neg, and ESBL-neg/AmpC-pos isolates with cefepime MIC in the 2014 new susceptible-dose dependent category (Table 3) [21].

### Statistical analysis to identify factors associated with ESBL and AmpC-β-lactamase producers

By univariate analysis, factors associated with carriage of ESBL and AmpC included age, geographic origin of bacterial isolates, and TSAR study period (Table 4). By multivariate analysis, age (elderly vs. pediatric patients), geographic origin (central Taiwan vs. eastern Taiwan) and later study years (2010 and 2012 vs. 2002) remained independent factors associated with ESBL gene carriage (Table 4). Age (elderly vs. pediatric patients), geographic origin (southern, central Taiwan vs. eastern Taiwan) and study period (2006 to 2012 vs. 2002) were also independent factors associated with carriage of AmpC β-lactamase (Table 4).

**Table 4:** Factors associated with carriage of ESBL and AmpC β-lactamases genes in *E*. *coli*.

Strata	ESBL				AmpC β-lactamase			
	*P* ^a^	OR^b^	95%CI^b^	*P* ^b^	*P* ^a^	OR^b^	95%CI^b^	*P* ^b^
Study year (using TSAR III [2002] as baseline)								
TSAR IV (2004)	0.456	0.77	0.39–1.52	NS	0.116	1.64	0.88–3.05	NS
TSAR V (2006)	0.135	0.55	0.27–1.13	NS	0.031	1.85	1.02–3.38	0.044
TSAR VI (2008)	0.287	1.31	0.73–2.35	NS	<0.001	2.68	1.52–4.72	<0.001
TSAR VII (2010)	0.019	1.81	1.05–3.14	0.034	<0.001	3.30	1.90–5.74	<0.001
TSAR VIII (2012)	<0.001	3.02	1.79–5.10	<0.001	0.003	2.21	1.26–3.87	0.006
Age groups (using Pediatric as baseline)								
Adult patients	0.776	0.98	0.54–1.78	NS	0.354	1.27	0.71–2.27	NS
Elderly patients	0.007	1.93	1.09–3.42	0.024	<0.001	2.84	1.62–4.98	<0.001
Specimen types (using urine as baseline)								
Blood	0.932				0.277			
Others	0.744				0.295			
Geographic region (using eastern region as baseline)								
Northern	0.98	1.02	0.59–4.27	NS	0.461	1.24	0.77–2.00	NS
Central	<0.001	2.56	1.53–1.76	<0.001	0.006	1.99	1.25–3.17	0.004
Southern	0.097	1.63	0.96–2.75	NS	0.014	1.80	1.13–2.86	0.014

^a^ *P* value by univariate analysis. Only variables having statistical significant difference by univariate analysis were subject to multivariate analysis.

^b^ *P* value by multivariate analysis; OR, odds ratio; CI, confidence interval

## Discussion

The present study analyzed data from a nationwide surveillance program and revealed that over the past decade (2002 to 2012), significant increase in non-susceptibility to most β-lactam agents and ciprofloxacin occurred in *E*. *coli* isolates from community settings (outpatients and patients visiting emergency rooms) in Taiwan. In addition, non-susceptibility of our 2010–2012 isolates to several agents were higher than those from similar time periods in western countries, including cefazolin (59.7% against 27.7% in Canada), cefotaxime (19.6% against lower than 10% in Canada, USA, and Europe, and lower than 7.4% in UK based on non-susceptibility to 3^rd^ generation cephalosporins, gentamicin (24,1% against 9.8% in Canada and lower than 6% in UK), and trimethoprim/sulfamethoxazole (47.2% against lower than 27.4% in Canada and Europe), even though some of those studies included isolates from inpatients [8, 19, 20, 30].

The increased non-susceptibility to nearly all broad-spectrum β-lactams (except imipenem) in our isolates correlated with the increased ESBL- and AmpC β-lactamase-producers, which increased to 10.7% for both, respectively, in 2010–2012. Although increased prevalence of ESBL-producing *E*. *coli* has been noted in several countries [31], the 10.7% prevalence of ESBL and AmpC -producers in our 2010–2012 isolates was higher than recent reports on isolates from community settings in USA (ESBL: 5.9%, AmpC-producers: around 0.6%), and Canada (AmpC: 2.6%), and from combination of community and healthcare-associated settings in Canada (ESBL: 5.4%) and northern European countries (ESBL: around 5%, AmpC: 0–3.8%) [8, 11, 12, 15, 20, 30–33].

In the present study, most ESBL genes were CTX-M types (96.3%), and the predominant AmpC β-lactamases genes was the CMY type (90.7%). Prior studies have shown that the most prevalent ESBL and AmpC enzymes types worldwide, including Taiwan, were CTX-M and CMY types, respectively [34–36]. Since only a few isolates had low level non-susceptibility to ertapenem and/or imipenem. Carbapenemase (IMP-type) was detected only in 1 isolate (data not shown). Thus carbapenemase producing *E*. *coli* appeared not to be a major problem in community settings in Taiwan yet.

CLSI lowered the susceptibility breakpoints of third-generation cephalosporins for *Enterobacteriaceae* in 2010 to facilitate the identification of ESBL- and/or AmpC- β-lactamases -producing isolates [37]. By applying the revised breakpoints, nearly all (98.7%) of our ESBL and/or AmpC positive isolates were non-susceptible to cefotaxime, yet only 77.6% of them were non-susceptible to ceftazidime. Thus cefotaxime is a better predictor for ESBLs and/or AmpC β-lactamase-producers than ceftazidime in terms of sensitivity (98.7% vs. 77.6%). These results are similar to prior studies [23, 38, 39], and reflects the predominance of CTX-M since this enzyme hydrolyzes cefotaxime more efficiently than ceftazidime [40].

Of noteworthy is that 16 of the 52 PCR ESBL-pos/AmpC-pos isolates in the present study tested negative by the CLSI phenotypic ESBL confirmatory test so the overall false-negative phenotypic ESBL confirmatory test rate was 6.6% (16/242). Co-carriage of ESBL and AmpC β-lactamase genes has become common among Enterobacteriaceae in our region [23, 36]. It has been demonstrated that the presence of AmpC β-lactamases could mask the underlying ESBLs by traditional phenotypic confirmatory test [41]. Our result echoes this finding and lends support to using the revised CLSI breakpoints to avoid missing ESBL-producers by the phenotypic ESBL confirmatory test.

Based on the 2014 revised cefepime breakpoints, over 90% of the ESBL producers are non-susceptible to cefepime including 23.6% with cefepime MIC in the susceptible dose dependent range, indicating that higher dosing regimens are needed if cefepime was used for these isolates [21]. Significant difference on non-susceptibility to cefazolin in the non-urine (60.4%) vs. urine isolates (23.9%) from the 2010–2012 isolates exists using the 2014 cefazolin non-urine and urine breakpoints. This information is important in antibiotic selection for uncomplicated UTI also.

Non-susceptibility to ciprofloxacin in our 2010–2012 isolates (25.1%) was much higher than that of community *E*. *coli* isolates from the 15.5% (UTI hospital and community isolates combined from 2013) reported in UK [19] and other countries in Europe (0.5–7.6% in 2007–2008) [20], but was lower than the rate (32.4%) found by a multicenter surveillance of 2009–2011 *E*. *coli* from inpatients with community-acquired UTI in USA [8]. Whether such differences were dependent on patient age is unknown since isolates from elderly patients in our study had the highest rate of ciprofloxacin non-susceptibility, which reached 34.5% in 2010–2012.

Isolates from recent years were more likely to carry ESBL and AmpC β-lactamase genes, and these isolates were most likely to be found in elderly patients as well as those from central or southern Taiwan (compared to those from eastern Taiwan). Elderly patients probably had more recent exposure to medical care and antibiotics, resided in long-term care facilities, which have been associated with acquisition of drug-resistant bacteria [15, 42–44]. This in turn would explain why isolates from elderly patients were more resistant than those from adult and pediatric patients. The reason for isolates from central and southern Taiwan being more likely to carry ESBL and AmpC β-lactamase genes, and thus more non-susceptible to most agents, needs further investigation.

We also showed that isolates from pediatric patients had higher rates of non-susceptibility to amoxicillin/clavulanic acid than those from adults and elderly. In addition, the pediatric isolates had similarly high rate (near 25%) of decreased ciprofloxacin susceptibility (DCS, ciprofloxacin MIC 0.12–1 mg/L), probably due to single mutation in the quinolone-resistance-determining region of the drug target or the presence of plasmid-mediated quinolone resistance determinants [45–47]. This is a cause for additional concern since the effectiveness of FQ in treating patients infected with DCS isolates may be compromised [47–49]. The reasons for the high rates of DCS and amoxicillin/clavulanic acid in the pediatric population needs further study.

Prior reports have pointed out that inappropriate empirical therapy was associated with added mortality among critically ill patients with severe infection [4, 50]. Therefore it has been suggested that the empirical antibiotic therapy for patients with severe infection should cover more than 90% spectrum of all the possible bacterial etiology [51]. For community uncomplicated UTI, the suggested prevalence of resistance threshold above which the agent is not recommended was 20% for SXT and 10% for fluoroquinolones [5]. Given our study results, cefotaxime might no longer be a reasonable empirical antibiotic for patients with severe community-acquired infections in Taiwan if *E*. *coli* is a probable etiologic agent since around 20% of the isolates from 2010–2012 were cefotaxime-resistant. Similarly, fluoroquinolones are also not appropriate empirical therapy option since non-susceptibility to ciprofloxacin reached 25% in 2010–2012. Instead, ertapenem or cefepime should be considered as the drugs of choice in such situation.

A recent study indicated that nitrofurantoin, an old antimicrobial agent, retained its activity against multidrug resistant *E*. *coli* isolates from the USA, with resistance observed ranging 2.1% to 24.1% of isolates resistant to three to five antimicrobial agents, respectively, with an overall resistance of only 1.2% [9]. Our data showed that nitrofurantoin non-susceptibility in urinary *E*. *coli* isolates from Taiwan remained at lower than 8% over the years. Non-susceptibility to fosfomycin was low (lower than 1%) in our 2012 urinary *E*. *coli* isolates. Therefore these 2 older antimicrobial agents could be options for the treatment of uncomplicated cystitis as suggested [5].

One limitation of the present study is that since we only have minimum clinical information on the source patients, we could not determine if other independent factors, such as prior antimicrobial use or hospitalization, exist for ESBL and/or AmpC–producers [15, 42, 43]. However, we did show age (elderly) to be an independent factor for ESBL and AmpC–producers, thus prior hospitalization and more antimicrobial use are likely associated factors. Another limitation is that our isolates were collected biennially during a three months period. However, these isolates were from a total of 28 hospitals located in all four geographical regions of Taiwan, and 25 of them, including 11 medical centers and 14 regional hospitals, participated in all 6 rounds of TSAR between 2002 and 2012. Therefore the results described here can be a representation of the *E*. *coli* from community settings in Taiwan.

In conclusion, this decade-long multicenter surveillance revealed significant increase in non-susceptibility to several broad spectrum antimicrobial agents, including cefotaxime and ciprofloxacin, occurred in *E*. *coli* from community settings in Taiwan. Therefore, multidrug resistance is prevalent not only in healthcare settings but also in the community in our region. The prevalence of ESBL- and AmpC β-lactamase-producing *E*. *coli* also increased significantly, especially in elderly patients. Whether such increase was due to clonal spread or horizontal gene transfer requires further study. The additional high rates of decreased ciprofloxacin susceptibility in *E*. *coli* isolates from all age groups are also a cause for concern. Identification of community reservoirs of the resistant bacteria may help to halt their spread. Finally, our data indicated that there is a need to re-evaluate appropriate treatment selection for community-acquired infections.

## Supporting Information

S1 File Dataset of 3481 *Escherichia coli* isolates from outpatients and patients visiting emergency rooms in Taiwan, 2002–2012. (PDF)

## References

[1] Kaper JB, Nataro JP, Mobley HL. Pathogenic *Escherichia coli* . Nat Rev Microbiol. 2004;2(2):123–40. Epub 2004/03/26. 10.1038/nrmicro818 . 15040260

[2] Foxman B. The epidemiology of urinary tract infection. Nat Rev Urol. 2010;7(12):653–60. 10.1038/nrurol.2010.190 . 21139641

[3] WHO. Technical consultation: strategies for global surveillance of antimicrobial resistance. http://apps.who.int/iris/bitstream/10665/90975/1/WHO_HSE_PED_2013.10358_eng.pdf. (2 March 2015, date last accessed).2013. Available from: http://apps.who.int/iris/bitstream/10665/90975/1/WHO_HSE_PED_2013.10358_eng.pdf.

[4] Harbarth S, Garbino J, Pugin J, Romand JA, Lew D, Pittet D. Inappropriate initial antimicrobial therapy and its effect on survival in a clinical trial of immunomodulating therapy for severe sepsis. Am J Med. 2003;115(7):529–35. 14599631 10.1016/j.amjmed.2003.07.005

[5] Hooton TM. Clinical practice. Uncomplicated urinary tract infection. N Engl J Med. 2012;366(11):1028–37. 10.1056/NEJMcp1104429 22417256

[6] Solomkin JS, Mazuski JE, Bradley JS, Rodvold KA, Goldstein EJ, Baron EJ, et al Diagnosis and management of complicated intra-abdominal infection in adults and children: guidelines by the Surgical Infection Society and the Infectious Diseases Society of America. Clin Infect Dis. 2010;50(2):133–64. 10.1086/649554 . 20034345

[7] Theuretzbacher U, Van Bambeke F, Canton R, Giske CG, Mouton JW, Nation RL, et al Reviving old antibiotics. J Antimicrob Chemother. 2015;70(8):2177–81. 10.1093/jac/dkv157 . 26063727

[8] Bouchillon SK, Badal RE, Hoban DJ, Hawser SP. Antimicrobial susceptibility of inpatient urinary tract isolates of gram-negative bacilli in the United States: results from the study for monitoring antimicrobial resistance trends (SMART) program: 2009–2011. Clin Ther. 2013;35(6):872–7. 10.1016/j.clinthera.2013.03.022 . 23623624

[9] Sanchez GV, Baird AM, Karlowsky JA, Master RN, Bordon JM. Nitrofurantoin retains antimicrobial activity against multidrug-resistant urinary *Escherichia coli* from US outpatients. J Antimicrob Chemother. 2014;69(12):3259–62. 10.1093/jac/dku282 . 25063776

[10] Allocati N, Masulli M, Alexeyev MF, Di Ilio C. *Escherichia coli* in Europe: an overview. Int J Environ Res Public Health. 2013;10(12):6235–54. 10.3390/ijerph10126235 24287850 PMC3881111

[11] Hanson ND, Moland ES, Hong SG, Propst K, Novak DJ, Cavalieri SJ. Surveillance of community-based reservoirs reveals the presence of CTX-M, imported AmpC, and OXA-30 β-lactamases in urine isolates of *Klebsiella pneumoniae* and *Escherichia coli* in a U.S. community. Antimicrob Agents Chemother. 2008;52(10):3814–6. 10.1128/aac.00877-08 PubMed Central PMCID: PMC2565870. 18663030 PMC2565870

[12] Tansarli GS, Poulikakos P, Kapaskelis A, Falagas ME. Proportion of extended-spectrum β-lactamase (ESBL)-producing isolates among Enterobacteriaceae in Africa: evaluation of the evidence—systematic review. J Antimicrob Chemother. 2014;69(5):1177–84. 10.1093/jac/dkt500 24398340

[13] Pitout JD. Enterobacteriaceae that produce extended-spectrum β-lactamases and AmpC β-lactamases in the community: the tip of the iceberg? Curr Pharm Des. 2013;19(2):257–63. 22934977

[14] Balode A, Punda-Polic V, Dowzicky MJ. Antimicrobial susceptibility of gram-negative and gram-positive bacteria collected from countries in Eastern Europe: results from the Tigecycline Evaluation and Surveillance Trial (T.E.S.T.) 2004–2010. Int J Antimicrob Agents. 2013;41(6):527–35. 10.1016/j.ijantimicag.2013.02.022 23590898

[15] Pascual V, Ortiz G, Simo M, Alonso N, Garcia MC, Xercavins M, et al Epidemiology and risk factors for infections due to AmpC β-lactamase-producing *Escherichia coli* . J Antimicrob Chemother. 2015;70(3):899–904. 10.1093/jac/dku468 25468902

[16] Zhang J, Zheng B, Zhao L, Wei Z, Ji J, Li L, et al Nationwide high prevalence of CTX-M and an increase of CTX-M-55 in *Escherichia coli* isolated from patients with community-onset infections in Chinese county hospitals. BMC Infect Dis. 2014;14:659 10.1186/s12879-014-0659-0 PubMed Central PMCID: PMC4265337. 25466590 PMC4265337

[17] Bouchillon S, Hoban DJ, Badal R, Hawser S. Fluoroquinolone resistance among gram-negative urinary tract pathogens: global SMART program results, 2009–2010. Open Microbiol J. 2012;6:74–8. 10.2174/1874285801206010074 PubMed Central PMCID: PMC3447161. 23002406 PMC3447161

[18] European Center for Disease Prevention and Control. http://ecdc.europa.eu/en/healthtopics/antimicrobial_resistance/database/Pages/graph_reports.aspx. European Center for Disease Prevention and Control. 2015.

[19] Ironmonger D, Edeghere O, Bains A, Loy R, Woodford N, Hawkey PM. Surveillance of antibiotic susceptibility of urinary tract pathogens for a population of 5.6 million over 4 years. J Antimicrob Chemother. 2015;70(6):1744–50. 10.1093/jac/dkv043 . 25733586

[20] Kahlmeter G, Poulsen HO. Antimicrobial susceptibility of *Escherichia coli* from community-acquired urinary tract infections in Europe: the ECO.SENS study revisited. Int J Antimicrob Agents. 2012;39(1):45–51. 10.1016/j.ijantimicag.2011.09.013 . 22055529

[21] CLSI. Performance Standards for Antimicrobial Susceptibility Testing: 24^th^ Informational Supplement M100-S24. CLSI, Wayne, PA, USA,. 2014.

[22] Kuo SC, Chen PC, Shiau YR, Wang HY, Lai JFs, Huang W, et al Levofloxacin-resistant *Haemophilus influenzae*, Taiwan, 2004–2010. Emerg Infect Dis. 2014;20(8):1386–90. 10.3201/eid2008.140341 PubMed Central PMCID: PMC4111205. 25061696 PMC4111205

[23] Wang JT, Chen PC, Chang SC, Shiau YR, Wang HY, Lai JF, et al Antimicrobial susceptibilities of *Proteus mirabilis*: a longitudinal nationwide study from the Taiwan surveillance of antimicrobial resistance (TSAR) program. BMC Infect Dis. 2014;14:486 10.1186/1471-2334-14-486 25192738 PMC4162950

[24] CLSI. Abbreviated Identification of Bacteria and Yeast; Approved Guideline—Second Edition M35-A2. CLSI, Wayne, PA, USA 2008.

[25] Edberg SC, Trepeta RW. Rapid and economical identification and antimicrobial susceptibility test methodology for urinary tract pathogens. J Clin Microbiol. 1983;18(6):1287–91. PubMed Central PMCID: PMC272894. 6361049 10.1128/jcm.18.6.1287-1291.1983 PMC272894

[26] Monstein HJ, Ostholm-Balkhed A, Nilsson MV, Nilsson M, Dornbusch K, Nilsson LE. Multiplex PCR amplification assay for the detection of *bla* _SHV_, *bla* _TEM_ and *bla* _CTX-M_ genes in Enterobacteriaceae. APMIS. 2007;115(12):1400–8. 10.1111/j.1600-0463.2007.00722.x . 18184411

[27] Perez-Perez FJ, Hanson ND. Detection of plasmid-mediated AmpC β-lactamase genes in clinical isolates by using multiplex PCR. J Clin Microbiol. 2002;40(6):2153–62. 12037080 10.1128/JCM.40.6.2153-2162.2002 PMC130804

[28] Queenan AM, Bush K. Carbapenemases: the versatile β-lactamases. Clin Microbiol Rev. 2007;20(3):440–58, table of contents. 10.1128/cmr.00001-07 17630334 PMC1932750

[29] O'Brien TF, Stelling J. Integrated multilevel surveillance of the world's infecting microbes and their resistance to antimicrobial agents. Clin Microbiol Rev. 2011;24(2):281–95. 10.1128/CMR.00021-10 21482726 PMC3122493

[30] Karlowsky JA, Adam HJ, Baxter MR, Lagace-Wiens PR, Walkty AJ, Hoban DJ, et al In vitro activity of ceftaroline-avibactam against gram-negative and gram-positive pathogens isolated from patients in Canadian hospitals from 2010 to 2012: results from the CANWARD surveillance study. Antimicrob Agents Chemother. 2013;57(11):5600–11. 10.1128/aac.01485-13 23979759 PMC3811279

[31] Oteo J, Perez-Vazquez M, Campos J. Extended-spectrum β-lactamase producing *Escherichia coli*: changing epidemiology and clinical impact. Curr Opin Infect Dis. 2010;23(4):320–6. . 20614578 10.1097/qco.0b013e3283398dc1

[32] Brolund A. Overview of ESBL-producing Enterobacteriaceae from a Nordic perspective. Infect Ecol Epidemiol. 2014;4 10.3402/iee.v4.24555 PubMed Central PMCID: PMC4185132. PMC4185132 25317262

[33] Carbonne A, Arnaud I, Maugat S, Marty N, Dumartin C, Bertrand X, et al National multidrug-resistant bacteria (MDRB) surveillance in France through the RAISIN network: a 9 year experience. J Antimicrob Chemother. 2013;68(4):954–9. 10.1093/jac/dks464 23194721

[34] D'Andrea MM, Arena F, Pallecchi L, Rossolini GM. CTX-M-type β-lactamases: a successful story of antibiotic resistance. Int J Med Microbiol. 2013;303(6–7):305–17. 10.1016/j.ijmm.2013.02.008 23490927

[35] Hawkey PM, Jones AM. The changing epidemiology of resistance. J Antimicrob Chemother. 2009;64 Suppl 1:i3–10. Epub 2009/08/19. 10.1093/jac/dkp256 19675017

[36] Sheng WH, Badal RE, Hsueh PR. Distribution of extended-spectrum β-lactamases, AmpC β-lactamases, and carbapenemases among Enterobacteriaceae isolates causing intra-abdominal infections in the Asia-Pacific region: results of the study for Monitoring Antimicrobial Resistance Trends (SMART). Antimicrob Agents Chemother. 2013;57(7):2981–8. 10.1128/aac.00971-12 23587958 PMC3697370

[37] Dudley MN, Ambrose PG, Bhavnani SM, Craig WA, Ferraro MJ, Jones RN. Background and rationale for revised clinical and laboratory standards institute interpretive criteria (Breakpoints) for Enterobacteriaceae and *Pseudomonas aeruginosa*: I. Cephalosporins and Aztreonam. Clin Infect Dis. 2013;56(9):1301–9. 10.1093/cid/cit017 23334813

[38] Hawser SP, Badal RE, Bouchillon SK, Hoban DJ, Hsueh PR. Comparison of CLSI 2009, CLSI 2010 and EUCAST cephalosporin clinical breakpoints in recent clinical isolates of *Escherichia coli*, *Klebsiella pneumoniae* and *Klebsiella oxytoca* from the SMART Global Surveillance Study. Int J Antimicrob Agents. 2010;36(3):293–4. 10.1016/j.ijantimicag.2010.05.012 20598511

[39] Tan TY, Ng LS, He J, Chen DM. Improved detection of AmpC β-lactamases in *Escherichia coli*, *Klebsiella pneumoniae*, and *Proteus mirabilis* using new susceptibility breakpoints for third-generation cephalosporins. Diagn Microbiol Infect Dis. 2011;70(3):423–4. 10.1016/j.diagmicrobio.2011.03.008 21549542

[40] Rossolini GM, D'Andrea MM, Mugnaioli C. The spread of CTX-M-type extended-spectrum β-lactamases. Clin Microbiol Infect. 2008;14 Suppl 1:33–41. 10.1111/j.1469-0691.2007.01867.x 18154526

[41] Poulou A, Grivakou E, Vrioni G, Koumaki V, Pittaras T, Pournaras S, et al Modified CLSI extended-spectrum β-lactamase (ESBL) confirmatory test for phenotypic detection of ESBLs among Enterobacteriaceae producing various β-lactamases. J Clin Microbiol. 2014;52(5):1483–9. Epub 2014/02/28. 10.1128/jcm.03361-13 PubMed Central PMCID: PMC3993656. 24574283 PMC3993656

[42] Ben-Ami R, Rodriguez-Bano J, Arslan H, Pitout JD, Quentin C, Calbo ES, et al A multinational survey of risk factors for infection with extended-spectrum β-lactamase-producing Enterobacteriaceae in nonhospitalized patients. Clin Infect Dis. 2009;49(5):682–90. Epub 2009/07/23. 10.1086/604713 . 19622043

[43] Lee CH, Lee YT, Kung CH, Ku WW, Kuo SC, Chen TL, et al Risk factors of community-onset urinary tract infections caused by plasmid-mediated AmpC β-lactamase-producing Enterobacteriaceae. J Microbiol Immunol Infect. 2015;48(3):269–75. Epub 2013/11/19. 10.1016/j.jmii.2013.08.010 24239065

[44] Rooney PJ, O'Leary MC, Loughrey AC, McCalmont M, Smyth B, Donaghy P, et al Nursing homes as a reservoir of extended-spectrum β-lactamase (ESBL)-producing ciprofloxacin-resistant *Escherichia coli* . J Antimicrob Chemother. 2009;64(3):635–41. Epub 2009/06/25. 10.1093/jac/dkp220 . 19549667

[45] McDonald LC, Chen FJ, Lo HJ, Yin HC, Lu PL, Huang CH, et al Emergence of reduced susceptibility and resistance to fluoroquinolones in *Escherichia coli* in Taiwan and contributions of distinct selective pressures. Antimicrob Agents Chemother. 2001;45(11):3084–91. 10.1128/aac.45.11.3084-3091.2001 PubMed Central PMCID: PMC90786. 11600360 PMC90786

[46] Piddock LJ. Mechanisms of fluoroquinolone resistance: an update 1994–1998. Drugs. 1999;58 Suppl 2:11–8. 10553699 10.2165/00003495-199958002-00003

[47] Strahilevitz J, Jacoby GA, Hooper DC, Robicsek A. Plasmid-mediated quinolone resistance: a multifaceted threat. Clin Microbiol Rev. 2009;22(4):664–89. 10.1128/cmr.00016-09 PubMed Central PMCID: PMC2772364. 19822894 PMC2772364

[48] Labreche MJ, Frei CR. Declining susceptibilities of gram-negative bacteria to the fluoroquinolones: effects on pharmacokinetics, pharmacodynamics, and clinical outcomes. Am J Health Syst Pharm. 2012;69(21):1863–70. 10.2146/ajhp110464 23111670

[49] Poirel L, Pitout JD, Calvo L, Rodriguez-Martinez JM, Church D, Nordmann P. In vivo selection of fluoroquinolone-resistant *Escherichia coli* isolates expressing plasmid-mediated quinolone resistance and expanded-spectrum β-lactamase. Antimicrob Agents Chemother. 2006;50(4):1525–7. 10.1128/aac.50.4.1525-1527.2006 PubMed Central PMCID: PMC1426952. 16569874 PMC1426952

[50] Valles J, Rello J, Ochagavia A, Garnacho J, Alcala MA. Community-acquired bloodstream infection in critically ill adult patients: impact of shock and inappropriate antibiotic therapy on survival. Chest. 2003;123(5):1615–24. 12740282 10.1378/chest.123.5.1615

[51] The American Thoracic Society and Infectious Diseases Society of America. Guidelines for the management of adults with hospital-acquired, ventilator-associated, and healthcare-associated pneumonia. Am J Respir Crit Care Med. 2005;171(4):388–416. 10.1164/rccm.200405-644ST 15699079

