# Supplementary material for: Antimicrobial Non-Susceptibility of Escherichia coli from Outpatients and Patients Visiting Emergency Rooms in Taiwan
Source: PLoS One. 2015 Dec 3;10(12):e0144103. doi: 10.1371/journal.pone.0144103 (PMC4669119; doi:10.1371/journal.pone.0144103)
Supplement: S1 File — (PDF) [file pone.0144103.s001.pdf]

S1. Dataset of 3481 *Escherichia coli* isolates from outpatients and patients visiting emergency rooms in Taiwan, 2002 - 2012.

TSAR	Specimen#	Specimen	Age	AMK_M	AMP_M	ATM_M	AUG_M	CAZ_M	CFZ_M	CIP_M
III	2002-S01-006	Blood	80	<=2	2	<=0.5	4	<=0.5	<=2	<=0.03
III	2002-S01-008	Blood	75	>32	>16	>16	8	>16	>16	>2
III	2002-S01-011	Urine	70	<=2	>16	8	>16	16	>16	<=0.03
III	2002-S01-020	Urine	46	4	>16	<=0.5	8	<=0.5	<=2	0.5
III	2002-S01-022	Other	29	<=2	4	<=0.5	4	<=0.5	<=2	<=0.03
III	2002-S01-032	Urine	67	<=2	>16	<=0.5	16	<=0.5	>16	<=0.03
III	2002-S01-038	Urine	67	<=2	>16	<=0.5	8	<=0.5	<=2	0.12
III	2002-S01-040	Urine	47	4	8	<=0.5	8	<=0.5	<=2	<=0.03
III	2002-S01-042	Urine	72	<=2	>16	<=0.5	8	<=0.5	4	<=0.03
III	2002-S01-048	Urine	63	<=2	4	<=0.5	8	<=0.5	<=2	<=0.03
III	2002-S01-049	Urine	55	<=2	>16	<=0.5	8	<=0.5	4	>2
III	2002-S01-050	Urine	51	4	<=1	<=0.5	4	<=0.5	<=2	<=0.03
III	2002-C01-009	Urine	35	<=2	>16	<=0.5	8	<=0.5	<=2	<=0.03
III	2002-C01-011	Urine	21	<=2	>16	<=0.5	8	<=0.5	<=2	0.12
III	2002-C01-020	Urine	19	<=2	>16	<=0.5	4	<=0.5	<=2	1
III	2002-C01-027	Other	32	<=2	>16	<=0.5	16	<=0.5	16	<=0.03
III	2002-C01-028	Urine	64	8	>16	<=0.5	8	<=0.5	4	>2
III	2002-C01-029	Urine	75	<=2	>16	<=0.5	8	<=0.5	8	0.12
III	2002-C01-032	Urine	66	16	>16	<=0.5	16	<=0.5	>16	0.5
III	2002-C01-035	Urine	68	<=2	>16	<=0.5	8	<=0.5	<=2	0.5
III	2002-C01-037	Urine	75	<=2	>16	<=0.5	8	<=0.5	<=2	<=0.03
III	2002-C01-038	Urine	73	<=2	2	<=0.5	4	<=0.5	4	<=0.03
III	2002-C01-039	Urine	16	<=2	8	<=0.5	8	<=0.5	4	<=0.03
III	2002-C01-040	Urine	2	4	>16	<=0.5	8	<=0.5	<=2	<=0.03
III	2002-C01-042	Urine	77	<=2	>16	16	>16	16	>16	<=0.03
III	2002-C01-043	Urine	57	<=2	4	<=0.5	4	<=0.5	<=2	<=0.03
III	2002-C02-001	Urine	58	<=2	>16	<=0.5	8	<=0.5	4	<=0.03
III	2002-C02-005	Urine	0	4	>16	<=0.5	8	<=0.5	4	<=0.03
III	2002-C02-006	Urine	44	<=2	4	<=0.5	4	<=0.5	<=2	<=0.03
III	2002-C02-008	Urine	60	<=2	>16	<=0.5	8	<=0.5	4	1
III	2002-C02-030	Other	39	<=2	>16	<=0.5	8	<=0.5	<=2	<=0.03
III	2002-C02-041	Urine	64	<=2	4	<=0.5	4	<=0.5	<=2	<=0.03
III	2002-C02-042	Urine	70	<=2	4	<=0.5	8	<=0.5	4	<=0.03
III	2002-C02-043	Urine	48	<=2	>16	<=0.5	8	<=0.5	<=2	<=0.03
III	2002-C02-048	Urine	64	<=2	>16	<=0.5	8	<=0.5	<=2	<=0.03
III	2002-C04-016	Urine	60	<=2	>16	<=0.5	16	<=0.5	<=2	0.12
III	2002-C04-018	Blood	85	<=2	>16	<=0.5	8	<=0.5	<=2	<=0.03
III	2002-C04-028	Other	59	<=2	>16	<=0.5	8	<=0.5	4	<=0.03
III	2002-C04-047	Other	84	<=2	8	<=0.5	8	1	<=2	>2
III	2002-C04-050	Urine	37	<=2	8	<=0.5	8	<=0.5	<=2	0.25
III	2002-C04-209	Blood	88	4	2	<=0.5	4	<=0.5	<=2	<=0.03
III	2002-C04-224	Blood	67	<=2	4	<=0.5	8	<=0.5	<=2	0.25
III	2002-C04-225	Blood	89	<=2	>16	<=0.5	8	<=0.5	<=2	<=0.03
III	2002-C04-242	Urine	80	4	>16	>16	>16	16	>16	>2
III	2002-S05-004	Urine	87	<=2	>16	<=0.5	8	<=0.5	4	0.12
III	2002-S05-005	Urine	56	8	>16	<=0.5	8	<=0.5	<=2	0.25
III	2002-S05-006	Urine	44	<=2	>16	<=0.5	8	<=0.5	<=2	0.12
III	2002-S05-007	Urine	56	<=2	8	<=0.5	8	<=0.5	<=2	<=0.03
III	2002-S05-023	Urine	79	<=2	2	<=0.5	2	<=0.5	<=2	1
III	2002-S05-024	Urine	47	<=2	2	<=0.5	2	<=0.5	<=2	<=0.03
III	2002-S05-031	Other	40	4	>16	<=0.5	8	<=0.5	4	0.5
III	2002-S05-035	Urine	60	<=2	>16	<=0.5	4	<=0.5	<=2	0.12
III	2002-S05-036	Blood	66	<=2	2	<=0.5	4	<=0.5	<=2	<=0.03
III	2002-S05-039	Urine	0	<=2	>16	<=0.5	8	<=0.5	<=2	0.12
III	2002-S05-045	Urine	20	<=2	>16	<=0.5	8	<=0.5	<=2	0.5

III	2002-S06-001	Urine	74	16	>16	>16	>16	>16	>16	>2
III	2002-S06-002	Urine	72	<=2	>16	<=0.5	8	<=0.5	4	>2
III	2002-S06-003	Urine	42	<=2	>16	<=0.5	8	<=0.5	8	0.25
III	2002-S06-005	Urine	69	8	>16	<=0.5	8	<=0.5	8	<=0.03
III	2002-S06-009	Urine	46	<=2	>16	<=0.5	8	<=0.5	<=2	<=0.03
III	2002-S06-011	Urine	65	4	>16	<=0.5	8	<=0.5	8	<=0.03
III	2002-S06-014	Urine	70	<=2	>16	<=0.5	8	<=0.5	<=2	<=0.03
III	2002-S06-017	Urine	81	8	>16	<=0.5	8	<=0.5	4	>2
III	2002-S06-020	Urine	72	<=2	>16	<=0.5	4	<=0.5	<=2	0.5
III	2002-S06-021	Urine	23	<=2	>16	<=0.5	8	<=0.5	<=2	>2
III	2002-S06-023	Urine	70	4	>16	<=0.5	16	<=0.5	4	>2
III	2002-S06-024	Urine	19	4	>16	<=0.5	8	<=0.5	<=2	<=0.03
III	2002-S06-029	Urine	64	<=2	>16	<=0.5	8	<=0.5	4	1
III	2002-S06-030	Urine	0	<=2	4	<=0.5	4	<=0.5	<=2	<=0.03
III	2002-S06-032	Other	24	<=2	>16	<=0.5	8	<=0.5	<=2	0.25
III	2002-S06-034	Other	40	<=2	>16	<=0.5	8	<=0.5	<=2	0.5
III	2002-S06-218	Blood	76	<=2	>16	<=0.5	16	<=0.5	8	<=0.03
III	2002-S06-220	Blood	55	<=2	4	<=0.5	8	<=0.5	<=2	<=0.03
III	2002-S07-001	Urine	60	<=2	>16	<=0.5	16	<=0.5	<=2	<=0.03
III	2002-S07-002	Urine	73	8	4	<=0.5	4	<=0.5	<=2	<=0.03
III	2002-S07-003	Urine	42	<=2	>16	<=0.5	8	<=0.5	<=2	0.12
III	2002-S07-005	Urine	3	<=2	>16	<=0.5	16	<=0.5	8	0.12
III	2002-S07-009	Urine	82	4	<=1	<=0.5	2	<=0.5	<=2	<=0.03
III	2002-S07-014	Urine	46	<=2	4	<=0.5	4	<=0.5	<=2	<=0.03
III	2002-S07-015	Urine	46	<=2	2	<=0.5	1	<=0.5	<=2	0.5
III	2002-S07-019	Urine	74	<=2	2	<=0.5	2	<=0.5	<=2	0.12
III	2002-S07-021	Blood	44	4	>16	<=0.5	8	<=0.5	<=2	>2
III	2002-S07-028	Urine	64	4	>16	<=0.5	8	<=0.5	<=2	0.5
III	2002-S07-029	Urine	0	<=2	>16	16	>16	>16	>16	0.25
III	2002-S07-035	Blood	70	<=2	>16	<=0.5	8	<=0.5	<=2	0.12
III	2002-S07-036	Urine	11	4	4	<=0.5	4	<=0.5	<=2	>2
III	2002-S07-040	Urine	69	4	4	<=0.5	4	<=0.5	<=2	<=0.03
III	2002-S07-043	Other	46	<=2	>16	<=0.5	16	<=0.5	4	0.5
III	2002-S07-045	Urine	24	<=2	>16	<=0.5	8	<=0.5	<=2	0.06
III	2002-S07-046	Urine	75	8	>16	<=0.5	8	<=0.5	<=2	<=0.03
III	2002-S07-048	Urine	25	<=2	>16	<=0.5	8	<=0.5	<=2	<=0.03
III	2002-S07-050	Urine	24	<=2	4	<=0.5	4	<=0.5	<=2	<=0.03
III	2002-S07-204	Blood	69	<=2	4	<=0.5	8	<=0.5	<=2	<=0.03
III	2002-S07-218	Blood	60	<=2	>16	<=0.5	8	<=0.5	8	<=0.03
III	2002-S07-220	Blood	78	<=2	>16	<=0.5	8	<=0.5	<=2	<=0.03
III	2002-N05-003	Urine	unk	<=2	8	<=0.5	4	<=0.5	<=2	0.5
III	2002-N05-005	Urine		<=2	>16	<=0.5	8	<=0.5	<=2	0.25
III	2002-N05-006	Urine		<=2	>16	<=0.5	8	<=0.5	<=2	0.12
III	2002-N05-007	Urine		<=2	>16	<=0.5	8	<=0.5	<=2	<=0.03
III	2002-N05-009	Urine		<=2	>16	<=0.5	16	<=0.5	4	<=0.03
III	2002-N05-012	Urine		<=2	4	<=0.5	4	<=0.5	<=2	<=0.03
III	2002-N05-013	Urine		<=2	4	<=0.5	4	<=0.5	<=2	<=0.03
III	2002-N05-014	Urine		<=2	2	<=0.5	2	<=0.5	<=2	<=0.03
III	2002-N05-016	Urine		4	>16	<=0.5	16	<=0.5	8	<=0.03
III	2002-N05-017	Urine	unk	<=2	>16	2	16	<=0.5	>16	>2
III	2002-N05-021	Urine		<=2	>16	<=0.5	8	<=0.5	<=2	0.12
III	2002-N05-024	Urine		<=2	<=1	<=0.5	1	<=0.5	<=2	0.5
III	2002-N05-028	Urine		<=2	>16	<=0.5	8	<=0.5	4	<=0.03
III	2002-N05-032	Urine		<=2	4	<=0.5	8	<=0.5	<=2	0.12
III	2002-N05-036	Urine		<=2	>16	<=0.5	8	<=0.5	4	0.5
III	2002-N05-037	Urine		<=2	>16	<=0.5	8	<=0.5	<=2	<=0.03
III	2002-N05-044	Urine		<=2	2	<=0.5	4	<=0.5	<=2	<=0.03
III	2002-N05-208	Blood		<=2	4	<=0.5	8	<=0.5	<=2	<=0.03
III	2002-N06-009	Urine	47	<=2	>16	<=0.5	8	<=0.5	8	>2
III	2002-N06-020	Urine	51(89?)	<=2	>16	8	>16	>16	>16	<=0.03
III	2002-N06-033	Urine	7	4	4	<=0.5	4	<=0.5	<=2	0.25

III	2002-N06-039	Other	1	<=2	>16	<=0.5	8	<=0.5	<=2	<=0.03
III	2002-N06-041	Blood	68	<=2	>16	<=0.5	8	<=0.5	<=2	0.25
III	2002-N06-042	Blood	78	4	>16	<=0.5	8	<=0.5	4	0.25
III	2002-N06-043	Blood	82	4	>16	<=0.5	8	<=0.5	<=2	>2
III	2002-N06-258	Blood	54	<=2	4	<=0.5	4	<=0.5	<=2	<=0.03
III	2002-N06-269	Blood	79	>32	>16	16	16	4	>16	0.25
III	2002-N07-002	Urine	65	4	>16	2	16	1	>16	0.06
III	2002-N07-004	Urine	64	<=2	>16	<=0.5	8	<=0.5	<=2	0.25
III	2002-N07-009	Urine	88	>32	>16	>16	>16	>16	>16	>2
III	2002-N07-010	Urine	73	>32	>16	16	>16	>16	>16	>2
III	2002-N07-021	Urine	4	<=2	>16	<=0.5	8	<=0.5	4	<=0.03
III	2002-N07-025	Urine	29	<=2	>16	<=0.5	8	<=0.5	4	0.12
III	2002-N07-032	Urine	76	<=2	>16	<=0.5	>16	<=0.5	16	>2
III	2002-N07-033	Urine	34	4	>16	<=0.5	16	<=0.5	8	>2
III	2002-N07-042	Urine	41	<=2	4	<=0.5	8	<=0.5	<=2	0.06
III	2002-N07-044	Blood	77	8	4	<=0.5	4	<=0.5	<=2	<=0.03
III	2002-N07-045	Blood	73	<=2	>16	<=0.5	8	<=0.5	4	2
III	2002-N07-047	Blood	47	4	>16	<=0.5	8	<=0.5	<=2	<=0.03
III	2002-N07-048	Blood	76	<=2	>16	<=0.5	16	<=0.5	4	0.25
III	2002-N07-202	Blood	62	8	2	<=0.5	2	<=0.5	<=2	<=0.03
III	2002-S08-008	Other	34	<=2	2	<=0.5	4	<=0.5	<=2	<=0.03
III	2002-S08-010	Urine	unk	<=2	>16	<=0.5	8	<=0.5	<=2	0.25
III	2002-S08-016	Urine	69	<=2	>16	<=0.5	8	<=0.5	<=2	<=0.03
III	2002-S08-017	Urine	39	>32	>16	8	16	2	>16	>2
III	2002-S08-020	Other	1	<=2	>16	<=0.5	8	<=0.5	<=2	<=0.03
III	2002-S08-029	Blood	82	<=2	>16	<=0.5	16	<=0.5	16	<=0.03
III	2002-S08-030	Urine		4	>16	<=0.5	8	<=0.5	<=2	<=0.03
III	2002-S08-031	Urine	79	<=2	>16	<=0.5	8	<=0.5	4	<=0.03
III	2002-S08-032	Urine	47	<=2	>16	<=0.5	8	<=0.5	<=2	0.12
III	2002-S08-033	Urine	unk	<=2	>16	<=0.5	>16	<=0.5	>16	>2
III	2002-S08-038	Urine		<=2	>16	<=0.5	>16	<=0.5	>16	>2
III	2002-S08-041	Urine	59	4	>16	<=0.5	4	<=0.5	16	>2
III	2002-E02-002	Urine	50	<=2	>16	4	>16	8	>16	>2
III	2002-E02-009	Urine	80	<=2	<=1	<=0.5	1	<=0.5	<=2	0.06
III	2002-E02-010	Urine	90	<=2	4	<=0.5	4	<=0.5	<=2	<=0.03
III	2002-E02-012	Urine	36	<=2	>16	<=0.5	8	<=0.5	<=2	<=0.03
III	2002-E02-015	Urine	29	<=2	4	<=0.5	4	<=0.5	<=2	<=0.03
III	2002-E02-019	Urine	52	<=2	4	<=0.5	4	<=0.5	<=2	<=0.03
III	2002-E02-022	Other	32	<=2	>16	<=0.5	8	<=0.5	4	<=0.03
III	2002-E02-027	Urine	58	<=2	>16	<=0.5	16	<=0.5	8	<=0.03
III	2002-E02-035	Urine	77	<=2	4	<=0.5	4	<=0.5	<=2	<=0.03
III	2002-E02-036	Urine	60	<=2	>16	<=0.5	8	<=0.5	<=2	0.12
III	2002-E02-041	Urine	21	<=2	2	<=0.5	4	<=0.5	<=2	<=0.03
III	2002-E02-042	Urine	38	<=2	>16	<=0.5	>16	<=0.5	<=2	<=0.03
III	2002-E02-043	Other	51	<=2	16	<=0.5	16	<=0.5	<=2	<=0.03
III	2002-E02-049	Urine	85	4	>16	<=0.5	8	<=0.5	4	<=0.03
III	2002-E02-203	Blood	59	<=2	>16	<=0.5	8	<=0.5	4	<=0.03
III	2002-E02-214	Blood	59	<=2	>16	<=0.5	8	<=0.5	4	<=0.03
III	2002-E02-218	Blood	46	<=2	>16	<=0.5	8	<=0.5	4	<=0.03
III	2002-N08-004	Urine	24	<=2	<=1	<=0.5	1	<=0.5	<=2	<=0.03
III	2002-N08-005	Urine	24	<=2	>16	<=0.5	8	<=0.5	4	<=0.03
III	2002-N08-006	Urine	58	4	>16	2	8	<=0.5	>16	0.25
III	2002-N08-008	Urine	38	4	>16	<=0.5	16	<=0.5	4	>2
III	2002-N08-009	Urine	59	<=2	>16	2	>16	1	>16	<=0.03
III	2002-N08-020	Urine	39	<=2	4	1	4	<=0.5	<=2	<=0.03
III	2002-N08-022	Urine	46	<=2	>16	<=0.5	8	<=0.5	4	<=0.03
III	2002-N08-023	Urine	48	<=2	2	<=0.5	4	<=0.5	4	0.5
III	2002-N08-024	Blood	69	<=2	>16	<=0.5	8	<=0.5	<=2	<=0.03
III	2002-N08-025	Urine	30	<=2	>16	<=0.5	8	<=0.5	<=2	>2
III	2002-N08-029	Urine	21	<=2	>16	<=0.5	8	<=0.5	<=2	<=0.03
III	2002-N08-034	Urine	79	>32	>16	4	16	1	>16	0.25

III	2002-N08-035	Urine	41	<=2	4	<=0.5	4	<=0.5	<=2	0.12
III	2002-N08-037	Other	22	<=2	4	<=0.5	4	<=0.5	<=2	<=0.03
III	2002-N08-181	Other	1	4	>16	<=0.5	8	<=0.5	4	<=0.03
III	2002-N08-193	Urine	0	<=2	4	<=0.5	4	<=0.5	<=2	<=0.03
III	2002-N08-207	Blood	63	<=2	<=1	<=0.5	1	<=0.5	<=2	0.25
III	2002-N08-210	Blood	56	4	>16	<=0.5	8	1	4	0.12
III	2002-S09-001	Blood	62	<=2	8	<=0.5	8	<=0.5	<=2	0.12
III	2002-S09-002	Blood	37	<=2	>16	<=0.5	8	<=0.5	4	<=0.03
III	2002-S09-003	Urine	19	<=2	4	<=0.5	8	<=0.5	<=2	>2
III	2002-S09-006	Urine	69	<=2	>16	<=0.5	8	<=0.5	4	>2
III	2002-S09-013	Blood	81	<=2	>16	<=0.5	4	<=0.5	<=2	<=0.03
III	2002-S09-014	Urine	67	<=2	>16	<=0.5	8	<=0.5	<=2	0.12
III	2002-S09-016	Urine	47	<=2	4	<=0.5	4	<=0.5	<=2	0.25
III	2002-S09-019	Blood	54	<=2	2	<=0.5	4	<=0.5	<=2	0.12
III	2002-S09-021	Urine	87	4	>16	<=0.5	4	<=0.5	<=2	>2
III	2002-S09-023	Urine	82	4	4	<=0.5	4	<=0.5	<=2	>2
III	2002-S09-024	Urine	4	4	4	<=0.5	2	<=0.5	<=2	<=0.03
III	2002-S09-026	Urine	23	4	>16	<=0.5	8	<=0.5	<=2	0.12
III	2002-S09-033	Blood	67	4	>16	<=0.5	16	<=0.5	4	<=0.03
III	2002-S09-034	Blood	70	<=2	>16	<=0.5	8	<=0.5	<=2	0.06
III	2002-S09-037	Urine	89	4	>16	<=0.5	8	<=0.5	<=2	<=0.03
III	2002-S09-041	Blood	48	<=2	>16	8	>16	>16	>16	>2
III	2002-S09-043	Urine	23	<=2	>16	<=0.5	8	<=0.5	<=2	0.25
III	2002-S09-045	Urine	77	4	>16	<=0.5	8	<=0.5	4	<=0.03
III	2002-S09-050	Other	65	<=2	>16	<=0.5	8	<=0.5	<=2	0.12
III	2002-S09-201	Blood	71	4	>16	<=0.5	16	<=0.5	4	>2
III	2002-S09-206	Blood	76	<=2	<=1	<=0.5	<=0.5	<=0.5	<=2	<=0.03
III	2002-S09-211	Blood	57	8	>16	4	>16	16	>16	0.12
III	2002-S09-217	Blood	57	8	4	<=0.5	4	1	4	<=0.03
III	2002-S09-220	Blood	81	<=2	4	<=0.5	4	<=0.5	<=2	<=0.03
III	2002-E03-016	Other	62	<=2	>16	<=0.5	8	<=0.5	<=2	>2
III	2002-E03-039	Urine	78	<=2	>16	<=0.5	8	<=0.5	8	>2
III	2002-E03-042	Other	78	8	>16	<=0.5	8	<=0.5	4	0.12
III	2002-C06-007	Urine	0	<=2	4	<=0.5	4	<=0.5	<=2	<=0.03
III	2002-C06-008	Urine	21	<=2	>16	<=0.5	8	<=0.5	4	>2
III	2002-C06-021	Other	24	<=2	>16	<=0.5	4	<=0.5	<=2	<=0.03
III	2002-C06-022	Urine	18	4	>16	<=0.5	8	<=0.5	<=2	<=0.03
III	2002-C06-026	Blood	3(13?)	4	>16	<=0.5	8	<=0.5	<=2	0.25
III	2002-C06-028	Blood	82	<=2	>16	<=0.5	8	<=0.5	<=2	<=0.03
III	2002-C06-029	Blood	70	4	>16	8	>16	>16	>16	>2
III	2002-C06-035	Urine	92	<=2	>16	<=0.5	8	<=0.5	4	<=0.03
III	2002-C06-039	Urine	82	<=2	>16	<=0.5	8	<=0.5	<=2	<=0.03
III	2002-C06-040	Blood	71	<=2	>16	<=0.5	8	<=0.5	4	0.25
III	2002-C06-042	Blood	92	<=2	>16	4	>16	8	>16	0.25
III	2002-C06-043	Other	50	4	>16	<=0.5	16	<=0.5	>16	>2
III	2002-C06-047	Urine	78	<=2	>16	<=0.5	8	<=0.5	<=2	0.12
III	2002-C07-003	Blood	35	<=2	4	<=0.5	4	<=0.5	<=2	<=0.03
III	2002-C07-008	Other	46	<=2	>16	<=0.5	8	<=0.5	<=2	0.25
III	2002-C07-010	Urine	50	4	>16	<=0.5	8	<=0.5	<=2	0.12
III	2002-C07-011	Urine	81	<=2	>16	<=0.5	8	<=0.5	4	0.12
III	2002-C07-012	Blood	48	>32	>16	8	>16	2	>16	0.25
III	2002-N16-003	Blood		<=2	>16	<=0.5	16	<=0.5	4	<=0.03
III	2002-N16-004	Urine	72	<=2	>16	<=0.5	8	<=0.5	4	>2
III	2002-N16-023	Urine	83	<=2	>16	<=0.5	16	<=0.5	4	<=0.03
III	2002-N16-024	Urine	0	<=2	>16	<=0.5	16	<=0.5	8	>2
III	2002-N16-034	Urine	69	<=2	2	<=0.5	4	<=0.5	<=2	<=0.03
III	2002-N16-035	Urine	0	<=2	>16	<=0.5	16	<=0.5	16	<=0.03
III	2002-N16-041	Urine		<=2	>16	<=0.5	8	<=0.5	<=2	<=0.03
III	2002-N16-044	Urine		<=2	4	<=0.5	8	<=0.5	<=2	<=0.03
III	2002-N16-203	Blood		<=2	>16	<=0.5	4	<=0.5	<=2	<=0.03
III	2002-N16-209	Blood		<=2	>16	<=0.5	8	<=0.5	8	>2

III	2002-N17-004	Urine	46	<=2	>16	<=0.5	8	<=0.5	4	0.25
III	2002-N17-006	Urine	33	<=2	>16	<=0.5	8	<=0.5	<=2	0.25
III	2002-N17-007	Urine	76	4	>16	<=0.5	8	<=0.5	4	0.25
III	2002-N17-013	Urine	22	4	>16	<=0.5	8	<=0.5	4	0.5
III	2002-N17-020	Other	68	<=2	4	<=0.5	4	<=0.5	<=2	<=0.03
III	2002-N17-021	Urine	43	<=2	>16	>16	>16	>16	>16	<=0.03
III	2002-N17-023	Urine	47	<=2	>16	<=0.5	8	<=0.5	4	<=0.03
III	2002-N17-025	Urine	60	<=2	4	<=0.5	4	<=0.5	<=2	<=0.03
III	2002-N17-027	Other	91	<=2	>16	<=0.5	8	<=0.5	<=2	<=0.03
III	2002-N17-034	Urine	22	4	4	<=0.5	4	<=0.5	<=2	<=0.03
III	2002-N17-039	Urine	43	<=2	4	<=0.5	4	<=0.5	<=2	<=0.03
III	2002-N17-041	Urine	83	<=2	>16	<=0.5	8	<=0.5	4	<=0.03
III	2002-N17-047	Blood	47	<=2	<=1	<=0.5	1	<=0.5	<=2	0.12
III	2002-N17-048	Urine	4	<=2	>16	<=0.5	8	<=0.5	4	1
III	2002-N17-050	Urine	43	<=2	>16	<=0.5	8	<=0.5	<=2	<=0.03
III	2002-N19-001	Other	15	<=2	>16	<=0.5	8	<=0.5	<=2	<=0.03
III	2002-N19-002	Other	71	8	>16	<=0.5	8	<=0.5	4	<=0.03
III	2002-N19-004	Urine	57	4	>16	<=0.5	8	<=0.5	<=2	0.5
III	2002-N19-005	Urine	24	4	<=1	<=0.5	2	<=0.5	<=2	0.06
III	2002-N19-007	Urine	71	4	8	<=0.5	8	<=0.5	8	0.25
III	2002-N19-010	Urine	75	4	>16	<=0.5	8	<=0.5	16	>2
III	2002-N19-012	Urine	20	<=2	>16	<=0.5	8	<=0.5	<=2	0.12
III	2002-N19-014	Urine	84	4	>16	<=0.5	4	<=0.5	<=2	<=0.03
III	2002-N19-015	Urine	31	4	2	<=0.5	4	<=0.5	<=2	<=0.03
III	2002-N19-017	Other	20	<=2	>16	<=0.5	8	<=0.5	4	<=0.03
III	2002-N19-018	Urine	61	<=2	>16	<=0.5	8	<=0.5	4	<=0.03
III	2002-N19-021	Blood	83	<=2	<=1	<=0.5	1	<=0.5	<=2	<=0.03
III	2002-N19-022	Urine	25	4	>16	<=0.5	8	<=0.5	4	<=0.03
III	2002-N19-024	Other	28	<=2	>16	<=0.5	8	<=0.5	4	<=0.03
III	2002-N19-025	Urine	71	4	>16	<=0.5	4	<=0.5	<=2	0.12
III	2002-N19-026	Urine	37	<=2	2	<=0.5	4	<=0.5	<=2	0.12
III	2002-N19-028	Urine	46	4	4	<=0.5	4	<=0.5	<=2	<=0.03
III	2002-N19-030	Other	47	<=2	>16	<=0.5	4	<=0.5	<=2	0.25
III	2002-N19-033	Urine	33	<=2	>16	<=0.5	8	<=0.5	<=2	<=0.03
III	2002-N19-034	Urine	83	<=2	>16	<=0.5	8	<=0.5	<=2	0.5
III	2002-N19-035	Urine	80	<=2	4	<=0.5	4	<=0.5	<=2	<=0.03
III	2002-N19-037	Urine	69	<=2	>16	<=0.5	8	<=0.5	4	<=0.03
III	2002-N19-042	Blood	51	<=2	4	<=0.5	4	<=0.5	<=2	<=0.03
III	2002-N19-049	Urine	35	4	>16	<=0.5	8	<=0.5	<=2	<=0.03
III	2002-N19-203	Blood	86	4	>16	<=0.5	16	<=0.5	16	<=0.03
III	2002-N19-205	Blood	70	4	>16	>16	16	16	>16	0.12
III	2002-N19-206	Blood	72	<=2	>16	<=0.5	8	<=0.5	<=2	<=0.03
III	2002-N19-207	Blood	70	<=2	>16	<=0.5	8	<=0.5	<=2	2
III	2002-N19-208	Blood	74	<=2	4	<=0.5	4	<=0.5	<=2	>2
III	2002-N19-209	Blood	75	<=2	>16	<=0.5	8	<=0.5	<=2	>2
III	2002-C08-001	Other	56	<=2	>16	<=0.5	8	<=0.5	4	<=0.03
III	2002-C08-003	Urine	69	<=2	>16	<=0.5	16	<=0.5	4	0.12
III	2002-C08-006	Urine	58	4	>16	<=0.5	8	<=0.5	<=2	<=0.03
III	2002-C08-007	Other	35	4	>16	<=0.5	8	<=0.5	<=2	<=0.03
III	2002-C08-008	Urine	77	<=2	2	<=0.5	4	<=0.5	<=2	<=0.03
III	2002-C08-013	Other	22	<=2	>16	<=0.5	8	<=0.5	<=2	<=0.03
III	2002-C08-014	Urine	24	<=2	>16	<=0.5	16	<=0.5	<=2	<=0.03
III	2002-C08-015	Urine	70	<=2	>16	<=0.5	8	<=0.5	4	<=0.03
III	2002-C08-018	Urine	23	<=2	>16	<=0.5	16	<=0.5	8	0.25
III	2002-C08-021	Urine	71	<=2	>16	<=0.5	8	<=0.5	<=2	<=0.03
III	2002-C08-028	Urine	66	4	>16	2	16	<=0.5	>16	0.25
III	2002-C08-032	Urine	83	4	>16	2	8	1	>16	0.06
III	2002-C08-036	Other	32	4	>16	2	8	<=0.5	>16	0.12
III	2002-C08-037	Urine	72	<=2	4	<=0.5	8	<=0.5	4	<=0.03
III	2002-C08-040	Urine	72	<=2	4	<=0.5	4	<=0.5	<=2	<=0.03
III	2002-C08-041	Urine	38	<=2	>16	<=0.5	8	<=0.5	<=2	<=0.03

III	2002-C08-045	Blood	58	<=2	>16	<=0.5	16	<=0.5	8	<=0.03
III	2002-C08-048	Urine	59	<=2	>16	<=0.5	16	<=0.5	16	>2
III	2002-C08-049	Blood	37	4	>16	<=0.5	16	<=0.5	<=2	<=0.03
III	2002-C08-050	Blood	76	<=2	>16	<=0.5	16	<=0.5	16	<=0.03
III	2002-S11-001	Blood	81	<=2	>16	<=0.5	4	<=0.5	4	0.06
III	2002-S11-003	Urine	64	4	>16	<=0.5	8	<=0.5	<=2	<=0.03
III	2002-S11-004	Urine	59	4	>16	>16	>16	>16	>16	>2
III	2002-S11-008	Urine	33	<=2	>16	<=0.5	8	<=0.5	<=2	0.12
III	2002-S11-009	Urine	67	<=2	>16	<=0.5	8	<=0.5	4	0.25
III	2002-S11-010	Urine	42	<=2	2	<=0.5	4	<=0.5	<=2	<=0.03
III	2002-S11-013	Urine	60	32	>16	2	>16	16	>16	<=0.03
III	2002-S11-014	Urine	80	<=2	>16	16	>16	>16	>16	0.12
III	2002-S11-021	Urine	40	<=2	4	<=0.5	4	<=0.5	<=2	<=0.03
III	2002-S11-022	Urine	83	<=2	8	<=0.5	4	<=0.5	<=2	<=0.03
III	2002-S11-023	Urine	77	4	4	<=0.5	4	<=0.5	<=2	<=0.03
III	2002-S11-027	Urine	74	<=2	4	<=0.5	4	<=0.5	<=2	<=0.03
III	2002-S11-034	Blood	22	<=2	>16	<=0.5	8	<=0.5	4	0.25
III	2002-S11-037	Blood	78	<=2	>16	<=0.5	>16	<=0.5	>16	<=0.03
III	2002-S11-039	Urine	71	4	2	<=0.5	2	<=0.5	<=2	0.06
III	2002-S11-043	Urine	55	<=2	>16	<=0.5	4	<=0.5	<=2	<=0.03
III	2002-S11-044	Urine	23	<=2	>16	<=0.5	16	<=0.5	8	<=0.03
III	2002-S11-050	Urine	80	4	>16	<=0.5	8	<=0.5	4	>2
III	2002-S11-194	Urine	0	<=2	4	<=0.5	4	<=0.5	<=2	<=0.03
III	2002-S11-204	Blood	73	4	>16	16	16	4	>16	>2
III	2002-S11-215	Blood	77	<=2	>16	<=0.5	8	<=0.5	<=2	0.12
III	2002-S11-229	Blood	56	<=2	>16	<=0.5	8	<=0.5	4	>2
III	2002-N21-001	Other	81	<=2	>16	<=0.5	8	<=0.5	<=2	<=0.03
III	2002-N21-006	Urine	60	<=2	2	<=0.5	4	<=0.5	<=2	<=0.03
III	2002-N21-007	Urine	18	<=2	>16	<=0.5	16	<=0.5	8	<=0.03
III	2002-N21-013	Urine	21	4	4	<=0.5	4	<=0.5	<=2	<=0.03
III	2002-N21-014	Urine	80	4	>16	<=0.5	4	<=0.5	<=2	<=0.03
III	2002-N21-017	Other	2	<=2	>16	<=0.5	8	<=0.5	4	<=0.03
III	2002-N21-025	Urine	49	<=2	>16	<=0.5	>16	<=0.5	>16	0.12
III	2002-N21-029	Other	52	<=2	>16	<=0.5	8	<=0.5	<=2	0.25
III	2002-N21-035	Urine	52	<=2	>16	<=0.5	8	<=0.5	<=2	0.5
III	2002-N21-037	Urine	51	4	2	<=0.5	4	<=0.5	<=2	<=0.03
III	2002-N21-039	Urine	52	<=2	>16	<=0.5	8	<=0.5	4	>2
III	2002-N21-040	Urine	36	<=2	>16	<=0.5	8	<=0.5	4	1
III	2002-N21-045	Urine	52	<=2	4	<=0.5	4	<=0.5	<=2	<=0.03
III	2002-N21-047	Urine	42	<=2	2	<=0.5	2	<=0.5	<=2	<=0.03
III	2002-N21-050	Urine	75	<=2	>16	2	>16	2	>16	0.25
III	2002-N21-204	Blood	57	<=2	4	<=0.5	4	<=0.5	<=2	<=0.03
III	2002-N21-218	Blood	58	<=2	>16	<=0.5	8	<=0.5	<=2	<=0.03
III	2002-N21-219	Blood	51	<=2	>16	<=0.5	8	<=0.5	<=2	0.06
III	2002-C09-001	Urine	72	<=2	>16	<=0.5	8	<=0.5	4	0.5
III	2002-C09-008	Other	54	<=2	4	<=0.5	8	<=0.5	<=2	<=0.03
III	2002-C09-009	Blood	70	<=2	>16	<=0.5	16	<=0.5	<=2	<=0.03
III	2002-C09-013	Urine	47	<=2	4	<=0.5	4	<=0.5	<=2	<=0.03
III	2002-C09-016	Other	22	<=2	>16	<=0.5	4	<=0.5	<=2	0.25
III	2002-C09-017	Other	16	<=2	>16	<=0.5	16	<=0.5	<=2	<=0.03
III	2002-C09-020	Urine	0	4	>16	<=0.5	>16	<=0.5	>16	<=0.03
III	2002-C09-021	Urine	51	<=2	4	<=0.5	4	<=0.5	<=2	<=0.03
III	2002-C09-023	Urine	79	<=2	>16	<=0.5	8	<=0.5	<=2	>2
III	2002-C09-024	Urine	81	8	>16	>16	8	16	>16	>2
III	2002-C09-034	Urine	87	<=2	>16	<=0.5	16	<=0.5	4	<=0.03
III	2002-C09-036	Other	86	4	>16	<=0.5	8	<=0.5	8	>2
III	2002-C09-038	Other	0	<=2	4	<=0.5	4	<=0.5	<=2	<=0.03
III	2002-C09-039	Other	13	<=2	>16	<=0.5	8	<=0.5	4	>2
III	2002-C10-002	Urine	24	<=2	>16	<=0.5	8	<=0.5	<=2	0.12
III	2002-C10-003	Blood	66	<=2	>16	<=0.5	8	<=0.5	4	<=0.03
III	2002-C10-006	Urine	75	<=2	>16	<=0.5	8	<=0.5	<=2	<=0.03

III	2002-C10-007	Urine	74	<=2	>16	<=0.5	8	<=0.5	<=2	<=0.03
III	2002-C10-009	Blood	79	<=2	>16	<=0.5	8	<=0.5	<=2	0.5
III	2002-C10-010	Urine	1	<=2	>16	<=0.5	8	<=0.5	<=2	<=0.03
III	2002-C10-012	Urine	71	<=2	>16	<=0.5	16	<=0.5	8	<=0.03
III	2002-C10-013	Urine	41	<=2	>16	<=0.5	8	<=0.5	4	<=0.03
III	2002-C10-019	Urine	64	<=2	>16	<=0.5	8	<=0.5	<=2	0.5
III	2002-C10-026	Urine	72	4	>16	<=0.5	16	<=0.5	8	>2
III	2002-C10-029	Urine	68	<=2	<=1	<=0.5	1	<=0.5	<=2	<=0.03
III	2002-C10-031	Blood	74	<=2	4	<=0.5	8	<=0.5	<=2	<=0.03
III	2002-C10-032	Blood	85	<=2	2	<=0.5	4	<=0.5	<=2	<=0.03
III	2002-C10-034	Urine	41	<=2	>16	<=0.5	8	<=0.5	4	0.5
III	2002-C10-035	Urine	82	<=2	4	<=0.5	4	<=0.5	<=2	<=0.03
III	2002-C10-038	Blood	65	<=2	>16	<=0.5	16	<=0.5	4	0.25
III	2002-C10-041	Blood	75	<=2	2	<=0.5	4	<=0.5	<=2	<=0.03
III	2002-C10-044	Urine	80	8	>16	<=0.5	8	<=0.5	<=2	<=0.03
III	2002-C10-049	Urine	68	8	>16	2	16	1	>16	>2
III	2002-C10-202	Blood	27	<=2	>16	<=0.5	16	<=0.5	16	<=0.03
III	2002-C10-213	Blood	55	<=2	>16	<=0.5	8	<=0.5	4	<=0.03
III	2002-C10-214	Blood	48	4	>16	<=0.5	8	<=0.5	<=2	0.25
III	2002-E04-001	Other	46	<=2	>16	<=0.5	16	<=0.5	8	<=0.03
III	2002-E04-002	Urine	77	<=2	>16	<=0.5	8	<=0.5	<=2	<=0.03
III	2002-E04-011	Urine	66	<=2	>16	<=0.5	4	<=0.5	<=2	0.12
III	2002-E04-020	Urine	10	4	>16	<=0.5	8	<=0.5	4	0.5
III	2002-E04-022	Urine	51	<=2	4	<=0.5	4	<=0.5	<=2	<=0.03
III	2002-E04-023	Urine	66	<=2	>16	<=0.5	16	<=0.5	4	<=0.03
III	2002-E04-027	Urine	57	<=2	4	<=0.5	4	<=0.5	<=2	0.12
III	2002-E04-030	Blood	53	<=2	>16	<=0.5	8	<=0.5	<=2	<=0.03
III	2002-E04-032	Urine		<=2	2	<=0.5	4	<=0.5	<=2	<=0.03
III	2002-E04-033	Urine	76	4	>16	>16	16	2	>16	>2
III	2002-E04-037	Urine	35	8	>16	<=0.5	8	<=0.5	<=2	<=0.03
III	2002-E04-038	Blood	56	<=2	4	<=0.5	4	<=0.5	<=2	<=0.03
III	2002-E04-039	Blood	73	<=2	>16	<=0.5	8	<=0.5	<=2	<=0.03
III	2002-E04-040	Blood	80	<=2	>16	<=0.5	8	<=0.5	<=2	<=0.03
III	2002-E04-045	Urine	32	4	>16	<=0.5	8	<=0.5	<=2	<=0.03
III	2002-E04-046	Urine	75	<=2	>16	<=0.5	8	<=0.5	<=2	<=0.03
III	2002-E04-201	Blood	33	<=2	>16	<=0.5	8	<=0.5	<=2	0.25
III	2002-E04-202	Blood	65	<=2	>16	<=0.5	8	<=0.5	<=2	0.06
III	2002-E04-205	Blood	82	4	>16	<=0.5	8	<=0.5	4	>2
III	2002-E04-208	Blood	75	<=2	>16	<=0.5	8	<=0.5	<=2	>2
III	2002-E04-215	Blood	46	<=2	4	<=0.5	4	<=0.5	<=2	0.06
III	2002-E04-218	Blood	61	<=2	>16	<=0.5	8	<=0.5	8	<=0.03
IV	2004-S01-002	Blood	19	4	>16	<=0.5	8	<=0.5	<=2	<=0.03
IV	2004-S01-003	Blood	42	<=2	>16	<=0.5	16	<=0.5	4	0.25
IV	2004-S01-004	Blood	53	<=2	>16	<=0.5	8	<=0.5	16	>2
IV	2004-S01-009	Urine	62	<=2	4	<=0.5	4	<=0.5	<=2	<=0.03
IV	2004-S01-015	Urine	73	<=2	>16	<=0.5	8	<=0.5	4	1
IV	2004-S01-020	Urine	62	<=2	>16	<=0.5	16	<=0.5	8	<=0.03
IV	2004-S01-021	Urine	40	4	4	<=0.5	8	<=0.5	4	<=0.03
IV	2004-S01-023	Other	32	<=2	>16	<=0.5	8	<=0.5	4	<=0.03
IV	2004-S01-031	Urine	56	4	>16	<=0.5	8	<=0.5	4	<=0.03
IV	2004-S01-033	Urine	59	4	>16	<=0.5	16	<=0.5	8	1
IV	2004-S01-037	Other	43	8	>16	<=0.5	8	<=0.5	8	>2
IV	2004-S01-042	Urine	80	<=2	4	<=0.5	4	<=0.5	<=2	<=0.03
IV	2004-S01-044	Urine	33	<=2	4	<=0.5	4	<=0.5	<=2	<=0.03
IV	2004-S01-048	Urine	22	4	4	<=0.5	4	<=0.5	<=2	<=0.03
IV	2004-S01-050	Urine	53	<=2	4	<=0.5	4	<=0.5	<=2	<=0.03
IV	2004-S01-185	Urine	1	4	4	<=0.5	4	<=0.5	4	<=0.03
IV	2004-S01-212	Blood	60	<=2	4	<=0.5	8	<=0.5	4	<=0.03
IV	2004-C01-003	Blood	88	<=2	<=1	<=0.5	2	<=0.5	<=2	0.12
IV	2004-C01-005	Urine	78	<=2	>16	<=0.5	8	<=0.5	<=2	0.12
IV	2004-C01-007	Urine	50	<=2	>16	<=0.5	8	<=0.5	<=2	0.25

IV	2004-C01-010	Blood	52	4	>16	<=0.5	8	<=0.5	4	0.06
IV	2004-C01-013	Urine	49	<=2	>16	<=0.5	8	<=0.5	8	>2
IV	2004-C01-017	Other	49	4	>16	<=0.5	8	<=0.5	4	0.25
IV	2004-C01-025	Urine	0	<=2	2	<=0.5	2	<=0.5	<=2	<=0.03
IV	2004-C01-030	Urine	52	8	>16	<=0.5	8	<=0.5	<=2	<=0.03
IV	2004-C01-031	Urine	47	<=2	>16	<=0.5	8	<=0.5	4	0.25
IV	2004-C01-032	Urine	1	4	>16	<=0.5	8	<=0.5	4	<=0.03
IV	2004-C01-036	Urine	73	4	>16	<=0.5	8	<=0.5	4	0.25
IV	2004-C01-037	Urine	76	4	4	<=0.5	8	<=0.5	8	0.25
IV	2004-C01-038	Urine	23	<=2	>16	<=0.5	16	<=0.5	16	<=0.03
IV	2004-C01-041	Urine	63	4	2	<=0.5	4	<=0.5	<=2	<=0.03
IV	2004-C01-042	Urine	86	<=2	>16	<=0.5	8	<=0.5	>16	0.06
IV	2004-C01-043	Urine	78	<=2	4	<=0.5	8	<=0.5	<=2	<=0.03
IV	2004-C01-045	Urine	76	8	>16	<=0.5	16	<=0.5	4	0.25
IV	2004-C01-214	Blood	61	<=2	4	<=0.5	8	<=0.5	<=2	<=0.03
IV	2004-C02-002	Urine	4	<=2	>16	<=0.5	16	<=0.5	>16	>2
IV	2004-C02-007	Urine	29	8	>16	<=0.5	8	<=0.5	8	<=0.03
IV	2004-C02-008	Other	0	<=2	>16	2	>16	1	>16	1
IV	2004-C02-014	Urine	10	4	4	<=0.5	4	<=0.5	<=2	<=0.03
IV	2004-C02-015	Urine	58	<=2	>16	<=0.5	8	<=0.5	4	<=0.03
IV	2004-C02-020	Other	41	<=2	>16	<=0.5	8	<=0.5	4	0.12
IV	2004-C02-024	Urine	72	4	>16	>16	>16	>16	>16	>2
IV	2004-C02-026	Urine	88	4	4	<=0.5	4	<=0.5	<=2	<=0.03
IV	2004-C02-028	Urine	23	4	>16	<=0.5	8	<=0.5	<=2	0.25
IV	2004-C02-030	Urine	3	<=2	>16	4	>16	8	>16	<=0.03
IV	2004-C02-031	Urine	62	<=2	>16	<=0.5	16	<=0.5	8	1
IV	2004-C02-032	Urine	28	<=2	>16	<=0.5	16	<=0.5	8	1
IV	2004-C02-033	Urine	62	4	>16	>16	>16	>16	>16	2
IV	2004-C02-035	Urine	26	<=2	>16	<=0.5	8	<=0.5	<=2	0.12
IV	2004-C02-039	Urine	63	<=2	>16	<=0.5	8	<=0.5	<=2	0.25
IV	2004-C02-040	Urine	0	4	>16	<=0.5	4	<=0.5	<=2	<=0.03
IV	2004-C02-041	Urine	30	<=2	>16	<=0.5	16	<=0.5	>16	<=0.03
IV	2004-C02-043	Urine	77	<=2	>16	<=0.5	8	<=0.5	4	<=0.03
IV	2004-C02-201	Blood	51	4	>16	4	>16	>16	>16	>2
IV	2004-C02-217	Blood	66	<=2	>16	8	>16	4	>16	<=0.03
IV	2004-C04-006	Urine	65	4	>16	<=0.5	8	<=0.5	<=2	0.25
IV	2004-C04-016	Urine	30	<=2	>16	<=0.5	8	<=0.5	<=2	<=0.03
IV	2004-C04-017	Blood	94	4	>16	<=0.5	16	<=0.5	4	0.25
IV	2004-C04-019	Other	56	<=2	8	<=0.5	4	<=0.5	4	<=0.03
IV	2004-C04-020	Blood	82	4	4	<=0.5	4	<=0.5	4	<=0.03
IV	2004-C04-021	Urine	46	16	>16	<=0.5	16	<=0.5	4	<=0.03
IV	2004-C04-028	Urine	78	>32	>16	>16	>16	>16	>16	>2
IV	2004-C04-030	Urine	67	<=2	>16	<=0.5	16	<=0.5	4	<=0.03
IV	2004-C04-032	Urine	57	4	>16	2	>16	4	>16	<=0.03
IV	2004-C04-033	Urine	30	<=2	>16	<=0.5	8	<=0.5	4	0.25
IV	2004-C04-037	Blood	89	>32	>16	>16	>16	>16	>16	>2
IV	2004-C04-042	Urine	32	4	>16	<=0.5	8	<=0.5	4	0.25
IV	2004-C04-043	Urine	57	4	8	<=0.5	4	<=0.5	<=2	<=0.03
IV	2004-C04-203	Blood	75	<=2	>16	<=0.5	16	<=0.5	<=2	0.25
IV	2004-C04-204	Blood	87	<=2	>16	<=0.5	16	<=0.5	>16	<=0.03
IV	2004-S05-001	Urine	70	<=2	>16	<=0.5	4	<=0.5	<=2	1
IV	2004-S05-005	Other	22	4	>16	<=0.5	8	<=0.5	4	<=0.03
IV	2004-S05-009	Other	79	<=2	>16	2	8	1	>16	>2
IV	2004-S05-010	Urine	59	<=2	>16	<=0.5	8	<=0.5	8	>2
IV	2004-S05-015	Urine	37	<=2	>16	<=0.5	8	<=0.5	<=2	0.12
IV	2004-S05-020	Other	53	4	4	<=0.5	4	<=0.5	<=2	<=0.03
IV	2004-S05-024	Urine	67	<=2	4	<=0.5	4	<=0.5	<=2	<=0.03
IV	2004-S05-025	Urine	9	4	>16	<=0.5	8	<=0.5	4	1
IV	2004-S05-033	Urine	37	<=2	>16	<=0.5	8	<=0.5	<=2	0.12
IV	2004-S05-036	Urine	62	<=2	>16	<=0.5	8	<=0.5	8	>2
IV	2004-S05-037	Urine	72	8	4	<=0.5	4	<=0.5	<=2	<=0.03

IV	2004-S05-038	Urine	70	<=2	>16	16	>16	16	>16	>2
IV	2004-S05-039	Urine	7	8	>16	<=0.5	16	<=0.5	8	<=0.03
IV	2004-S05-040	Urine	68	4	>16	<=0.5	16	<=0.5	4	<=0.03
IV	2004-S05-042	Urine	45	<=2	>16	<=0.5	8	<=0.5	<=2	<=0.03
IV	2004-S05-044	Urine	78	<=2	>16	16	>16	>16	>16	>2
IV	2004-S05-045	Urine	75	4	>16	<=0.5	8	<=0.5	8	0.5
IV	2004-S05-047	Urine	2	4	4	<=0.5	8	<=0.5	<=2	<=0.03
IV	2004-S05-048	Urine	78	<=2	>16	<=0.5	16	<=0.5	16	>2
IV	2004-S05-181	Urine	7	4	2	<=0.5	4	<=0.5	4	>2
IV	2004-S06-003	Urine	69	4	>16	<=0.5	8	<=0.5	16	>2
IV	2004-S06-004	Urine	57	4	4	<=0.5	4	<=0.5	<=2	<=0.03
IV	2004-S06-005	Urine	73	<=2	2	<=0.5	4	<=0.5	<=2	<=0.03
IV	2004-S06-008	Other	0	<=2	4	<=0.5	4	<=0.5	<=2	<=0.03
IV	2004-S06-009	Urine	79	4	>16	<=0.5	8	<=0.5	4	<=0.03
IV	2004-S06-011	Urine	24	<=2	>16	<=0.5	8	<=0.5	4	0.5
IV	2004-S06-012	Urine	51	<=2	>16	<=0.5	8	<=0.5	4	<=0.03
IV	2004-S06-014	Urine	58	4	2	<=0.5	4	<=0.5	<=2	<=0.03
IV	2004-S06-015	Other	28	<=2	>16	<=0.5	8	<=0.5	4	<=0.03
IV	2004-S06-016	Urine	83	4	4	<=0.5	8	<=0.5	<=2	1
IV	2004-S06-018	Other	55	16	>16	>16	16	16	>16	>2
IV	2004-S06-022	Urine	32	<=2	>16	<=0.5	8	<=0.5	4	<=0.03
IV	2004-S06-025	Urine	12	<=2	>16	<=0.5	8	<=0.5	<=2	0.12
IV	2004-S06-027	Urine	68	<=2	>16	8	8	1	>16	>2
IV	2004-S06-028	Urine	37	<=2	<=1	<=0.5	1	<=0.5	<=2	<=0.03
IV	2004-S06-029	Urine	39	<=2	4	<=0.5	4	<=0.5	<=2	<=0.03
IV	2004-S06-030	Other	30	<=2	>16	<=0.5	8	<=0.5	4	<=0.03
IV	2004-S06-036	Urine	41	4	>16	<=0.5	8	<=0.5	4	<=0.03
IV	2004-S06-038	Urine	44	4	4	<=0.5	4	<=0.5	<=2	0.25
IV	2004-S06-039	Urine	29	4	4	<=0.5	4	<=0.5	<=2	<=0.03
IV	2004-S06-040	Urine	80	<=2	>16	<=0.5	16	<=0.5	8	>2
IV	2004-S06-041	Urine	92	<=2	>16	<=0.5	8	<=0.5	4	0.25
IV	2004-S06-042	Other	27	4	>16	<=0.5	8	<=0.5	<=2	0.12
IV	2004-S06-047	Urine	67	<=2	4	<=0.5	8	<=0.5	4	0.12
IV	2004-S06-049	Urine	67	4	>16	<=0.5	8	<=0.5	8	>2
IV	2004-S06-201	Blood	68	8	>16	2	>16	16	>16	0.25
IV	2004-S06-213	Blood	61	<=2	2	<=0.5	2	<=0.5	<=2	0.25
IV	2004-S06-215	Blood	57	4	>16	<=0.5	8	<=0.5	4	<=0.03
IV	2004-S07-010	Urine	5	4	>16	<=0.5	8	<=0.5	<=2	0.5
IV	2004-S07-011	Urine	73	<=2	>16	<=0.5	8	<=0.5	<=2	0.25
IV	2004-S07-012	Other	29	4	4	<=0.5	8	<=0.5	4	<=0.03
IV	2004-S07-013	Urine	32	16	>16	<=0.5	8	<=0.5	<=2	<=0.03
IV	2004-S07-015	Urine	83	<=2	>16	<=0.5	8	<=0.5	<=2	0.25
IV	2004-S07-018	Urine	48	4	>16	<=0.5	8	<=0.5	<=2	0.25
IV	2004-S07-019	Urine	79	<=2	8	<=0.5	8	<=0.5	16	0.5
IV	2004-S07-022	Urine	54	4	>16	<=0.5	>16	<=0.5	>16	<=0.03
IV	2004-S07-026	Urine	77	<=2	>16	<=0.5	16	<=0.5	16	0.12
IV	2004-S07-027	Urine	67	<=2	>16	<=0.5	8	<=0.5	4	>2
IV	2004-S07-028	Urine	48	<=2	2	<=0.5	1	<=0.5	<=2	<=0.03
IV	2004-S07-031	Urine	70	4	>16	<=0.5	8	<=0.5	<=2	0.25
IV	2004-S07-034	Urine	32	<=2	>16	<=0.5	8	<=0.5	16	<=0.03
IV	2004-S07-037	Urine	44	<=2	4	<=0.5	8	<=0.5	<=2	<=0.03
IV	2004-S07-038	Urine	52	<=2	>16	<=0.5	8	<=0.5	<=2	<=0.03
IV	2004-S07-039	Urine	29	<=2	>16	<=0.5	8	<=0.5	4	0.5
IV	2004-S07-040	Urine	20	<=2	>16	<=0.5	4	<=0.5	<=2	<=0.03
IV	2004-S07-041	Urine	66	<=2	>16	<=0.5	16	<=0.5	16	<=0.03
IV	2004-S07-042	Urine	24	4	4	<=0.5	8	<=0.5	4	<=0.03
IV	2004-S07-043	Urine	71	<=2	2	<=0.5	4	<=0.5	<=2	0.25
IV	2004-S07-048	Urine	21	4	2	<=0.5	4	<=0.5	<=2	<=0.03
IV	2004-S07-202	Blood	75	<=2	<=1	<=0.5	1	<=0.5	<=2	<=0.03
IV	2004-S07-203	Blood	64	<=2	>16	<=0.5	8	<=0.5	4	0.25
IV	2004-S07-205	Blood	78	<=2	>16	<=0.5	8	<=0.5	<=2	0.25

IV	2004-S07-206	Blood	35	<=2	>16	<=0.5	8	<=0.5	4	<=0.03
IV	2004-S07-213	Blood	53	4	>16	<=0.5	8	<=0.5	4	0.25
IV	2004-S07-216	Blood	49	<=2	>16	<=0.5	8	<=0.5	<=2	<=0.03
IV	2004-N05-003	Urine	1	<=2	>16	<=0.5	16	<=0.5	16	<=0.03
IV	2004-N05-006	Urine	75	4	>16	<=0.5	16	<=0.5	>16	0.25
IV	2004-N05-009	Urine		4	>16	<=0.5	16	<=0.5	>16	0.12
IV	2004-N05-010	Urine	71	4	16	<=0.5	8	<=0.5	8	<=0.03
IV	2004-N05-011	Urine	75	<=2	>16	<=0.5	>16	<=0.5	>16	0.12
IV	2004-N05-015	Urine	29	8	4	<=0.5	4	<=0.5	<=2	<=0.03
IV	2004-N05-016	Urine	64	<=2	>16	<=0.5	8	<=0.5	<=2	<=0.03
IV	2004-N05-018	Urine	64	<=2	>16	<=0.5	8	<=0.5	4	<=0.03
IV	2004-N05-023	Urine	57	4	>16	1	8	<=0.5	<=2	<=0.03
IV	2004-N05-024	Urine	50	4	>16	<=0.5	8	<=0.5	4	0.25
IV	2004-N05-028	Urine		4	>16	<=0.5	8	<=0.5	<=2	0.25
IV	2004-N05-029	Urine	63	<=2	>16	<=0.5	8	<=0.5	8	>2
IV	2004-N05-030	Other	79	4	>16	16	>16	>16	>16	0.5
IV	2004-N05-032	Urine	3	<=2	>16	<=0.5	8	<=0.5	4	<=0.03
IV	2004-N05-033	Urine	26	<=2	4	<=0.5	4	<=0.5	<=2	<=0.03
IV	2004-N05-035	Urine		4	>16	<=0.5	8	<=0.5	4	>2
IV	2004-N05-038	Urine		4	>16	<=0.5	8	<=0.5	4	0.25
IV	2004-N05-049	Urine	39	<=2	2	<=0.5	4	<=0.5	<=2	<=0.03
IV	2004-N05-206	Blood	72	<=2	4	<=0.5	4	<=0.5	<=2	<=0.03
IV	2004-N05-218	Blood	82	<=2	>16	4	16	1	>16	>2
IV	2004-N06-006	Other	26	<=2	>16	<=0.5	8	<=0.5	<=2	<=0.03
IV	2004-N06-013	Blood	66	<=2	>16	<=0.5	8	<=0.5	4	0.25
IV	2004-N06-014	Blood	67	<=2	>16	<=0.5	8	<=0.5	<=2	0.25
IV	2004-N06-016	Other	46	<=2	>16	<=0.5	8	<=0.5	<=2	<=0.03
IV	2004-N06-017	Urine	45	<=2	>16	16	>16	>16	>16	>2
IV	2004-N06-018	Urine	88	4	>16	<=0.5	4	<=0.5	<=2	<=0.03
IV	2004-N06-025	Urine	5	<=2	>16	<=0.5	8	<=0.5	<=2	0.5
IV	2004-N06-026	Urine	61	<=2	>16	<=0.5	8	<=0.5	<=2	0.5
IV	2004-N06-030	Urine	69	<=2	>16	<=0.5	8	<=0.5	4	<=0.03
IV	2004-N06-044	Urine	62	4	4	<=0.5	4	<=0.5	4	>2
IV	2004-N06-045	Urine	79	<=2	>16	<=0.5	8	<=0.5	4	<=0.03
IV	2004-N06-046	Urine	6	<=2	4	<=0.5	4	<=0.5	<=2	<=0.03
IV	2004-N06-207	Blood	67	<=2	>16	<=0.5	8	<=0.5	<=2	<=0.03
IV	2004-N06-213	Blood	54	<=2	4	<=0.5	4	<=0.5	<=2	<=0.03
IV	2004-N06-214	Blood	70	<=2	4	<=0.5	4	<=0.5	<=2	<=0.03
IV	2004-N06-215	Blood	57	<=2	>16	<=0.5	8	2	4	0.12
IV	2004-N06-220	Blood	74	<=2	4	<=0.5	8	<=0.5	<=2	<=0.03
IV	2004-N07-001	Urine	69	<=2	>16	<=0.5	8	<=0.5	8	<=0.03
IV	2004-N07-011	Other	42	4	>16	<=0.5	16	<=0.5	>16	>2
IV	2004-N07-014	Urine	36	4	>16	<=0.5	8	<=0.5	<=2	0.25
IV	2004-N07-022	Urine	57	<=2	>16	<=0.5	8	1	4	<=0.03
IV	2004-N07-023	Urine	76	4	4	<=0.5	8	<=0.5	<=2	<=0.03
IV	2004-N07-028	Blood	47	<=2	>16	<=0.5	8	<=0.5	<=2	0.5
IV	2004-N07-031	Urine	52	8	>16	<=0.5	8	<=0.5	<=2	>2
IV	2004-N07-033	Urine	78	4	>16	8	>16	8	>16	>2
IV	2004-N07-034	Urine	63	<=2	>16	<=0.5	16	<=0.5	8	<=0.03
IV	2004-N07-038	Blood	70	<=2	>16	<=0.5	8	<=0.5	4	0.25
IV	2004-N07-046	Urine	63	8	4	<=0.5	4	<=0.5	<=2	1
IV	2004-N07-047	Other	25	<=2	2	<=0.5	4	<=0.5	<=2	<=0.03
IV	2004-N07-195	Urine	5	4	>16	<=0.5	8	<=0.5	8	1
IV	2004-N07-201	Blood	76	4	4	<=0.5	4	<=0.5	<=2	0.5
IV	2004-N07-210	Blood	53	8	8	<=0.5	8	<=0.5	8	>2
IV	2004-N07-211	Blood	88	4	>16	<=0.5	8	<=0.5	8	<=0.03
IV	2004-S08-004	Urine	67	4	8	<=0.5	8	<=0.5	4	<=0.03
IV	2004-S08-011	Urine	49	4	>16	<=0.5	8	<=0.5	<=2	>2
IV	2004-S08-013	Urine	65	4	>16	<=0.5	8	<=0.5	4	<=0.03
IV	2004-S08-025	Urine	48	4	>16	<=0.5	8	<=0.5	4	<=0.03
IV	2004-S08-028	Urine	81	4	>16	<=0.5	8	<=0.5	16	>2

IV	2004-E02-001	Urine	80	4	>16	<=0.5	8	<=0.5	16	<=0.03
IV	2004-E02-005	Urine	33	<=2	>16	<=0.5	8	<=0.5	8	>2
IV	2004-E02-007	Urine	71	<=2	>16	<=0.5	8	<=0.5	<=2	0.25
IV	2004-E02-011	Urine	27	<=2	>16	<=0.5	8	<=0.5	<=2	<=0.03
IV	2004-E02-016	Urine	61	4	2	<=0.5	4	<=0.5	<=2	0.25
IV	2004-E02-017	Urine	0	8	>16	<=0.5	16	<=0.5	8	0.25
IV	2004-E02-020	Urine	1	4	>16	<=0.5	8	<=0.5	<=2	<=0.03
IV	2004-E02-022	Urine	69	<=2	4	<=0.5	4	<=0.5	<=2	<=0.03
IV	2004-E02-024	Urine	0	<=2	>16	<=0.5	8	<=0.5	8	0.25
IV	2004-E02-026	Other	39	<=2	2	<=0.5	4	<=0.5	<=2	<=0.03
IV	2004-E02-030	Urine	20	<=2	>16	<=0.5	16	<=0.5	4	<=0.03
IV	2004-E02-034	Urine	68	<=2	>16	<=0.5	8	<=0.5	8	<=0.03
IV	2004-E02-038	Urine	44	<=2	>16	<=0.5	8	<=0.5	<=2	0.5
IV	2004-E02-040	Urine	0	4	2	<=0.5	4	<=0.5	<=2	<=0.03
IV	2004-E02-041	Urine	76	<=2	>16	<=0.5	4	<=0.5	<=2	0.12
IV	2004-E02-042	Urine	88	<=2	>16	<=0.5	8	<=0.5	4	<=0.03
IV	2004-E02-043	Urine	24	4	4	<=0.5	8	<=0.5	<=2	<=0.03
IV	2004-E02-044	Urine	24	8	>16	<=0.5	8	<=0.5	4	0.25
IV	2004-E02-048	Urine	42	<=2	2	<=0.5	8	<=0.5	<=2	<=0.03
IV	2004-E02-049	Urine	24	<=2	>16	<=0.5	8	<=0.5	4	0.25
IV	2004-N08-001	Blood	75	16	>16	>16	>16	>16	>16	>2
IV	2004-N08-005	Urine	26	<=2	>16	<=0.5	8	<=0.5	4	<=0.03
IV	2004-N08-009	Urine	37	<=2	>16	<=0.5	8	<=0.5	16	<=0.03
IV	2004-N08-010	Urine	23	<=2	4	<=0.5	4	<=0.5	<=2	<=0.03
IV	2004-N08-012	Urine	31	<=2	4	<=0.5	2	<=0.5	<=2	<=0.03
IV	2004-N08-021	Urine	3	<=2	>16	<=0.5	8	<=0.5	<=2	1
IV	2004-N08-022	Urine	29	<=2	2	<=0.5	4	<=0.5	<=2	<=0.03
IV	2004-N08-023	Blood	87	<=2	>16	8	4	16	>16	>2
IV	2004-N08-028	Urine	29	4	4	<=0.5	2	<=0.5	<=2	<=0.03
IV	2004-N08-038	Blood	78	<=2	>16	<=0.5	8	<=0.5	<=2	<=0.03
IV	2004-N08-042	Blood	31	<=2	>16	<=0.5	16	<=0.5	>16	<=0.03
IV	2004-N08-044	Urine	41	<=2	2	<=0.5	4	<=0.5	<=2	<=0.03
IV	2004-N08-045	Urine	89	4	>16	<=0.5	8	<=0.5	>16	>2
IV	2004-N08-216	Blood	57	4	>16	<=0.5	8	<=0.5	8	0.25
IV	2004-S09-009	Urine	91	16	>16	<=0.5	4	<=0.5	16	>2
IV	2004-S09-010	Urine	39	4	>16	<=0.5	16	<=0.5	4	<=0.03
IV	2004-S09-011	Urine	23	<=2	<=1	<=0.5	1	<=0.5	<=2	<=0.03
IV	2004-S09-012	Urine	61	<=2	<=1	<=0.5	1	<=0.5	<=2	<=0.03
IV	2004-S09-016	Urine	43	<=2	>16	<=0.5	8	<=0.5	<=2	<=0.03
IV	2004-S09-017	Urine	91	<=2	>16	<=0.5	8	<=0.5	16	>2
IV	2004-S09-019	Blood	74	<=2	>16	<=0.5	8	<=0.5	<=2	<=0.03
IV	2004-S09-022	Blood	69	4	>16	4	>16	16	>16	<=0.03
IV	2004-S09-023	Blood	51	<=2	>16	<=0.5	>16	1	>16	0.25
IV	2004-S09-025	Urine	79	<=2	>16	8	>16	16	>16	<=0.03
IV	2004-S09-035	Other	35	<=2	4	<=0.5	8	<=0.5	<=2	0.25
IV	2004-S09-037	Urine	75	<=2	>16	<=0.5	8	<=0.5	<=2	0.25
IV	2004-S09-038	Urine	39	4	4	<=0.5	4	<=0.5	<=2	<=0.03
IV	2004-S09-039	Urine	91	<=2	2	<=0.5	2	<=0.5	<=2	0.12
IV	2004-S09-045	Blood	83	4	2	<=0.5	4	<=0.5	<=2	<=0.03
IV	2004-S09-047	Blood	78	<=2	>16	<=0.5	8	<=0.5	4	>2
IV	2004-S09-048	Blood	56	<=2	>16	<=0.5	8	<=0.5	<=2	<=0.03
IV	2004-S09-183	Blood	0	<=2	>16	<=0.5	8	<=0.5	4	<=0.03
IV	2004-S09-202	Blood	0	<=2	<=1	<=0.5	1	<=0.5	<=2	<=0.03
IV	2004-S09-204	Blood	74	4	>16	<=0.5	16	<=0.5	16	<=0.03
IV	2004-E03-001	Urine	41	<=2	2	<=0.5	4	<=0.5	<=2	0.25
IV	2004-E03-004	Urine	51	<=2	4	<=0.5	4	<=0.5	<=2	<=0.03
IV	2004-E03-013	Urine	91	<=2	16	<=0.5	2	<=0.5	<=2	<=0.03
IV	2004-E03-020	Other	34	<=2	>16	<=0.5	8	<=0.5	4	>2
IV	2004-E03-026	Urine	78	<=2	>16	<=0.5	8	<=0.5	<=2	0.25
IV	2004-E03-030	Urine	67	<=2	4	<=0.5	8	<=0.5	<=2	<=0.03
IV	2004-E03-031	Urine	53	<=2	>16	>16	16	4	>16	>2

IV	2004-E03-044	Urine	64	<=2	2	<=0.5	4	<=0.5	<=2	<=0.03
IV	2004-E03-046	Urine	68	<=2	>16	16	>16	16	>16	0.25
IV	2004-E03-050	Urine	26	<=2	>16	8	>16	16	>16	>2
IV	2004-E03-203	Blood	62	4	>16	<=0.5	8	<=0.5	4	<=0.03
IV	2004-E03-204	Blood	59	4	8	<=0.5	8	<=0.5	<=2	<=0.03
IV	2004-C06-003	Urine	57	<=2	>16	<=0.5	8	<=0.5	<=2	>2
IV	2004-C06-007	Urine	39	8	>16	<=0.5	8	<=0.5	8	0.25
IV	2004-C06-008	Blood	73	<=2	>16	4	>16	16	>16	1
IV	2004-C06-016	Blood	70	<=2	>16	8	16	2	>16	>2
IV	2004-C06-018	Urine	16	<=2	>16	<=0.5	16	<=0.5	8	0.25
IV	2004-C06-019	Urine	84	<=2	>16	>16	16	4	>16	>2
IV	2004-C06-021	Urine	83	<=2	2	<=0.5	4	<=0.5	<=2	<=0.03
IV	2004-C06-024	Urine	80	<=2	>16	<=0.5	4	<=0.5	<=2	0.12
IV	2004-C06-026	Urine	59	<=2	>16	<=0.5	16	<=0.5	16	<=0.03
IV	2004-C06-030	Other	79	4	8	<=0.5	8	<=0.5	<=2	0.5
IV	2004-C06-032	Urine	8	<=2	>16	<=0.5	8	<=0.5	<=2	<=0.03
IV	2004-C06-034	Urine	82	<=2	>16	<=0.5	8	<=0.5	4	0.25
IV	2004-C06-037	Blood	70	<=2	>16	<=0.5	8	<=0.5	4	<=0.03
IV	2004-C06-038	Urine	80	<=2	>16	<=0.5	8	<=0.5	4	0.25
IV	2004-C06-045	Urine	76	4	>16	>16	>16	>16	>16	>2
IV	2004-C07-002	Urine	75	<=2	2	<=0.5	4	<=0.5	<=2	0.12
IV	2004-C07-005	Urine	4	4	>16	<=0.5	8	<=0.5	4	0.5
IV	2004-C07-006	Urine	35	<=2	>16	<=0.5	8	<=0.5	8	0.5
IV	2004-C07-010	Urine	50	<=2	>16	<=0.5	8	<=0.5	4	0.5
IV	2004-C07-011	Urine	92	4	4	<=0.5	4	<=0.5	<=2	0.5
IV	2004-C07-013	Urine	28	<=2	>16	<=0.5	8	<=0.5	<=2	<=0.03
IV	2004-C07-014	Urine	84	8	>16	<=0.5	8	<=0.5	<=2	0.12
IV	2004-N16-003	Other	19	<=2	4	<=0.5	4	<=0.5	<=2	<=0.03
IV	2004-N16-011	Urine	78	<=2	>16	<=0.5	8	<=0.5	4	<=0.03
IV	2004-N16-015	Urine	76	<=2	>16	<=0.5	16	<=0.5	16	>2
IV	2004-N16-017	Other	80	<=2	>16	<=0.5	>16	<=0.5	<=2	<=0.03
IV	2004-N16-021	Urine	77	<=2	4	1	4	<=0.5	<=2	<=0.03
IV	2004-N16-035	Urine	83	<=2	>16	<=0.5	4	<=0.5	4	0.25
IV	2004-N16-038	Urine	18	<=2	8	<=0.5	4	<=0.5	<=2	<=0.03
IV	2004-N16-042	Urine	79	<=2	>16	<=0.5	8	<=0.5	<=2	0.25
IV	2004-N16-049	Other	78	>32	>16	>16	>16	>16	>16	>2
IV	2004-N16-050	Other	33	<=2	>16	<=0.5	8	<=0.5	4	<=0.03
IV	2004-N16-201	Blood	53	<=2	4	<=0.5	4	<=0.5	<=2	<=0.03
IV	2004-N16-210	Blood	82	<=2	>16	<=0.5	16	<=0.5	8	0.25
IV	2004-N16-212	Blood	78	<=2	2	<=0.5	4	<=0.5	<=2	<=0.03
IV	2004-N16-213	Blood	71	<=2	>16	<=0.5	8	<=0.5	<=2	1
IV	2004-N16-214	Blood	56	<=2	>16	<=0.5	8	<=0.5	8	2
IV	2004-N16-216	Blood	49	<=2	2	<=0.5	4	<=0.5	<=2	0.12
IV	2004-N17-004	Urine	26	<=2	<=1	<=0.5	2	<=0.5	<=2	<=0.03
IV	2004-N17-005	Urine	78	<=2	4	<=0.5	4	<=0.5	<=2	<=0.03
IV	2004-N17-006	Urine	33	<=2	>16	<=0.5	8	<=0.5	<=2	0.12
IV	2004-N17-010	Urine	70	4	>16	<=0.5	8	<=0.5	<=2	<=0.03
IV	2004-N17-011	Urine	27	4	4	<=0.5	4	<=0.5	4	<=0.03
IV	2004-N17-012	Urine	76	4	>16	<=0.5	8	<=0.5	4	0.25
IV	2004-N17-017	Other	82	<=2	>16	<=0.5	8	<=0.5	8	0.25
IV	2004-N17-024	Urine	26	<=2	>16	<=0.5	8	<=0.5	<=2	<=0.03
IV	2004-N17-025	Urine	90	<=2	8	<=0.5	16	<=0.5	<=2	<=0.03
IV	2004-N17-028	Urine	20	<=2	>16	<=0.5	8	<=0.5	<=2	>2
IV	2004-N17-029	Urine	57	4	2	<=0.5	2	<=0.5	<=2	<=0.03
IV	2004-N17-030	Urine	19	4	>16	<=0.5	8	<=0.5	4	0.12
IV	2004-N17-033	Urine	64	4	>16	<=0.5	8	<=0.5	<=2	0.25
IV	2004-N17-036	Urine	26	4	4	<=0.5	4	<=0.5	<=2	<=0.03
IV	2004-N17-039	Urine	24	<=2	>16	<=0.5	8	<=0.5	4	<=0.03
IV	2004-N17-042	Urine	78	4	>16	<=0.5	8	<=0.5	<=2	<=0.03
IV	2004-N17-150	Urine	67	<=2	>16	<=0.5	16	<=0.5	>16	>2
IV	2004-N17-205	Blood	76	4	8	<=0.5	4	<=0.5	4	<=0.03

IV	2004-N19-002	Urine	62	4	>16	<=0.5	8	<=0.5	4	<=0.03
IV	2004-N19-003	Urine	82	4	2	<=0.5	4	<=0.5	16	<=0.03
IV	2004-N19-004	Urine	77	16	<=1	<=0.5	1	<=0.5	4	0.12
IV	2004-N19-006	Blood	78	8	>16	<=0.5	8	<=0.5	4	<=0.03
IV	2004-N19-012	Blood	83	4	>16	<=0.5	8	<=0.5	8	0.12
IV	2004-N19-013	Blood	70	<=2	>16	<=0.5	8	<=0.5	<=2	<=0.03
IV	2004-N19-014	Urine	78	<=2	>16	<=0.5	8	<=0.5	<=2	0.25
IV	2004-N19-018	Urine	73	<=2	>16	<=0.5	8	<=0.5	4	<=0.03
IV	2004-N19-021	Urine	50	<=2	>16	<=0.5	8	<=0.5	<=2	0.5
IV	2004-N19-022	Urine	73	4	>16	<=0.5	8	<=0.5	8	>2
IV	2004-N19-024	Urine	83	<=2	4	<=0.5	4	<=0.5	<=2	<=0.03
IV	2004-N19-025	Urine	26	8	>16	<=0.5	8	<=0.5	4	<=0.03
IV	2004-N19-027	Urine	65	<=2	>16	<=0.5	8	<=0.5	8	0.12
IV	2004-N19-031	Urine	20	<=2	>16	<=0.5	8	<=0.5	<=2	<=0.03
IV	2004-N19-032	Urine	36	<=2	4	<=0.5	4	<=0.5	<=2	<=0.03
IV	2004-N19-036	Urine	91	4	>16	<=0.5	4	<=0.5	4	0.12
IV	2004-N19-037	Urine	84	4	16	<=0.5	4	<=0.5	8	2
IV	2004-N19-040	Urine	52	<=2	>16	<=0.5	8	<=0.5	<=2	0.25
IV	2004-N19-041	Urine	80	<=2	>16	<=0.5	8	<=0.5	8	>2
IV	2004-N19-045	Urine	27	8	>16	<=0.5	8	<=0.5	<=2	0.25
IV	2004-N19-049	Urine	26	8	>16	<=0.5	8	1	8	1
IV	2004-N19-205	Blood	81	>32	>16	>16	16	2	>16	>2
IV	2004-N19-216	Blood	72	<=2	>16	<=0.5	8	<=0.5	<=2	<=0.03
IV	2004-N19-217	Blood	70	4	>16	<=0.5	8	<=0.5	4	>2
IV	2004-N19-218	Blood	68	<=2	>16	<=0.5	16	<=0.5	8	0.25
IV	2004-C08-002	Other	52	<=2	>16	8	>16	16	>16	1
IV	2004-C08-007	Urine	56	<=2	<=1	<=0.5	1	<=0.5	<=2	<=0.03
IV	2004-C08-012	Urine	77	<=2	>16	<=0.5	8	<=0.5	<=2	0.25
IV	2004-C08-014	Urine	76	4	>16	<=0.5	8	<=0.5	4	<=0.03
IV	2004-C08-017	Other	63	<=2	<=1	<=0.5	2	<=0.5	<=2	<=0.03
IV	2004-C08-022	Urine	91	8	>16	<=0.5	8	<=0.5	8	<=0.03
IV	2004-C08-031	Urine	49	>32	>16	16	>16	4	>16	>2
IV	2004-C08-041	Other	30	4	4	<=0.5	4	<=0.5	<=2	<=0.03
IV	2004-C08-044	Other	17	<=2	>16	<=0.5	8	<=0.5	<=2	<=0.03
IV	2004-C08-202	Blood	77	<=2	>16	<=0.5	16	<=0.5	8	>2
IV	2004-C08-205	Blood	47	<=2	>16	<=0.5	16	<=0.5	4	<=0.03
IV	2004-S11-003	Urine	69	<=2	>16	<=0.5	8	<=0.5	<=2	<=0.03
IV	2004-S11-013	Urine	71	<=2	>16	<=0.5	8	<=0.5	4	0.25
IV	2004-S11-014	Urine	45	4	4	<=0.5	4	<=0.5	<=2	<=0.03
IV	2004-S11-015	Urine	78	<=2	>16	<=0.5	8	<=0.5	4	0.25
IV	2004-S11-016	Urine	67	4	>16	<=0.5	8	<=0.5	4	0.06
IV	2004-S11-025	Urine	85	4	4	<=0.5	4	<=0.5	<=2	<=0.03
IV	2004-S11-030	Urine	87	<=2	2	<=0.5	4	<=0.5	<=2	0.12
IV	2004-S11-031	Urine	78	4	4	<=0.5	4	<=0.5	<=2	<=0.03
IV	2004-S11-036	Urine	64	4	>16	<=0.5	8	<=0.5	4	<=0.03
IV	2004-S11-039	Urine	75	<=2	4	<=0.5	8	<=0.5	<=2	<=0.03
IV	2004-S11-041	Urine	65	<=2	>16	<=0.5	16	<=0.5	16	<=0.03
IV	2004-S11-042	Urine	26	<=2	>16	<=0.5	8	<=0.5	<=2	>2
IV	2004-S11-044	Urine	59	4	>16	<=0.5	8	<=0.5	4	<=0.03
IV	2004-S11-045	Urine	27	<=2	>16	<=0.5	8	<=0.5	4	0.12
IV	2004-S11-046	Urine	32	<=2	>16	<=0.5	16	<=0.5	16	<=0.03
IV	2004-S11-047	Urine	73	<=2	4	<=0.5	4	<=0.5	4	0.25
IV	2004-S11-193	Urine	4	4	>16	<=0.5	16	<=0.5	>16	0.25
IV	2004-S11-203	Blood	37	<=2	>16	<=0.5	8	<=0.5	4	0.25
IV	2004-S11-206	Blood	79	<=2	>16	<=0.5	8	<=0.5	4	0.25
IV	2004-S11-207	Blood	77	4	2	<=0.5	4	<=0.5	<=2	<=0.03
IV	2004-N21-017	Urine	50	<=2	>16	8	>16	16	>16	0.25
IV	2004-N21-022	Urine	1	<=2	>16	<=0.5	16	<=0.5	>16	<=0.03
IV	2004-N21-029	Urine	90	<=2	2	<=0.5	4	<=0.5	<=2	<=0.03
IV	2004-N21-033	Urine	68	4	>16	<=0.5	8	<=0.5	<=2	<=0.03
IV	2004-N21-037	Urine	46	<=2	2	<=0.5	2	<=0.5	<=2	<=0.03

IV	2004-N21-042	Urine	4	<=2	>16	<=0.5	8	<=0.5	4	1
IV	2004-N21-050	Urine	25	<=2	2	<=0.5	2	<=0.5	<=2	<=0.03
IV	2004-N21-196	Urine	1	<=2	>16	<=0.5	8	<=0.5	8	<=0.03
IV	2004-N21-197	Urine	0	4	>16	<=0.5	8	<=0.5	4	<=0.03
IV	2004-N21-206	Blood	68	4	>16	<=0.5	8	<=0.5	4	<=0.03
IV	2004-N21-209	Blood	87	4	>16	<=0.5	8	<=0.5	8	<=0.03
IV	2004-C09-002	Urine	82	<=2	>16	<=0.5	8	<=0.5	4	>2
IV	2004-C09-004	Urine	80	<=2	>16	<=0.5	8	<=0.5	4	0.25
IV	2004-C09-006	Urine	5	<=2	>16	16	>16	>16	>16	>2
IV	2004-C09-020	Urine	71	8	>16	<=0.5	16	<=0.5	8	0.5
IV	2004-C09-022	Urine	36	<=2	2	<=0.5	4	<=0.5	<=2	<=0.03
IV	2004-C09-023	Urine	67	<=2	4	<=0.5	4	<=0.5	4	<=0.03
IV	2004-C09-033	Other	65	<=2	>16	<=0.5	8	<=0.5	4	>2
IV	2004-C09-037	Urine	5	<=2	>16	<=0.5	8	<=0.5	4	>2
IV	2004-C09-040	Urine	68	<=2	>16	2	>16	4	>16	>2
IV	2004-C09-041	Urine	37	<=2	>16	<=0.5	>16	<=0.5	8	>2
IV	2004-C09-045	Other	57	<=2	>16	<=0.5	8	<=0.5	8	>2
IV	2004-C09-047	Urine	25	<=2	>16	<=0.5	8	<=0.5	4	<=0.03
IV	2004-C09-048	Urine	79	>32	>16	16	>16	>16	>16	<=0.03
IV	2004-C09-049	Urine	1	<=2	>16	<=0.5	16	<=0.5	<=2	<=0.03
IV	2004-C09-206	Blood	73	4	>16	<=0.5	8	<=0.5	<=2	<=0.03
IV	2004-C09-210	Blood	41	<=2	>16	<=0.5	8	<=0.5	<=2	<=0.03
IV	2004-C10-003	Urine	87	4	>16	<=0.5	8	<=0.5	4	<=0.03
IV	2004-C10-004	Urine	70	<=2	>16	<=0.5	8	<=0.5	<=2	0.5
IV	2004-C10-008	Blood	57	<=2	>16	<=0.5	8	<=0.5	4	<=0.03
IV	2004-C10-012	Urine	70	<=2	>16	<=0.5	8	<=0.5	<=2	0.5
IV	2004-C10-013	Urine	58	4	4	<=0.5	4	<=0.5	<=2	<=0.03
IV	2004-C10-017	Urine	78	<=2	>16	16	>16	>16	>16	>2
IV	2004-C10-018	Urine	21	4	<=1	<=0.5	2	<=0.5	<=2	<=0.03
IV	2004-C10-019	Urine	78	<=2	>16	<=0.5	8	<=0.5	4	<=0.03
IV	2004-C10-020	Urine	80	8	>16	>16	>16	>16	>16	>2
IV	2004-C10-024	Blood	61	<=2	>16	8	>16	16	>16	0.5
IV	2004-C10-025	Urine	77	<=2	>16	<=0.5	16	<=0.5	16	<=0.03
IV	2004-C10-031	Urine	67	<=2	4	<=0.5	8	<=0.5	8	0.12
IV	2004-C10-032	Urine	65	4	>16	<=0.5	8	<=0.5	8	<=0.03
IV	2004-C10-036	Urine	45	4	>16	<=0.5	16	<=0.5	16	<=0.03
IV	2004-C10-037	Urine	49	4	>16	<=0.5	16	<=0.5	16	<=0.03
IV	2004-C10-038	Blood	35	<=2	>16	<=0.5	8	<=0.5	4	>2
IV	2004-C10-039	Urine	82	<=2	>16	<=0.5	8	<=0.5	<=2	0.25
IV	2004-C10-040	Urine	35	4	>16	<=0.5	16	<=0.5	4	<=0.03
IV	2004-C10-041	Urine	87	<=2	>16	<=0.5	16	<=0.5	4	<=0.03
IV	2004-C10-045	Urine	63	4	>16	<=0.5	8	<=0.5	8	>2
IV	2004-C10-046	Urine	63	4	>16	<=0.5	8	<=0.5	<=2	<=0.03
IV	2004-C10-047	Blood	49	4	2	<=0.5	4	<=0.5	<=2	<=0.03
IV	2004-C10-192	Urine	1	4	>16	<=0.5	8	<=0.5	4	<=0.03
IV	2004-C10-205	Blood	75	8	>16	<=0.5	8	<=0.5	4	0.25
IV	2004-C10-219	Blood	92	<=2	>16	<=0.5	8	<=0.5	<=2	<=0.03
IV	2004-E04-003	Urine	85	4	>16	<=0.5	16	<=0.5	16	0.25
IV	2004-E04-004	Blood	66	4	4	<=0.5	4	<=0.5	<=2	<=0.03
IV	2004-E04-009	Urine	34	16	4	<=0.5	4	<=0.5	<=2	<=0.03
IV	2004-E04-015	Blood	56	<=2	>16	<=0.5	8	<=0.5	4	0.25
IV	2004-E04-020	Urine	42	<=2	2	<=0.5	4	<=0.5	4	<=0.03
IV	2004-E04-027	Blood	53	<=2	4	<=0.5	4	<=0.5	4	<=0.03
IV	2004-E04-031	Urine	22	<=2	>16	<=0.5	16	<=0.5	>16	<=0.03
IV	2004-E04-032	Blood	74	4	>16	<=0.5	8	<=0.5	<=2	<=0.03
IV	2004-E04-036	Urine	70	<=2	>16	<=0.5	8	<=0.5	<=2	>2
IV	2004-E04-037	Urine	47	<=2	<=1	<=0.5	2	<=0.5	<=2	<=0.03
IV	2004-E04-039	Urine	77	<=2	4	<=0.5	4	<=0.5	<=2	<=0.03
IV	2004-E04-043	Urine	65	<=2	>16	<=0.5	8	<=0.5	<=2	<=0.03
IV	2004-E04-046	Urine	81	<=2	>16	<=0.5	8	<=0.5	4	<=0.03
IV	2004-E04-048	Urine	79	4	<=1	<=0.5	2	<=0.5	<=2	<=0.03

IV	2004-E04-055	Urine	71	8	>16	<=0.5	8	<=0.5	4	>2
IV	2004-E04-057	Other	53	8	>16	>16	>16	>16	>16	<=0.03
IV	2004-E04-068	Other	62	8	>16	<=0.5	16	<=0.5	4	0.12
IV	2004-E04-069	Urine	48	4	>16	16	8	4	>16	0.25
IV	2004-E04-073	Blood	44	8	>16	<=0.5	8	<=0.5	4	>2
IV	2004-E04-079	Other	80	8	>16	16	>16	>16	>16	>2
IV	2004-E04-081	Urine	62	4	>16	<=0.5	8	<=0.5	<=2	0.12
IV	2004-E04-084	Blood	51	<=2	4	<=0.5	4	<=0.5	<=2	0.25
IV	2004-E04-093	Other	81	<=2	>16	<=0.5	16	<=0.5	16	<=0.03
IV	2004-E04-097	Urine	61	4	>16	<=0.5	8	<=0.5	4	>2
IV	2004-E04-100	Other	77	<=2	>16	<=0.5	16	<=0.5	<=2	>2
IV	2004-E04-101	Urine	73	<=2	>16	<=0.5	16	<=0.5	8	2
IV	2004-E04-107	Urine	60	<=2	4	<=0.5	4	<=0.5	<=2	>2
IV	2004-E04-113	Urine	75	4	>16	<=0.5	8	<=0.5	4	0.25
IV	2004-E04-114	Other	37	<=2	>16	<=0.5	8	<=0.5	4	0.25
IV	2004-E04-120	Other	63	4	>16	<=0.5	16	<=0.5	8	>2
IV	2004-E04-122	Urine	61	8	4	<=0.5	4	<=0.5	8	<=0.03
IV	2004-E04-125	Urine	38	4	4	<=0.5	8	<=0.5	<=2	>2
IV	2004-E04-127	Other	69	<=2	>16	<=0.5	16	<=0.5	16	>2
IV	2004-E04-128	Blood	79	8	>16	<=0.5	8	<=0.5	4	<=0.03
IV	2004-E04-133	Blood	50	<=2	2	<=0.5	4	<=0.5	<=2	<=0.03
IV	2004-E04-135	Urine	36	4	>16	<=0.5	8	<=0.5	16	>2
IV	2004-E04-137	Urine	73	4	>16	<=0.5	>16	<=0.5	>16	<=0.03
IV	2004-E04-139	Urine	52	<=2	>16	>16	>16	>16	>16	>2
IV	2004-E04-151	Urine	73	4	4	<=0.5	8	<=0.5	<=2	<=0.03
IV	2004-E04-154	Blood	49	<=2	4	<=0.5	4	<=0.5	<=2	<=0.03
IV	2004-E04-155	Other	60	<=2	2	<=0.5	4	<=0.5	<=2	<=0.03
IV	2004-E04-161	Blood	16	<=2	>16	16	>16	>16	>16	>2
IV	2004-E04-170	Other	72	<=2	>16	4	>16	16	>16	1
IV	2004-E04-178	Urine	77	<=2	>16	<=0.5	8	<=0.5	<=2	<=0.03
IV	2004-E04-195	Urine	0	<=2	>16	<=0.5	8	<=0.5	4	1
IV	2004-E04-202	Blood	49	4	2	<=0.5	4	<=0.5	<=2	<=0.03
IV	2004-E04-207	Blood	26	<=2	4	<=0.5	4	<=0.5	<=2	<=0.03
IV	2004-E04-210	Blood	76	<=2	>16	<=0.5	8	<=0.5	4	0.12
IV	2004-E04-212	Blood	77	<=2	4	<=0.5	4	<=0.5	<=2	<=0.03
IV	2004-E04-214	Blood	77	<=2	2	<=0.5	4	<=0.5	4	<=0.03
IV	2004-E04-217	Blood	73	<=2	>16	<=0.5	16	<=0.5	4	0.5
IV	2004-E04-220	Blood	70	<=2	4	<=0.5	4	<=0.5	<=2	0.25
V	2006-S01-001	Urine	69	<=2	>16	<=0.5	4	<=0.5	<=2	1
V	2006-S01-002	Urine	22	<=2	>16	<=0.5	8	<=0.5	<=2	<=0.03
V	2006-S01-003	Urine	77	<=2	>16	<=0.5	8	<=0.5	4	<=0.03
V	2006-S01-004	Urine	64	<=2	>16	<=0.5	8	<=0.5	4	0.12
V	2006-S01-007	Urine	81	<=2	>16	<=0.5	8	<=0.5	<=2	0.12
V	2006-S01-008	Urine	80	4	>16	<=0.5	8	<=0.5	<=2	<=0.03
V	2006-S01-010	Urine	51	<=2	>16	<=0.5	8	<=0.5	4	<=0.03
V	2006-S01-014	Urine	70	4	>16	<=0.5	8	<=0.5	4	>2
V	2006-S01-019	Urine	75	<=2	>16	<=0.5	16	<=0.5	>16	<=0.03
V	2006-S01-021	Urine	49	8	>16	<=0.5	16	<=0.5	16	>2
V	2006-S01-025	Urine	76	<=2	>16	<=0.5	8	<=0.5	4	0.12
V	2006-S01-028	Urine	78	<=2	4	<=0.5	4	<=0.5	<=2	<=0.03
V	2006-S01-029	Urine	21	<=2	>16	<=0.5	8	<=0.5	<=2	<=0.03
V	2006-S01-033	Urine	76	8	>16	<=0.5	8	<=0.5	4	1
V	2006-S01-036	Urine	68	<=2	>16	<=0.5	8	<=0.5	4	0.12
V	2006-S01-041	Urine	41	<=2	>16	<=0.5	8	<=0.5	4	<=0.03
V	2006-S01-042	Urine	73	4	>16	<=0.5	8	<=0.5	16	>2
V	2006-S01-046	Urine	80	4	>16	<=0.5	16	<=0.5	16	>2
V	2006-S01-048	Urine	22	<=2	>16	<=0.5	16	<=0.5	8	<=0.03
V	2006-S01-050	Urine	68	4	4	<=0.5	4	<=0.5	<=2	<=0.03
V	2006-S01-211	Blood	59	<=2	>16	<=0.5	>16	<=0.5	8	<=0.03
V	2006-S01-217	Blood	76	<=2	>16	<=0.5	16	<=0.5	8	0.5
V	2006-S01-218	Blood	76	<=2	>16	<=0.5	16	<=0.5	8	>2

V	2006-S01-219	Blood	31	<=2	>16	<=0.5	8	<=0.5	<=2	<=0.03
V	2006-C01-002	Urine	66	<=2	>16	<=0.5	8	<=0.5	<=2	<=0.03
V	2006-C01-003	Urine	5	<=2	>16	<=0.5	16	<=0.5	8	0.12
V	2006-C01-004	Urine	26	<=2	2	<=0.5	2	<=0.5	<=2	<=0.03
V	2006-C01-008	Urine	52	<=2	>16	<=0.5	16	<=0.5	>16	<=0.03
V	2006-C01-009	Urine	76	4	>16	<=0.5	8	<=0.5	<=2	0.25
V	2006-C01-014	Blood	74	<=2	2	<=0.5	4	<=0.5	<=2	0.12
V	2006-C01-015	Blood	65	<=2	>16	<=0.5	8	<=0.5	4	<=0.03
V	2006-C01-016	Blood	57	<=2	2	<=0.5	1	<=0.5	<=2	<=0.03
V	2006-C01-018	Urine	73	4	2	<=0.5	4	<=0.5	<=2	<=0.03
V	2006-C01-019	Urine	49	<=2	>16	<=0.5	8	<=0.5	8	0.12
V	2006-C01-021	Urine	1	4	>16	<=0.5	8	<=0.5	4	0.25
V	2006-C01-024	Urine	83	<=2	>16	<=0.5	8	<=0.5	<=2	0.25
V	2006-C01-026	Urine	5	<=2	>16	<=0.5	8	<=0.5	8	<=0.03
V	2006-C01-029	Other	70	8	4	<=0.5	8	<=0.5	<=2	<=0.03
V	2006-C01-030	Other	60	<=2	>16	<=0.5	8	<=0.5	8	<=0.03
V	2006-C01-034	Blood	69	<=2	>16	<=0.5	8	<=0.5	8	<=0.03
V	2006-C01-037	Urine	50	4	>16	<=0.5	16	<=0.5	>16	<=0.03
V	2006-C01-039	Urine	4	4	2	<=0.5	4	<=0.5	<=2	<=0.03
V	2006-C01-040	Urine	83	<=2	2	<=0.5	2	<=0.5	<=2	<=0.03
V	2006-C01-041	Urine	78	4	>16	8	>16	16	>16	0.25
V	2006-C01-048	Blood	77	<=2	4	<=0.5	4	<=0.5	4	<=0.03
V	2006-C01-049	Urine	53	<=2	>16	<=0.5	8	<=0.5	4	<=0.03
V	2006-C01-050	Urine	44	<=2	4	<=0.5	4	<=0.5	<=2	0.12
V	2006-C01-202	Blood	77	<=2	>16	<=0.5	8	<=0.5	<=2	<=0.03
V	2006-C02-002	Urine	80	4	>16	2	>16	16	>16	<=0.03
V	2006-C02-008	Urine	68	4	>16	4	>16	16	>16	<=0.03
V	2006-C02-009	Urine	57	4	>16	<=0.5	8	<=0.5	4	0.25
V	2006-C02-010	Urine	63	4	>16	<=0.5	8	<=0.5	4	<=0.03
V	2006-C02-011	Urine	36	<=2	<=1	<=0.5	4	<=0.5	<=2	<=0.03
V	2006-C02-012	Urine	33	<=2	>16	<=0.5	4	<=0.5	<=2	<=0.03
V	2006-C02-027	Other	40	4	>16	<=0.5	4	<=0.5	<=2	>2
V	2006-C02-028	Urine	77	4	>16	8	>16	>16	>16	>2
V	2006-C02-032	Urine	36	<=2	>16	<=0.5	8	<=0.5	8	0.25
V	2006-C02-048	Blood	56	<=2	>16	<=0.5	8	<=0.5	4	<=0.03
V	2006-C02-194	Urine	33	4	>16	<=0.5	8	<=0.5	4	<=0.03
V	2006-C02-206	Blood	27	<=2	>16	<=0.5	8	<=0.5	<=2	<=0.03
V	2006-C02-209	Blood	69	<=2	>16	<=0.5	8	<=0.5	8	<=0.03
V	2006-C02-212	Blood	74	<=2	>16	<=0.5	8	<=0.5	<=2	0.25
V	2006-C02-213	Blood	81	8	>16	<=0.5	8	<=0.5	8	0.5
V	2006-C02-216	Blood	71	4	>16	<=0.5	8	<=0.5	<=2	0.25
V	2006-C02-218	Blood	85	<=2	<=1	<=0.5	2	<=0.5	<=2	<=0.03
V	2006-C04-003	Urine	49	<=2	2	<=0.5	4	<=0.5	<=2	<=0.03
V	2006-C04-012	Urine	69	<=2	>16	4	>16	>16	>16	>2
V	2006-C04-019	Urine	45	<=2	>16	<=0.5	8	<=0.5	<=2	<=0.03
V	2006-C04-022	Urine	85	<=2	>16	<=0.5	16	<=0.5	16	>2
V	2006-C04-032	Urine	77	<=2	2	<=0.5	2	<=0.5	<=2	0.25
V	2006-C04-192	Urine	8	<=2	>16	<=0.5	16	<=0.5	16	<=0.03
V	2006-C04-201	Blood	50	<=2	>16	<=0.5	16	<=0.5	4	>2
V	2006-C04-205	Blood	67	<=2	>16	<=0.5	8	<=0.5	<=2	<=0.03
V	2006-C04-208	Blood	62	4	4	<=0.5	4	<=0.5	<=2	<=0.03
V	2006-C04-213	Blood	40	<=2	>16	<=0.5	8	<=0.5	<=2	<=0.03
V	2006-C04-217	Blood	54	<=2	4	<=0.5	4	<=0.5	<=2	<=0.03
V	2006-S05-001	Urine	49	<=2	>16	<=0.5	16	<=0.5	16	<=0.03
V	2006-S05-007	Urine	99	<=2	>16	16	>16	>16	>16	>2
V	2006-S05-008	Other	43	<=2	>16	<=0.5	8	<=0.5	8	<=0.03
V	2006-S05-014	Urine	58	<=2	2	<=0.5	2	<=0.5	<=2	<=0.03
V	2006-S05-015	Urine	47	4	>16	<=0.5	16	<=0.5	4	<=0.03
V	2006-S05-016	Urine	81	<=2	>16	<=0.5	16	<=0.5	4	>2
V	2006-S05-026	Urine	1	4	>16	<=0.5	8	<=0.5	4	<=0.03
V	2006-S05-030	Other	40	<=2	4	<=0.5	8	<=0.5	<=2	<=0.03

V	2006-S05-032	Urine	36	<=2	>16	<=0.5	16	<=0.5	16	0.25
V	2006-S05-035	Urine	74	8	>16	<=0.5	16	<=0.5	16	0.5
V	2006-S05-039	Urine	28	<=2	>16	<=0.5	8	<=0.5	<=2	<=0.03
V	2006-S05-041	Urine	21	<=2	4	<=0.5	4	<=0.5	<=2	<=0.03
V	2006-S05-042	Urine	38	<=2	>16	<=0.5	8	<=0.5	<=2	<=0.03
V	2006-S05-043	Urine	44	4	2	<=0.5	4	<=0.5	<=2	0.5
V	2006-S05-048	Urine	14	4	2	<=0.5	4	<=0.5	<=2	0.5
V	2006-S05-050	Blood	67	4	>16	<=0.5	8	<=0.5	4	0.12
V	2006-S05-193	Urine	0	8	<=1	<=0.5	2	<=0.5	<=2	<=0.03
V	2006-S05-203	Blood	69	<=2	>16	<=0.5	4	<=0.5	<=2	<=0.03
V	2006-S05-204	Blood	82	<=2	>16	<=0.5	8	<=0.5	<=2	<=0.03
V	2006-S06-003	Other	57	<=2	>16	<=0.5	4	<=0.5	<=2	<=0.03
V	2006-S06-005	Urine	91	4	4	<=0.5	4	<=0.5	<=2	<=0.03
V	2006-S06-006	Other	58	4	>16	<=0.5	8	<=0.5	<=2	<=0.03
V	2006-S06-007	Urine	6	<=2	>16	<=0.5	8	<=0.5	<=2	0.12
V	2006-S06-009	Urine	22	<=2	>16	<=0.5	8	<=0.5	8	<=0.03
V	2006-S06-010	Urine	72	<=2	>16	<=0.5	16	<=0.5	4	0.25
V	2006-S06-011	Urine	52	4	>16	<=0.5	4	<=0.5	<=2	<=0.03
V	2006-S06-012	Urine	87	<=2	<=1	<=0.5	1	<=0.5	<=2	<=0.03
V	2006-S06-013	Urine	55	<=2	>16	<=0.5	8	<=0.5	4	<=0.03
V	2006-S06-014	Urine	38	4	>16	<=0.5	16	<=0.5	8	<=0.03
V	2006-S06-015	Other	83	<=2	>16	<=0.5	8	<=0.5	<=2	<=0.03
V	2006-S06-016	Blood	86	4	>16	16	>16	8	>16	1
V	2006-S06-024	Urine	23	<=2	>16	<=0.5	8	<=0.5	<=2	<=0.03
V	2006-S06-025	Urine	39	<=2	>16	<=0.5	8	<=0.5	<=2	0.12
V	2006-S06-026	Other	81	4	4	<=0.5	4	<=0.5	<=2	<=0.03
V	2006-S06-029	Urine	60	<=2	>16	<=0.5	4	<=0.5	<=2	<=0.03
V	2006-S06-030	Urine	34	4	>16	<=0.5	8	<=0.5	4	<=0.03
V	2006-S06-031	Other	45	4	>16	<=0.5	8	<=0.5	4	<=0.03
V	2006-S06-035	Other	41	<=2	4	<=0.5	4	<=0.5	<=2	<=0.03
V	2006-S06-037	Urine	33	<=2	>16	<=0.5	8	<=0.5	4	0.5
V	2006-S06-043	Urine	49	8	4	<=0.5	4	<=0.5	<=2	<=0.03
V	2006-S06-047	Urine	45	4	2	<=0.5	4	<=0.5	<=2	0.25
V	2006-S06-212	Blood	31	4	2	<=0.5	2	<=0.5	<=2	<=0.03
V	2006-S07-002	Blood	62	4	>16	<=0.5	4	<=0.5	4	0.5
V	2006-S07-003	Urine	8	4	>16	<=0.5	16	<=0.5	8	>2
V	2006-S07-004	Urine	64	<=2	>16	<=0.5	16	<=0.5	4	0.12
V	2006-S07-005	Urine	86	<=2	>16	<=0.5	8	<=0.5	<=2	<=0.03
V	2006-S07-007	Urine	1	<=2	>16	<=0.5	16	<=0.5	16	<=0.03
V	2006-S07-011	Urine	43	4	>16	<=0.5	8	<=0.5	<=2	<=0.03
V	2006-S07-012	Urine	56	<=2	>16	<=0.5	16	<=0.5	8	0.25
V	2006-S07-013	Other	75	<=2	>16	<=0.5	8	<=0.5	4	>2
V	2006-S07-015	Urine	47	<=2	>16	<=0.5	8	<=0.5	<=2	0.25
V	2006-S07-019	Urine	67	<=2	>16	<=0.5	8	<=0.5	4	<=0.03
V	2006-S07-027	Urine	86	<=2	4	<=0.5	4	<=0.5	<=2	<=0.03
V	2006-S07-028	Urine	68	<=2	>16	4	>16	16	>16	>2
V	2006-S07-033	Urine	57	<=2	>16	<=0.5	16	<=0.5	16	<=0.03
V	2006-S07-034	Urine	53	<=2	2	<=0.5	4	<=0.5	<=2	<=0.03
V	2006-S07-035	Urine	52	<=2	>16	<=0.5	8	<=0.5	<=2	<=0.03
V	2006-S07-036	Urine	67	<=2	4	<=0.5	4	<=0.5	<=2	<=0.03
V	2006-S07-038	Urine	3	<=2	>16	<=0.5	8	<=0.5	<=2	0.25
V	2006-S07-041	Urine	28	<=2	>16	<=0.5	8	<=0.5	<=2	0.25
V	2006-S07-042	Urine	59	<=2	>16	<=0.5	8	<=0.5	<=2	0.25
V	2006-S07-045	Urine	59	<=2	>16	<=0.5	16	<=0.5	>16	<=0.03
V	2006-S07-046	Other	8	<=2	>16	<=0.5	8	<=0.5	4	<=0.03
V	2006-S07-048	Urine	17	<=2	4	<=0.5	8	<=0.5	<=2	<=0.03
V	2006-S07-049	Urine	85	4	>16	<=0.5	8	<=0.5	<=2	0.5
V	2006-S07-050	Other	68	<=2	>16	<=0.5	4	<=0.5	<=2	1
V	2006-S07-184	Other	10	8	>16	<=0.5	8	<=0.5	4	<=0.03
V	2006-S07-207	Blood	72	4	<=1	<=0.5	<=0.5	<=0.5	<=2	<=0.03
V	2006-S07-212	Blood	36	<=2	>16	<=0.5	8	<=0.5	8	0.25

V	2006-S07-216	Blood	77	8	>16	<=0.5	8	<=0.5	4	<=0.03
V	2006-S07-219	Blood	81	16	>16	<=0.5	8	<=0.5	4	<=0.03
V	2006-N05-001	Urine	unk	<=2	2	<=0.5	4	<=0.5	<=2	<=0.03
V	2006-N05-002	Urine	1	4	2	<=0.5	4	<=0.5	<=2	<=0.03
V	2006-N05-008	Urine	84	<=2	2	<=0.5	2	<=0.5	<=2	<=0.03
V	2006-N05-010	Urine		4	>16	<=0.5	4	<=0.5	4	<=0.03
V	2006-N05-012	Urine	71	4	>16	<=0.5	8	<=0.5	8	0.25
V	2006-N05-013	Urine	61	<=2	2	<=0.5	2	<=0.5	<=2	0.12
V	2006-N05-019	Urine	30	<=2	2	<=0.5	4	<=0.5	<=2	0.25
V	2006-N05-020	Urine	73	4	2	<=0.5	4	<=0.5	<=2	<=0.03
V	2006-N05-024	Blood		<=2	2	<=0.5	4	<=0.5	<=2	<=0.03
V	2006-N05-028	Urine		4	>16	<=0.5	8	<=0.5	8	<=0.03
V	2006-N05-029	Urine	75	<=2	4	<=0.5	4	<=0.5	4	0.25
V	2006-N05-030	Urine	62	<=2	>16	<=0.5	8	<=0.5	<=2	<=0.03
V	2006-N05-031	Urine	unk	<=2	>16	<=0.5	8	<=0.5	4	>2
V	2006-N05-032	Urine	69	8	>16	<=0.5	8	<=0.5	<=2	>2
V	2006-N05-036	Urine	28	<=2	>16	<=0.5	8	<=0.5	<=2	1
V	2006-N05-037	Urine	33	<=2	>16	<=0.5	8	<=0.5	<=2	<=0.03
V	2006-N05-039	Urine		4	4	<=0.5	4	<=0.5	<=2	0.12
V	2006-N05-040	Urine		<=2	>16	<=0.5	8	<=0.5	<=2	0.25
V	2006-N05-041	Urine	55	<=2	8	<=0.5	8	<=0.5	4	>2
V	2006-N05-043	Urine		4	4	<=0.5	4	<=0.5	<=2	<=0.03
V	2006-N05-045	Urine		<=2	2	<=0.5	4	<=0.5	<=2	<=0.03
V	2006-N05-046	Urine		<=2	>16	<=0.5	8	<=0.5	<=2	0.12
V	2006-N05-050	Urine		<=2	>16	<=0.5	8	<=0.5	4	<=0.03
V	2006-N06-001	Urine	21	4	>16	<=0.5	16	<=0.5	4	<=0.03
V	2006-N06-006	Urine	70	<=2	4	<=0.5	4	<=0.5	<=2	<=0.03
V	2006-N06-007	Urine	66	<=2	>16	<=0.5	16	<=0.5	8	>2
V	2006-N06-011	Urine	89	<=2	>16	16	>16	>16	>16	>2
V	2006-N06-014	Urine	35	4	>16	<=0.5	8	<=0.5	4	<=0.03
V	2006-N06-016	Urine	1	4	>16	<=0.5	>16	1	>16	<=0.03
V	2006-N06-026	Urine	74	<=2	>16	<=0.5	>16	<=0.5	>16	>2
V	2006-N06-027	Urine	89	4	8	<=0.5	8	<=0.5	16	>2
V	2006-N06-028	Urine	44	8	>16	<=0.5	8	<=0.5	4	<=0.03
V	2006-N06-033	Other	78	<=2	>16	<=0.5	8	<=0.5	4	0.25
V	2006-N06-034	Urine	82	<=2	>16	<=0.5	8	<=0.5	4	0.25
V	2006-N06-035	Urine	30	<=2	>16	<=0.5	4	<=0.5	<=2	<=0.03
V	2006-N06-038	Urine	75	<=2	>16	<=0.5	8	<=0.5	<=2	0.12
V	2006-N06-039	Urine	31	4	>16	<=0.5	>16	<=0.5	4	<=0.03
V	2006-N06-046	Other	80	4	>16	>16	16	4	>16	<=0.03
V	2006-N06-211	Blood	79	4	>16	8	>16	16	>16	>2
V	2006-N06-212	Blood	87	<=2	2	<=0.5	4	<=0.5	<=2	<=0.03
V	2006-N06-214	Blood	67	<=2	4	<=0.5	8	<=0.5	<=2	<=0.03
V	2006-N06-217	Blood	85	4	4	<=0.5	4	<=0.5	<=2	0.25
V	2006-N07-001	Blood	85	<=2	4	<=0.5	8	<=0.5	4	<=0.03
V	2006-N07-002	Blood	76	<=2	>16	<=0.5	8	<=0.5	<=2	0.12
V	2006-N07-004	Urine	54	4	>16	<=0.5	8	<=0.5	16	0.25
V	2006-N07-010	Urine	94	4	>16	<=0.5	16	<=0.5	8	<=0.03
V	2006-N07-013	Other	35	<=2	>16	<=0.5	16	<=0.5	8	<=0.03
V	2006-N07-014	Urine	74	<=2	>16	<=0.5	8	<=0.5	4	0.5
V	2006-N07-016	Urine	66	<=2	<=1	<=0.5	2	<=0.5	<=2	<=0.03
V	2006-N07-019	Blood	59	8	4	<=0.5	8	<=0.5	4	<=0.03
V	2006-N07-020	Urine	83	<=2	>16	16	>16	>16	>16	>2
V	2006-N07-021	Urine	69	8	>16	<=0.5	>16	<=0.5	>16	>2
V	2006-N07-022	Blood	72	<=2	>16	<=0.5	8	<=0.5	4	<=0.03
V	2006-N07-027	Urine	42	8	>16	<=0.5	16	<=0.5	>16	<=0.03
V	2006-N07-028	Blood	91	<=2	>16	>16	>16	>16	>16	>2
V	2006-N07-032	Blood	70	4	>16	<=0.5	16	<=0.5	8	0.25
V	2006-N07-033	Blood	82	4	>16	<=0.5	4	<=0.5	<=2	<=0.03
V	2006-N07-034	Urine	75	<=2	>16	<=0.5	8	<=0.5	4	<=0.03
V	2006-N07-036	Urine	47	<=2	>16	<=0.5	>16	<=0.5	>16	<=0.03

V	2006-N07-037	Urine	20	4	4	<=0.5	4	<=0.5	<=2	<=0.03
V	2006-N07-040	Urine	31	4	2	<=0.5	4	<=0.5	<=2	<=0.03
V	2006-N07-041	Urine	64	<=2	>16	<=0.5	16	<=0.5	8	0.12
V	2006-N07-043	Blood	71	<=2	4	<=0.5	4	<=0.5	<=2	<=0.03
V	2006-N07-044	Urine	21	<=2	8	<=0.5	4	<=0.5	<=2	<=0.03
V	2006-N07-045	Urine	75	4	>16	<=0.5	8	<=0.5	4	<=0.03
V	2006-N07-048	Blood	69	<=2	2	<=0.5	2	<=0.5	<=2	0.25
V	2006-N07-210	Blood	64	<=2	2	<=0.5	4	<=0.5	<=2	<=0.03
V	2006-N07-216	Blood	72	4	4	<=0.5	4	<=0.5	<=2	0.25
V	2006-N07-219	Blood	65	4	>16	<=0.5	4	<=0.5	4	<=0.03
V	2006-S08-001	Urine	48	8	>16	<=0.5	8	<=0.5	4	0.12
V	2006-S08-004	Urine	23	4	>16	<=0.5	8	<=0.5	4	<=0.03
V	2006-S08-006	Urine	77	4	4	<=0.5	4	<=0.5	<=2	>2
V	2006-S08-007	Urine	81	<=2	<=1	<=0.5	1	<=0.5	<=2	<=0.03
V	2006-S08-011	Urine	81	4	<=1	<=0.5	1	<=0.5	<=2	<=0.03
V	2006-S08-013	Urine	56	4	>16	8	>16	16	>16	0.12
V	2006-S08-016	Urine	81	<=2	>16	<=0.5	8	<=0.5	<=2	<=0.03
V	2006-S08-017	Urine	82	4	>16	<=0.5	8	<=0.5	16	<=0.03
V	2006-S08-019	Urine	79	4	>16	8	>16	16	>16	>2
V	2006-S08-022	Urine	53	4	>16	<=0.5	16	<=0.5	16	<=0.03
V	2006-S08-023	Urine	22	<=2	>16	<=0.5	16	<=0.5	>16	<=0.03
V	2006-S08-026	Urine	35	8	2	<=0.5	4	<=0.5	<=2	<=0.03
V	2006-S08-033	Urine	82	4	>16	<=0.5	16	1	8	0.5
V	2006-S08-039	Urine	55	<=2	>16	<=0.5	16	<=0.5	<=2	<=0.03
V	2006-S08-040	Other	74	8	<=1	<=0.5	4	<=0.5	<=2	<=0.03
V	2006-S08-041	Urine	68	8	>16	<=0.5	8	<=0.5	4	<=0.03
V	2006-S08-043	Urine	53	<=2	4	<=0.5	4	<=0.5	4	<=0.03
V	2006-S08-045	Urine	8	<=2	>16	<=0.5	8	<=0.5	<=2	>2
V	2006-S08-046	Urine	81	8	4	<=0.5	4	<=0.5	<=2	0.25
V	2006-E02-002	Urine	73	<=2	>16	<=0.5	8	<=0.5	4	0.25
V	2006-E02-003	Urine	41	<=2	4	<=0.5	4	<=0.5	4	<=0.03
V	2006-E02-005	Urine	78	8	>16	<=0.5	8	<=0.5	8	0.06
V	2006-E02-006	Urine	23	<=2	>16	<=0.5	8	<=0.5	4	>2
V	2006-E02-009	Urine	56	8	>16	<=0.5	16	<=0.5	<=2	>2
V	2006-E02-011	Urine	53	<=2	>16	<=0.5	8	<=0.5	<=2	0.25
V	2006-E02-015	Urine	56	4	>16	<=0.5	8	<=0.5	8	>2
V	2006-E02-017	Urine	55	<=2	>16	<=0.5	16	<=0.5	>16	<=0.03
V	2006-E02-019	Urine	44	<=2	>16	<=0.5	8	<=0.5	<=2	0.12
V	2006-E02-022	Other	81	<=2	>16	<=0.5	8	<=0.5	<=2	0.12
V	2006-E02-024	Urine	53	<=2	>16	<=0.5	16	<=0.5	8	0.25
V	2006-E02-030	Urine	49	<=2	>16	<=0.5	8	<=0.5	<=2	<=0.03
V	2006-E02-033	Urine	69	<=2	>16	<=0.5	8	<=0.5	<=2	<=0.03
V	2006-E02-046	Urine	62	8	2	<=0.5	4	<=0.5	<=2	<=0.03
V	2006-E02-048	Urine	81	<=2	>16	<=0.5	8	<=0.5	<=2	0.12
V	2006-E02-203	Blood	73	<=2	2	<=0.5	4	<=0.5	<=2	<=0.03
V	2006-E02-205	Blood	67	4	2	<=0.5	4	<=0.5	<=2	<=0.03
V	2006-E02-213	Blood	1	<=2	>16	<=0.5	8	<=0.5	<=2	<=0.03
V	2006-E02-216	Blood	82	4	>16	<=0.5	8	<=0.5	<=2	0.25
V	2006-E02-217	Blood	53	<=2	>16	<=0.5	8	<=0.5	4	<=0.03
V	2006-N08-001	Blood	83	<=2	>16	8	>16	16	>16	>2
V	2006-N08-008	Urine	17	4	4	<=0.5	4	<=0.5	<=2	<=0.03
V	2006-N08-009	Urine	40	<=2	>16	<=0.5	8	<=0.5	<=2	<=0.03
V	2006-N08-011	Urine	17	4	2	<=0.5	4	<=0.5	<=2	<=0.03
V	2006-N08-015	Urine	27	<=2	>16	<=0.5	8	<=0.5	<=2	0.25
V	2006-N08-018	Urine	26	<=2	>16	<=0.5	8	<=0.5	<=2	0.12
V	2006-N08-019	Urine	56	4	>16	<=0.5	8	<=0.5	<=2	<=0.03
V	2006-N08-021	Urine	32	<=2	4	<=0.5	4	<=0.5	<=2	<=0.03
V	2006-N08-022	Urine	40	<=2	>16	2	>16	4	>16	0.25
V	2006-N08-023	Urine	23	4	4	<=0.5	4	<=0.5	4	<=0.03
V	2006-N08-024	Urine	36	<=2	4	<=0.5	4	<=0.5	<=2	<=0.03
V	2006-N08-025	Urine	79	4	>16	<=0.5	8	<=0.5	4	<=0.03

V	2006-N08-026	Urine	39	8	>16	<=0.5	8	<=0.5	<=2	<=0.03
V	2006-N08-028	Urine	73	<=2	4	<=0.5	4	<=0.5	<=2	<=0.03
V	2006-N08-029	Urine	9	<=2	>16	<=0.5	8	<=0.5	>16	0.25
V	2006-N08-031	Urine	81	<=2	4	<=0.5	4	<=0.5	<=2	0.25
V	2006-N08-032	Urine	66	<=2	>16	<=0.5	4	<=0.5	4	<=0.03
V	2006-N08-034	Urine	31	8	>16	<=0.5	8	<=0.5	<=2	<=0.03
V	2006-N08-036	Urine	24	<=2	>16	<=0.5	16	<=0.5	16	>2
V	2006-N08-042	Urine	64	8	>16	<=0.5	8	<=0.5	4	<=0.03
V	2006-N08-047	Urine	19	<=2	4	<=0.5	4	<=0.5	<=2	0.25
V	2006-N08-194	Urine	0	<=2	4	<=0.5	4	<=0.5	<=2	<=0.03
V	2006-N08-208	Blood	77	4	4	<=0.5	4	<=0.5	<=2	<=0.03
V	2006-S09-002	Urine	69	4	4	<=0.5	4	<=0.5	<=2	<=0.03
V	2006-S09-003	Urine	26	4	>16	<=0.5	8	<=0.5	<=2	<=0.03
V	2006-S09-011	Urine	80	4	2	<=0.5	4	<=0.5	<=2	0.25
V	2006-S09-017	Urine	29	4	4	<=0.5	4	<=0.5	<=2	<=0.03
V	2006-S09-021	Urine	63	4	4	<=0.5	4	<=0.5	<=2	<=0.03
V	2006-S09-027	Urine	81	<=2	>16	<=0.5	8	<=0.5	4	>2
V	2006-S09-031	Urine	68	<=2	>16	<=0.5	8	<=0.5	<=2	>2
V	2006-S09-033	Urine	70	<=2	>16	<=0.5	8	<=0.5	<=2	0.25
V	2006-S09-034	Urine	52	<=2	>16	<=0.5	>16	<=0.5	16	<=0.03
V	2006-S09-046	Blood	49	<=2	4	<=0.5	4	<=0.5	<=2	<=0.03
V	2006-S09-047	Blood	78	<=2	>16	<=0.5	4	<=0.5	4	<=0.03
V	2006-S09-201	Blood	57	8	>16	>16	16	4	>16	>2
V	2006-S09-202	Blood	45	4	4	<=0.5	4	<=0.5	<=2	1
V	2006-S09-205	Blood	56	<=2	>16	<=0.5	8	<=0.5	4	<=0.03
V	2006-S09-213	Blood	72	<=2	>16	<=0.5	8	<=0.5	<=2	0.12
V	2006-S09-214	Blood	72	8	>16	4	16	1	>16	0.25
V	2006-S09-215	Blood	86	<=2	2	<=0.5	4	<=0.5	<=2	<=0.03
V	2006-S09-217	Blood	56	<=2	>16	<=0.5	8	<=0.5	<=2	0.12
V	2006-E03-002	Blood	43	4	>16	<=0.5	8	<=0.5	4	0.12
V	2006-E03-003	Blood	45	<=2	>16	<=0.5	8	<=0.5	4	>2
V	2006-E03-004	Blood	68	<=2	>16	<=0.5	8	<=0.5	<=2	<=0.03
V	2006-E03-005	Urine	78	<=2	>16	<=0.5	8	<=0.5	<=2	<=0.03
V	2006-E03-006	Urine	18	<=2	>16	<=0.5	4	<=0.5	<=2	1
V	2006-E03-009	Urine	54	<=2	>16	<=0.5	8	<=0.5	4	0.25
V	2006-E03-010	Urine	58	<=2	>16	<=0.5	16	<=0.5	4	0.12
V	2006-E03-023	Urine	37	<=2	>16	<=0.5	8	<=0.5	4	<=0.03
V	2006-E03-024	Urine	22	<=2	4	<=0.5	4	<=0.5	<=2	0.06
V	2006-E03-028	Urine	68	<=2	>16	<=0.5	16	<=0.5	8	<=0.03
V	2006-E03-032	Urine	64	<=2	2	<=0.5	4	<=0.5	<=2	<=0.03
V	2006-E03-038	Urine	76	<=2	>16	<=0.5	8	<=0.5	8	0.06
V	2006-E03-042	Urine	76	<=2	>16	<=0.5	8	<=0.5	16	0.5
V	2006-E03-044	Urine	54	4	>16	<=0.5	8	<=0.5	8	>2
V	2006-E03-047	Urine	31	4	>16	<=0.5	8	<=0.5	<=2	0.25
V	2006-E03-215	Blood	51	8	>16	<=0.5	4	<=0.5	<=2	<=0.03
V	2006-C06-001	Blood	45	4	>16	<=0.5	4	<=0.5	4	0.12
V	2006-C06-004	Blood	58	<=2	4	<=0.5	8	<=0.5	<=2	<=0.03
V	2006-C06-006	Urine	51	8	>16	>16	>16	>16	>16	>2
V	2006-C06-007	Urine	59	<=2	4	<=0.5	4	<=0.5	<=2	<=0.03
V	2006-C06-008	Blood	55	4	>16	<=0.5	8	<=0.5	<=2	<=0.03
V	2006-C06-009	Blood	78	8	2	<=0.5	4	<=0.5	<=2	<=0.03
V	2006-C06-019	Urine	76	<=2	>16	<=0.5	8	<=0.5	4	<=0.03
V	2006-C06-020	Urine	45	8	>16	<=0.5	16	<=0.5	16	<=0.03
V	2006-C06-024	Urine	85	<=2	2	<=0.5	4	<=0.5	<=2	0.12
V	2006-C06-025	Blood	71	<=2	4	<=0.5	4	<=0.5	<=2	<=0.03
V	2006-C06-036	Blood	84	4	>16	<=0.5	4	<=0.5	<=2	<=0.03
V	2006-C06-041	Other	72	4	>16	<=0.5	4	<=0.5	<=2	0.5
V	2006-C06-042	Urine	59	4	>16	<=0.5	8	<=0.5	4	0.12
V	2006-C06-050	Urine	88	<=2	>16	2	>16	8	>16	0.25
V	2006-C06-219	Blood	79	<=2	>16	2	16	2	16	>2
V	2006-C07-001	Urine	40	<=2	>16	>16	>16	>16	>16	<=0.03

V	2006-C07-002	Urine	93	4	>16	2	8	1	>16	>2
V	2006-C07-004	Urine	80	4	>16	16	>16	>16	>16	>2
V	2006-C07-007	Urine	71	4	4	<=0.5	8	<=0.5	<=2	0.12
V	2006-C07-008	Urine	66	8	>16	>16	>16	>16	>16	>2
V	2006-C07-009	Urine	74	8	>16	4	>16	>16	>16	>2
V	2006-C07-010	Urine	73	8	>16	<=0.5	8	<=0.5	8	>2
V	2006-C07-015	Urine	33	4	>16	<=0.5	8	<=0.5	<=2	<=0.03
V	2006-C07-016	Urine	73	<=2	>16	8	>16	16	>16	>2
V	2006-C07-018	Blood	44	<=2	4	2	8	<=0.5	<=2	<=0.03
V	2006-C07-019	Blood	60	<=2	>16	<=0.5	8	<=0.5	<=2	<=0.03
V	2006-C07-022	Urine	84	<=2	>16	1	>16	4	>16	>2
V	2006-C07-025	Urine	65	4	>16	<=0.5	8	<=0.5	<=2	<=0.03
V	2006-C07-026	Urine	62	4	>16	<=0.5	8	<=0.5	<=2	0.25
V	2006-C07-027	Other	43	<=2	>16	<=0.5	8	<=0.5	<=2	>2
V	2006-C07-033	Urine	74	<=2	>16	<=0.5	>16	<=0.5	<=2	>2
V	2006-C07-035	Urine	78	<=2	>16	>16	>16	>16	>16	>2
V	2006-C07-036	Urine	78	<=2	>16	16	>16	>16	>16	>2
V	2006-C07-038	Urine	67	<=2	2	16	>16	8	8	0.25
V	2006-C07-040	Blood	62	4	>16	<=0.5	8	<=0.5	<=2	<=0.03
V	2006-C07-041	Urine	71	<=2	2	<=0.5	4	<=0.5	<=2	<=0.03
V	2006-C07-043	Blood	70	<=2	2	<=0.5	4	<=0.5	<=2	<=0.03
V	2006-C07-050	Blood	84	<=2	>16	<=0.5	8	<=0.5	4	>2
V	2006-C07-205	Blood	51	<=2	>16	<=0.5	16	<=0.5	4	0.25
V	2006-C07-216	Blood	81	8	>16	4	>16	2	>16	0.25
V	2006-C07-217	Blood	50	<=2	>16	<=0.5	8	<=0.5	<=2	0.12
V	2006-N16-001	Urine	83	8	<=1	<=0.5	4	<=0.5	<=2	<=0.03
V	2006-N16-002	Urine	81	4	>16	<=0.5	16	<=0.5	<=2	<=0.03
V	2006-N16-008	Blood	86	4	>16	<=0.5	8	<=0.5	4	>2
V	2006-N16-011	Blood	55	<=2	>16	16	>16	16	>16	1
V	2006-N16-016	Urine	68	<=2	>16	>16	>16	>16	>16	>2
V	2006-N16-020	Other	35	4	>16	<=0.5	8	<=0.5	<=2	0.5
V	2006-N16-021	Blood	88	4	>16	<=0.5	16	<=0.5	4	>2
V	2006-N16-024	Other	71	<=2	>16	<=0.5	8	<=0.5	4	<=0.03
V	2006-N16-027	Urine	66	<=2	>16	<=0.5	8	<=0.5	4	>2
V	2006-N16-031	Blood	72	4	>16	<=0.5	8	<=0.5	<=2	<=0.03
V	2006-N16-032	Urine	85	<=2	2	<=0.5	4	<=0.5	<=2	<=0.03
V	2006-N16-036	Urine	19	8	4	<=0.5	4	<=0.5	<=2	0.25
V	2006-N16-038	Urine	83	4	>16	<=0.5	8	<=0.5	>16	>2
V	2006-N16-040	Blood	63	4	4	<=0.5	4	<=0.5	<=2	0.25
V	2006-N16-044	Other	2	<=2	2	<=0.5	4	<=0.5	<=2	<=0.03
V	2006-N16-048	Urine	69	<=2	>16	>16	>16	>16	>16	>2
V	2006-N16-050	Urine	46	4	4	<=0.5	4	<=0.5	<=2	<=0.03
V	2006-N16-202	Blood	70	<=2	>16	4	>16	16	>16	<=0.03
V	2006-N16-203	Blood	57	4	>16	16	>16	>16	>16	>2
V	2006-N16-206	Blood	79	<=2	>16	<=0.5	8	<=0.5	4	0.5
V	2006-N16-216	Blood	82	4	2	<=0.5	4	<=0.5	<=2	0.25
V	2006-N19-001	Urine	43	4	4	<=0.5	4	<=0.5	4	<=0.03
V	2006-N19-002	Urine	24	8	>16	<=0.5	8	<=0.5	<=2	0.25
V	2006-N19-006	Urine	35	16	>16	<=0.5	16	<=0.5	4	>2
V	2006-N19-007	Urine	38	4	>16	<=0.5	8	<=0.5	8	>2
V	2006-N19-010	Urine	45	4	>16	<=0.5	8	<=0.5	4	<=0.03
V	2006-N19-011	Urine	80	<=2	>16	<=0.5	8	<=0.5	<=2	0.25
V	2006-N19-012	Urine	22	8	4	<=0.5	4	<=0.5	<=2	0.5
V	2006-N19-018	Urine	57	<=2	4	<=0.5	8	<=0.5	<=2	>2
V	2006-N19-022	Urine	64	8	4	<=0.5	8	<=0.5	<=2	<=0.03
V	2006-N19-024	Blood	50	4	4	<=0.5	4	<=0.5	<=2	0.25
V	2006-N19-026	Urine	41	4	>16	<=0.5	8	<=0.5	4	<=0.03
V	2006-N19-027	Other	37	<=2	>16	<=0.5	8	<=0.5	<=2	0.12
V	2006-N19-033	Urine	25	8	>16	<=0.5	8	<=0.5	8	0.12
V	2006-N19-034	Urine	77	4	4	<=0.5	4	<=0.5	<=2	<=0.03
V	2006-N19-035	Urine	42	4	4	<=0.5	4	<=0.5	<=2	<=0.03

V	2006-N19-037	Urine	39	<=2	>16	<=0.5	16	<=0.5	8	<=0.03
V	2006-N19-046	Urine	18	<=2	4	<=0.5	4	<=0.5	<=2	<=0.03
V	2006-N19-047	Urine	85	>32	>16	8	>16	2	>16	>2
V	2006-N19-048	Urine	42	4	8	<=0.5	4	<=0.5	<=2	<=0.03
V	2006-N19-050	Urine	88	4	>16	8	>16	16	>16	>2
V	2006-N19-201	Blood	35	4	>16	<=0.5	8	<=0.5	4	<=0.03
V	2006-N19-202	Blood	87	<=2	>16	<=0.5	8	<=0.5	4	0.5
V	2006-N19-204	Blood	69	4	2	<=0.5	4	<=0.5	<=2	<=0.03
V	2006-N19-207	Blood	78	4	>16	<=0.5	8	<=0.5	<=2	<=0.03
V	2006-N19-208	Blood	72	<=2	>16	<=0.5	8	<=0.5	<=2	<=0.03
V	2006-N19-209	Blood	32	4	4	<=0.5	4	<=0.5	<=2	<=0.03
V	2006-N19-210	Blood	77	4	>16	<=0.5	8	<=0.5	<=2	0.25
V	2006-N19-211	Blood	71	4	>16	<=0.5	16	<=0.5	16	<=0.03
V	2006-N19-213	Blood	79	4	2	<=0.5	4	<=0.5	<=2	<=0.03
V	2006-N19-215	Blood	62	4	>16	<=0.5	8	<=0.5	4	>2
V	2006-N19-217	Blood	79	8	>16	<=0.5	8	<=0.5	8	<=0.03
V	2006-C08-004	Urine	54	<=2	>16	<=0.5	16	<=0.5	8	<=0.03
V	2006-C08-009	Urine	68	<=2	>16	16	>16	>16	>16	>2
V	2006-C08-028	Urine	7	<=2	>16	<=0.5	8	<=0.5	<=2	<=0.03
V	2006-C08-030	Urine	25	<=2	>16	4	>16	16	>16	>2
V	2006-C08-031	Urine	0	8	>16	<=0.5	16	<=0.5	<=2	<=0.03
V	2006-C08-035	Urine	69	<=2	>16	<=0.5	8	<=0.5	<=2	0.25
V	2006-C08-039	Other	50	16	>16	<=0.5	16	<=0.5	16	<=0.03
V	2006-C08-042	Urine	25	4	>16	<=0.5	8	<=0.5	<=2	<=0.03
V	2006-C08-044	Other	78	4	>16	<=0.5	4	<=0.5	4	0.5
V	2006-C08-049	Urine	62	8	>16	2	>16	16	>16	>2
V	2006-C08-209	Blood	24	<=2	>16	<=0.5	8	<=0.5	<=2	>2
V	2006-C08-211	Blood	74	8	4	<=0.5	4	<=0.5	<=2	<=0.03
V	2006-S11-002	Urine	55	4	2	<=0.5	2	<=0.5	<=2	<=0.03
V	2006-S11-006	Blood	73	<=2	>16	8	>16	16	>16	>2
V	2006-S11-007	Blood	63	16	>16	<=0.5	8	1	16	0.25
V	2006-S11-009	Urine	25	<=2	4	<=0.5	4	<=0.5	<=2	<=0.03
V	2006-S11-013	Urine	78	<=2	>16	<=0.5	16	<=0.5	8	<=0.03
V	2006-S11-014	Urine	29	<=2	>16	<=0.5	16	<=0.5	16	>2
V	2006-S11-017	Urine	41	<=2	>16	<=0.5	4	<=0.5	<=2	0.5
V	2006-S11-018	Urine	61	<=2	4	<=0.5	4	<=0.5	<=2	0.25
V	2006-S11-024	Urine	51	4	2	<=0.5	4	<=0.5	<=2	>2
V	2006-S11-026	Urine	27	<=2	2	<=0.5	4	<=0.5	<=2	<=0.03
V	2006-S11-027	Urine	82	32	>16	>16	>16	>16	>16	>2
V	2006-S11-033	Urine	37	<=2	>16	<=0.5	8	<=0.5	<=2	0.25
V	2006-S11-034	Blood	87	<=2	>16	<=0.5	8	<=0.5	4	0.25
V	2006-S11-039	Urine	43	4	>16	<=0.5	16	<=0.5	4	0.5
V	2006-S11-041	Urine	56	4	>16	4	8	1	>16	>2
V	2006-S11-042	Urine	82	<=2	4	<=0.5	8	<=0.5	<=2	<=0.03
V	2006-S11-044	Urine	71	4	>16	<=0.5	16	<=0.5	8	<=0.03
V	2006-S11-046	Blood	77	<=2	4	<=0.5	4	<=0.5	<=2	<=0.03
V	2006-S11-047	Blood	76	<=2	4	<=0.5	4	<=0.5	<=2	<=0.03
V	2006-S11-048	Urine	53	4	>16	<=0.5	4	<=0.5	<=2	0.12
V	2006-S11-049	Urine	52	<=2	>16	<=0.5	8	<=0.5	4	0.12
V	2006-S11-187	Urine	6	4	>16	<=0.5	8	<=0.5	4	<=0.03
V	2006-S11-190	Urine	3	<=2	>16	<=0.5	16	<=0.5	8	0.06
V	2006-S11-191	Urine	6	4	>16	<=0.5	16	<=0.5	>16	0.12
V	2006-S11-196	Urine	0	<=2	>16	16	>16	>16	>16	<=0.03
V	2006-S11-199	Urine	10	4	>16	<=0.5	>16	1	>16	>2
V	2006-S11-204	Blood	58	<=2	2	<=0.5	2	<=0.5	<=2	0.12
V	2006-S11-212	Blood	70	<=2	>16	<=0.5	16	<=0.5	8	<=0.03
V	2006-N21-010	Urine	64	4	>16	<=0.5	8	<=0.5	4	0.25
V	2006-N21-012	Urine	55	4	>16	<=0.5	8	<=0.5	16	<=0.03
V	2006-N21-013	Urine	81	<=2	>16	<=0.5	8	<=0.5	<=2	>2
V	2006-N21-015	Urine	43	8	>16	<=0.5	8	<=0.5	8	<=0.03
V	2006-N21-020	Urine	60	4	4	<=0.5	4	<=0.5	<=2	0.25

V	2006-N21-021	Urine	1	4	>16	<=0.5	8	<=0.5	4	1
V	2006-N21-040	Other	83	<=2	>16	<=0.5	8	<=0.5	4	0.25
V	2006-N21-041	Other	31	4	>16	<=0.5	8	<=0.5	<=2	0.25
V	2006-N21-042	Urine	53	<=2	>16	1	8	<=0.5	<=2	<=0.03
V	2006-N21-044	Urine	83	<=2	>16	<=0.5	16	<=0.5	>16	>2
V	2006-N21-047	Urine	0	<=2	>16	4	16	1	>16	0.25
V	2006-N21-198	Urine	0	8	>16	<=0.5	8	<=0.5	<=2	<=0.03
V	2006-N21-215	Blood	70	4	>16	<=0.5	8	<=0.5	4	0.25
V	2006-C09-002	Urine	60	<=2	>16	<=0.5	8	<=0.5	<=2	0.25
V	2006-C09-003	Other	70	<=2	2	<=0.5	2	<=0.5	<=2	<=0.03
V	2006-C09-005	Urine	18	<=2	>16	<=0.5	8	<=0.5	4	<=0.03
V	2006-C09-012	Blood	41	4	>16	<=0.5	16	<=0.5	>16	<=0.03
V	2006-C09-015	Blood	87	4	>16	<=0.5	16	<=0.5	4	<=0.03
V	2006-C09-019	Urine	46	<=2	>16	<=0.5	8	<=0.5	<=2	<=0.03
V	2006-C09-021	Urine	9	4	4	<=0.5	4	<=0.5	<=2	<=0.03
V	2006-C09-022	Urine	64	<=2	4	<=0.5	4	<=0.5	<=2	<=0.03
V	2006-C09-025	Urine	74	<=2	>16	<=0.5	8	<=0.5	<=2	>2
V	2006-C09-026	Urine	72	>32	>16	>16	>16	>16	>16	>2
V	2006-C09-028	Urine	72	<=2	>16	<=0.5	8	<=0.5	4	<=0.03
V	2006-C09-029	Blood	71	<=2	>16	<=0.5	8	<=0.5	<=2	0.25
V	2006-C09-030	Blood	61	8	>16	<=0.5	16	<=0.5	4	<=0.03
V	2006-C09-031	Urine	44	<=2	>16	<=0.5	16	<=0.5	8	<=0.03
V	2006-C09-033	Urine	76	4	>16	>16	>16	>16	>16	<=0.03
V	2006-C09-035	Urine	61	4	>16	<=0.5	>16	<=0.5	8	<=0.03
V	2006-C09-042	Urine	56	<=2	>16	<=0.5	4	<=0.5	<=2	0.25
V	2006-C09-044	Urine	78	<=2	4	<=0.5	4	<=0.5	<=2	<=0.03
V	2006-C09-045	Urine	84	<=2	2	<=0.5	4	<=0.5	<=2	<=0.03
V	2006-C09-048	Blood	40	4	4	<=0.5	4	<=0.5	<=2	<=0.03
V	2006-C09-050	Urine	75	4	4	1	4	<=0.5	4	0.25
V	2006-C09-188	Urine	1	<=2	>16	16	>16	16	>16	0.5
V	2006-C09-193	Blood	27	<=2	>16	<=0.5	16	<=0.5	>16	0.12
V	2006-C09-203	Blood	74	<=2	4	<=0.5	8	<=0.5	<=2	<=0.03
V	2006-C09-207	Blood	82	<=2	4	<=0.5	4	<=0.5	<=2	<=0.03
V	2006-C09-210	Blood	50	<=2	>16	<=0.5	8	<=0.5	4	0.25
V	2006-C09-212	Blood	71	<=2	>16	<=0.5	8	<=0.5	4	<=0.03
V	2006-C09-218	Blood	34	4	>16	<=0.5	8	<=0.5	<=2	0.12
V	2006-C10-001	Blood	49	4	>16	<=0.5	8	<=0.5	<=2	<=0.03
V	2006-C10-002	Urine	56	<=2	>16	<=0.5	16	<=0.5	8	<=0.03
V	2006-C10-007	Urine	28	<=2	>16	<=0.5	>16	1	>16	<=0.03
V	2006-C10-008	Urine	55	4	>16	<=0.5	8	<=0.5	4	0.25
V	2006-C10-009	Urine	93	<=2	4	<=0.5	4	<=0.5	<=2	<=0.03
V	2006-C10-014	Urine	32	<=2	>16	<=0.5	16	<=0.5	>16	<=0.03
V	2006-C10-027	Urine	54	4	4	<=0.5	4	<=0.5	<=2	<=0.03
V	2006-C10-028	Urine	36	<=2	>16	16	>16	>16	>16	>2
V	2006-C10-032	Urine	79	4	>16	8	>16	16	>16	2
V	2006-C10-033	Blood	74	4	>16	<=0.5	16	<=0.5	16	<=0.03
V	2006-C10-034	Blood	47	4	>16	<=0.5	8	<=0.5	<=2	<=0.03
V	2006-C10-039	Urine	48	<=2	>16	<=0.5	8	<=0.5	4	<=0.03
V	2006-C10-040	Urine	1	<=2	>16	<=0.5	8	<=0.5	4	>2
V	2006-C10-041	Urine	83	<=2	4	<=0.5	4	<=0.5	<=2	<=0.03
V	2006-C10-047	Urine	66	<=2	>16	<=0.5	8	<=0.5	<=2	1
V	2006-C10-048	Urine	35	4	>16	<=0.5	8	<=0.5	4	<=0.03
V	2006-C10-202	Blood	70	<=2	>16	<=0.5	8	<=0.5	4	0.25
V	2006-C10-207	Blood	75	8	<=1	<=0.5	1	<=0.5	<=2	<=0.03
V	2006-C10-208	Blood	58	<=2	>16	<=0.5	8	<=0.5	8	>2
V	2006-C10-213	Blood	71	8	4	<=0.5	4	<=0.5	<=2	<=0.03
V	2006-C10-219	Blood	51	4	>16	<=0.5	8	<=0.5	<=2	0.25
V	2006-E04-003	Urine	37	<=2	>16	<=0.5	8	<=0.5	16	1
V	2006-E04-005	Urine	6	<=2	>16	<=0.5	16	<=0.5	8	<=0.03
V	2006-E04-012	Urine	34	<=2	>16	<=0.5	8	<=0.5	4	>2
V	2006-E04-014	Blood	72	<=2	>16	<=0.5	8	<=0.5	4	0.12

V	2006-E04-020	Other	57	8	>16	<=0.5	>16	<=0.5	16	>2
V	2006-E04-024	Urine	1	<=2	>16	<=0.5	8	<=0.5	<=2	<=0.03
V	2006-E04-029	Other	64	<=2	>16	>16	8	4	>16	<=0.03
V	2006-E04-030	Urine	29	<=2	>16	<=0.5	8	<=0.5	4	<=0.03
V	2006-E04-031	Urine	65	<=2	>16	<=0.5	8	<=0.5	4	0.25
V	2006-E04-032	Urine	7	<=2	>16	<=0.5	16	<=0.5	4	0.06
V	2006-E04-033	Urine	83	<=2	2	<=0.5	4	<=0.5	<=2	0.12
V	2006-E04-035	Urine	61	<=2	>16	<=0.5	>16	<=0.5	8	<=0.03
V	2006-E04-042	Other	62	<=2	8	<=0.5	8	<=0.5	8	0.5
V	2006-E04-047	Urine	23	4	>16	<=0.5	16	<=0.5	4	0.25
V	2006-E04-205	Blood	84	<=2	>16	<=0.5	8	<=0.5	4	0.25
V	2006-E04-207	Blood	70	4	4	<=0.5	4	<=0.5	<=2	<=0.03
V	2006-E04-212	Blood	21	4	>16	<=0.5	8	<=0.5	4	<=0.03
V	2006-E04-213	Blood	17	8	>16	<=0.5	8	<=0.5	4	<=0.03
V	2006-E04-214	Blood	62	<=2	4	<=0.5	8	<=0.5	4	<=0.03
VI	2008-S01-002	Blood	75	<=2	>16	<=0.5	8	<=0.5	4	<=0.03
VI	2008-S01-003	Urine	84	4	>16	4	>16	16	>16	0.5
VI	2008-S01-006	Urine	70	4	>16	<=0.5	8	<=0.5	4	<=0.03
VI	2008-S01-008	Urine	72	<=2	4	>16	2	>16	<=2	0.06
VI	2008-S01-009	Urine	45	<=2	>16	<=0.5	8	<=0.5	4	0.06
VI	2008-S01-023	Urine	2	<=2	4	<=0.5	4	<=0.5	<=2	<=0.03
VI	2008-S01-024	Urine	71	<=2	>16	<=0.5	16	<=0.5	16	1
VI	2008-S01-025	Urine	62	<=2	2	<=0.5	4	<=0.5	<=2	0.25
VI	2008-S01-038	Urine	67	<=2	>16	<=0.5	16	<=0.5	4	>2
VI	2008-S01-046	Urine	73	16	>16	2	8	<=0.5	>16	>2
VI	2008-S01-047	Blood	80	4	4	1	8	<=0.5	<=2	<=0.03
VI	2008-S01-049	Urine	21	<=2	4	<=0.5	4	<=0.5	<=2	0.25
VI	2008-S01-215	Blood	61	<=2	8	<=0.5	8	<=0.5	<=2	<=0.03
VI	2008-S01-223	Blood	72	4	4	<=0.5	4	<=0.5	<=2	<=0.03
VI	2008-S01-230	Blood	49	<=2	>16	<=0.5	8	<=0.5	<=2	<=0.03
VI	2008-S01-242	Blood	34	<=2	>16	<=0.5	8	<=0.5	<=2	0.25
VI	2008-S01-243	Blood	63	4	4	<=0.5	4	<=0.5	<=2	<=0.03
VI	2008-S01-245	Blood	36	<=2	4	<=0.5	4	<=0.5	<=2	<=0.03
VI	2008-S01-250	Blood	53	4	2	<=0.5	8	<=0.5	<=2	<=0.03
VI	2008-C01-005	Urine	12	4	2	<=0.5	4	<=0.5	4	<=0.03
VI	2008-C01-006	Urine	0(10m)	<=2	>16	<=0.5	>16	<=0.5	16	<=0.03
VI	2008-C01-012	Urine	80	<=2	4	<=0.5	4	<=0.5	<=2	<=0.03
VI	2008-C01-023	Urine	28	<=2	>16	4	16	2	>16	>2
VI	2008-C01-026	Urine	41	<=2	4	<=0.5	4	<=0.5	<=2	<=0.03
VI	2008-C01-027	Urine	55	16	>16	2	>16	1	>16	0.25
VI	2008-C01-030	Urine	27	<=2	4	<=0.5	4	<=0.5	<=2	<=0.03
VI	2008-C01-044	Urine	50	<=2	>16	16	>16	4	>16	>2
VI	2008-C01-045	Urine	10	<=2	>16	<=0.5	16	<=0.5	4	0.5
VI	2008-C01-046	Urine	64	4	>16	<=0.5	16	<=0.5	8	0.5
VI	2008-C01-047	Urine	70	<=2	>16	<=0.5	8	<=0.5	<=2	<=0.03
VI	2008-C02-002	Urine	unk	4	4	<=0.5	8	<=0.5	4	<=0.03
VI	2008-C02-003	Urine	ink	8	>16	<=0.5	16	1	>16	>2
VI	2008-C02-015	Urine	unk	4	>16	8	8	8	>16	>2
VI	2008-C02-016	Urine	unk	8	>16	8	>16	16	>16	>2
VI	2008-C02-017	Urine		4	>16	2	>16	8	>16	>2
VI	2008-C02-022	Urine	unk	<=2	>16	2	8	<=0.5	>16	>2
VI	2008-C02-024	Urine	unk	<=2	>16	>16	>16	>16	>16	>2
VI	2008-C02-030	Urine		4	4	<=0.5	4	<=0.5	<=2	<=0.03
VI	2008-C02-031	Urine		<=2	4	<=0.5	8	<=0.5	4	<=0.03
VI	2008-C02-033	Urine		<=2	4	<=0.5	8	<=0.5	<=2	0.12
VI	2008-C02-035	Urine		4	4	<=0.5	4	<=0.5	<=2	0.06
VI	2008-C02-036	Other		<=2	4	<=0.5	4	<=0.5	<=2	<=0.03
VI	2008-C02-040	Urine		<=2	2	<=0.5	4	<=0.5	<=2	0.12
VI	2008-C02-045	Blood		<=2	>16	<=0.5	8	<=0.5	8	<=0.03
VI	2008-C02-205	Blood		<=2	>16	<=0.5	8	<=0.5	<=2	<=0.03
VI	2008-C02-211	Blood		<=2	2	<=0.5	4	<=0.5	<=2	<=0.03

VI	2008-C02-220	Blood		<=2	>16	<=0.5	8	<=0.5	4	<=0.03
VI	2008-C02-222	Blood		<=2	>16	<=0.5	8	<=0.5	<=2	0.12
VI	2008-C02-223	Blood		<=2	8	<=0.5	8	<=0.5	4	<=0.03
VI	2008-C02-225	Blood		<=2	2	<=0.5	2	<=0.5	<=2	<=0.03
VI	2008-C02-226	Blood		<=2	>16	16	>16	>16	>16	>2
VI	2008-C02-227	Blood		<=2	>16	<=0.5	8	<=0.5	<=2	<=0.03
VI	2008-C02-229	Blood		<=2	>16	<=0.5	8	<=0.5	<=2	<=0.03
VI	2008-C02-230	Blood		<=2	>16	16	>16	>16	>16	>2
VI	2008-C02-240	Blood		<=2	4	<=0.5	4	<=0.5	<=2	<=0.03
VI	2008-C04-009	Blood	61	<=2	>16	<=0.5	8	<=0.5	4	<=0.03
VI	2008-C04-013	Urine	0	8	4	<=0.5	8	<=0.5	<=2	<=0.03
VI	2008-C04-017	Urine	74	<=2	>16	<=0.5	16	<=0.5	16	>2
VI	2008-C04-021	Urine	76	4	>16	<=0.5	16	2	>16	>2
VI	2008-C04-023	Blood	78	<=2	>16	<=0.5	8	<=0.5	<=2	0.25
VI	2008-C04-031	Urine	80	4	4	<=0.5	8	<=0.5	<=2	<=0.03
VI	2008-C04-039	Urine	83	4	>16	8	>16	>16	>16	>2
VI	2008-C04-042	Blood	60	>32	>16	<=0.5	16	<=0.5	>16	<=0.03
VI	2008-C04-044	Urine	46	<=2	>16	<=0.5	16	<=0.5	4	0.5
VI	2008-C04-045	Urine	36	<=2	>16	<=0.5	16	<=0.5	4	0.25
VI	2008-C04-225	Blood	75	<=2	>16	<=0.5	16	<=0.5	8	0.25
VI	2008-S05-001	Urine	84	<=2	>16	<=0.5	16	<=0.5	8	>2
VI	2008-S05-003	Blood	90	<=2	<=1	<=0.5	1	<=0.5	<=2	<=0.03
VI	2008-S05-004	Blood	63	<=2	>16	16	>16	>16	>16	>2
VI	2008-S05-006	Urine	71	4	>16	<=0.5	8	<=0.5	4	>2
VI	2008-S05-009	Blood	71	<=2	>16	<=0.5	4	<=0.5	<=2	<=0.03
VI	2008-S05-012	Urine	72	4	>16	<=0.5	16	<=0.5	16	0.25
VI	2008-S05-017	Urine	79	<=2	2	<=0.5	2	<=0.5	4	<=0.03
VI	2008-S05-018	Urine	42	<=2	<=1	<=0.5	1	<=0.5	<=2	<=0.03
VI	2008-S05-023	Urine	8	<=2	>16	<=0.5	16	<=0.5	4	<=0.03
VI	2008-S05-024	Urine	0	8	>16	<=0.5	16	<=0.5	4	2
VI	2008-S05-029	Urine	57	<=2	<=1	<=0.5	1	<=0.5	<=2	<=0.03
VI	2008-S05-031	Urine	21	<=2	>16	<=0.5	8	<=0.5	<=2	<=0.03
VI	2008-S05-032	Urine	68	<=2	>16	<=0.5	8	<=0.5	4	0.25
VI	2008-S05-033	Other	47	<=2	>16	<=0.5	8	<=0.5	4	>2
VI	2008-S05-035	Urine	79	<=2	>16	<=0.5	16	<=0.5	>16	<=0.03
VI	2008-S05-038	Urine	77	4	>16	8	>16	8	>16	0.25
VI	2008-S05-039	Blood	49	<=2	>16	<=0.5	8	<=0.5	4	<=0.03
VI	2008-S05-040	Blood	57	4	4	<=0.5	8	<=0.5	<=2	<=0.03
VI	2008-S05-047	Urine	17	<=2	>16	<=0.5	>16	<=0.5	4	<=0.03
VI	2008-S05-048	Urine	64	<=2	>16	<=0.5	8	<=0.5	<=2	<=0.03
VI	2008-S05-204	Blood	76	4	>16	<=0.5	8	<=0.5	4	>2
VI	2008-S05-205	Blood	86	4	>16	<=0.5	8	<=0.5	<=2	0.25
VI	2008-S05-220	Blood	84	<=2	4	<=0.5	8	<=0.5	4	0.25
VI	2008-S05-225	Blood	84	<=2	>16	1	>16	2	>16	>2
VI	2008-S05-227	Blood	82	<=2	>16	<=0.5	8	<=0.5	<=2	<=0.03
VI	2008-S05-233	Blood	63	<=2	>16	>16	>16	>16	>16	>2
VI	2008-S05-243	Blood	80	<=2	>16	<=0.5	8	<=0.5	4	0.25
VI	2008-S06-003	Urine	62	4	>16	>16	>16	>16	>16	>2
VI	2008-S06-008	Urine	32	<=2	>16	>16	16	8	>16	0.25
VI	2008-S06-012	Urine	56	4	>16	<=0.5	8	<=0.5	4	0.25
VI	2008-S06-013	Urine	69	<=2	>16	<=0.5	>16	<=0.5	>16	0.25
VI	2008-S06-017	Urine	15	<=2	4	<=0.5	4	<=0.5	<=2	<=0.03
VI	2008-S06-019	Urine	80	<=2	<=1	<=0.5	2	<=0.5	<=2	0.12
VI	2008-S06-020	Urine	49	<=2	>16	<=0.5	8	<=0.5	4	<=0.03
VI	2008-S06-023	Urine	74	4	>16	<=0.5	8	<=0.5	<=2	<=0.03
VI	2008-S06-031	Urine	48	<=2	4	<=0.5	4	<=0.5	<=2	<=0.03
VI	2008-S06-040	Urine	73	<=2	>16	<=0.5	16	<=0.5	<=2	0.5
VI	2008-S06-041	Urine	24	<=2	4	<=0.5	4	<=0.5	<=2	<=0.03
VI	2008-S06-042	Urine	64	<=2	2	<=0.5	2	<=0.5	<=2	<=0.03
VI	2008-S06-047	Urine	26	4	>16	<=0.5	16	<=0.5	4	<=0.03
VI	2008-S06-065	Urine	70	4	>16	<=0.5	16	<=0.5	8	<=0.03

VI	2008-S06-107	Other	77	4	>16	1	8	<=0.5	<=2	<=0.03
VI	2008-S06-120	Urine	73	16	>16	8	>16	>16	>16	>2
VI	2008-S06-126	Urine	93	4	>16	<=0.5	8	<=0.5	>16	>2
VI	2008-S06-142	Urine	78	4	4	<=0.5	4	<=0.5	<=2	0.25
VI	2008-S06-143	Urine	87	4	4	<=0.5	4	<=0.5	8	>2
VI	2008-S06-146	Urine	81	4	>16	<=0.5	16	<=0.5	16	<=0.03
VI	2008-S06-159	Urine	69	<=2	>16	<=0.5	8	<=0.5	4	>2
VI	2008-S06-167	Urine	91	<=4	>16	>16	>16	>16	>16	>2
VI	2008-S06-170	Blood	56	<=2	>16	2	8	<=0.5	>16	<=0.03
VI	2008-S06-171	Urine	92	<=2	4	<=0.5	4	<=0.5	<=2	<=0.03
VI	2008-S06-175	Urine	83	<=2	>16	<=0.5	>16	<=0.5	16	>2
VI	2008-S06-177	Blood	83	<=2	4	<=0.5	4	<=0.5	<=2	0.25
VI	2008-S06-180	Urine	91	<=2	>16	<=0.5	8	<=0.5	<=2	0.25
VI	2008-S06-203	Blood	68	<=2	4	<=0.5	4	<=0.5	<=2	<=0.03
VI	2008-S06-204	Other	57	<=2	>16	<=0.5	8	<=0.5	<=2	0.12
VI	2008-S06-214	Blood	85	<=2	>16	<=0.5	8	<=0.5	4	<=0.03
VI	2008-S06-215	Blood	62	16	>16	8	>16	16	>16	>2
VI	2008-S06-216	Blood	82	4	4	<=0.5	4	<=0.5	<=2	>2
VI	2008-S06-217	Blood	62	4	8	<=0.5	4	<=0.5	<=2	<=0.03
VI	2008-S06-221	Blood	68	<=2	>16	8	>16	>16	>16	>2
VI	2008-S06-223	Blood	62	4	>16	<=0.5	16	<=0.5	8	<=0.03
VI	2008-S06-226	Blood	80	4	>16	<=0.5	8	<=0.5	<=2	0.12
VI	2008-S06-227	Blood	76	<=2	>16	<=0.5	16	<=0.5	<=2	<=0.03
VI	2008-S06-229	Blood	50	8	>16	<=0.5	8	1	8	2
VI	2008-S07-001	Urine	8	16	>16	<=0.5	8	<=0.5	4	<=0.03
VI	2008-S07-002	Urine	1	<=2	>16	8	>16	16	>16	>2
VI	2008-S07-004	Urine	70	4	16	<=0.5	8	<=0.5	4	0.06
VI	2008-S07-007	Urine	29	<=2	>16	<=0.5	16	<=0.5	4	0.25
VI	2008-S07-009	Urine	80	<=2	>16	16	>16	>16	>16	>2
VI	2008-S07-011	Urine	31	<=2	>16	<=0.5	8	<=0.5	4	<=0.03
VI	2008-S07-019	Urine	3	<=2	>16	<=0.5	8	<=0.5	<=2	<=0.03
VI	2008-S07-024	Urine	74	16	>16	>16	>16	>16	>16	>2
VI	2008-S07-025	Urine	59	<=2	>16	<=0.5	8	<=0.5	4	<=0.03
VI	2008-S07-026	Urine	40	16	>16	>16	>16	>16	>16	>2
VI	2008-S07-034	Urine	70	<=2	>16	8	16	2	>16	>2
VI	2008-S07-043	Other	56	<=2	>16	>16	8	4	>16	>2
VI	2008-S07-044	Urine	89	<=2	>16	<=0.5	8	<=0.5	4	0.25
VI	2008-S07-202	Other	70	4	>16	<=0.5	16	<=0.5	8	>2
VI	2008-S07-215	Blood	62	4	>16	<=0.5	8	<=0.5	<=2	<=0.03
VI	2008-S07-222	Blood	18	4	>16	<=0.5	16	<=0.5	<=2	0.25
VI	2008-S07-224	Blood	82	<=2	>16	<=0.5	8	<=0.5	<=2	<=0.03
VI	2008-S07-232	Blood	74	<=2	4	<=0.5	8	<=0.5	<=2	<=0.03
VI	2008-S07-239	Blood	53	<=2	>16	8	>16	16	>16	0.25
VI	2008-S07-247	Blood	70	<=2	4	<=0.5	4	<=0.5	<=2	0.06
VI	2008-S07-248	Blood	84	4	>16	<=0.5	16	<=0.5	8	>2
VI	2008-N05-001	Urine	31	4	>16	<=0.5	16	<=0.5	<=2	<=0.03
VI	2008-N05-004	Urine	72	4	8	<=0.5	8	<=0.5	<=2	<=0.03
VI	2008-N05-008	Urine	26	<=2	>16	<=0.5	16	<=0.5	4	<=0.03
VI	2008-N05-010	Urine	77	<=2	>16	<=0.5	16	<=0.5	16	>2
VI	2008-N05-015	Urine	6	<=2	8	<=0.5	8	<=0.5	<=2	<=0.03
VI	2008-N05-016	Urine	54	<=2	>16	2	>16	8	>16	0.06
VI	2008-N05-017	Urine	30	4	>16	<=0.5	8	<=0.5	<=2	<=0.03
VI	2008-N05-018	Urine	82	<=2	>16	<=0.5	16	<=0.5	8	<=0.03
VI	2008-N05-019	Urine	34	<=2	>16	<=0.5	8	<=0.5	<=2	<=0.03
VI	2008-N05-020	Urine	31	<=2	8	<=0.5	8	<=0.5	<=2	<=0.03
VI	2008-N05-021	Urine	27	4	>16	<=0.5	>16	<=0.5	16	<=0.03
VI	2008-N05-022	Urine	63	<=2	4	<=0.5	4	<=0.5	<=2	0.06
VI	2008-N05-027	Urine	83	<=2	2	<=0.5	4	<=0.5	<=2	<=0.03
VI	2008-N05-029	Urine	67	4	>16	<=0.5	16	<=0.5	4	>2
VI	2008-N05-031	Urine	39	<=2	4	<=0.5	4	<=0.5	<=2	<=0.03
VI	2008-N05-032	Urine	47	<=2	>16	<=0.5	8	<=0.5	<=2	0.12

VI	2008-N05-037	Urine	53	<=2	4	<=0.5	4	<=0.5	<=2	<=0.03
VI	2008-N05-050	Urine	53	4	8	<=0.5	8	<=0.5	<=2	>2
VI	2008-N05-212	Blood	46	<=2	4	<=0.5	4	<=0.5	<=2	0.25
VI	2008-N05-223	Blood	64	<=2	>16	<=0.5	8	<=0.5	<=2	<=0.03
VI	2008-N05-230	Blood	38	<=2	>16	<=0.5	16	<=0.5	16	0.25
VI	2008-N06-001	Urine	81	<=2	>16	<=0.5	8	<=0.5	<=2	>2
VI	2008-N06-004	Urine	48	4	>16	<=0.5	16	<=0.5	8	0.25
VI	2008-N06-006	Urine	76	4	>16	<=0.5	4	<=0.5	<=2	<=0.03
VI	2008-N06-007	Urine	37	4	>16	<=0.5	8	<=0.5	<=2	<=0.03
VI	2008-N06-010	Urine	78	8	>16	<=0.5	8	<=0.5	4	0.12
VI	2008-N06-018	Urine	30	4	>16	<=0.5	8	<=0.5	4	<=0.03
VI	2008-N06-020	Urine	82	<=2	>16	4	16	2	>16	>2
VI	2008-N06-021	Urine	49	<=2	4	<=0.5	4	<=0.5	<=2	0.25
VI	2008-N06-022	Urine	77	4	>16	<=0.5	8	<=0.5	4	>2
VI	2008-N06-023	Urine	39	4	>16	<=0.5	8	<=0.5	4	>2
VI	2008-N06-031	Blood	91	<=2	4	<=0.5	8	<=0.5	<=2	<=0.03
VI	2008-N06-032	Blood	65	<=2	>16	<=0.5	8	<=0.5	<=2	<=0.03
VI	2008-N06-043	Urine	85	4	4	<=0.5	4	<=0.5	<=2	<=0.03
VI	2008-N06-044	Urine	37	<=2	<=1	<=0.5	1	<=0.5	<=2	<=0.03
VI	2008-N06-045	Urine	28	<=2	>16	<=0.5	8	<=0.5	4	0.12
VI	2008-N06-201	Blood	57	<=2	4	<=0.5	4	<=0.5	<=2	<=0.03
VI	2008-N06-205	Blood	80	<=2	4	<=0.5	4	<=0.5	<=2	0.25
VI	2008-N06-216	Blood	55	8	>16	>16	16	>16	>16	>2
VI	2008-N06-217	Blood	65	<=2	4	<=0.5	4	<=0.5	<=2	<=0.03
VI	2008-N06-218	Blood	89	4	>16	<=0.5	8	<=0.5	4	0.12
VI	2008-N06-226	Other	76	<=2	8	<=0.5	8	<=0.5	16	0.12
VI	2008-N06-231	Blood	82	<=2	4	<=0.5	4	<=0.5	<=2	0.5
VI	2008-N06-234	Blood	69	<=2	4	<=0.5	4	<=0.5	<=2	0.25
VI	2008-N06-236	Blood	76	4	>16	<=0.5	8	<=0.5	<=2	0.12
VI	2008-N06-237	Blood	53	<=2	2	<=0.5	2	<=0.5	<=2	<=0.03
VI	2008-N06-249	Blood	47	4	>16	<=0.5	8	<=0.5	8	0.25
VI	2008-N07-001	Blood	64	<=2	>16	<=0.5	8	<=0.5	<=2	<=0.03
VI	2008-N07-002	Urine	44	<=2	>16	<=0.5	8	<=0.5	<=2	0.12
VI	2008-N07-007	Other	42	<=2	>16	8	>16	16	>16	0.5
VI	2008-N07-010	Blood	58	<=2	>16	<=0.5	8	<=0.5	<=2	0.25
VI	2008-N07-011	Urine	57	4	>16	<=0.5	8	<=0.5	<=2	<=0.03
VI	2008-N07-013	Blood	82	<=2	>16	<=0.5	16	<=0.5	4	1
VI	2008-N07-023	Urine	83	>32	>16	>16	>16	>16	>16	>2
VI	2008-N07-025	Urine	49	4	>16	<=0.5	8	<=0.5	8	0.25
VI	2008-N07-026	Blood	75	<=2	>16	<=0.5	8	<=0.5	4	>2
VI	2008-N07-027	Urine	36	<=2	>16	<=0.5	16	<=0.5	<=2	<=0.03
VI	2008-N07-029	Urine	30	<=2	>16	<=0.5	8	<=0.5	<=2	1
VI	2008-N07-031	Urine	83	<=2	>16	<=0.5	8	<=0.5	4	0.25
VI	2008-N07-032	Urine	20	4	4	<=0.5	4	<=0.5	<=2	<=0.03
VI	2008-N07-033	Urine	80	<=2	>16	>16	>16	>16	>16	1
VI	2008-N07-035	Urine	29	4	>16	<=0.5	16	<=0.5	8	>2
VI	2008-N07-042	Urine	49	<=2	>16	8	>16	8	>16	>2
VI	2008-N07-044	Urine	54	4	>16	<=0.5	8	<=0.5	4	<=0.03
VI	2008-N07-206	Blood	76	<=2	>16	4	>16	16	>16	<=0.03
VI	2008-N07-212	Blood	91	8	>16	<=0.5	16	<=0.5	8	1
VI	2008-N07-216	Blood	26	<=2	>16	<=0.5	8	<=0.5	<=2	<=0.03
VI	2008-N07-219	Blood	85	4	>16	>16	>16	4	>16	0.25
VI	2008-N07-228	Blood	70	<=2	>16	<=0.5	4	<=0.5	<=2	0.25
VI	2008-N07-231	Blood	24	8	>16	<=0.5	8	<=0.5	<=2	0.25
VI	2008-N07-237	Blood	60	4	>16	<=0.5	8	<=0.5	<=2	<=0.03
VI	2008-N07-242	Blood	72	4	4	<=0.5	8	<=0.5	<=2	<=0.03
VI	2008-N07-244	Blood	65	<=2	4	<=0.5	8	<=0.5	<=2	<=0.03
VI	2008-N07-250	Blood	78	<=2	>16	<=0.5	8	<=0.5	4	<=0.03
VI	2008-S08-001	Urine	48	4	4	<=0.5	4	<=0.5	<=2	0.25
VI	2008-S08-002	Urine	51	<=2	>16	<=0.5	8	<=0.5	<=2	<=0.03
VI	2008-S08-006	Urine	46	<=2	>16	<=0.5	16	<=0.5	16	<=0.03

VI	2008-S08-007	Urine	21	<=2	>16	<=0.5	16	<=0.5	4	<=0.03
VI	2008-S08-010	Urine	93	<=2	>16	<=0.5	16	<=0.5	4	<=0.03
VI	2008-S08-011	Urine	80	<=2	>16	>16	>16	>16	>16	>2
VI	2008-S08-015	Urine	53	<=2	>16	<=0.5	8	<=0.5	4	0.25
VI	2008-S08-019	Urine	19	4	4	<=0.5	4	<=0.5	4	<=0.03
VI	2008-S08-020	Urine	49	<=2	>16	<=0.5	>16	2	>16	0.25
VI	2008-S08-023	Urine	72	4	4	<=0.5	4	<=0.5	<=2	<=0.03
VI	2008-S08-024	Urine	80	<=2	>16	<=0.5	8	<=0.5	8	<=0.03
VI	2008-S08-027	Urine	43	<=2	>16	<=0.5	16	<=0.5	8	0.25
VI	2008-S08-029	Urine	30	<=2	>16	<=0.5	16	<=0.5	4	0.12
VI	2008-S08-033	Urine	81	<=2	>16	<=0.5	8	<=0.5	4	>2
VI	2008-S08-035	Urine	70	8	>16	<=0.5	8	<=0.5	4	>2
VI	2008-S08-036	Urine	82	>32	>16	>16	8	2	>16	0.12
VI	2008-S08-037	Urine	62	<=2	>16	>16	>16	>16	>16	>2
VI	2008-S08-039	Urine	39	<=2	>16	<=0.5	8	<=0.5	4	<=0.03
VI	2008-S08-042	Urine	68	<=2	>16	>16	>16	>16	>16	>2
VI	2008-S08-043	Urine	77	<=2	>16	16	>16	>16	>16	<=0.03
VI	2008-S08-044	Urine	86	<=2	4	<=0.5	4	<=0.5	<=2	0.06
VI	2008-S08-050	Urine	85	<=2	4	<=0.5	8	<=0.5	<=2	<=0.03
VI	2008-S08-076	Urine	62	<=2	>16	<=0.5	8	<=0.5	<=2	0.25
VI	2008-S08-079	Urine	46	<=2	>16	>16	16	>16	>16	>2
VI	2008-S08-123	Urine	64	<=2	>16	8	>16	16	>16	>2
VI	2008-S08-181	Urine	8	4	>16	<=0.5	16	<=0.5	8	0.25
VI	2008-E02-004	Urine	22	<=2	4	<=0.5	4	<=0.5	<=2	<=0.03
VI	2008-E02-009	Urine	71	<=2	4	<=0.5	4	<=0.5	<=2	<=0.03
VI	2008-E02-016	Urine	69	<=2	>16	<=0.5	8	<=0.5	4	<=0.03
VI	2008-E02-018	Blood	42	8	>16	<=0.5	8	<=0.5	4	<=0.03
VI	2008-E02-019	Blood	68	4	4	<=0.5	4	<=0.5	<=2	0.25
VI	2008-E02-021	Urine	82	<=2	>16	<=0.5	16	<=0.5	4	>2
VI	2008-E02-022	Urine	76	<=2	>16	<=0.5	8	<=0.5	4	<=0.03
VI	2008-E02-025	Urine	62	4	>16	<=0.5	16	<=0.5	<=2	0.25
VI	2008-E02-026	Urine	93	<=2	4	<=0.5	4	<=0.5	<=2	<=0.03
VI	2008-E02-030	Blood	54	8	>16	<=0.5	16	<=0.5	8	0.12
VI	2008-E02-031	Urine	30	<=2	2	<=0.5	4	<=0.5	<=2	<=0.03
VI	2008-E02-033	Urine	25	<=2	>16	<=0.5	8	<=0.5	<=2	0.12
VI	2008-E02-038	Urine	83	<=2	>16	8	16	2	>16	<=0.03
VI	2008-E02-039	Urine	72	<=2	>16	<=0.5	16	<=0.5	4	<=0.03
VI	2008-E02-043	Urine	33	4	4	<=0.5	8	<=0.5	<=2	<=0.03
VI	2008-E02-044	Urine	90	<=2	>16	<=0.5	8	<=0.5	<=2	<=0.03
VI	2008-E02-045	Urine	76	<=2	2	<=0.5	4	<=0.5	<=2	<=0.03
VI	2008-E02-203	Other	30	4	2	<=0.5	2	<=0.5	<=2	<=0.03
VI	2008-E02-208	Blood	58	<=2	>16	<=0.5	16	<=0.5	<=2	0.5
VI	2008-E02-209	Blood	57	<=2	>16	<=0.5	16	<=0.5	4	<=0.03
VI	2008-E02-210	Blood	72	<=2	8	<=0.5	4	<=0.5	<=2	0.25
VI	2008-N08-002	Urine	19	<=2	>16	<=0.5	8	<=0.5	<=2	0.5
VI	2008-N08-005	Urine	76	<=2	>16	<=0.5	8	<=0.5	4	1
VI	2008-N08-007	Urine	74	<=2	>16	<=0.5	>16	<=0.5	>16	0.25
VI	2008-N08-008	Urine	61	<=2	>16	<=0.5	8	<=0.5	<=2	<=0.03
VI	2008-N08-014	Urine	87	<=2	>16	>16	>16	>16	>16	0.25
VI	2008-N08-015	Urine	0	<=2	>16	<=0.5	8	<=0.5	<=2	0.25
VI	2008-N08-018	Urine	83	<=2	2	<=0.5	4	<=0.5	<=2	0.25
VI	2008-N08-023	Urine	42	8	>16	<=0.5	8	<=0.5	4	0.12
VI	2008-N08-027	Urine	36	<=2	2	<=0.5	4	<=0.5	<=2	<=0.03
VI	2008-N08-028	Urine	40	<=2	>16	<=0.5	8	<=0.5	4	<=0.03
VI	2008-N08-029	Urine	49	<=2	4	<=0.5	4	<=0.5	<=2	0.25
VI	2008-N08-030	Urine	58	4	>16	<=0.5	16	<=0.5	4	0.12
VI	2008-N08-032	Urine	54	<=2	4	<=0.5	4	<=0.5	<=2	<=0.03
VI	2008-N08-038	Blood	76	4	>16	<=0.5	16	<=0.5	8	<=0.03
VI	2008-N08-039	Blood	40	<=2	2	<=0.5	4	<=0.5	<=2	<=0.03
VI	2008-N08-041	Blood	74	<=2	>16	<=0.5	8	<=0.5	4	>2
VI	2008-N08-044	Urine	45	<=2	>16	4	>16	8	>16	<=0.03

VI	2008-N08-047	Urine	0	<=2	>16	<=0.5	8	<=0.5	4	<=0.03
VI	2008-N08-211	Blood	74	<=2	2	<=0.5	4	<=0.5	<=2	0.25
VI	2008-N08-219	Blood	0	<=2	>16	<=0.5	16	<=0.5	8	0.25
VI	2008-S09-009	Urine	68	4	>16	<=0.5	4	<=0.5	>16	0.06
VI	2008-S09-012	Urine	20	4	>16	<=0.5	8	<=0.5	<=2	<=0.03
VI	2008-S09-013	Urine	21	<=2	>16	<=0.5	8	<=0.5	<=2	<=0.03
VI	2008-S09-014	Urine	55	<=2	>16	<=0.5	8	<=0.5	<=2	<=0.03
VI	2008-S09-019	Blood	69	4	<=1	<=0.5	2	<=0.5	<=2	0.12
VI	2008-S09-023	Blood	58	4	>16	<=0.5	16	<=0.5	8	<=0.03
VI	2008-S09-025	Blood	73	8	>16	<=0.5	8	<=0.5	8	>2
VI	2008-S09-027	Urine	52	<=2	4	<=0.5	4	<=0.5	<=2	<=0.03
VI	2008-S09-029	Urine	41	<=2	>16	<=0.5	16	<=0.5	4	<=0.03
VI	2008-S09-030	Blood	58	<=2	>16	<=0.5	8	<=0.5	<=2	0.12
VI	2008-S09-035	Urine	71	4	4	<=0.5	4	<=0.5	<=2	<=0.03
VI	2008-S09-036	Urine	80	4	2	<=0.5	4	<=0.5	4	<=0.03
VI	2008-S09-037	Urine	79	8	>16	<=0.5	8	<=0.5	<=2	<=0.03
VI	2008-S09-038	Blood	76	<=2	>16	<=0.5	8	<=0.5	<=2	0.25
VI	2008-S09-043	Urine	61	<=2	>16	<=0.5	8	<=0.5	<=2	0.25
VI	2008-S09-044	Urine	33	<=2	>16	<=0.5	8	<=0.5	<=2	0.25
VI	2008-S09-046	Blood	78	<=2	>16	>16	>16	>16	>16	>2
VI	2008-S09-047	Urine	78	<=2	2	<=0.5	4	<=0.5	<=2	<=0.03
VI	2008-S09-050	Urine	67	<=2	>16	<=0.5	8	<=0.5	4	>2
VI	2008-S09-186	Urine	5	4	>16	<=0.5	8	<=0.5	<=2	0.25
VI	2008-S09-204	Blood	78	<=2	4	<=0.5	4	<=0.5	<=2	<=0.03
VI	2008-S09-205	Blood	81	<=2	4	<=0.5	4	<=0.5	<=2	<=0.03
VI	2008-S09-209	Blood	73	<=2	>16	<=0.5	8	<=0.5	<=2	<=0.03
VI	2008-S09-210	Blood	76	<=2	>16	<=0.5	8	<=0.5	<=2	0.06
VI	2008-S09-214	Blood	49	<=2	>16	<=0.5	8	<=0.5	<=2	<=0.03
VI	2008-S09-216	Blood	83	8	>16	>16	>16	>16	>16	>2
VI	2008-S09-218	Blood	51	<=2	4	<=0.5	8	<=0.5	<=2	<=0.03
VI	2008-S09-223	Blood	43	<=2	>16	<=0.5	8	<=0.5	4	>2
VI	2008-S09-224	Blood	64	<=2	>16	<=0.5	16	<=0.5	4	0.25
VI	2008-S09-225	Blood	66	4	>16	<=0.5	8	<=0.5	8	>2
VI	2008-S09-230	Blood	57	4	>16	<=0.5	8	<=0.5	<=2	<=0.03
VI	2008-S09-235	Blood	75	4	>16	>16	>16	>16	>16	>2
VI	2008-S09-240	Blood	58	<=2	>16	<=0.5	8	<=0.5	<=2	1
VI	2008-S09-241	Blood	85	<=2	>16	<=0.5	8	<=0.5	<=2	<=0.03
VI	2008-S09-242	Blood	53	4	4	<=0.5	4	<=0.5	<=2	<=0.03
VI	2008-S09-246	Blood	76	8	>16	>16	16	16	>16	>2
VI	2008-E03-003	Urine	89	<=2	>16	<=0.5	8	<=0.5	<=2	0.25
VI	2008-E03-004	Urine	88	8	>16	<=0.5	16	1	16	>2
VI	2008-E03-008	Urine	98	<=2	>16	<=0.5	16	<=0.5	8	0.06
VI	2008-E03-011	Urine	77	<=2	>16	<=0.5	16	<=0.5	4	<=0.03
VI	2008-E03-013	Urine	98	<=2	>16	<=0.5	8	<=0.5	4	>2
VI	2008-E03-016	Urine	46	<=2	>16	<=0.5	16	<=0.5	4	<=0.03
VI	2008-E03-017	Urine	88	4	4	<=0.5	4	<=0.5	<=2	<=0.03
VI	2008-E03-019	Urine	67	8	>16	<=0.5	8	<=0.5	4	<=0.03
VI	2008-E03-030	Blood	54	<=2	>16	<=0.5	8	<=0.5	<=2	<=0.03
VI	2008-E03-031	Blood	59	<=2	4	<=0.5	8	<=0.5	4	<=0.03
VI	2008-E03-033	Urine	41	<=2	>16	<=0.5	8	<=0.5	8	<=0.03
VI	2008-E03-034	Urine	84	4	>16	<=0.5	16	<=0.5	8	0.25
VI	2008-E03-039	Urine	86	4	>16	<=0.5	8	<=0.5	<=2	1
VI	2008-E03-041	Urine	81	<=2	4	<=0.5	4	<=0.5	<=2	<=0.03
VI	2008-E03-042	Urine	77	<=2	2	<=0.5	4	<=0.5	<=2	<=0.03
VI	2008-E03-045	Urine	69	<=2	>16	<=0.5	16	<=0.5	8	>2
VI	2008-E03-048	Urine	93	4	>16	<=0.5	8	<=0.5	4	>2
VI	2008-E03-049	Urine	49	<=2	>16	<=0.5	8	<=0.5	<=2	<=0.03
VI	2008-E03-231	Blood	78	<=2	>16	8	>16	16	>16	>2
VI	2008-E03-235	Blood	84	<=2	>16	<=0.5	8	<=0.5	4	0.25
VI	2008-C06-002	Blood	82	4	>16	<=0.5	8	<=0.5	<=2	<=0.03
VI	2008-C06-006	Urine	46	4	>16	<=0.5	16	<=0.5	>16	<=0.03

VI	2008-C06-017	Urine	70	16	>16	16	16	4	>16	>2
VI	2008-C06-018	Urine	25	<=2	>16	>16	>16	>16	>16	>2
VI	2008-C06-025	Urine	60	4	>16	<=0.5	16	<=0.5	>16	>2
VI	2008-C06-027	Urine	27	<=2	>16	<=0.5	8	<=0.5	4	0.25
VI	2008-C06-032	Urine	66	<=2	>16	<=0.5	8	<=0.5	<=2	>2
VI	2008-C06-034	Blood	64	4	>16	<=0.5	8	<=0.5	<=2	<=0.03
VI	2008-C06-039	Urine	33	<=2	>16	<=0.5	4	<=0.5	<=2	<=0.03
VI	2008-C06-042	Urine	52	<=2	2	<=0.5	4	<=0.5	<=2	<=0.03
VI	2008-C06-043	Urine	80	<=2	>16	<=0.5	8	<=0.5	<=2	0.25
VI	2008-C06-045	Urine	25	<=2	4	<=0.5	4	<=0.5	<=2	<=0.03
VI	2008-C06-199	Urine	2	<=2	>16	<=0.5	8	<=0.5	4	0.5
VI	2008-C06-211	Blood	73	<=2	>16	<=0.5	16	<=0.5	8	<=0.03
VI	2008-C07-001	Blood	42	4	>16	8	8	4	>16	>2
VI	2008-C07-003	Blood	81	4	>16	<=0.5	>16	2	>16	<=0.03
VI	2008-C07-005	Blood	81	<=2	2	<=0.5	4	<=0.5	<=2	<=0.03
VI	2008-C07-007	Blood	45	<=2	>16	<=0.5	16	<=0.5	8	0.25
VI	2008-C07-008	Blood	93	8	>16	<=0.5	8	<=0.5	>16	>2
VI	2008-C07-009	Blood	74	8	>16	<=0.5	8	<=0.5	8	>2
VI	2008-C07-011	Blood	70	<=2	4	<=0.5	8	<=0.5	<=2	>2
VI	2008-C07-012	Blood	29	4	>16	<=0.5	16	<=0.5	8	0.25
VI	2008-C07-013	Blood	83	8	2	<=0.5	4	<=0.5	<=2	0.12
VI	2008-C07-014	Blood	66	4	>16	4	>16	16	>16	>2
VI	2008-C07-015	Blood	98	<=2	>16	<=0.5	8	<=0.5	<=2	<=0.03
VI	2008-C07-016	Blood	76	<=2	>16	16	>16	>16	>16	>2
VI	2008-C07-017	Blood	61	<=2	4	<=0.5	4	<=0.5	<=2	<=0.03
VI	2008-C07-018	Blood	67	4	>16	<=0.5	4	<=0.5	<=2	1
VI	2008-C07-020	Blood	77	<=2	>16	1	>16	1	8	<=0.03
VI	2008-C07-021	Blood	44	4	>16	<=0.5	8	<=0.5	<=2	0.25
VI	2008-C07-022	Blood	53	<=2	4	<=0.5	4	<=0.5	<=2	<=0.03
VI	2008-C07-024	Blood	87	4	>16	<=0.5	16	<=0.5	8	0.12
VI	2008-C07-026	Blood	73	<=2	4	<=0.5	2	<=0.5	<=2	<=0.03
VI	2008-C07-028	Blood	82	4	2	<=0.5	2	<=0.5	<=2	<=0.03
VI	2008-C07-029	Blood	72	<=2	>16	<=0.5	8	<=0.5	4	<=0.03
VI	2008-C07-035	Blood	71	<=2	>16	16	>16	>16	>16	>2
VI	2008-C07-044	Blood	86	<=2	4	<=0.5	4	<=0.5	<=2	<=0.03
VI	2008-C07-049	Blood	78	<=2	>16	16	>16	>16	>16	>2
VI	2008-C07-205	Blood	64	<=2	>16	<=0.5	8	<=0.5	<=2	<=0.03
VI	2008-C07-209	Blood	75	4	<=1	<=0.5	2	<=0.5	<=2	<=0.03
VI	2008-C07-214	Blood	88	<=2	>16	8	>16	8	>16	>2
VI	2008-C07-216	Blood	57	4	>16	<=0.5	8	<=0.5	<=2	<=0.03
VI	2008-C07-218	Blood	71	4	2	<=0.5	4	<=0.5	<=2	0.06
VI	2008-C07-234	Blood	81	<=2	4	<=0.5	4	<=0.5	<=2	0.06
VI	2008-C07-237	Blood	74	<=2	2	<=0.5	4	<=0.5	<=2	<=0.03
VI	2008-C07-240	Blood	58	<=2	>16	<=0.5	8	<=0.5	4	0.25
VI	2008-C07-242	Blood	75	4	>16	<=0.5	8	<=0.5	4	0.25
VI	2008-C07-247	Blood	44	<=2	>16	<=0.5	8	<=0.5	<=2	0.25
VI	2008-C07-248	Blood	76	<=2	>16	<=0.5	8	<=0.5	4	0.25
VI	2008-N16-001	Urine	23	<=2	4	<=0.5	4	1	<=2	<=0.03
VI	2008-N16-002	Urine	20	<=2	>16	<=0.5	4	<=0.5	4	<=0.03
VI	2008-N16-003	Blood	40	<=2	2	<=0.5	4	<=0.5	<=2	<=0.03
VI	2008-N16-006	Urine	23	<=2	>16	<=0.5	8	<=0.5	4	0.12
VI	2008-N16-020	Urine	1	<=2	>16	>16	8	16	>16	>2
VI	2008-N16-023	Urine	3	<=2	>16	<=0.5	16	<=0.5	>16	<=0.03
VI	2008-N16-025	Urine	86	<=2	>16	<=0.5	16	<=0.5	16	<=0.03
VI	2008-N16-028	Urine	86	16	>16	<=0.5	>16	4	>16	>2
VI	2008-N16-033	Urine	83	8	>16	>16	>16	>16	>16	>2
VI	2008-N16-034	Urine	23	<=2	4	<=0.5	4	<=0.5	<=2	<=0.03
VI	2008-N16-047	Urine	54	<=2	<=1	<=0.5	2	<=0.5	<=2	<=0.03
VI	2008-N16-048	Urine	67	<=2	4	<=0.5	4	<=0.5	<=2	<=0.03
VI	2008-N16-049	Urine	92	<=2	>16	<=0.5	16	<=0.5	16	>2
VI	2008-N16-050	Urine	74	<=2	8	<=0.5	4	<=0.5	8	>2

VI	2008-N16-211	Blood	87	<=2	>16	<=0.5	16	<=0.5	16	>2
VI	2008-N16-218	Blood	82	4	>16	<=0.5	8	<=0.5	4	>2
VI	2008-N16-220	Blood	77	<=2	>16	<=0.5	8	<=0.5	<=2	<=0.03
VI	2008-N16-221	Blood	83	<=2	4	<=0.5	4	<=0.5	<=2	<=0.03
VI	2008-N16-232	Blood	81	4	4	<=0.5	8	<=0.5	4	0.12
VI	2008-N16-249	Blood	80	<=2	>16	<=0.5	4	<=0.5	<=2	<=0.03
VI	2008-N17-001	Urine	47	4	>16	<=0.5	16	<=0.5	<=2	0.06
VI	2008-N17-003	Urine	78	<=2	>16	<=0.5	16	<=0.5	8	<=0.03
VI	2008-N17-004	Urine	75	4	8	<=0.5	8	<=0.5	<=2	0.12
VI	2008-N17-017	Urine	94	4	>16	8	>16	8	>16	<=0.03
VI	2008-N17-018	Urine	86	8	>16	>16	16	16	>16	>2
VI	2008-N17-022	Urine	24	<=2	4	<=0.5	4	<=0.5	<=2	<=0.03
VI	2008-N17-023	Urine	28	<=2	>16	<=0.5	8	<=0.5	4	<=0.03
VI	2008-N17-024	Urine	71	4	>16	<=0.5	16	<=0.5	16	<=0.03
VI	2008-N17-025	Urine	26	<=2	>16	<=0.5	8	<=0.5	<=2	<=0.03
VI	2008-N17-026	Urine	69	<=2	>16	16	>16	>16	>16	>2
VI	2008-N17-027	Urine	51	<=2	4	<=0.5	4	<=0.5	<=2	<=0.03
VI	2008-N17-028	Blood	65	4	>16	<=0.5	8	<=0.5	<=2	<=0.03
VI	2008-N17-031	Urine	40	<=2	>16	<=0.5	16	<=0.5	16	<=0.03
VI	2008-N17-032	Blood	71	<=2	2	<=0.5	4	<=0.5	<=2	<=0.03
VI	2008-N17-033	Urine	75	<=2	4	<=0.5	4	<=0.5	<=2	<=0.03
VI	2008-N17-034	Urine	53	<=2	2	<=0.5	4	<=0.5	<=2	<=0.03
VI	2008-N17-035	Urine	64	<=2	2	<=0.5	4	<=0.5	<=2	0.25
VI	2008-N17-036	Urine	52	4	>16	<=0.5	8	<=0.5	4	<=0.03
VI	2008-N17-038	Blood	84	<=2	4	<=0.5	4	<=0.5	<=2	<=0.03
VI	2008-N17-039	Blood	73	<=2	2	<=0.5	4	<=0.5	<=2	<=0.03
VI	2008-N17-042	Urine	44	4	>16	8	>16	16	>16	0.25
VI	2008-N17-049	Urine	43	4	8	<=0.5	8	<=0.5	4	>2
VI	2008-N17-050	Blood	93	<=2	>16	4	16	4	>16	>2
VI	2008-N17-165	Blood	72	8	>16	<=0.5	16	<=0.5	8	0.25
VI	2008-N17-181	Urine	69	4	>16	<=0.5	8	<=0.5	<=2	0.06
VI	2008-N17-184	Urine	4	<=2	>16	16	>16	>16	>16	>2
VI	2008-N17-185	Urine	5	<=2	2	<=0.5	4	<=0.5	<=2	<=0.03
VI	2008-N19-002	Blood	74	<=2	>16	<=0.5	8	<=0.5	<=2	0.5
VI	2008-N19-003	Urine	27	<=2	>16	<=0.5	16	<=0.5	8	<=0.03
VI	2008-N19-004	Urine	24	<=2	>16	<=0.5	8	<=0.5	<=2	<=0.03
VI	2008-N19-007	Urine	68	<=2	>16	<=0.5	16	<=0.5	8	<=0.03
VI	2008-N19-011	Urine	92	<=2	>16	8	>16	16	>16	>2
VI	2008-N19-012	Urine	36	<=2	>16	<=0.5	16	<=0.5	16	<=0.03
VI	2008-N19-013	Urine	48	4	>16	<=0.5	16	<=0.5	16	0.25
VI	2008-N19-015	Urine	69	4	>16	<=0.5	8	<=0.5	8	<=0.03
VI	2008-N19-019	Urine	70	4	>16	16	>16	>16	>16	>2
VI	2008-N19-024	Blood	51	<=2	>16	<=0.5	16	<=0.5	<=2	<=0.03
VI	2008-N19-028	Urine	76	4	>16	2	>16	8	>16	>2
VI	2008-N19-029	Urine	87	<=2	4	<=0.5	4	<=0.5	<=2	<=0.03
VI	2008-N19-030	Urine	82	<=2	4	<=0.5	4	<=0.5	<=2	<=0.03
VI	2008-N19-031	Urine	94	<=2	>16	<=0.5	16	<=0.5	8	<=0.03
VI	2008-N19-034	Urine	54	<=2	2	<=0.5	4	<=0.5	<=2	0.12
VI	2008-N19-041	Urine	20	<=2	>16	<=0.5	8	<=0.5	<=2	<=0.03
VI	2008-N19-042	Blood	77	<=2	2	<=0.5	2	<=0.5	<=2	<=0.03
VI	2008-N19-043	Urine	31	<=2	<=1	<=0.5	1	<=0.5	<=2	<=0.03
VI	2008-N19-048	Urine	23	<=2	>16	<=0.5	8	<=0.5	<=2	<=0.03
VI	2008-N19-204	Blood	83	<=2	>16	<=0.5	16	<=0.5	16	0.5
VI	2008-N19-205	Blood	60	4	2	<=0.5	4	<=0.5	<=2	0.12
VI	2008-N19-206	Blood	38	<=2	4	<=0.5	4	<=0.5	<=2	<=0.03
VI	2008-N19-214	Blood	71	<=2	>16	<=0.5	8	<=0.5	<=2	>2
VI	2008-N19-217	Blood	77	<=2	>16	<=0.5	8	<=0.5	<=2	<=0.03
VI	2008-N19-223	Blood	86	<=2	>16	<=0.5	16	<=0.5	16	<=0.03
VI	2008-N19-233	Blood	83	<=2	>16	<=0.5	8	<=0.5	<=2	<=0.03
VI	2008-C08-002	Urine	72	<=2	2	<=0.5	4	<=0.5	<=2	<=0.03
VI	2008-C08-005	Urine	78	4	4	<=0.5	4	<=0.5	<=2	<=0.03

VI	2008-C08-006	Urine	74	<=2	>16	2	16	<=0.5	>16	>2
VI	2008-C08-010	Urine	0	4	>16	<=0.5	8	<=0.5	<=2	<=0.03
VI	2008-C08-013	Urine	10	4	8	<=0.5	4	<=0.5	<=2	<=0.03
VI	2008-C08-014	Urine	21	4	4	<=0.5	4	<=0.5	<=2	<=0.03
VI	2008-C08-015	Urine	77	<=2	>16	<=0.5	8	<=0.5	8	0.12
VI	2008-C08-018	Urine	42	<=2	>16	<=0.5	8	<=0.5	<=2	<=0.03
VI	2008-C08-023	Urine	23	4	>16	<=0.5	16	<=0.5	8	<=0.03
VI	2008-C08-024	Urine	56	16	>16	<=0.5	8	<=0.5	4	1
VI	2008-C08-025	Urine	33	<=2	>16	<=0.5	16	<=0.5	16	<=0.03
VI	2008-C08-030	Urine	33	<=2	2	<=0.5	4	<=0.5	<=2	<=0.03
VI	2008-C08-031	Urine	71	<=2	4	<=0.5	4	<=0.5	<=2	>2
VI	2008-C08-042	Urine	44	4	>16	<=0.5	8	<=0.5	4	<=0.03
VI	2008-C08-047	Urine	74	<=2	>16	<=0.5	8	<=0.5	4	0.25
VI	2008-C08-049	Urine	0	<=2	>16	<=0.5	8	<=0.5	4	<=0.03
VI	2008-C08-186	Urine	0	4	>16	<=0.5	8	<=0.5	4	<=0.03
VI	2008-C08-195	Urine	0	<=2	4	<=0.5	4	<=0.5	<=2	<=0.03
VI	2008-C08-212	Blood	78	<=2	>16	>16	>16	>16	>16	0.25
VI	2008-C08-219	Blood		4	>16	<=0.5	8	<=0.5	<=2	0.25
VI	2008-C08-237	Blood	76	<=2	>16	16	>16	16	>16	>2
VI	2008-S11-002	Blood	81	<=2	>16	<=0.5	8	<=0.5	4	<=0.03
VI	2008-S11-005	Blood	59	<=2	>16	<=0.5	8	<=0.5	<=2	<=0.03
VI	2008-S11-006	Urine	48	<=2	4	<=0.5	4	<=0.5	<=2	<=0.03
VI	2008-S11-015	Urine	66	<=2	4	<=0.5	4	<=0.5	<=2	<=0.03
VI	2008-S11-022	Urine	88	<=2	>16	<=0.5	8	<=0.5	4	>2
VI	2008-S11-026	Urine	13	<=2	>16	<=0.5	16	<=0.5	<=2	<=0.03
VI	2008-S11-035	Urine	0(6m)	<=2	>16	<=0.5	16	<=0.5	<=2	<=0.03
VI	2008-S11-036	Urine	81	<=2	>16	<=0.5	16	<=0.5	<=2	<=0.03
VI	2008-S11-037	Urine	79	<=2	>16	<=0.5	8	<=0.5	16	<=0.03
VI	2008-S11-041	Urine	45	<=2	4	<=0.5	4	<=0.5	<=2	0.25
VI	2008-S11-042	Urine	54	<=2	>16	<=0.5	8	<=0.5	4	0.25
VI	2008-S11-043	Urine	58	<=2	>16	<=0.5	16	<=0.5	4	<=0.03
VI	2008-S11-045	Urine	76	<=2	2	<=0.5	4	<=0.5	<=2	<=0.03
VI	2008-S11-046	Urine	74	4	>16	<=0.5	8	<=0.5	4	<=0.03
VI	2008-S11-050	Urine	69	<=2	>16	8	>16	16	>16	0.25
VI	2008-S11-191	Urine	9	4	2	<=0.5	2	<=0.5	<=2	<=0.03
VI	2008-N21-005	Urine	27	4	>16	<=0.5	8	<=0.5	4	0.25
VI	2008-N21-010	Urine	56	<=2	>16	<=0.5	8	<=0.5	4	0.25
VI	2008-N21-011	Urine	49	4	>16	<=0.5	8	<=0.5	4	>2
VI	2008-N21-021	Urine	89	<=2	>16	16	>16	16	>16	0.25
VI	2008-N21-023	Urine	27	<=2	>16	<=0.5	16	<=0.5	8	0.5
VI	2008-N21-025	Urine	22	<=2	>16	<=0.5	8	<=0.5	<=2	<=0.03
VI	2008-N21-026	Urine	22	4	>16	<=0.5	8	<=0.5	4	0.12
VI	2008-N21-027	Urine	40	8	>16	<=0.5	8	<=0.5	4	<=0.03
VI	2008-N21-028	Urine	62	<=2	>16	<=0.5	16	<=0.5	16	<=0.03
VI	2008-N21-029	Urine	67	<=2	>16	<=0.5	8	<=0.5	8	<=0.03
VI	2008-N21-032	Urine	82	4	>16	<=0.5	16	<=0.5	16	<=0.03
VI	2008-N21-033	Urine	36	4	>16	<=0.5	8	<=0.5	4	<=0.03
VI	2008-N21-040	Blood	73	4	>16	<=0.5	8	<=0.5	4	0.25
VI	2008-N21-049	Urine	98	<=2	>16	<=0.5	8	<=0.5	4	>2
VI	2008-N21-181	Urine	1	4	4	<=0.5	4	<=0.5	4	<=0.03
VI	2008-N21-185	Urine	2	4	4	<=0.5	4	<=0.5	<=2	1
VI	2008-N21-189	Urine	0	8	>16	<=0.5	16	<=0.5	>16	<=0.03
VI	2008-N21-193	Urine	10	4	>16	<=0.5	8	<=0.5	4	0.06
VI	2008-N21-196	Urine	1	4	4	<=0.5	4	<=0.5	<=2	<=0.03
VI	2008-N21-197	Urine	0	8	>16	<=0.5	4	<=0.5	4	1
VI	2008-N21-208	Blood	60	<=2	4	<=0.5	4	<=0.5	<=2	<=0.03
VI	2008-N21-216	Blood	64	4	>16	<=0.5	8	<=0.5	4	>2
VI	2008-N21-217	Blood	63	4	>16	<=0.5	8	<=0.5	4	<=0.03
VI	2008-N21-219	Blood	51	4	>16	<=0.5	16	<=0.5	16	>2
VI	2008-N21-227	Blood	33	4	4	<=0.5	4	<=0.5	<=2	<=0.03
VI	2008-N21-230	Blood	60	<=2	>16	>16	>16	>16	>16	>2

VI	2008-N21-233	Blood	65	<=2	>16	<=0.5	8	<=0.5	4	<=0.03
VI	2008-N21-234	Blood	71	4	>16	<=0.5	8	<=0.5	<=2	<=0.03
VI	2008-N21-237	Blood	70	4	>16	<=0.5	4	<=0.5	<=2	1
VI	2008-N21-245	Blood	66	<=2	4	<=0.5	4	<=0.5	<=2	<=0.03
VI	2008-N21-249	Blood	74	4	4	<=0.5	4	<=0.5	<=2	<=0.03
VI	2008-N21-250	Blood	60	<=2	>16	2	>16	>16	>16	0.5
VI	2008-C09-001	Urine	10	<=2	>16	<=0.5	16	<=0.5	8	<=0.03
VI	2008-C09-002	Urine	56	<=2	>16	<=0.5	8	<=0.5	<=2	0.25
VI	2008-C09-003	Urine	69	>32	>16	>16	>16	8	>16	>2
VI	2008-C09-008	Urine	73	8	>16	16	>16	>16	>16	>2
VI	2008-C09-011	Urine	77	<=2	>16	<=0.5	8	<=0.5	<=2	<=0.03
VI	2008-C09-020	Urine	23	4	>16	<=0.5	>16	<=0.5	16	<=0.03
VI	2008-C09-024	Urine	55	<=2	>16	<=0.5	8	<=0.5	<=2	<=0.03
VI	2008-C09-032	Urine	81	4	>16	8	>16	16	>16	>2
VI	2008-C09-035	Urine	79	>32	>16	16	>16	>16	>16	>2
VI	2008-C09-036	Urine	87	<=2	>16	>16	>16	>16	>16	>2
VI	2008-C09-042	Urine	79	4	4	<=0.5	4	<=0.5	<=2	<=0.03
VI	2008-C09-047	Urine	45	<=2	>16	<=0.5	8	<=0.5	<=2	<=0.03
VI	2008-C09-049	Urine	77	8	>16	2	16	1	>16	>2
VI	2008-C09-207	Blood	74	<=2	>16	<=0.5	8	<=0.5	4	0.25
VI	2008-C09-212	Blood	42	4	4	<=0.5	2	<=0.5	<=2	<=0.03
VI	2008-C09-214	Blood	34	<=2	>16	<=0.5	16	<=0.5	16	<=0.03
VI	2008-C09-222	Blood	32	4	>16	<=0.5	16	<=0.5	8	0.25
VI	2008-C09-227	Blood	42	8	>16	8	>16	16	>16	>2
VI	2008-C09-230	Blood	54	>32	>16	2	>16	4	>16	<=0.03
VI	2008-C09-233	Blood	75	<=2	>16	<=0.5	16	<=0.5	4	0.5
VI	2008-C09-238	Blood	50	<=2	>16	<=0.5	8	<=0.5	4	0.12
VI	2008-C09-242	Blood	94	4	2	<=0.5	4	<=0.5	<=2	<=0.03
VI	2008-C09-244	Blood	65	8	>16	<=0.5	16	<=0.5	8	0.5
VI	2008-C09-250	Blood	60	<=2	>16	<=0.5	8	<=0.5	<=2	<=0.03
VI	2008-C10-001	Blood	77	<=2	>16	<=0.5	8	<=0.5	<=2	0.25
VI	2008-C10-002	Urine	60	<=2	>16	<=0.5	>16	<=0.5	16	<=0.03
VI	2008-C10-003	Urine	84	<=2	2	<=0.5	4	<=0.5	<=2	0.25
VI	2008-C10-004	Urine	56	>32	>16	>16	>16	>16	>16	>2
VI	2008-C10-015	Urine	40	4	>16	<=0.5	16	<=0.5	16	<=0.03
VI	2008-C10-016	Urine	34	<=2	2	<=0.5	4	<=0.5	<=2	<=0.03
VI	2008-C10-018	Urine	48	<=2	>16	<=0.5	16	<=0.5	4	<=0.03
VI	2008-C10-026	Blood	72	4	8	<=0.5	8	<=0.5	<=2	<=0.03
VI	2008-C10-027	Blood	24	<=2	>16	<=0.5	16	<=0.5	8	0.25
VI	2008-C10-028	Blood	42	<=2	>16	<=0.5	8	<=0.5	<=2	<=0.03
VI	2008-C10-029	Urine	56	<=2	>16	<=0.5	8	<=0.5	4	0.25
VI	2008-C10-041	Urine	24	<=2	4	<=0.5	4	<=0.5	<=2	<=0.03
VI	2008-C10-042	Blood	71	4	4	<=0.5	4	<=0.5	<=2	<=0.03
VI	2008-C10-043	Urine	73	<=2	>16	<=0.5	8	<=0.5	4	>2
VI	2008-C10-047	Urine	59	4	2	<=0.5	4	<=0.5	<=2	<=0.03
VI	2008-C10-048	Urine	78	<=2	4	<=0.5	4	<=0.5	<=2	<=0.03
VI	2008-C10-204	Blood	66	<=2	>16	<=0.5	16	<=0.5	8	<=0.03
VI	2008-C10-213	Blood	50	<=2	4	<=0.5	4	<=0.5	<=2	<=0.03
VI	2008-C10-215	Blood	21	4	4	<=0.5	4	<=0.5	<=2	<=0.03
VI	2008-C10-222	Blood	72	<=2	>16	<=0.5	16	<=0.5	4	1
VI	2008-C10-224	Blood	80	4	4	<=0.5	8	<=0.5	<=2	<=0.03
VI	2008-C10-225	Blood	46	<=2	>16	<=0.5	16	<=0.5	<=2	<=0.03
VI	2008-C10-227	Blood	58	4	4	<=0.5	4	<=0.5	4	<=0.03
VI	2008-C10-234	Blood	73	<=2	>16	8	>16	>16	>16	>2
VI	2008-C10-239	Blood	69	4	4	<=0.5	8	<=0.5	<=2	0.5
VI	2008-C10-247	Blood	63	8	>16	<=0.5	>16	<=0.5	16	<=0.03
VI	2008-C10-248	Blood	83	4	4	<=0.5	4	<=0.5	<=2	<=0.03
VI	2008-C10-249	Blood	87	4	>16	<=0.5	16	<=0.5	16	<=0.03
VI	2008-E04-002	Blood	75	<=2	<=1	<=0.5	1	<=0.5	<=2	<=0.03
VI	2008-E04-003	Blood	68	4	4	<=0.5	4	<=0.5	<=2	<=0.03
VI	2008-E04-008	Blood	56	4	>16	4	8	1	>16	0.25

VI	2008-E04-010	Blood	51	<=2	>16	<=0.5	8	<=0.5	4	<=0.03
VI	2008-E04-012	Blood	75	<=2	>16	8	>16	16	>16	>2
VI	2008-E04-014	Blood	74	4	>16	<=0.5	8	<=0.5	4	<=0.03
VI	2008-E04-015	Blood	54	4	4	<=0.5	4	<=0.5	<=2	<=0.03
VI	2008-E04-017	Blood	62	4	2	<=0.5	1	<=0.5	<=2	<=0.03
VI	2008-E04-019	Blood	79	4	>16	16	>16	16	>16	<=0.03
VI	2008-E04-020	Blood	66	4	>16	<=0.5	16	<=0.5	8	<=0.03
VI	2008-E04-026	Blood	48	4	>16	<=0.5	4	<=0.5	<=2	<=0.03
VI	2008-E04-027	Blood	45	>32	>16	>16	16	8	>16	>2
VI	2008-E04-029	Blood	67	<=2	>16	<=0.5	8	<=0.5	<=2	<=0.03
VI	2008-E04-031	Blood	73	<=2	4	<=0.5	4	<=0.5	<=2	0.25
VI	2008-E04-033	Blood	63	4	4	<=0.5	4	<=0.5	<=2	<=0.03
VI	2008-E04-036	Blood	53	8	8	<=0.5	8	<=0.5	4	<=0.03
VI	2008-E04-039	Blood	70	<=2	>16	<=0.5	8	<=0.5	4	<=0.03
VI	2008-E04-040	Blood	88	<=2	>16	<=0.5	8	<=0.5	<=2	0.06
VI	2008-E04-043	Blood	87	<=2	>16	<=0.5	8	<=0.5	<=2	<=0.03
VI	2008-E04-050	Blood	80	<=2	>16	<=0.5	16	<=0.5	16	<=0.03
VII	2010-S01-004	Other	45	<=4	4	<=1		<=1	<=2	<=0.06
VII	2010-S01-009	Urine	80	<=4	>16	<=1		<=1	4	0.25
VII	2010-S01-011	Urine	81	<=4	>16	>16		4	>16	>2
VII	2010-S01-016	Urine	0	<=4	4	<=1		<=1	<=2	<=0.06
VII	2010-S01-017	Urine	60	<=4	<=2	<=1		<=1	<=2	<=0.06
VII	2010-S01-018	Urine	85	8	>16	<=1		<=1	8	0.25
VII	2010-S01-020	Urine	83	<=4	8	<=1		<=1	<=2	<=0.06
VII	2010-S01-021	Urine	59	<=4	>16	16		>16	>16	>2
VII	2010-S01-022	Urine	84	<=4	>16	<=1		<=1	4	<=0.06
VII	2010-S01-027	Other	68	<=4	>16	<=1		<=1	<=2	<=0.06
VII	2010-S01-028	Urine	81	<=4	>16	4		<=1	>16	0.5
VII	2010-S01-030	Urine	49	<=4	>16	<=1		8	>16	0.5
VII	2010-S01-033	Urine	72	<=4	>16	16		>16	>16	0.5
VII	2010-S01-039	Urine	61	<=4	>16	<=1		<=1	4	>2
VII	2010-S01-041	Urine	59	<=4	4	<=1		<=1	4	0.12
VII	2010-S01-046	Urine	48	<=4	4	<=1		<=1	<=2	<=0.06
VII	2010-S01-047	Urine	30	<=4	>16	<=1		<=1	8	0.25
VII	2010-S01-204	Blood	57	<=4	>16	<=2		<=1	4	<=0.06
VII	2010-S01-216	Blood	88	<=4	>16	<=2		<=1	4	<=0.06
VII	2010-S01-218	Blood	68	<=4	>16	<=2		<=1	8	0.25
VII	2010-S01-227	Blood	65	<=4	>16	<=2		<=1	8	<=0.06
VII	2010-S01-230	Blood	75	<=4	>16	<=2		<=1	4	<=0.06
VII	2010-S01-233	Blood	83	<=4	>16	<=2		<=1	4	<=0.06
VII	2010-S01-234	Other	55	<=4	>16	4		8	>16	>2
VII	2010-S01-240	Blood	70	8	4	<=2		<=1	4	<=0.06
VII	2010-S01-241	Blood	95	<=4	8	<=2		<=1	2	<=0.06
VII	2010-S01-243	Blood	55	<=4	4	<=2		<=1	2	<=0.06
VII	2010-S01-245	Blood	28	<=4	<=2	<=2		<=1	2	>2
VII	2010-S01-246	Blood	62	8	>16	>16		16	>16	>2
VII	2010-S01-247	Blood	52	<=4	>16	<=2		<=1	4	<=0.06
VII	2010-C01-003	Urine	72	<=4	4	<=1		<=1	<=2	<=0.06
VII	2010-C01-005	Urine	19	<=4	4	<=1		<=1	<=2	<=0.06
VII	2010-C01-007	Other	51	<=4	>16	<=1		<=1	4	<=0.06
VII	2010-C01-012	Other	51	<=4	>16	<=1		<=1	4	0.25
VII	2010-C01-015	Other	44	<=4	>16	<=1		<=1	8	0.25
VII	2010-C01-017	Blood	75	<=4	>16	16		2	>16	>2
VII	2010-C01-018	Blood	43	<=4	>16	<=2		<=1	8	>2
VII	2010-C01-020	Blood	59	<=4	>16	<=2		<=1	8	0.25
VII	2010-C01-028	Blood	82	<=4	>16	<=2		<=1	8	<=0.06
VII	2010-C01-029	Urine	15	<=4	>16	<=1		<=1	<=2	<=0.06
VII	2010-C01-030	Urine	78	<=4	<=2	<=1		<=1	<=2	0.12
VII	2010-C01-031	Urine	4	<=4	>16	<=1		<=1	16	<=0.06
VII	2010-C01-033	Urine	9	<=4	>16	<=1		<=1	<=2	<=0.06
VII	2010-C01-035	Urine	0	<=4	>16	<=1		<=1	4	<=0.06

VII	2010-C01-037	Urine	75	<=4	>16	4		16	>16	>2
VII	2010-C01-041	Urine	33	<=4	<=2	<=1		<=1	4	<=0.06
VII	2010-C01-046	Urine	20	<=4	>16	<=1		<=1	4	0.12
VII	2010-C01-047	Urine	79	<=4	>16	2		<=1	>16	>2
VII	2010-C01-049	Urine	46	<=4	>16	<=1		<=1	4	<=0.06
VII	2010-C01-050	Urine	71	<=4	>16	<=1		<=1	4	0.25
VII	2010-C01-183	Urine	1	<=4	>16	<=1		<=1	4	<=0.06
VII	2010-C02-001	Blood	57	<=4	>16	>16		>16	>16	>2
VII	2010-C02-002	Blood	69	<=4	<=2	<=2		<=1	<=1	<=0.06
VII	2010-C02-003	Blood	50	<=4	<=2	<=2		<=1	<=1	<=0.06
VII	2010-C02-004	Blood	58	<=4	>16	<=2		<=1	2	<=0.06
VII	2010-C02-006	Blood	52	<=4	>16	<=2		<=1	4	<=0.06
VII	2010-C02-007	Blood	54	<=4	>16	<=2		<=1	2	<=0.06
VII	2010-C02-008	Urine	55	<=4	>16	8		16	>16	>2
VII	2010-C02-009	Urine	64	<=4	>16	<=1		<=1	4	<=0.06
VII	2010-C02-013	Urine	93	<=4	>16	<=1		<=1	4	<=0.06
VII	2010-C02-015	Urine	69	<=4	>16	4		16	>16	0.5
VII	2010-C02-017	Urine	19	<=4	<=2	<=1		<=1	<=2	<=0.06
VII	2010-C02-020	Urine	49	<=4	>16	4		<=1	>16	0.25
VII	2010-C02-024	Urine	49	<=4	<=2	<=1		<=1	<=2	<=0.06
VII	2010-C02-025	Urine	50	<=4	>16	<=1		<=1	<=2	<=0.06
VII	2010-C02-027	Urine	42	<=4	>16	8		16	>16	<=0.06
VII	2010-C02-029	Urine	43	<=4	>16	4		16	>16	<=0.06
VII	2010-C02-030	Urine	82	<=4	>16	<=1		<=1	<=2	<=0.06
VII	2010-C02-031	Urine	74	<=4	<=2	<=1		<=1	<=2	<=0.06
VII	2010-C02-032	Urine	52	<=4	>16	<=1		<=1	4	0.5
VII	2010-C02-033	Other	70	<=4	>16	<=1		<=1	<=2	<=0.06
VII	2010-C02-035	Urine	73	8	>16	>16		>16	>16	>2
VII	2010-C02-040	Blood	31	<=4	>16	<=2		<=1	2	1
VII	2010-C02-041	Blood	75	<=4	>16	<=2		<=1	8	>2
VII	2010-C02-047	Blood	26	<=4	<=2	<=2		<=1	2	<=0.06
VII	2010-C02-048	Blood	78	<=4	>16	<=2		<=1	8	0.25
VII	2010-C02-049	Urine	47	<=4	>16	<=1		<=1	4	0.25
VII	2010-C02-050	Urine	81	<=4	>16	<=1		<=1	8	<=0.06
VII	2010-C02-207	Blood	73	8	>16	<=2		2	2	<=0.06
VII	2010-C02-212	Blood	73	<=4	4	<=2		<=1	<=1	<=0.06
VII	2010-C02-219	Blood	44	<=4	<=2	<=2		<=1	2	<=0.06
VII	2010-C02-224	Blood	64	<=4	>16	<=2		<=1	8	>2
VII	2010-C02-225	Blood	71	<=4	4	<=2		<=1	2	<=0.06
VII	2010-C02-226	Blood	80	<=4	<=2	<=2		<=1	2	<=0.06
VII	2010-C02-227	Other	58	<=4	<=2	<=1		<=1	<=2	<=0.06
VII	2010-C02-231	Blood	58	8	>16	<=2		<=1	4	<=0.06
VII	2010-C02-232	Blood	73	<=4	4	<=2		<=1	2	0.25
VII	2010-C02-233	Blood	38	<=4	>16	<=2		<=1	2	<=0.06
VII	2010-C02-244	Blood	77	<=4	>16	<=2		<=1	8	<=0.06
VII	2010-C04-001	Blood	64	<=4	>16	<=2		<=1	2	<=0.06
VII	2010-C04-003	Urine	22	<=4	4	<=1		<=1	<=2	<=0.06
VII	2010-C04-009	Urine	73	<=4	>16	16		4	>16	>2
VII	2010-C04-010	Urine	0	<=4	<=2	<=1		<=1	<=2	<=0.06
VII	2010-C04-013	Urine	54	<=4	>16	<=1		<=1	16	<=0.06
VII	2010-C04-016	Urine	72	<=4	>16	<=1		<=1	4	0.25
VII	2010-C04-021	Urine	71	<=4	>16	<=1		<=1	16	0.25
VII	2010-C04-023	Urine	67	<=4	>16	<=1		<=1	4	1
VII	2010-C04-026	Blood	83	<=4	>16	<=2		<=1	2	0.12
VII	2010-C04-031	Other	46	<=4	4	<=1		<=1	<=2	<=0.06
VII	2010-C04-035	Urine	67	<=4	<=2	<=1		<=1	<=2	<=0.06
VII	2010-C04-039	Urine	88	<=4	>16	<=1		<=1	>16	<=0.06
VII	2010-C04-042	Blood	75	<=4	>16	4		<=1	>16	0.12
VII	2010-C04-044	Blood	88	<=4	<=2	<=2		<=1	2	<=0.06
VII	2010-C04-230	Blood	26	<=4	<=2	<=2		<=1	2	<=0.06
VII	2010-C04-246	Blood	80	<=4	>16	<=2		<=1	>16	0.25

VII	2010-S05-001	Urine	78	<=4	4	<=1		<=1	<=2	<=0.06
VII	2010-S05-005	Urine	69	<=4	>16	<=1		<=1	<=2	0.25
VII	2010-S05-009	Urine	87	<=4	>16	>16		>16	>16	>2
VII	2010-S05-017	Urine	78	<=4	>16	>16		>16	>16	>2
VII	2010-S05-018	Urine	68	<=4	4	<=1		<=1	4	<=0.06
VII	2010-S05-019	Urine	90	<=4	>16	<=1		<=1	<=2	>2
VII	2010-S05-020	Urine	83	<=4	>16	<=1		<=1	4	>2
VII	2010-S05-023	Blood	35	<=4	>16	<=2		<=1	16	<=0.06
VII	2010-S05-027	Blood	89	<=4	>16	16		16	>16	>2
VII	2010-S05-028	Urine	98	<=4	>16	<=1		<=1	4	<=0.06
VII	2010-S05-029	Urine	58	<=4	<=2	<=1		<=1	<=2	<=0.06
VII	2010-S05-032	Urine	3	<=4	>16	<=1		<=1	>16	<=0.06
VII	2010-S05-036	Urine		<=4	>16	<=1		<=1	4	<=0.06
VII	2010-S05-037	Urine	53	<=4	4	<=1		<=1	<=2	<=0.06
VII	2010-S05-038	Blood	91	8	>16	>16		>16	>16	>2
VII	2010-S05-040	Urine	90	<=4	>16	16		16	>16	>2
VII	2010-S05-041	Urine	58	<=4	>16	<=1		<=1	<=2	0.5
VII	2010-S05-043	Urine	71	<=4	4	<=1		<=1	<=2	<=0.06
VII	2010-S05-045	Blood	64	8	>16	>16		>16	>16	>2
VII	2010-S05-047	Urine	76	<=4	>16	16		8	>16	>2
VII	2010-S05-181	Urine		<=4	>16	<=1		<=1	4	<=0.06
VII	2010-S05-185	Other	unk	<=4	<=2	<=1		<=1	<=2	<=0.06
VII	2010-S05-188	Urine	3	<=4	>16	<=1		<=1	8	<=0.06
VII	2010-S05-192	Urine	9	<=4	>16	<=1		<=1	8	0.25
VII	2010-S05-216	Blood	73	<=4	>16	<=2		<=1	>16	<=0.06
VII	2010-S05-218	Blood	73	16	>16	>16		>16	>16	>2
VII	2010-S05-247	Blood	69	<=4	>16	<=2		<=1	4	1
VII	2010-S06-003	Urine	53	<=4	>16	>16		>16	>16	>2
VII	2010-S06-009	Urine	59	<=4	>16	<=1		<=1	4	<=0.06
VII	2010-S06-011	Urine	39	<=4	>16	8		>16	>16	1
VII	2010-S06-012	Urine	66	<=4	>16	<=1		<=1	4	<=0.06
VII	2010-S06-013	Urine	75	<=4	>16	<=1		<=1	<=2	<=0.06
VII	2010-S06-016	Urine	77	<=4	>16	<=1		<=1	8	<=0.06
VII	2010-S06-017	Urine	75	<=4	<=2	<=1		<=1	<=2	0.12
VII	2010-S06-021	Urine	80	<=4	>16	4		2	>16	>2
VII	2010-S06-023	Urine	25	<=4	>16	8		16	>16	>2
VII	2010-S06-026	Urine	59	<=4	>16	<=1		<=1	<=2	>2
VII	2010-S06-032	Urine	19	<=4	<=2	<=1		2	<=2	<=0.06
VII	2010-S06-033	Urine	68	<=4	>16	<=1		<=1	4	>2
VII	2010-S06-034	Urine	73	<=4	<=2	<=1		<=1	<=2	<=0.06
VII	2010-S06-037	Urine	53	<=4	<=2	<=1		<=1	<=2	<=0.06
VII	2010-S06-039	Urine	81	<=4	>16	16		>16	>16	>2
VII	2010-S06-040	Urine	76	<=4	>16	<=1		<=1	16	<=0.06
VII	2010-S06-041	Urine	45	<=4	4	<=1		<=1	<=2	<=0.06
VII	2010-S06-043	Urine	53	<=4	>16	<=1		<=1	4	<=0.06
VII	2010-S06-045	Urine	51	<=4	<=2	<=1		<=1	<=2	<=0.06
VII	2010-S06-048	Urine	79	<=4	>16	4		16	>16	>2
VII	2010-S06-049	Urine	74	<=4	>16	<=1		<=1	4	<=0.06
VII	2010-S06-050	Urine	27	<=4	<=2	<=1		<=1	<=2	<=0.06
VII	2010-S06-226	Blood	33	<=4	<=2	<=2		<=1	2	<=0.06
VII	2010-S06-237	Blood	33	<=4	4	<=2		<=1	16	<=0.06
VII	2010-S06-243	Blood	91	<=4	>16	<=2		<=1	2	<=0.06
VII	2010-S07-001	Blood	59	<=4	4	<=2		<=1	2	<=0.06
VII	2010-S07-002	Blood	33	<=4	<=2	<=2		<=1	2	<=0.06
VII	2010-S07-006	Blood	40	8	>16	>16		>16	>16	>2
VII	2010-S07-007	Urine	66	<=4	>16	16		2	>16	>2
VII	2010-S07-012	Blood	77	<=4	>16	<=2		<=1	>16	>2
VII	2010-S07-013	Blood	58	<=4	4	<=2		<=1	2	0.12
VII	2010-S07-015	Blood	60	8	>16	<=2		<=1	4	0.25
VII	2010-S07-023	Urine	1	<=4	>16	8		2	>16	<=0.06
VII	2010-S07-025	Blood	59	<=4	>16	<=2		<=1	>16	<=0.06

VII	2010-S07-038	Urine	68	<=4	>16	>16		4	>16	>2
VII	2010-S07-039	Other	87	<=4	>16	16		>16	>16	>2
VII	2010-S07-047	Urine	83	<=4	>16	4		4	>16	>2
VII	2010-S07-201	Blood	55	<=4	4	<=2		<=1	2	<=0.06
VII	2010-S07-207	Blood	82	<=4	>16	<=2		<=1	8	0.25
VII	2010-S07-208	Blood	81	<=4	4	<=2		<=1	4	<=0.06
VII	2010-S07-209	Blood	62	<=4	>16	8		16	>16	<=0.06
VII	2010-S07-210	Blood	91	16	>16	8		16	>16	>2
VII	2010-S07-219	Blood	85	<=4	>16	4		16	>16	>2
VII	2010-S07-220	Blood	32	<=4	>16	<=2		<=1	4	0.25
VII	2010-S07-229	Blood	27	<=4	>16	4		8	>16	0.5
VII	2010-S07-230	Blood	53	<=4	<=2	<=2		<=1	4	<=0.06
VII	2010-S07-237	Blood	32	8	>16	<=2		<=1	8	>2
VII	2010-S07-246	Blood	74	<=4	>16	8		16	>16	>2
VII	2010-S07-249	Blood	70	<=4	>16	<=2		<=1	2	>2
VII	2010-N05-003	Urine	75	<=4	<=2	<=1		<=1	<=2	0.12
VII	2010-N05-006	Urine	46	<=4	>16	<=1		<=1	<=2	<=0.06
VII	2010-N05-007	Urine	20	<=4	>16	<=1		<=1	4	<=0.06
VII	2010-N05-009	Urine	26	<=4	4	<=1		<=1	<=2	<=0.06
VII	2010-N05-010	Urine	94	16	<=2	<=1		<=1	<=2	<=0.06
VII	2010-N05-011	Urine	39	<=4	>16	<=1		<=1	<=2	>2
VII	2010-N05-012	Urine	62	<=4	>16	<=2		<=1	>16	0.12
VII	2010-N05-013	Urine	89	<=4	>16	<=1		<=1	4	>2
VII	2010-N05-014	Other	30	<=4	<=2	<=1		<=1	<=2	<=0.06
VII	2010-N05-016	Urine	43	<=4	>16	4		<=1	>16	0.25
VII	2010-N05-019	Urine	60	<=4	>16	<=1		<=1	4	>2
VII	2010-N05-020	Urine	50	<=4	<=2	<=1		<=1	<=2	<=0.06
VII	2010-N05-022	Urine	55	<=4	>16	<=1		<=1	4	<=0.06
VII	2010-N05-025	Urine	66	<=4	4	<=1		<=1	<=2	<=0.06
VII	2010-N05-028	Urine	34	<=4	4	<=1		<=1	<=2	<=0.06
VII	2010-N05-029	Urine	48	<=4	>16	<=2		<=1	16	0.5
VII	2010-N05-033	Urine	56	<=4	>16	<=1		<=1	4	0.5
VII	2010-N05-035	Urine	40	<=4	4	<=1		<=1	<=2	<=0.06
VII	2010-N05-039	Urine	76	<=4	>16	<=2		2	>16	0.5
VII	2010-N05-040	Urine	79	<=4	<=2	<=1		<=1	<=2	<=0.06
VII	2010-N05-043	Urine	94	<=4	>16	<=1		<=1	4	<=0.06
VII	2010-N05-044	Other	51	<=4	4	<=1		<=1	<=2	<=0.06
VII	2010-N05-045	Urine	25	<=4	4	<=1		<=1	<=2	<=0.06
VII	2010-N05-047	Other	63	<=4	>16	<=1		2	4	0.25
VII	2010-N05-048	Urine	51	<=4	>16	<=1		<=1	<=2	<=0.06
VII	2010-N05-214	Blood		<=4	<=2	<=2		<=1	2	<=0.06
VII	2010-N06-004	Blood	81	<=4	4	<=2		<=1	2	<=0.06
VII	2010-N06-005	Blood	82	<=4	4	<=2		<=1	4	<=0.06
VII	2010-N06-007	Urine	74	<=4	4	<=1		<=1	<=2	<=0.06
VII	2010-N06-008	Urine	83	8	4	<=1		<=1	<=2	<=0.06
VII	2010-N06-010	Urine	27	<=4	<=2	<=1		<=1	<=2	<=0.06
VII	2010-N06-011	Urine	72	<=4	>16	<=1		<=1	8	<=0.06
VII	2010-N06-014	Urine	51	<=4	>16	<=1		<=1	<=2	<=0.06
VII	2010-N06-018	Urine	28	<=4	>16	<=1		<=1	<=2	<=0.06
VII	2010-N06-019	Urine	80	<=4	>16	8		16	>16	0.25
VII	2010-N06-021	Urine	60	<=4	<=2	<=1		<=1	<=2	<=0.06
VII	2010-N06-022	Urine	66	<=4	>16	<=1		<=1	4	<=0.06
VII	2010-N06-031	Urine	28	<=4	>16	<=1		<=1	4	<=0.06
VII	2010-N06-033	Urine	37	<=4	<=2	<=1		<=1	<=2	<=0.06
VII	2010-N06-035	Urine	92	<=4	>16	<=1		<=1	4	<=0.06
VII	2010-N06-040	Urine	85	<=4	>16	4		16	>16	>2
VII	2010-N06-042	Urine	58	<=4	>16	<=1		<=1	16	<=0.06
VII	2010-N06-043	Urine	69	<=4	>16	<=1		<=1	4	<=0.06
VII	2010-N06-044	Urine	72	<=4	>16	>16		>16	>16	>2
VII	2010-N06-050	Urine	36	<=4	4	<=1		<=1	<=2	<=0.06
VII	2010-N06-212	Blood	80	<=4	4	<=2		<=1	2	<=0.06

VII	2010-N06-214	Blood	76	<=4	>16	<=2		2	>16	>2
VII	2010-N06-221	Blood	69	<=4	>16	<=2		<=1	4	<=0.06
VII	2010-N06-225	Blood	26	<=4	4	<=2		<=1	2	<=0.06
VII	2010-N06-230	Blood	51	<=4	>16	<=2		<=1	4	<=0.06
VII	2010-N06-247	Blood	70	<=4	>16	<=2		<=1	4	0.12
VII	2010-N07-001	Urine	50	<=4	<=2	<=1		<=1	<=2	<=0.06
VII	2010-N07-002	Urine	67	<=4	<=2	<=1		<=1	<=2	<=0.06
VII	2010-N07-003	Urine	84	<=4	>16	<=1		<=1	16	>2
VII	2010-N07-004	Urine	79	<=4	>16	<=1		<=1	8	<=0.06
VII	2010-N07-008	Urine	68	<=4	4	<=1		<=1	<=2	0.25
VII	2010-N07-010	Urine	47	<=4	>16	2		2	>16	<=0.06
VII	2010-N07-012	Urine	57	<=4	<=2	<=1		<=1	<=2	<=0.06
VII	2010-N07-014	Urine	76	>32	>16	>16		16	>16	>2
VII	2010-N07-017	Other	68	<=4	4	<=1		<=1	<=2	<=0.06
VII	2010-N07-021	Urine	56	<=4	>16	<=1		<=1	16	0.5
VII	2010-N07-022	Blood	67	<=4	>16	<=2		<=1	4	0.25
VII	2010-N07-023	Urine	94	32	>16	8		16	>16	>2
VII	2010-N07-026	Blood	47	<=4	<=2	<=2		<=1	2	0.12
VII	2010-N07-028	Blood	51	<=4	>16	>16		>16	>16	>2
VII	2010-N07-030	Urine	75	<=4	<=2	<=1		<=1	<=2	<=0.06
VII	2010-N07-032	Blood	80	<=4	>16	<=2		<=1	>16	<=0.06
VII	2010-N07-033	Blood	65	<=4	16	<=2		<=1	2	0.12
VII	2010-N07-034	Urine	76	<=4	>16	<=1		<=1	8	0.25
VII	2010-N07-037	Urine	41	<=4	>16	16		>16	>16	>2
VII	2010-N07-039	Urine	20	<=4	4	<=1		<=1	<=2	<=0.06
VII	2010-N07-040	Urine	41	<=4	<=2	<=1		<=1	<=2	<=0.06
VII	2010-N07-042	Blood	52	<=4	>16	<=2		<=1	2	<=0.06
VII	2010-N07-046	Blood	53	<=4	>16	<=2		4	>16	>2
VII	2010-N07-201	Blood	58	<=4	4	<=2		<=1	2	<=0.06
VII	2010-N07-205	Blood	68	<=4	4	<=2		<=1	2	<=0.06
VII	2010-N07-225	Blood	79	<=4	>16	<=2		<=1	4	<=0.06
VII	2010-N07-237	Blood	74	<=4	>16	<=2		<=1	8	<=0.06
VII	2010-N07-238	Blood	40	<=4	<=2	<=2		<=1	2	<=0.06
VII	2010-N07-243	Blood	64	<=4	>16	<=2		<=1	8	<=0.06
VII	2010-N07-247	Blood	61	<=4	<=2	<=2		<=1	2	<=0.06
VII	2010-N07-250	Blood	55	<=4	>16	<=2		<=1	8	<=0.06
VII	2010-S08-002	Urine	60	16	>16	<=1		<=1	4	<=0.06
VII	2010-S08-003	Urine	52	8	>16	<=1		<=1	8	<=0.06
VII	2010-S08-004	Urine	28	<=4	<=2	<=1		<=1	<=2	<=0.06
VII	2010-S08-013	Urine	73	<=4	>16	8		4	>16	>2
VII	2010-S08-017	Blood	68	<=4	>16	<=2		<=1	>16	<=0.06
VII	2010-S08-018	Urine	79	<=4	>16	>16		>16	>16	0.25
VII	2010-S08-023	Urine	73	<=4	>16	<=1		<=1	8	0.25
VII	2010-S08-025	Urine	24	<=4	<=2	<=1		<=1	<=2	<=0.06
VII	2010-S08-027	Urine	69	<=4	>16	<=1		<=1	8	<=0.06
VII	2010-S08-029	Urine	90	<=4	>16	2		<=1	>16	<=0.06
VII	2010-S08-032	Urine	71	<=4	>16	>16		4	>16	>2
VII	2010-S08-033	Urine	27	<=4	>16	<=1		<=1	<=2	0.12
VII	2010-S08-035	Urine	80	<=4	>16	8		16	>16	>2
VII	2010-S08-037	Urine	75	<=4	>16	<=1		<=1	4	<=0.06
VII	2010-S08-039	Blood	60	<=4	<=2	<=2		<=1	2	<=0.06
VII	2010-S08-044	Urine	48	<=4	>16	>16		>16	>16	>2
VII	2010-S08-045	Urine	77	>32	>16	>16		>16	>16	>2
VII	2010-S08-201	Other	41	<=4	4	<=1		<=1	<=2	<=0.06
VII	2010-S08-209	Blood	59	<=4	>16	<=2		<=1	8	0.25
VII	2010-S08-214	Blood	88	<=4	>16	8		16	>16	>2
VII	2010-S08-229	Blood	90	16	>16	>16		>16	>16	>2
VII	2010-S08-237	Blood	62	<=4	>16	>16		>16	>16	>2
VII	2010-E02-005	Urine	92	<=4	>16	<=1		<=1	<=2	<=0.06
VII	2010-E02-008	Urine	42	<=4	>16	<=1		<=1	<=2	<=0.06
VII	2010-E02-010	Urine	73	<=4	>16	<=1		<=1	4	<=0.06

VII	2010-E02-011	Urine	57	<=4	>16	<=1		<=1	4	<=0.06
VII	2010-E02-012	Urine	82	<=4	>16	<=1		<=1	8	<=0.06
VII	2010-E02-013	Urine	20	<=4	<=2	<=1		<=1	<=2	<=0.06
VII	2010-E02-014	Urine	72	<=4	>16	<=1		<=1	4	<=0.06
VII	2010-E02-018	Urine	49	<=4	>16	<=1		<=1	4	>2
VII	2010-E02-019	Urine	101	<=4	4	<=1		<=1	<=2	<=0.06
VII	2010-E02-021	Urine	80	<=4	<=2	<=1		<=1	<=2	<=0.06
VII	2010-E02-022	Urine	74	<=4	4	<=1		<=1	<=2	0.25
VII	2010-E02-026	Urine	58	<=4	>16	<=1		<=1	<=2	<=0.06
VII	2010-E02-027	Urine	72	<=4	>16	<=1		<=1	<=2	0.12
VII	2010-E02-031	Urine	0	<=4	<=2	<=1		<=1	<=2	<=0.06
VII	2010-E02-032	Urine	71	<=4	>16	<=1		<=1	4	<=0.06
VII	2010-E02-033	Urine	78	<=4	<=2	<=1		<=1	<=2	0.5
VII	2010-E02-034	Urine	82	8	>16	<=1		<=1	>16	0.12
VII	2010-E02-036	Urine	84	<=4	<=2	<=1		<=1	4	<=0.06
VII	2010-E02-038	Other	65	<=4	<=2	<=1		<=1	<=2	>2
VII	2010-E02-040	Urine	82	8	>16	>16		16	>16	>2
VII	2010-E02-042	Urine	87	<=4	>16	<=1		<=1	4	>2
VII	2010-E02-043	Urine	82	<=4	>16	<=1		<=1	4	0.12
VII	2010-E02-044	Blood	49	<=4	4	<=2		<=1	2	<=0.06
VII	2010-E02-045	Blood	60	<=4	>16	<=2		<=1	2	0.25
VII	2010-E02-046	Blood	68	<=4	>16	<=2		<=1	4	0.5
VII	2010-E02-047	Blood	76	<=4	>16	<=2		<=1	2	0.12
VII	2010-E02-049	Blood	73	<=4	4	<=2		<=1	2	1
VII	2010-E02-050	Urine	0	<=4	4	<=1		<=1	<=2	<=0.06
VII	2010-E02-201	Blood	52	<=4	<=2	<=2		<=1	2	<=0.06
VII	2010-N08-005	Urine	54	<=4	>16	<=1		<=1	<=2	>2
VII	2010-N08-006	Urine	74	<=4	>16	<=1		<=1	<=2	<=0.06
VII	2010-N08-007	Urine	32	<=4	4	<=1		<=1	<=2	<=0.06
VII	2010-N08-008	Urine	77	<=4	<=2	<=1		<=1	<=2	<=0.06
VII	2010-N08-016	Urine	21	<=4	>16	<=1		<=1	4	0.5
VII	2010-N08-020	Urine	74	<=4	>16	16		>16	>16	>2
VII	2010-N08-022	Urine	35	<=4	>16	<=1		<=1	4	<=0.06
VII	2010-N08-023	Urine	28	<=4	>16	16		16	>16	<=0.06
VII	2010-N08-025	Urine	3	<=4	>16	<=1		<=1	8	0.25
VII	2010-N08-027	Urine	31	<=4	>16	<=1		<=1	4	<=0.06
VII	2010-N08-028	Urine	84	<=4	>16	<=1		<=1	4	>2
VII	2010-N08-030	Urine	21	<=4	<=2	<=1		<=1	<=2	0.25
VII	2010-N08-031	Urine	77	<=4	>16	<=1		<=1	<=2	>2
VII	2010-N08-032	Urine	85	8	16	<=1		<=1	4	<=0.06
VII	2010-N08-035	Urine	22	<=4	>16	<=1		<=1	4	>2
VII	2010-N08-036	Urine	0	<=4	>16	<=1		<=1	4	<=0.06
VII	2010-N08-037	Urine	73	<=4	>16	<=1		<=1	<=2	>2
VII	2010-N08-038	Urine	30	8	>16	<=1		<=1	<=2	<=0.06
VII	2010-N08-048	Urine	55	<=4	<=2	<=1		<=1	<=2	0.12
VII	2010-N08-050	Urine	26	<=4	>16	<=1		<=1	<=2	>2
VII	2010-S09-008	Blood	51	<=4	>16	<=2		<=1	4	<=0.06
VII	2010-S09-009	Urine	79	<=4	>16	16		4	>16	>2
VII	2010-S09-010	Urine	54	<=4	>16	16		>16	>16	2
VII	2010-S09-016	Urine	51	<=4	>16	8		16	>16	>2
VII	2010-S09-018	Urine	75	16	<=2	<=1		<=1	<=2	<=0.06
VII	2010-S09-021	Urine	52	8	<=2	<=1		<=1	<=2	0.12
VII	2010-S09-022	Blood	83	<=4	<=2	<=2		<=1	2	<=0.06
VII	2010-S09-023	Blood	47	<=4	<=2	<=2		<=1	2	<=0.06
VII	2010-S09-024	Blood	72	<=4	>16	16		>16	>16	>2
VII	2010-S09-034	Urine	49	<=4	>16	4		8	>16	1
VII	2010-S09-035	Urine	28	<=4	>16	<=1		<=1	16	<=0.06
VII	2010-S09-036	Urine	55	<=4	<=2	<=1		<=1	<=2	<=0.06
VII	2010-S09-040	Urine	97	<=4	<=2	<=1		<=1	<=2	<=0.06
VII	2010-S09-041	Blood	73	<=4	4	<=2		<=1	2	<=0.06
VII	2010-S09-042	Blood	55	<=4	>16	8		16	>16	<=0.06

VII	2010-S09-043	Blood	53	<=4	>16	<=2		<=1	4	<=0.06
VII	2010-S09-044	Blood	70	<=4	>16	<=2		<=1	4	<=0.06
VII	2010-S09-194	Urine	16	<=4	4	<=1		<=1	4	<=0.06
VII	2010-S09-204	Blood	76	<=4	<=2	<=2		<=1	<=1	<=0.06
VII	2010-S09-205	Blood	68	<=4	<=2	<=2		<=1	2	<=0.06
VII	2010-S09-209	Blood	84	<=4	<=2	<=2		<=1	2	<=0.06
VII	2010-S09-210	Blood	74	<=4	<=2	<=2		<=1	2	<=0.06
VII	2010-S09-212	Blood	60	<=4	>16	<=2		<=1	2	0.5
VII	2010-S09-218	Blood	89	<=4	>16	<=2		<=1	4	0.25
VII	2010-S09-222	Blood	57	<=4	>16	<=2		<=1	4	<=0.06
VII	2010-S09-223	Blood	88	<=4	>16	<=2		<=1	4	<=0.06
VII	2010-S09-231	Blood	69	<=4	>16	<=2		<=1	4	<=0.06
VII	2010-S09-232	Blood	73	<=4	>16	16		>16	>16	>2
VII	2010-S09-239	Blood	59	8	>16	16		>16	>16	>2
VII	2010-S09-241	Blood	54	<=4	4	<=2		<=1	2	<=0.06
VII	2010-S09-243	Blood	52	8	>16	<=2		<=1	4	<=0.06
VII	2010-S09-246	Blood	71	<=4	<=2	<=2		<=1	<=1	<=0.06
VII	2010-S09-247	Blood	73	<=4	<=2	<=2		<=1	<=1	<=0.06
VII	2010-E03-002	Urine	81	<=4	>16	16		>16	>16	>2
VII	2010-E03-004	Urine	85	<=4	4	<=1		<=1	<=2	<=0.06
VII	2010-E03-010	Urine	53	<=4	<=2	<=1		<=1	<=2	<=0.06
VII	2010-E03-021	Urine	78	<=4	<=2	<=1		<=1	<=2	<=0.06
VII	2010-E03-025	Urine	50	<=4	>16	<=1		<=1	<=2	<=0.06
VII	2010-E03-026	Urine	26	<=4	<=2	<=1		<=1	<=2	<=0.06
VII	2010-E03-032	Urine	66	<=4	>16	>16		>16	>16	>2
VII	2010-E03-033	Urine	35	<=4	4	<=1		<=1	<=2	<=0.06
VII	2010-E03-037	Urine	80	<=4	4	<=1		<=1	<=2	<=0.06
VII	2010-E03-039	Urine	47	8	>16	<=2		<=1	16	>2
VII	2010-E03-040	Urine	62	<=4	>16	<=1		<=1	8	0.5
VII	2010-E03-045	Urine	38	<=4	>16	<=2		<=1	16	<=0.06
VII	2010-E03-046	Urine	59	<=4	>16	<=2		<=1	4	<=0.06
VII	2010-E03-048	Urine	74	<=4	>16	<=1		<=1	4	<=0.06
VII	2010-E03-210	Blood	42	<=4	>16	<=2		<=1	2	0.12
VII	2010-E03-215	Blood	65	8	8	<=2		<=1	8	<=0.06
VII	2010-E03-217	Blood	52	<=4	>16	16		4	>16	>2
VII	2010-E03-222	Blood	78	<=4	>16	8		16	>16	0.12
VII	2010-E03-227	Blood	84	<=4	>16	<=2		<=1	16	>2
VII	2010-E03-240	Blood	73	<=4	>16	<=2		<=1	16	0.5
VII	2010-C06-006	Urine	58	<=4	4	<=1		<=1	<=2	<=0.06
VII	2010-C06-009	Urine	0	<=4	>16	<=1		<=1	8	0.12
VII	2010-C06-010	Urine	65	<=4	>16	<=1		<=1	8	>2
VII	2010-C06-014	Urine	83	<=4	>16	8		16	>16	1
VII	2010-C06-015	Urine	86	<=4	>16	>16		8	>16	>2
VII	2010-C06-016	Urine	36	<=4	>16	<=1		<=1	4	<=0.06
VII	2010-C06-021	Urine	71	8	>16	16		>16	>16	>2
VII	2010-C06-022	Urine	69	<=4	>16	<=1		<=1	4	<=0.06
VII	2010-C06-025	Other	48	<=4	>16	<=1		<=1	8	2
VII	2010-C06-027	Urine	55	<=4	>16	<=1		<=1	16	0.25
VII	2010-C06-028	Urine	67	<=4	>16	4		16	>16	>2
VII	2010-C06-030	Urine	74	<=4	>16	>16		16	>16	>2
VII	2010-C06-034	Urine	63	<=4	<=2	<=1		<=1	<=2	<=0.06
VII	2010-C06-037	Urine	69	<=4	>16	<=1		2	>16	>2
VII	2010-C06-043	Other	52	<=4	4	<=1		<=1	<=2	<=0.06
VII	2010-C06-044	Urine	72	<=4	4	<=1		<=1	<=2	<=0.06
VII	2010-C06-045	Urine	45	<=4	>16	<=1		<=1	8	<=0.06
VII	2010-C06-046	Urine	59	<=4	>16	<=1		<=1	4	>2
VII	2010-C06-049	Other	32	<=4	>16	<=1		<=1	4	<=0.06
VII	2010-C06-057	Other	52	<=4	>16	<=1		<=1	8	>2
VII	2010-C06-174	Urine	91	<=4	>16	16		4	>16	>2
VII	2010-C06-177	Urine	82	<=4	>16	>16		>16	>16	>2
VII	2010-C07-008	Other	39	<=4	>16	4		>16	>16	>2

VII	2010-C07-014	Urine	1	<=4	>16	4		8	>16	<=0.06
VII	2010-C07-015	Urine	81	<=4	<=2	<=1		<=1	<=2	<=0.06
VII	2010-C07-017	Urine	25	<=4	>16	<=1		<=1	>16	<=0.06
VII	2010-C07-021	Urine	44	<=4	>16	<=1		<=1	8	>2
VII	2010-C07-022	Urine	45	<=4	>16	<=1		<=1	<=2	<=0.06
VII	2010-C07-024	Urine	37	<=4	>16	<=1		<=1	<=2	<=0.06
VII	2010-C07-025	Urine	81	<=4	>16	<=1		<=1	4	0.25
VII	2010-C07-031	Other	47	<=4	>16	<=1		<=1	4	>2
VII	2010-C07-033	Urine	82	<=4	>16	<=1		<=1	<=2	<=0.06
VII	2010-C07-036	Other	38	<=4	4	<=1		<=1	4	<=0.06
VII	2010-C07-037	Urine	80	<=4	>16	4		2	>16	>2
VII	2010-C07-039	Other	35	<=4	4	<=1		<=1	<=2	<=0.06
VII	2010-C07-041	Urine	70	<=4	8	<=1		2	4	<=0.06
VII	2010-C07-043	Urine	54	8	>16	<=1		<=1	16	>2
VII	2010-C07-045	Blood	76	<=4	>16	>16		16	>16	>2
VII	2010-C07-050	Urine	59	<=4	<=2	<=1		<=1	<=2	<=0.06
VII	2010-C07-202	Blood	85	<=4	>16	<=2		<=1	4	<=0.06
VII	2010-C07-210	Blood	67	<=4	4	<=2		<=1	4	<=0.06
VII	2010-C07-211	Blood	64	<=4	4	<=2		<=1	2	0.12
VII	2010-C07-212	Blood	66	<=4	>16	>16		>16	>16	>2
VII	2010-C07-224	Blood	78	<=4	<=2	<=2		<=1	2	>2
VII	2010-C07-226	Other	58	<=4	>16	<=1		<=1	>16	>2
VII	2010-C07-234	Blood	56	<=4	>16	4		8	>16	<=0.06
VII	2010-C07-238	Other	62	<=4	>16	<=1		<=1	4	>2
VII	2010-C07-243	Blood	84	<=4	>16	<=2		<=1	4	0.25
VII	2010-N16-002	Urine	61	<=4	4	<=1		<=1	4	<=0.06
VII	2010-N16-010	Urine	61	<=4	>16	<=1		<=1	<=2	>2
VII	2010-N16-013	Urine	66	<=4	>16	<=1		<=1	16	>2
VII	2010-N16-014	Blood	71	<=4	>16	<=2		<=1	2	<=0.06
VII	2010-N16-016	Blood	81	<=4	<=2	<=2		<=1	<=1	<=0.06
VII	2010-N16-019	Urine	58	<=4	>16	<=1		<=1	4	0.25
VII	2010-N16-023	Urine	0	<=4	>16	<=1		<=1	<=2	0.5
VII	2010-N16-026	Urine	89	<=4	>16	<=1		<=1	4	>2
VII	2010-N16-029	Other	18	<=4	>16	<=1		<=1	4	0.25
VII	2010-N16-031	Urine	2	<=4	>16	8		2	>16	0.25
VII	2010-N16-033	Urine	42	<=4	>16	4		<=1	>16	>2
VII	2010-N16-034	Urine	59	<=4	>16	<=1		<=1	<=2	0.12
VII	2010-N16-038	Blood	79	<=4	<=2	<=2		<=1	<=1	2
VII	2010-N16-041	Blood	85	<=4	>16	<=2		<=1	16	<=0.06
VII	2010-N16-201	Blood	81	<=4	>16	<=2		<=1	2	<=0.06
VII	2010-N16-202	Blood	80	<=4	>16	<=2		<=1	16	<=0.06
VII	2010-N16-203	Blood	89	<=4	<=2	<=2		<=1	2	<=0.06
VII	2010-N16-204	Blood	68	<=4	>16	<=2		<=1	4	>2
VII	2010-N16-207	Blood	71	<=4	<=2	<=2		<=1	4	<=0.06
VII	2010-N16-208	Blood	86	<=4	>16	<=2		<=1	16	<=0.06
VII	2010-N16-212	Blood	79	<=4	>16	<=2		<=1	2	<=0.06
VII	2010-N16-213	Blood	74	<=4	>16	<=2		<=1	4	<=0.06
VII	2010-N16-215	Other	75	<=4	>16	<=1		<=1	<=2	<=0.06
VII	2010-N16-218	Blood	56	<=4	>16	<=2		<=1	4	0.25
VII	2010-N16-221	Blood	87	<=4	<=2	<=2		<=1	<=1	<=0.06
VII	2010-N16-229	Blood	70	8	>16	16		2	>16	>2
VII	2010-N16-232	Blood	88	<=4	>16	16		>16	>16	0.5
VII	2010-N16-238	Blood	68	<=4	<=2	<=2		<=1	2	<=0.06
VII	2010-N16-244	Blood	57	<=4	>16	8		>16	>16	<=0.06
VII	2010-N16-245	Blood	88	<=4	<=2	<=2		<=1	2	<=0.06
VII	2010-N16-246	Blood	85	<=4	<=2	<=2		<=1	<=1	<=0.06
VII	2010-N17-002	Urine	64	<=4	<=2	<=1		<=1	<=2	<=0.06
VII	2010-N17-004	Urine	0	<=4	>16	<=1		<=1	<=2	<=0.06
VII	2010-N17-005	Urine	0	<=4	>16	<=1		<=1	<=2	0.25
VII	2010-N17-006	Urine	1	<=4	>16	<=1		<=1	<=2	<=0.06
VII	2010-N17-013	Urine	33	<=4	4	<=1		<=1	<=2	<=0.06

VII	2010-N17-017	Urine	32	<=4	<=2	<=1		<=1	<=2	<=0.06
VII	2010-N17-022	Urine	7	<=4	<=2	<=1		<=1	<=2	0.25
VII	2010-N17-024	Urine	3	<=4	4	<=1		<=1	<=2	<=0.06
VII	2010-N17-026	Urine	82	<=4	8	<=1		<=1	<=2	<=0.06
VII	2010-N17-032	Urine	54	<=4	>16	<=1		<=1	<=2	0.5
VII	2010-N17-033	Urine	2	<=4	4	<=1		<=1	<=2	<=0.06
VII	2010-N17-043	Urine	84	<=4	<=2	<=1		<=1	<=2	<=0.06
VII	2010-N17-044	Urine	71	<=4	>16	<=1		<=1	8	<=0.06
VII	2010-N17-045	Urine	36	<=4	>16	<=1		<=1	4	<=0.06
VII	2010-N17-047	Urine	89	<=4	>16	4		8	>16	0.5
VII	2010-N17-048	Urine	89	<=4	>16	<=1		<=1	<=2	0.25
VII	2010-N17-049	Urine	25	<=4	>16	<=1		<=1	8	>2
VII	2010-N17-205	Blood	91	<=4	>16	<=2		<=1	8	<=0.06
VII	2010-N17-213	Blood	58	<=4	>16	<=2		<=1	2	<=0.06
VII	2010-N17-227	Blood	88	<=4	>16	>16		>16	>16	>2
VII	2010-N17-228	Blood	77	<=4	>16	<=2		<=1	8	<=0.06
VII	2010-N17-229	Blood	71	<=4	>16	<=2		<=1	4	<=0.06
VII	2010-N17-234	Blood	53	8	>16	>16		>16	>16	>2
VII	2010-N17-240	Blood	73	<=4	>16	8		4	>16	>2
VII	2010-N19-001	Urine	33	<=4	>16	<=1		<=1	16	>2
VII	2010-N19-002	Urine	69	<=4	<=2	<=1		<=1	<=2	<=0.06
VII	2010-N19-003	Urine	29	<=4	>16	<=1		<=1	4	>2
VII	2010-N19-004	Urine	85	<=4	>16	>16		>16	>16	>2
VII	2010-N19-006	Urine	43	<=4	>16	<=1		<=1	8	>2
VII	2010-N19-007	Urine	80	<=4	>16	4		16	>16	<=0.06
VII	2010-N19-008	Urine	37	<=4	>16	<=1		<=1	<=2	<=0.06
VII	2010-N19-011	Other	60	<=4	<=2	<=1		<=1	<=2	0.12
VII	2010-N19-014	Urine	53	<=4	<=2	<=1		<=1	<=2	<=0.06
VII	2010-N19-015	Urine	77	<=4	>16	>16		16	>16	>2
VII	2010-N19-016	Urine	81	8	>16	>16		2	>16	>2
VII	2010-N19-017	Urine	83	<=4	<=2	<=1		<=1	<=2	<=0.06
VII	2010-N19-018	Other	61	<=4	>16	<=1		<=1	4	0.5
VII	2010-N19-025	Urine	50	<=4	<=2	<=1		<=1	<=2	<=0.06
VII	2010-N19-033	Urine	39	<=4	<=2	<=1		<=1	<=2	<=0.06
VII	2010-N19-034	Urine	78	<=4	>16	<=1		<=1	<=2	<=0.06
VII	2010-N19-035	Urine	66	<=4	<=2	<=1		<=1	<=2	<=0.06
VII	2010-N19-039	Urine	57	<=4	>16	<=1		<=1	<=2	<=0.06
VII	2010-N19-040	Urine	76	<=4	4	<=1		<=1	<=2	>2
VII	2010-N19-043	Urine	85	<=4	<=2	<=1		<=1	<=2	<=0.06
VII	2010-N19-044	Blood	58	<=4	4	<=2		<=1	2	<=0.06
VII	2010-N19-046	Urine	55	<=4	>16	<=1		<=1	16	>2
VII	2010-N19-047	Urine	45	<=4	<=2	<=1		<=1	<=2	<=0.06
VII	2010-N19-048	Urine	22	<=4	4	<=1		<=1	<=2	0.25
VII	2010-N19-050	Urine	52	<=4	>16	<=1		<=1	<=2	<=0.06
VII	2010-C08-004	Urine	47	<=4	<=2	<=1		<=1	<=2	<=0.06
VII	2010-C08-005	Urine	53	<=4	>16	<=1		<=1	<=2	>2
VII	2010-C08-006	Urine	22	<=4	4	<=1		<=1	<=2	<=0.06
VII	2010-C08-007	Urine	50	<=4	>16	<=1		<=1	>16	<=0.06
VII	2010-C08-008	Urine	66	<=4	>16	<=1		<=1	8	0.12
VII	2010-C08-013	Urine	75	<=4	>16	<=1		<=1	4	>2
VII	2010-C08-017	Urine	28	<=4	>16	<=1		<=1	<=2	0.5
VII	2010-C08-018	Urine	82	<=4	4	<=1		<=1	<=2	<=0.06
VII	2010-C08-019	Urine	78	<=4	<=2	<=1		<=1	<=2	<=0.06
VII	2010-C08-020	Urine	42	<=4	4	<=1		<=1	<=2	<=0.06
VII	2010-C08-021	Urine	20m	<=4	>16	<=1		<=1	<=2	0.12
VII	2010-C08-022	Urine	50	8	>16	<=1		<=1	8	1
VII	2010-C08-024	Urine	53	<=4	>16	<=1		<=1	<=2	0.5
VII	2010-C08-027	Other	59	<=4	>16	<=1		<=1	<=2	<=0.06
VII	2010-C08-028	Other	73	<=4	16	<=1		<=1	16	<=0.06
VII	2010-C08-030	Urine	57	<=4	>16	<=1		<=1	>16	<=0.06
VII	2010-C08-032	Urine	51	<=4	>16	>16		>16	>16	>2

VII	2010-C08-033	Urine	75	<=4	4	<=1		<=1	<=2	<=0.06
VII	2010-C08-034	Urine	84	<=4	>16	<=1		<=1	4	>2
VII	2010-C08-035	Urine	57	<=4	>16	<=1		<=1	8	<=0.06
VII	2010-C08-038	Other	19	<=4	>16	<=1		<=1	8	<=0.06
VII	2010-C08-040	Urine	28	<=4	>16	<=1		<=1	<=2	<=0.06
VII	2010-C08-193	Urine	6	<=4	4	<=1		<=1	<=2	<=0.06
VII	2010-C08-200	Urine	3	<=4	>16	<=1		<=1	8	<=0.06
VII	2010-C08-209	Blood	60	<=4	<=2	<=2		<=1	2	<=0.06
VII	2010-C08-213	Blood	73	<=4	>16	<=2		<=1	2	<=0.06
VII	2010-C08-216	Blood	21	<=4	>16	<=2		<=1	8	0.25
VII	2010-C08-226	Blood	51	<=4	>16	<=2		<=1	4	<=0.06
VII	2010-C08-232	Blood	58	<=4	>16	<=2		<=1	2	0.12
VII	2010-C08-241	Blood	78	<=4	>16	<=2		4	>16	>2
VII	2010-S11-005	Urine	1	<=4	>16	<=1		<=1	<=2	<=0.06
VII	2010-S11-007	Urine	56	<=4	>16	<=1		<=1	4	<=0.06
VII	2010-S11-008	Urine	72	<=4	4	<=1		<=1	<=2	<=0.06
VII	2010-S11-009	Urine	85	<=4	<=2	<=1		<=1	<=2	<=0.06
VII	2010-S11-010	Urine	64	<=4	>16	<=1		<=1	<=2	<=0.06
VII	2010-S11-011	Urine	71	<=4	4	<=1		<=1	<=2	<=0.06
VII	2010-S11-012	Blood	64	<=4	<=2	<=2		<=1	2	<=0.06
VII	2010-S11-015	Urine	67	<=4	>16	<=1		<=1	4	<=0.06
VII	2010-S11-016	Urine	14	<=4	>16	<=1		2	>16	>2
VII	2010-S11-017	Urine	71	<=4	>16	<=1		<=1	4	>2
VII	2010-S11-018	Urine	73	<=4	4	<=1		<=1	4	<=0.06
VII	2010-S11-021	Urine	87	<=4	>16	<=1		<=1	<=2	>2
VII	2010-S11-031	Blood	62	8	>16	<=2		<=1	8	<=0.06
VII	2010-S11-033	Urine	72	<=4	<=2	<=1		<=1	<=2	<=0.06
VII	2010-S11-039	Urine	75	<=4	>16	4		8	>16	>2
VII	2010-S11-042	Urine	78	<=4	>16	<=1		<=1	4	<=0.06
VII	2010-S11-045	Urine	83	8	>16	>16		16	>16	>2
VII	2010-S11-046	Urine	0	<=4	>16	<=1		<=1	4	>2
VII	2010-S11-048	Urine	66	<=4	>16	<=1		<=1	>16	<=0.06
VII	2010-S11-201	Blood	77	<=4	4	<=2		<=1	2	0.12
VII	2010-S11-205	Blood	70	32	>16	16		<=1	4	>2
VII	2010-S11-207	Blood	89	<=4	>16	<=2		<=1	16	<=0.06
VII	2010-S11-208	Blood	87	<=4	>16	<=2		<=1	8	<=0.06
VII	2010-S11-209	Blood	45	<=4	>16	<=2		<=1	4	0.25
VII	2010-S11-210	Blood	59	<=4	>16	<=2		<=1	8	<=0.06
VII	2010-S11-211	Blood	36	16	>16	<=2		<=1	8	>2
VII	2010-S11-219	Blood	48	<=4	>16	<=2		<=1	2	<=0.06
VII	2010-S11-226	Blood	77	>32	>16	>16		>16	>16	>2
VII	2010-S11-231	Blood	59	<=4	>16	<=2		<=1	8	0.12
VII	2010-S11-234	Blood	53	8	>16	>16		16	>16	>2
VII	2010-S11-237	Blood	79	<=4	<=2	<=2		<=1	2	<=0.06
VII	2010-S11-242	Blood	33	<=4	>16	<=2		<=1	2	0.12
VII	2010-N21-003	Blood	62	<=4	>16	4		8	>16	>2
VII	2010-N21-005	Blood	73	<=4	>16	<=2		<=1	2	<=0.06
VII	2010-N21-009	Other	71	<=4	>16	8		16	>16	>2
VII	2010-N21-013	Urine	31	<=4	>16	<=1		<=1	<=2	<=0.06
VII	2010-N21-019	Urine	0	<=4	4	<=1		<=1	<=2	<=0.06
VII	2010-N21-022	Urine	47	<=4	<=2	<=1		<=1	<=2	<=0.06
VII	2010-N21-026	Blood	53	<=4	<=2	<=2		<=1	2	<=0.06
VII	2010-N21-028	Blood	82	<=4	>16	<=2		<=1	8	0.25
VII	2010-N21-029	Blood	74	<=4	4	<=2		<=1	2	<=0.06
VII	2010-N21-030	Blood	40	<=4	>16	<=2		<=1	16	<=0.06
VII	2010-N21-031	Blood	65	<=4	>16	<=2		<=1	4	0.25
VII	2010-N21-032	Blood	64	8	4	<=2		<=1	2	<=0.06
VII	2010-N21-033	Blood	83	<=4	4	<=2		<=1	2	<=0.06
VII	2010-N21-034	Blood	18	<=4	>16	<=2		<=1	4	<=0.06
VII	2010-N21-035	Blood	74	<=4	>16	<=2		<=1	2	0.12
VII	2010-N21-036	Blood	37	<=4	<=2	<=2		<=1	2	<=0.06

VII	2010-N21-193	Urine	0	<=4	>16	<=1		<=1	<=2	<=0.06
VII	2010-N21-216	Blood	87	<=4	>16	<=2		<=1	16	<=0.06
VII	2010-N21-223	Blood	49	<=4	<=2	<=2		<=1	<=1	<=0.06
VII	2010-N21-225	Blood	51	<=4	>16	<=2		<=1	4	0.12
VII	2010-N21-228	Blood	79	<=4	>16	<=2		<=1	8	<=0.06
VII	2010-N21-237	Blood	66	<=4	>16	<=2		<=1	2	0.12
VII	2010-N21-240	Blood	43	<=4	4	<=2		<=1	2	0.5
VII	2010-N21-241	Blood	59	<=4	<=2	<=2		<=1	<=1	<=0.06
VII	2010-N21-247	Blood	81	<=4	>16	<=2		<=1	2	0.12
VII	2010-N21-249	Blood	69	<=4	<=2	<=2		<=1	2	<=0.06
VII	2010-N21-250	Blood	76	<=4	>16	<=2		<=1	>16	>2
VII	2010-C09-001	Urine	57	<=4	>16	<=1		<=1	4	0.25
VII	2010-C09-002	Urine	69	<=4	>16	<=1		<=1	>16	>2
VII	2010-C09-004	Urine	38	<=4	>16	<=1		<=1	<=2	0.12
VII	2010-C09-009	Urine	21	<=4	>16	<=1		<=1	<=2	<=0.06
VII	2010-C09-013	Urine	79	<=4	<=2	<=1		<=1	<=2	<=0.06
VII	2010-C09-014	Other		<=4	>16	<=1		<=1	<=2	<=0.06
VII	2010-C09-015	Urine	48	<=4	>16	<=1		<=1	<=2	0.12
VII	2010-C09-017	Blood	91	<=4	<=2	<=2		<=1	2	<=0.06
VII	2010-C09-020	Blood	54	<=4	>16	<=2		<=1	4	<=0.06
VII	2010-C09-023	Blood	55	<=4	4	<=2		<=1	2	<=0.06
VII	2010-C09-024	Urine	48	<=4	>16	<=1		<=1	4	>2
VII	2010-C09-026	Other	59	<=4	>16	<=1		<=1	<=2	<=0.06
VII	2010-C09-028	Urine	2	<=4	>16	<=1		<=1	4	<=0.06
VII	2010-C09-034	Urine	79	<=4	>16	<=1		<=1	16	>2
VII	2010-C09-035	Urine	64	<=4	>16	<=1		<=1	4	0.25
VII	2010-C09-045	Urine	92	8	>16	>16		>16	>16	>2
VII	2010-C09-048	Urine	1	<=4	4	<=1		<=1	<=2	<=0.06
VII	2010-C09-049	Other	60	<=4	>16	<=1		<=1	<=2	<=0.06
VII	2010-C09-050	Urine	32	<=4	4	<=1		<=1	<=2	<=0.06
VII	2010-C09-211	Blood	82	<=4	<=2	<=2		<=1	<=1	<=0.06
VII	2010-C09-237	Blood	58	<=4	>16	<=2		<=1	8	0.25
VII	2010-C09-238	Blood	82	<=4	4	<=2		<=1	2	0.12
VII	2010-C09-246	Blood	36	<=4	>16	<=2		<=1	4	<=0.06
VII	2010-C09-248	Blood	81	<=4	>16	<=2		<=1	16	<=0.06
VII	2010-C10-001	Blood	64	<=4	<=2	<=2		<=1	2	0.25
VII	2010-C10-002	Urine	78	<=4	4	<=1		<=1	<=2	<=0.06
VII	2010-C10-005	Urine	64	<=4	>16	<=1		<=1	<=2	0.12
VII	2010-C10-006	Urine	35	<=4	4	<=1		<=1	<=2	0.12
VII	2010-C10-007	Urine	77	<=4	8	<=1		<=1	<=2	<=0.06
VII	2010-C10-011	Blood	74	<=4	>16	<=2		<=1	4	0.25
VII	2010-C10-012	Urine	70	<=4	>16	8		4	>16	>2
VII	2010-C10-013	Blood	66	<=4	<=2	<=2		<=1	2	<=0.06
VII	2010-C10-020	Urine	58	<=4	>16	>16		>16	>16	>2
VII	2010-C10-021	Blood	78	<=4	>16	<=2		<=1	4	<=0.06
VII	2010-C10-022	Blood	86	16	>16	16		>16	>16	>2
VII	2010-C10-023	Urine	87	<=4	>16	<=1		<=1	8	0.25
VII	2010-C10-032	Urine	51	<=4	>16	<=1		<=1	<=2	0.12
VII	2010-C10-034	Urine	38	<=4	>16	<=1		<=1	4	<=0.06
VII	2010-C10-035	Urine	72	>32	>16	>16		>16	>16	>2
VII	2010-C10-039	Urine	74	<=4	4	<=1		<=1	<=2	<=0.06
VII	2010-C10-040	Urine	27	<=4	4	<=1		<=1	<=2	<=0.06
VII	2010-C10-041	Urine	53	<=4	>16	<=1		<=1	4	<=0.06
VII	2010-C10-042	Urine	54	<=4	4	<=1		<=1	<=2	<=0.06
VII	2010-C10-043	Urine	46	<=4	<=2	<=1		<=1	<=2	<=0.06
VII	2010-C10-184	Urine	1	<=4	>16	<=1		<=1	4	<=0.06
VII	2010-C10-194	Urine	7	<=4	>16	<=1		<=1	4	>2
VII	2010-C10-198	Urine	1	<=4	>16	<=1		<=1	<=2	<=0.06
VII	2010-C10-208	Blood	68	<=4	<=2	<=2		<=1	2	<=0.06
VII	2010-C10-209	Blood	46	8	>16	16		>16	>16	0.12
VII	2010-C10-210	Blood	27	<=4	>16	<=2		<=1	8	<=0.06

VII	2010-C10-215	Blood	92	<=4	4	<=2		<=1	2	<=0.06
VII	2010-C10-216	Blood	74	<=4	>16	<=2		<=1	4	<=0.06
VII	2010-C10-222	Other	49	<=4	>16	>16		8	>16	0.5
VII	2010-C10-230	Blood	58	<=4	4	<=2		<=1	2	<=0.06
VII	2010-C10-249	Blood	88	<=4	>16	<=2		<=1	8	<=0.06
VII	2010-E04-002	Blood	36	<=4	4	<=2		<=1	2	<=0.06
VII	2010-E04-004	Blood	91	<=4	>16	>16		>16	>16	>2
VII	2010-E04-005	Blood	74	<=4	>16	<=2		<=1	2	0.12
VII	2010-E04-014	Blood	67	<=4	>16	<=2		<=1	4	<=0.06
VII	2010-E04-016	Blood	88	<=4	>16	<=2		<=1	4	0.25
VII	2010-E04-019	Blood	59	<=4	<=2	<=2		<=1	2	<=0.06
VII	2010-E04-020	Blood	70	<=4	<=2	<=2		<=1	2	<=0.06
VII	2010-E04-021	Blood	90	<=4	4	<=2		<=1	2	<=0.06
VII	2010-E04-022	Blood	88	<=4	4	<=2		<=1	2	<=0.06
VII	2010-E04-024	Blood	64	<=4	>16	<=2		<=1	4	<=0.06
VII	2010-E04-029	Blood	73	<=4	>16	<=2		<=1	4	<=0.06
VII	2010-E04-032	Other	79	<=4	>16	<=1		<=1	<=2	<=0.06
VII	2010-E04-037	Blood	70	<=4	>16	<=2		<=1	8	<=0.06
VII	2010-E04-041	Blood	74	<=4	>16	<=2		<=1	4	<=0.06
VII	2010-E04-044	Blood	77	<=4	4	<=2		<=1	4	<=0.06
VIII	2012-S01-001	Urine	69	<=4	>16	4		<=1	>16	>2
VIII	2012-S01-002	Urine	51	8	>16	>16		>16	>16	>2
VIII	2012-S01-003	Urine	62	<=4	>16	>16		16	>16	<=0.06
VIII	2012-S01-004	Urine	84	8	>16	<=1		<=1	4	>2
VIII	2012-S01-005	Urine	50	<=4	>16	<=1		<=1	<=2	<=0.06
VIII	2012-S01-007	Urine	76	<=4	>16	<=1		<=1	<=2	<=0.06
VIII	2012-S01-009	Urine	23	<=4	>16	<=1		<=1	<=2	<=0.06
VIII	2012-S01-011	Urine	56	<=4	<=2	<=1		<=1	<=2	<=0.06
VIII	2012-S01-012	Urine	52	<=4	<=2	<=1		<=1	<=2	<=0.06
VIII	2012-S01-014	Urine	76	<=4	>16	<=1		<=1	16	<=0.06
VIII	2012-S01-015	Urine	39	<=4	<=2	<=1		<=1	<=2	<=0.06
VIII	2012-S01-017	Urine	21	<=4	>16	<=1		<=1	4	<=0.06
VIII	2012-S01-019	Urine	69	<=4	>16	16		16	>16	>2
VIII	2012-S01-022	Urine	76	<=4	>16	<=1		<=1	<=2	<=0.06
VIII	2012-S01-024	Other	60	<=4	<=2	<=1		<=1	<=2	<=0.06
VIII	2012-S01-027	Urine	39	<=4	<=2	<=1		<=1	<=2	<=0.06
VIII	2012-S01-028	Urine	50	<=4	>16	<=1		<=1	4	>2
VIII	2012-S01-029	Urine	75	<=4	<=2	<=1		<=1	<=2	<=0.06
VIII	2012-S01-030	Urine	23	<=4	>16	<=1		<=1	<=2	<=0.06
VIII	2012-S01-031	Urine	47	<=4	>16	>16		>16	>16	0.12
VIII	2012-S01-032	Urine	82	<=4	<=2	<=1		<=1	<=2	<=0.06
VIII	2012-S01-034	Urine	0	<=4	>16	4		<=1	>16	>2
VIII	2012-S01-035	Urine	64	<=4	<=2	<=1		<=1	<=2	<=0.06
VIII	2012-S01-038	Urine	70	<=4	4	<=1		<=1	<=2	<=0.06
VIII	2012-S01-041	Urine	27	<=4	>16	<=1		<=1	<=2	<=0.06
VIII	2012-S01-042	Urine	63	<=4	>16	<=1		<=1	4	0.25
VIII	2012-S01-043	Urine	64	8	>16	<=1		<=1	4	0.12
VIII	2012-S01-046	Urine	38	<=4	>16	>16		16	>16	>2
VIII	2012-S01-050	Urine	86	<=4	>16	8		<=1	>16	>2
VIII	2012-S01-200	Urine	0	<=4	>16	4		<=1	>16	>2
VIII	2012-S01-208	Blood	69	<=4	>16	<=1		<=1	4	1
VIII	2012-S01-214	Blood	75	<=4	>16	<=1		<=1	4	<=0.06
VIII	2012-S01-232	Blood	62	<=4	8	<=1		<=1	4	<=0.06
VIII	2012-S01-233	Blood	71	<=4	<=2	<=1		<=1	<=2	<=0.06
VIII	2012-S01-236	Blood	24	<=4	>16	<=1		<=1	4	0.5
VIII	2012-S01-238	Blood	86	<=4	>16	16		16	>16	>2
VIII	2012-S01-240	Blood	62	<=4	>16	4		2	>16	<=0.06
VIII	2012-C01-001	Urine	81	<=4	>16	16		>16	>16	2
VIII	2012-C01-002	Urine	63	<=4	>16	8		2	>16	>2
VIII	2012-C01-003	Urine	66	<=4	>16	8		4	>16	>2
VIII	2012-C01-011	Other	70	<=4	>16	>16		>16	>16	>2

VIII	2012-C01-012	Urine	51	<=4	>16	4		<=1	>16	>2
VIII	2012-C01-013	Urine	84	<=4	>16	8		<=1	>16	<=0.06
VIII	2012-C01-015	Urine	77	<=4	>16	>16		16	>16	>2
VIII	2012-C01-016	Urine	81	<=4	>16	4		<=1	>16	>2
VIII	2012-C01-017	Urine	83	<=4	>16	>16		16	>16	>2
VIII	2012-C01-018	Urine	29	<=4	>16	>16		>16	>16	>2
VIII	2012-C01-019	Urine	22	<=4	>16	>16		>16	>16	>2
VIII	2012-C01-020	Urine	31	<=4	>16	8		8	>16	>2
VIII	2012-C01-021	Urine	1	<=4	>16	<=1		<=1	>16	1
VIII	2012-C01-023	Urine	75	<=4	>16	>16		16	>16	>2
VIII	2012-C01-024	Urine	89	>32	>16	>16		>16	>16	>2
VIII	2012-C01-025	Blood	49	<=4	>16	16		2	>16	>2
VIII	2012-C01-026	Urine	60	<=4	>16	2		2	>16	>2
VIII	2012-C01-027	Urine	40	<=4	>16	8		2	>16	>2
VIII	2012-C01-028	Urine	32	8	>16	8		<=1	>16	>2
VIII	2012-C01-029	Urine	0	<=4	>16	>16		16	>16	>2
VIII	2012-C01-042	Urine	61	32	>16	16		4	>16	>2
VIII	2012-C01-043	Urine	61	<=4	>16	16		2	>16	>2
VIII	2012-C01-044	Urine	85	16	>16	>16		4	>16	>2
VIII	2012-C01-046	Urine	52	<=4	>16	>16		8	>16	<=0.06
VIII	2012-C01-047	Other	82	<=4	>16	8		4	>16	>2
VIII	2012-C02-004	Other	35	<=4	>16	<=1		<=1	8	<=0.06
VIII	2012-C02-009	Urine	84	<=4	>16	8		8	>16	<=0.06
VIII	2012-C02-010	Urine	61	<=4	>16	<=1		<=1	4	<=0.06
VIII	2012-C02-011	Urine	24	<=4	>16	<=1		<=1	4	0.25
VIII	2012-C02-020	Urine	69	<=4	>16	>16		>16	>16	>2
VIII	2012-C02-021	Urine	42	8	>16	<=1		<=1	4	<=0.06
VIII	2012-C02-023	Urine	54	<=4	>16	<=1		<=1	4	>2
VIII	2012-C02-024	Urine	57	<=4	>16	<=1		<=1	4	<=0.06
VIII	2012-C02-025	Urine	59	<=4	>16	<=1		<=1	4	0.5
VIII	2012-C02-034	Urine	60	8	>16	<=1		<=1	>16	0.25
VIII	2012-C02-035	Urine	54	<=4	>16	<=1		<=1	8	<=0.06
VIII	2012-C02-036	Urine	22	8	>16	<=1		<=1	16	<=0.06
VIII	2012-C02-038	Urine	38	<=4	4	<=1		<=1	<=2	<=0.06
VIII	2012-C02-040	Urine	56	<=4	<=2	<=1		<=1	<=2	0.12
VIII	2012-C02-041	Blood	70	<=4	4	<=1		<=1	<=2	<=0.06
VIII	2012-C02-043	Urine	87	<=4	>16	>16		16	>16	>2
VIII	2012-C02-044	Urine	59	<=4	>16	>16		16	>16	<=0.06
VIII	2012-C02-047	Urine	29	<=4	4	<=1		<=1	<=2	<=0.06
VIII	2012-C02-183	Urine	13	<=4	>16	<=1		<=1	>16	0.25
VIII	2012-C02-213	Blood	43	<=4	<=2	<=1		<=1	<=2	0.12
VIII	2012-C02-214	Blood	86	<=4	4	<=1		<=1	<=2	<=0.06
VIII	2012-C02-238	Blood	69	<=4	<=2	<=1		<=1	<=2	<=0.06
VIII	2012-C11-001	Urine	83	<=4	>16	<=1		<=1	8	0.25
VIII	2012-C11-004	Urine	37	<=4	>16	<=1		<=1	8	0.25
VIII	2012-C11-005	Other	41	<=4	>16	<=1		<=1	4	0.25
VIII	2012-C11-007	Urine	17	<=4	>16	<=1		<=1	<=2	0.12
VIII	2012-C11-013	Urine	83	<=4	>16	>16		>16	>16	2
VIII	2012-C11-014	Urine	36	<=4	>16	<=1		<=1	4	<=0.06
VIII	2012-C11-015	Blood	80	<=4	>16	<=1		<=1	8	<=0.06
VIII	2012-C11-016	Urine	49	<=4	>16	<=1		<=1	16	<=0.06
VIII	2012-C11-017	Urine	64	<=4	>16	<=1		<=1	4	<=0.06
VIII	2012-C11-023	Urine	25	<=4	>16	<=1		<=1	8	<=0.06
VIII	2012-C11-027	Urine	37	<=4	>16	<=1		<=1	4	<=0.06
VIII	2012-C11-028	Urine	74	<=4	>16	<=1		<=1	<=2	<=0.06
VIII	2012-C11-029	Blood	29	<=4	>16	<=1		<=1	4	0.25
VIII	2012-C11-031	Blood	30	<=4	>16	<=1		<=1	<=2	<=0.06
VIII	2012-C11-033	Blood	77	<=4	>16	>16		>16	>16	>2
VIII	2012-C11-036	Urine	39	<=4	>16	<=1		<=1	4	<=0.06
VIII	2012-C11-042	Urine	40	<=4	>16	<=1		<=1	4	<=0.06
VIII	2012-C11-046	Urine	38	<=4	>16	<=1		<=1	<=2	<=0.06

VIII	2012-C11-047	Urine	0	<=4	>16	<=1		<=1	4	0.25
VIII	2012-C11-201	Blood	61	<=4	>16	<=1		<=1	4	<=0.06
VIII	2012-C11-203	Blood	52	8	<=2	<=1		<=1	<=2	<=0.06
VIII	2012-C11-206	Blood	90	<=4	<=2	<=1		<=1	<=2	0.12
VIII	2012-C11-212	Blood	54	<=4	4	<=1		<=1	<=2	0.25
VIII	2012-C11-215	Blood	82	<=4	>16	>16		>16	>16	>2
VIII	2012-C11-231	Blood	54	<=4	4	<=1		<=1	<=2	<=0.06
VIII	2012-C11-238	Other	55	<=4	>16	<=1		<=1	4	<=0.06
VIII	2012-C11-245	Blood	44	<=4	>16	<=1		<=1	<=2	<=0.06
VIII	2012-C11-247	Blood	84	<=4	>16	<=1		<=1	<=2	<=0.06
VIII	2012-C04-001	Blood	81	<=4	>16	<=1		<=1	8	<=0.06
VIII	2012-C04-008	Urine	80	<=4	>16	<=1		<=1	>16	0.5
VIII	2012-C04-020	Urine	55	<=4	4	<=1		<=1	<=2	<=0.06
VIII	2012-C04-036	Urine	5	<=4	>16	8		16	>16	<=0.06
VIII	2012-C04-037	Urine	78	<=4	>16	4		8	>16	>2
VIII	2012-C04-042	Urine	82	<=4	>16	<=1		<=1	4	<=0.06
VIII	2012-C04-204	Blood	37	<=4	4	<=1		<=1	<=2	<=0.06
VIII	2012-C04-214	Blood	53	<=4	>16	<=1		<=1	<=2	<=0.06
VIII	2012-C04-215	Blood	69	8	>16	<=1		<=1	<=2	1
VIII	2012-C04-216	Blood	54	8	>16	>16		>16	>16	>2
VIII	2012-C04-233	Blood	81	8	>16	<=1		<=1	8	>2
VIII	2012-C04-241	Blood	101	<=4	>16	<=1		<=1	4	>2
VIII	2012-C04-249	Blood	59	<=4	>16	8		16	>16	>2
VIII	2012-S05-003	Urine	86	8	4	<=1		<=1	<=2	<=0.06
VIII	2012-S05-006	Urine	68	<=4	>16	<=1		<=1	8	<=0.06
VIII	2012-S05-008	Urine	68	8	>16	8		16	>16	>2
VIII	2012-S05-009	Urine	82	<=4	>16	<=1		<=1	4	>2
VIII	2012-S05-010	Urine	36	<=4	4	<=1		<=1	<=2	<=0.06
VIII	2012-S05-012	Urine	78	<=4	>16	8		>16	>16	>2
VIII	2012-S05-015	Urine	28	<=4	>16	<=1		<=1	4	<=0.06
VIII	2012-S05-025	Urine	74	<=4	>16	<=1		<=1	8	0.25
VIII	2012-S05-028	Blood	75	<=4	>16	<=1		<=1	4	<=0.06
VIII	2012-S05-036	Urine	52	<=4	>16	8		<=1	>16	>2
VIII	2012-S05-043	Urine	70	<=4	>16	4		4	>16	>2
VIII	2012-S05-211	Blood	82	<=4	4	<=1		<=1	<=2	>2
VIII	2012-S05-220	Blood	77	<=4	>16	4		<=1	>16	>2
VIII	2012-S05-222	Blood	59	<=4	>16	<=1		<=1	4	<=0.06
VIII	2012-S05-225	Blood	74	<=4	4	<=1		<=1	4	0.5
VIII	2012-S05-229	Blood	80	<=4	4	<=1		<=1	4	<=0.06
VIII	2012-S05-231	Blood	93	<=4	>16	>16		16	>16	>2
VIII	2012-S05-233	Blood	79	<=4	>16	<=1		<=1	8	<=0.06
VIII	2012-S05-234	Blood	61	<=4	4	<=1		<=1	<=2	<=0.06
VIII	2012-S05-239	Blood	72	<=4	>16	<=1		<=1	16	<=0.06
VIII	2012-S05-240	Blood	76	<=4	>16	<=1		4	>16	<=0.06
VIII	2012-S05-246	Blood	86	<=4	>16	<=1		<=1	8	>2
VIII	2012-S05-250	Blood	78	<=4	>16	<=1		<=1	4	<=0.06
VIII	2012-S06-002	Urine	60	<=4	>16	<=1		<=1	4	>2
VIII	2012-S06-004	Urine	55	<=4	>16	<=1		<=1	4	<=0.06
VIII	2012-S06-005	Urine	51	<=4	>16	>16		4	>16	>2
VIII	2012-S06-006	Urine	75	<=4	>16	>16		>16	>16	>2
VIII	2012-S06-008	Urine	64	<=4	>16	<=1		<=1	<=2	<=0.06
VIII	2012-S06-010	Urine	68	<=4	8	<=1		<=1	4	<=0.06
VIII	2012-S06-015	Urine	73	<=4	>16	<=1		<=1	4	0.5
VIII	2012-S06-017	Urine	77	8	>16	16		>16	>16	>2
VIII	2012-S06-018	Urine	62	8	4	<=1		<=1	4	<=0.06
VIII	2012-S06-019	Urine	31	<=4	<=2	<=1		<=1	<=2	<=0.06
VIII	2012-S06-022	Urine	57	<=4	>16	<=1		<=1	8	<=0.06
VIII	2012-S06-024	Urine	23	<=4	4	<=1		<=1	<=2	<=0.06
VIII	2012-S06-025	Urine	84	8	>16	<=1		<=1	>16	2
VIII	2012-S06-028	Urine	46	<=4	>16	<=1		<=1	8	<=0.06
VIII	2012-S06-030	Urine	65	<=4	4	<=1		<=1	<=2	<=0.06

VIII	2012-S06-032	Urine	63	<=4	>16	<=1		<=1	16	<=0.06
VIII	2012-S06-033	Urine	5	<=4	>16	<=1		<=1	4	<=0.06
VIII	2012-S06-035	Urine	77	<=4	>16	<=1		<=1	8	0.5
VIII	2012-S06-036	Urine	81	<=4	>16	>16		>16	>16	>2
VIII	2012-S06-037	Urine	62	8	>16	<=1		<=1	4	<=0.06
VIII	2012-S06-043	Blood	73	<=4	>16	<=1		<=1	>16	>2
VIII	2012-S06-046	Urine	70	<=4	>16	<=1		<=1	8	<=0.06
VIII	2012-S06-205	Blood	65	<=4	4	<=1		<=1	<=2	<=0.06
VIII	2012-S06-206	Blood	63	<=4	>16	2		<=1	>16	0.25
VIII	2012-S06-207	Blood	83	<=4	<=2	<=1		<=1	<=2	<=0.06
VIII	2012-S06-211	Blood	88	<=4	>16	<=1		<=1	4	2
VIII	2012-S06-220	Blood	61	<=4	>16	<=1		<=1	16	0.25
VIII	2012-S06-222	Blood	38	<=4	>16	<=1		<=1	16	<=0.06
VIII	2012-S06-223	Blood	74	<=4	>16	<=1		<=1	<=2	0.5
VIII	2012-S06-246	Blood	84	<=4	>16	<=1		<=1	8	>2
VIII	2012-S07-002	Blood	69	8	>16	<=1		<=1	4	<=0.06
VIII	2012-S07-007	Blood	80	<=4	>16	<=1		<=1	4	0.25
VIII	2012-S07-008	Blood	50	<=4	<=2	<=1		<=1	<=2	<=0.06
VIII	2012-S07-011	Urine	78	<=4	4	<=1		<=1	4	0.25
VIII	2012-S07-012	Urine	24	<=4	>16	8		16	>16	>2
VIII	2012-S07-013	Urine	94	<=4	>16	16		>16	>16	>2
VIII	2012-S07-014	Other	46	<=4	<=2	<=1		<=1	4	<=0.06
VIII	2012-S07-018	Urine	3	<=4	>16	<=1		<=1	4	>2
VIII	2012-S07-023	Urine	54	<=4	<=2	<=1		<=1	<=2	<=0.06
VIII	2012-S07-027	Other	81	<=4	4	<=1		<=1	<=2	<=0.06
VIII	2012-S07-034	Urine	98	<=4	>16	<=1		<=1	>16	>2
VIII	2012-S07-040	Urine	82	8	>16	<=1		<=1	16	>2
VIII	2012-S07-049	Other	72	<=4	>16	<=1		<=1	<=2	<=0.06
VIII	2012-S07-212	Blood	57	<=4	<=2	<=1		<=1	<=2	>2
VIII	2012-S07-215	Blood	60	<=4	>16	<=1		<=1	<=2	<=0.06
VIII	2012-S07-216	Blood	73	<=4	>16	<=1		<=1	4	<=0.06
VIII	2012-S07-223	Blood	73	<=4	>16	<=1		<=1	4	0.25
VIII	2012-S07-228	Blood	58	<=4	>16	16		8	>16	>2
VIII	2012-S07-229	Blood	36	<=4	>16	<=1		<=1	4	0.5
VIII	2012-S07-230	Blood	53	<=4	4	<=1		<=1	<=2	<=0.06
VIII	2012-S07-231	Blood	83	<=4	4	<=1		<=1	4	<=0.06
VIII	2012-S07-241	Blood	81	<=4	>16	<=1		<=1	<=2	<=0.06
VIII	2012-S07-243	Blood	60	<=4	>16	4		16	>16	<=0.06
VIII	2012-N05-001	Urine	22	<=4	>16	<=1		<=1	16	0.12
VIII	2012-N05-002	Urine	51	<=4	>16	<=1		<=1	>16	<=0.06
VIII	2012-N05-003	Urine	78	<=4	>16	<=1		<=1	4	0.25
VIII	2012-N05-007	Urine	29	<=4	16	<=1		<=1	<=2	0.5
VIII	2012-N05-009	Urine	45	<=4	>16	<=1		<=1	4	<=0.06
VIII	2012-N05-017	Urine	39	<=4	>16	<=1		<=1	4	<=0.06
VIII	2012-N05-019	Urine	23	<=4	4	<=1		<=1	<=2	<=0.06
VIII	2012-N05-022	Urine	55	<=4	<=2	<=1		<=1	<=2	<=0.06
VIII	2012-N05-023	Urine	81	<=4	>16	<=1		<=1	<=2	>2
VIII	2012-N05-025	Urine	51	<=4	>16	<=1		<=1	16	<=0.06
VIII	2012-N05-028	Urine	61	<=4	>16	2		<=1	4	<=0.06
VIII	2012-N05-032	Urine	14	<=4	<=2	<=1		<=1	<=2	<=0.06
VIII	2012-N05-033	Urine	83	<=4	4	<=1		<=1	<=2	<=0.06
VIII	2012-N05-034	Urine	53	<=4	<=2	<=1		<=1	<=2	>2
VIII	2012-N05-035	Urine	57	<=4	>16	<=1		<=1	4	1
VIII	2012-N05-036	Urine	76	<=4	>16	<=1		<=1	<=2	<=0.06
VIII	2012-N05-037	Urine	40	<=4	>16	<=1		<=1	4	<=0.06
VIII	2012-N05-038	Urine	54	<=4	>16	<=1		<=1	4	<=0.06
VIII	2012-N05-040	Urine	32	<=4	4	<=1		<=1	<=2	<=0.06
VIII	2012-N05-041	Urine	24	<=4	>16	<=1		<=1	>16	1
VIII	2012-N05-042	Urine	36	<=4	>16	<=1		<=1	4	<=0.06
VIII	2012-N05-043	Urine	64	<=4	<=2	<=1		<=1	<=2	<=0.06
VIII	2012-N05-044	Other	68	<=4	>16	<=1		<=1	>16	>2

VIII	2012-N05-047	Urine	27	<=4	>16	<=1		<=1	8	<=0.06
VIII	2012-N05-048	Urine	24	<=4	>16	<=1		<=1	4	0.25
VIII	2012-N05-195	Urine	12	<=4	>16	<=1		<=1	<=2	0.12
VIII	2012-N05-207	Blood	93	<=4	<=2	<=1		<=1	<=2	<=0.06
VIII	2012-N05-223	Blood	93	<=4	>16	<=1		<=1	4	<=0.06
VIII	2012-N05-224	Blood	49	<=4	4	<=1		<=1	<=2	<=0.06
VIII	2012-N05-238	Blood	62	<=4	<=2	<=1		<=1	<=2	<=0.06
VIII	2012-N05-244	Blood	3	<=4	>16	<=1		<=1	4	>2
VIII	2012-N05-251	Urine	28	<=4	>16	<=1		<=1	<=2	<=0.06
VIII	2012-N05-280	Urine	49	<=4	<=2	<=1		<=1	<=2	0.25
VIII	2012-N06-002	Other	59	<=4	>16	8		4	>16	>2
VIII	2012-N06-003	Urine	38	<=4	>16	<=1		<=1	<=2	<=0.06
VIII	2012-N06-004	Urine	28	<=4	>16	<=1		<=1	16	<=0.06
VIII	2012-N06-005	Urine	79	<=4	>16	<=1		<=1	8	0.25
VIII	2012-N06-007	Urine	78	<=4	>16	<=1		<=1	4	0.25
VIII	2012-N06-011	Urine	72	<=4	>16	<=1		<=1	8	>2
VIII	2012-N06-013	Urine	81	<=4	>16	<=1		<=1	>16	0.25
VIII	2012-N06-017	Urine	82	<=4	>16	<=1		<=1	4	0.25
VIII	2012-N06-020	Urine	42	<=4	>16	<=1		<=1	<=2	<=0.06
VIII	2012-N06-021	Urine	75	<=4	>16	>16		4	>16	>2
VIII	2012-N06-024	Urine	82	<=4	4	<=1		<=1	<=2	0.25
VIII	2012-N06-025	Urine	84	<=4	>16	8		16	>16	>2
VIII	2012-N06-027	Urine	28	<=4	4	<=1		<=1	<=2	<=0.06
VIII	2012-N06-028	Urine	45	<=4	>16	<=1		<=1	4	<=0.06
VIII	2012-N06-030	Urine	1	<=4	>16	<=1		<=1	8	<=0.06
VIII	2012-N06-031	Urine	81	<=4	4	<=1		<=1	<=2	<=0.06
VIII	2012-N06-035	Urine	61	<=4	>16	2		8	>16	>2
VIII	2012-N06-036	Urine	86	<=4	4	<=1		<=1	<=2	<=0.06
VIII	2012-N06-039	Urine	52	<=4	4	<=1		<=1	<=2	<=0.06
VIII	2012-N06-040	Urine	38	<=4	4	<=1		<=1	<=2	<=0.06
VIII	2012-N06-041	Urine	89	<=4	>16	<=1		<=1	<=2	<=0.06
VIII	2012-N06-044	Urine	65	<=4	>16	<=1		<=1	4	<=0.06
VIII	2012-N06-045	Urine	78	<=4	>16	16		4	>16	1
VIII	2012-N06-047	Urine	27	<=4	4	<=1		<=1	<=2	<=0.06
VIII	2012-N06-050	Urine	36	<=4	<=2	<=1		<=1	<=2	<=0.06
VIII	2012-N06-205	Blood	93	<=4	>16	>16		>16	>16	>2
VIII	2012-N06-206	Blood	75	<=4	<=2	<=1		<=1	<=2	<=0.06
VIII	2012-N06-212	Blood	54	<=4	>16	<=1		<=1	4	0.25
VIII	2012-N06-216	Blood	82	8	<=2	<=1		<=1	<=2	0.25
VIII	2012-N06-218	Blood	68	<=4	>16	<=1		<=1	<=2	0.12
VIII	2012-N06-223	Blood	77	<=4	>16	2		<=1	>16	0.12
VIII	2012-N06-229	Blood	79	<=4	>16	<=1		<=1	4	0.25
VIII	2012-N06-230	Blood	63	<=4	>16	>16		>16	>16	>2
VIII	2012-N06-250	Blood	69	<=4	4	<=1		<=1	4	0.25
VIII	2012-N07-002	Blood	80	<=4	>16	<=1		<=1	8	<=0.06
VIII	2012-N07-003	Urine	36	<=4	>16	>16		>16	>16	<=0.06
VIII	2012-N07-004	Urine	88	<=4	<=2	<=1		<=1	<=2	<=0.06
VIII	2012-N07-006	Urine	46	<=4	>16	<=1		<=1	4	2
VIII	2012-N07-007	Urine	89	<=4	>16	<=1		<=1	4	0.12
VIII	2012-N07-010	Urine	29	<=4	>16	<=1		<=1	4	<=0.06
VIII	2012-N07-016	Blood	45	<=4	>16	<=1		<=1	<=2	<=0.06
VIII	2012-N07-017	Blood	69	<=4	>16	<=1		<=1	4	<=0.06
VIII	2012-N07-021	Urine	45	<=4	<=2	<=1		<=1	<=2	<=0.06
VIII	2012-N07-024	Urine	57	<=4	>16	<=1		<=1	8	<=0.06
VIII	2012-N07-030	Urine	45	<=4	<=2	<=1		<=1	<=2	<=0.06
VIII	2012-N07-031	Urine	72	<=4	>16	8		16	>16	<=0.06
VIII	2012-N07-035	Blood	84	<=4	>16	<=1		<=1	4	0.12
VIII	2012-N07-037	Urine	56	8	<=2	<=1		<=1	<=2	<=0.06
VIII	2012-N07-039	Blood	86	<=4	4	<=1		<=1	<=2	<=0.06
VIII	2012-N07-040	Other	44	<=4	>16	8		16	>16	>2
VIII	2012-N07-043	Urine	81	8	<=2	<=1		<=1	<=2	<=0.06

VIII	2012-N07-044	Urine	65	<=4	>16	>16		>16	>16	<=0.06
VIII	2012-N07-045	Urine	78	<=4	>16	>16		4	>16	>2
VIII	2012-N07-046	Blood	19	<=4	<=2	<=1		<=1	<=2	<=0.06
VIII	2012-N07-047	Urine	29	<=4	>16	8		8	>16	<=0.06
VIII	2012-N07-048	Urine	19	<=4	<=2	<=1		<=1	<=2	<=0.06
VIII	2012-N07-049	Urine	33	<=4	>16	<=1		<=1	4	<=0.06
VIII	2012-N07-050	Urine	68	<=4	<=2	<=1		<=1	4	<=0.06
VIII	2012-N07-191	Urine	0	<=4	>16	<=1		<=1	<=2	<=0.06
VIII	2012-N07-195	Urine	4	<=4	>16	<=1		<=1	4	<=0.06
VIII	2012-N07-196	Urine	7	<=4	4	<=1		<=1	<=2	<=0.06
VIII	2012-N07-198	Urine	16	<=4	<=2	<=1		<=1	<=2	<=0.06
VIII	2012-N07-218	Blood	67	<=4	4	<=1		<=1	<=2	<=0.06
VIII	2012-N07-222	Blood	79	8	>16	<=1		<=1	<=2	<=0.06
VIII	2012-N07-226	Blood	31	<=4	>16	<=1		<=1	16	<=0.06
VIII	2012-N07-228	Blood	76	<=4	>16	<=1		<=1	4	0.12
VIII	2012-N07-230	Blood	48	8	<=2	<=1		<=1	<=2	<=0.06
VIII	2012-N07-233	Blood	58	<=4	4	<=1		<=1	4	0.25
VIII	2012-N07-234	Blood	72	8	>16	<=1		<=1	<=2	0.25
VIII	2012-N07-235	Blood	36	8	<=2	<=1		<=1	<=2	0.25
VIII	2012-N07-241	Blood	70	<=4	>16	>16		>16	>16	>2
VIII	2012-N07-242	Blood	88	<=4	>16	>16		>16	>16	>2
VIII	2012-N07-247	Blood	51	<=4	>16	<=1		<=1	<=2	<=0.06
VIII	2012-N07-249	Blood	82	<=4	>16	<=1		<=1	<=2	<=0.06
VIII	2012-S08-001	Urine	27	<=4	>16	<=1		<=1	8	<=0.06
VIII	2012-S08-002	Urine	78	16	>16	>16		>16	>16	>2
VIII	2012-S08-007	Urine	66	8	>16	<=1		<=1	8	0.25
VIII	2012-S08-008	Urine	60	<=4	>16	<=1		<=1	4	>2
VIII	2012-S08-009	Urine	70	<=4	>16	<=1		<=1	8	<=0.06
VIII	2012-S08-010	Blood	78	<=4	>16	<=1		<=1	4	<=0.06
VIII	2012-S08-013	Urine	84	<=4	>16	<=1		<=1	4	<=0.06
VIII	2012-S08-014	Urine	85	<=4	>16	<=1		<=1	8	>2
VIII	2012-S08-016	Urine	87	<=4	>16	8		4	>16	>2
VIII	2012-S08-020	Urine	57	<=4	>16	<=1		<=1	4	1
VIII	2012-S08-021	Urine	51	<=4	<=2	<=1		<=1	<=2	<=0.06
VIII	2012-S08-023	Urine	30	<=4	<=2	<=1		<=1	<=2	<=0.06
VIII	2012-S08-025	Urine	54	8	>16	>16		>16	>16	>2
VIII	2012-S08-026	Urine	66	<=4	4	<=1		<=1	<=2	<=0.06
VIII	2012-S08-028	Urine	87	<=4	>16	<=1		<=1	<=2	<=0.06
VIII	2012-S08-029	Urine	69	<=4	>16	<=1		<=1	4	<=0.06
VIII	2012-S08-031	Urine	47	<=4	>16	<=1		<=1	<=2	<=0.06
VIII	2012-S08-032	Urine	87	<=4	>16	<=1		<=1	8	<=0.06
VIII	2012-S08-033	Urine	84	<=4	>16	<=1		<=1	16	>2
VIII	2012-S08-034	Urine	91	<=4	>16	<=1		<=1	>16	<=0.06
VIII	2012-S08-035	Urine	21	<=4	>16	<=1		<=1	4	<=0.06
VIII	2012-S08-036	Urine	51	8	>16	16		>16	>16	>2
VIII	2012-S08-037	Urine	28	<=4	>16	<=1		<=1	4	0.12
VIII	2012-S08-042	Urine	58	16	>16	8		16	>16	>2
VIII	2012-S08-043	Urine	23	<=4	>16	<=1		<=1	4	0.12
VIII	2012-S08-049	Urine	27	<=4	>16	<=1		<=1	<=2	0.12
VIII	2012-E02-004	Blood	61	<=4	<=2	<=1		<=1	<=2	<=0.06
VIII	2012-E02-005	Urine	82	<=4	>16	<=1		<=1	8	<=0.06
VIII	2012-E02-007	Urine	46	<=4	4	<=1		<=1	<=2	<=0.06
VIII	2012-E02-008	Urine	86	<=4	>16	>16		8	>16	>2
VIII	2012-E02-009	Urine	82	<=4	<=2	<=1		<=1	<=2	>2
VIII	2012-E02-010	Urine	17	<=4	>16	8		2	>16	<=0.06
VIII	2012-E02-012	Urine	51	<=4	>16	>16		4	>16	>2
VIII	2012-E02-014	Urine	57	<=4	<=2	<=1		<=1	<=2	<=0.06
VIII	2012-E02-015	Blood	70	<=4	>16	<=1		<=1	8	>2
VIII	2012-E02-017	Urine	73	<=4	4	<=1		<=1	<=2	<=0.06
VIII	2012-E02-019	Urine	58	<=4	>16	<=1		<=1	4	0.25
VIII	2012-E02-020	Urine	75	<=4	>16	<=1		<=1	>16	>2

VIII	2012-E02-024	Urine	0	<=4	>16	<=1		<=1	<=2	<=0.06
VIII	2012-E02-029	Urine	79	<=4	>16	<=1		<=1	4	<=0.06
VIII	2012-E02-030	Urine	81	<=4	4	<=1		<=1	<=2	0.25
VIII	2012-E02-031	Urine	72	<=4	>16	<=1		<=1	<=2	<=0.06
VIII	2012-E02-033	Urine	91	<=4	>16	>16		16	>16	0.12
VIII	2012-E02-035	Urine	23	<=4	>16	<=1		<=1	4	0.25
VIII	2012-E02-039	Urine	33	<=4	>16	<=1		<=1	<=2	>2
VIII	2012-E02-040	Urine	74	<=4	8	<=1		<=1	4	0.25
VIII	2012-E02-041	Urine	65	<=4	>16	>16		>16	>16	>2
VIII	2012-E02-043	Blood	69	<=4	>16	<=1		<=1	4	0.25
VIII	2012-E02-045	Urine	20	<=4	4	<=1		<=1	<=2	<=0.06
VIII	2012-E02-049	Blood	76	<=4	<=2	<=1		<=1	4	>2
VIII	2012-E02-184	Urine	7	<=4	>16	<=1		<=1	8	0.5
VIII	2012-E02-185	Urine	10	<=4	>16	<=1		<=1	8	1
VIII	2012-E02-191	Urine	1	8	>16	<=1		<=1	>16	<=0.06
VIII	2012-E02-198	Urine	8	<=4	>16	<=1		<=1	4	<=0.06
VIII	2012-E02-201	Blood	75	<=4	>16	<=1		<=1	4	0.5
VIII	2012-E02-202	Blood	83	<=4	4	<=1		<=1	<=2	<=0.06
VIII	2012-E02-203	Blood	64	<=4	>16	<=1		<=1	4	<=0.06
VIII	2012-E02-204	Blood	75	<=4	4	<=1		<=1	<=2	<=0.06
VIII	2012-E02-205	Blood	49	<=4	4	<=1		<=1	<=2	<=0.06
VIII	2012-E02-209	Blood	67	<=4	<=2	<=1		<=1	<=2	<=0.06
VIII	2012-E02-227	Blood	70	<=4	>16	<=1		<=1	4	<=0.06
VIII	2012-E02-228	Blood	56	<=4	<=2	<=1		<=1	<=2	<=0.06
VIII	2012-E02-229	Blood	74	<=4	>16	<=1		<=1	<=2	<=0.06
VIII	2012-E02-240	Blood	58	<=4	>16	<=1		<=1	4	<=0.06
VIII	2012-E02-242	Blood	87	<=4	<=2	<=1		<=1	<=2	0.12
VIII	2012-E02-243	Blood	68	<=4	<=2	<=1		<=1	<=2	<=0.06
VIII	2012-E02-246	Blood	57	<=4	>16	<=1		<=1	>16	<=0.06
VIII	2012-N08-002	Blood	65	<=4	>16	2		8	>16	<=0.06
VIII	2012-N08-004	Urine	44	<=4	>16	<=1		<=1	<=2	<=0.06
VIII	2012-N08-008	Blood	68	<=4	>16	<=1		<=1	16	0.25
VIII	2012-N08-009	Urine	75	8	>16	<=1		<=1	4	>2
VIII	2012-N08-014	Urine	73	<=4	>16	4		16	>16	0.25
VIII	2012-N08-015	Urine	87	<=4	>16	8		>16	>16	<=0.06
VIII	2012-N08-016	Urine	37	<=4	<=2	<=1		<=1	<=2	<=0.06
VIII	2012-N08-023	Blood	59	<=4	>16	8		>16	>16	0.5
VIII	2012-N08-027	Urine	26	<=4	<=2	<=1		2	<=2	<=0.06
VIII	2012-N08-028	Other	0	<=4	>16	<=1		<=1	4	1
VIII	2012-N08-031	Urine	58	<=4	>16	<=1		<=1	>16	<=0.06
VIII	2012-N08-036	Urine	26	<=4	>16	<=1		<=1	4	<=0.06
VIII	2012-N08-038	Urine	23	<=4	>16	<=1		<=1	4	>2
VIII	2012-N08-044	Urine	83	<=4	>16	<=1		<=1	8	<=0.06
VIII	2012-N08-048	Urine	46	<=4	<=2	<=1		<=1	<=2	<=0.06
VIII	2012-N08-049	Urine	45	<=4	>16	<=1		<=1	<=2	<=0.06
VIII	2012-N08-201	Blood	64	<=4	4	<=1		<=1	<=2	>2
VIII	2012-N08-203	Blood	62	<=4	<=2	<=1		<=1	<=2	<=0.06
VIII	2012-N08-223	Blood	80	<=4	>16	<=1		<=1	4	0.12
VIII	2012-N08-226	Blood	72	<=4	>16	>16		16	>16	0.5
VIII	2012-N08-231	Blood	90	<=4	>16	<=1		<=1	8	0.5
VIII	2012-N08-232	Blood	90	<=4	>16	8		>16	>16	>2
VIII	2012-N08-239	Blood	59	<=4	>16	<=1		<=1	<=2	<=0.06
VIII	2012-N08-240	Blood	77	<=4	>16	<=1		<=1	8	0.12
VIII	2012-N08-241	Blood	70	<=4	<=2	<=1		<=1	<=2	<=0.06
VIII	2012-N08-249	Blood	54	<=4	<=2	<=1		<=1	<=2	0.25
VIII	2012-S09-002	Blood	69	<=4	4	<=1		<=1	<=2	<=0.06
VIII	2012-S09-003	Blood	61	<=4	>16	<=1		<=1	4	>2
VIII	2012-S09-004	Urine	78	16	>16	16		<=1	8	>2
VIII	2012-S09-005	Urine	78	<=4	>16	<=1		<=1	4	<=0.06
VIII	2012-S09-006	Other	29	<=4	<=2	<=1		<=1	<=2	<=0.06
VIII	2012-S09-007	Urine	19	<=4	>16	<=1		<=1	4	<=0.06

VIII	2012-S09-009	Urine	15	<=4	>16	<=1		<=1	4	<=0.06
VIII	2012-S09-010	Urine	57	<=4	>16	<=1		<=1	<=2	>2
VIII	2012-S09-011	Urine	66	<=4	>16	<=1		<=1	<=2	<=0.06
VIII	2012-S09-012	Blood	42	<=4	>16	<=1		<=1	>16	<=0.06
VIII	2012-S09-017	Urine	53	<=4	>16	<=1		<=1	<=2	<=0.06
VIII	2012-S09-018	Urine	68	<=4	>16	<=1		<=1	<=2	0.12
VIII	2012-S09-021	Blood	68	<=4	<=2	<=1		<=1	<=2	<=0.06
VIII	2012-S09-024	Blood	68	<=4	>16	<=1		<=1	<=2	0.12
VIII	2012-S09-025	Blood	78	<=4	<=2	<=1		<=1	<=2	<=0.06
VIII	2012-S09-026	Urine	64	<=4	>16	>16		>16	>16	>2
VIII	2012-S09-027	Urine	63	<=4	>16	>16		16	>16	>2
VIII	2012-S09-033	Urine	81	<=4	>16	<=1		<=1	4	<=0.06
VIII	2012-S09-039	Urine	69	<=4	8	<=1		<=1	8	>2
VIII	2012-S09-044	Blood	78	8	>16	<=1		<=1	8	<=0.06
VIII	2012-S09-045	Blood	74	<=4	>16	<=1		<=1	4	0.12
VIII	2012-S09-047	Urine	83	8	4	<=1		<=1	4	>2
VIII	2012-S09-203	Blood	61	<=4	>16	<=1		<=1	8	<=0.06
VIII	2012-S09-209	Blood	88	<=4	<=2	<=1		<=1	<=2	<=0.06
VIII	2012-S09-210	Blood	69	<=4	>16	>16		16	>16	0.5
VIII	2012-S09-215	Other	74	<=4	>16	<=1		<=1	4	>2
VIII	2012-S09-219	Blood	41	<=4	>16	<=1		<=1	4	<=0.06
VIII	2012-S09-226	Blood	46	<=4	>16	>16		>16	>16	>2
VIII	2012-S09-232	Blood	78	<=4	>16	<=1		<=1	4	<=0.06
VIII	2012-S09-234	Blood	85	<=4	>16	<=1		<=1	<=2	<=0.06
VIII	2012-S09-235	Blood	83	<=4	>16	<=1		<=1	4	<=0.06
VIII	2012-S09-236	Blood	86	8	>16	>16		>16	>16	>2
VIII	2012-S09-244	Blood	65	<=4	4	<=1		<=1	<=2	<=0.06
VIII	2012-E03-001	Urine	19	<=4	<=2	<=1		<=1	<=2	<=0.06
VIII	2012-E03-003	Urine	26	<=4	>16	<=1		<=1	<=2	0.25
VIII	2012-E03-004	Other	60	<=4	>16	<=1		<=1	<=2	<=0.06
VIII	2012-E03-005	Urine	31	<=4	<=2	<=1		<=1	<=2	<=0.06
VIII	2012-E03-006	Urine	38	<=4	>16	<=1		<=1	8	<=0.06
VIII	2012-E03-008	Urine	68	<=4	>16	<=1		<=1	8	>2
VIII	2012-E03-009	Urine	73	<=4	>16	<=1		<=1	8	0.25
VIII	2012-E03-014	Urine	54	<=4	<=2	<=1		<=1	<=2	<=0.06
VIII	2012-E03-016	Urine	77	<=4	>16	8		>16	>16	<=0.06
VIII	2012-E03-017	Urine	65	<=4	>16	<=1		<=1	4	>2
VIII	2012-E03-020	Urine	64	<=4	>16	<=1		<=1	<=2	<=0.06
VIII	2012-E03-021	Urine	56	<=4	>16	<=1		<=1	>16	<=0.06
VIII	2012-E03-022	Urine	76	<=4	>16	<=1		<=1	4	<=0.06
VIII	2012-E03-024	Urine	81	<=4	4	<=1		<=1	4	<=0.06
VIII	2012-E03-025	Urine	69	32	>16	>16		>16	>16	>2
VIII	2012-E03-026	Urine	23	<=4	>16	<=1		<=1	4	<=0.06
VIII	2012-E03-027	Urine	67	<=4	>16	<=1		<=1	4	<=0.06
VIII	2012-E03-030	Urine	54	<=4	>16	<=1		<=1	4	>2
VIII	2012-E03-035	Urine	38	<=4	>16	8		4	>16	>2
VIII	2012-E03-041	Urine	94	<=4	>16	8		>16	>16	<=0.06
VIII	2012-E03-045	Urine	55	<=4	>16	<=1		<=1	8	>2
VIII	2012-E03-046	Urine	67	<=4	<=2	<=1		<=1	<=2	<=0.06
VIII	2012-E03-234	Blood	58	<=4	<=2	<=1		<=1	<=2	<=0.06
VIII	2012-E03-239	Blood	38	<=4	<=2	<=1		<=1	<=2	<=0.06
VIII	2012-E03-249	Blood	78	<=4	>16	16		>16	>16	>2
VIII	2012-C06-003	Urine	53	<=4	>16	<=1		<=1	<=2	0.12
VIII	2012-C06-008	Blood	83	>32	>16	<=1		2	>16	>2
VIII	2012-C06-013	Urine	81	<=4	>16	<=1		<=1	>16	>2
VIII	2012-C06-014	Urine	71	<=4	>16	<=1		<=1	8	>2
VIII	2012-C06-017	Urine	72	<=4	8	<=1		<=1	8	>2
VIII	2012-C06-018	Blood	87	<=4	>16	8		4	>16	>2
VIII	2012-C06-019	Blood	55	<=4	>16	<=1		<=1	4	<=0.06
VIII	2012-C06-026	Urine	55	<=4	>16	8		2	>16	>2
VIII	2012-C06-027	Urine	59	<=4	>16	<=1		<=1	<=2	>2

VIII	2012-C06-028	Urine	93	<=4	>16	16	>16	>16	>2
VIII	2012-C06-031	Blood	76	<=4	>16	<=1	<=1	16	<=0.06
VIII	2012-C06-035	Urine	84	<=4	<=2	<=1	<=1	<=2	<=0.06
VIII	2012-C06-036	Urine	54	<=4	>16	<=1	<=1	<=2	<=0.06
VIII	2012-C06-040	Urine	52	<=4	4	<=1	<=1	<=2	<=0.06
VIII	2012-C06-041	Other	73	<=4	>16	<=1	<=1	<=2	<=0.06
VIII	2012-C06-043	Urine	78	<=4	>16	<=1	<=1	>16	>2
VIII	2012-C06-044	Urine	86	<=4	>16	<=1	<=1	<=2	0.25
VIII	2012-C06-047	Urine	40	<=4	4	<=1	<=1	<=2	<=0.06
VIII	2012-C06-201	Blood	70	<=4	>16	8	4	>16	>2
VIII	2012-C06-202	Blood	76	<=4	>16	8	2	>16	>2
VIII	2012-C06-209	Blood	69	<=4	>16	4	16	>16	<=0.06
VIII	2012-C06-213	Blood	21	<=4	>16	<=1	<=1	8	0.25
VIII	2012-C06-219	Blood	69	<=4	8	<=1	4	16	>2
VIII	2012-C06-220	Blood	33	<=4	>16	16	2	>16	>2
VIII	2012-C06-235	Blood	66	<=4	>16	4	<=1	>16	>2
VIII	2012-C06-239	Blood	77	<=4	>16	<=1	<=1	16	>2
VIII	2012-C06-244	Blood	68	<=4	>16	<=1	<=1	4	<=0.06
VIII	2012-C06-245	Blood	78	<=4	<=2	<=1	<=1	<=2	<=0.06
VIII	2012-C06-246	Blood	73	<=4	>16	<=1	<=1	16	<=0.06
VIII	2012-C07-004	Blood	81	<=4	>16	4	<=1	>16	>2
VIII	2012-C07-005	Blood	47	<=4	>16	>16	16	>16	<=0.06
VIII	2012-C07-010	Blood	70	8	>16	>16	>16	>16	<=0.06
VIII	2012-C07-019	Blood	84	<=4	>16	<=1	<=1	8	0.25
VIII	2012-C07-024	Urine	56	<=4	<=2	<=1	<=1	<=2	<=0.06
VIII	2012-C07-027	Urine	25	<=4	>16	<=1	<=1	8	<=0.06
VIII	2012-C07-033	Urine	36	<=4	<=2	<=1	<=1	<=2	<=0.06
VIII	2012-C07-034	Blood	53	<=4	>16	<=1	<=1	<=2	<=0.06
VIII	2012-C07-040	Urine	69	<=4	>16	4	16	>16	0.5
VIII	2012-C07-043	Urine	0	<=4	>16	<=1	<=1	8	<=0.06
VIII	2012-C07-044	Urine	56	<=4	>16	<=1	<=1	<=2	0.12
VIII	2012-C07-048	Urine	66	8	>16	<=1	<=1	4	<=0.06
VIII	2012-C07-209	Blood	66	<=4	>16	>16	8	>16	<=0.06
VIII	2012-C07-214	Blood	57	<=4	>16	>16	>16	>16	>2
VIII	2012-C07-247	Blood	68	<=4	4	<=1	<=1	<=2	<=0.06
VIII	2012-C07-249	Other	35	<=4	>16	>16	8	>16	>2
VIII	2012-N16-001	Urine	60	<=4	<=2	<=1	<=1	<=2	<=0.06
VIII	2012-N16-002	Urine	68	<=4	>16	<=1	<=1	4	<=0.06
VIII	2012-N16-004	Urine	23	<=4	>16	<=1	<=1	<=2	<=0.06
VIII	2012-N16-005	Urine	47	<=4	>16	<=1	<=1	<=2	>2
VIII	2012-N16-006	Urine	70	<=4	<=2	<=1	<=1	<=2	<=0.06
VIII	2012-N16-008	Urine	74	<=4	>16	<=1	<=1	8	>2
VIII	2012-N16-011	Urine	78	<=4	4	<=1	<=1	<=2	0.25
VIII	2012-N16-013	Urine	29	<=4	>16	<=1	<=1	8	1
VIII	2012-N16-016	Urine	64	<=4	>16	8	2	>16	>2
VIII	2012-N16-018	Other	80	<=4	>16	<=1	<=1	8	>2
VIII	2012-N16-020	Urine	81	<=4	>16	<=1	<=1	4	0.12
VIII	2012-N16-024	Urine	77	<=4	>16	<=1	<=1	8	>2
VIII	2012-N16-026	Urine	93	<=4	>16	16	>16	>16	>2
VIII	2012-N16-030	Urine	79	<=4	>16	<=1	<=1	4	0.12
VIII	2012-N16-034	Urine	74	<=4	>16	<=1	<=1	<=2	0.5
VIII	2012-N16-042	Urine	84	<=4	>16	<=1	<=1	4	>2
VIII	2012-N16-043	Urine	71	<=4	>16	<=1	<=1	4	<=0.06
VIII	2012-N16-182	Urine	0	<=4	>16	<=1	<=1	<=2	<=0.06
VIII	2012-N16-190	Urine	0	<=4	>16	<=1	<=1	<=2	<=0.06
VIII	2012-N16-191	Urine	17	<=4	4	<=1	<=1	<=2	<=0.06
VIII	2012-N16-196	Urine	3	<=4	<=2	<=1	<=1	<=2	<=0.06
VIII	2012-N16-202	Blood	79	16	>16	8	4	>16	>2
VIII	2012-N16-210	Blood	39	<=4	>16	<=1	<=1	8	0.12
VIII	2012-N16-212	Blood	75	<=4	>16	<=1	<=1	<=2	0.25
VIII	2012-N16-213	Other	31	<=4	<=2	<=1	<=1	<=2	<=0.06

VIII	2012-N16-223	Blood	86	<=4	<=2	<=1		<=1	<=2	<=0.06
VIII	2012-N16-232	Other	60	<=4	>16	<=1		<=1	4	<=0.06
VIII	2012-N16-234	Other	34	<=4	>16	<=1		<=1	4	0.25
VIII	2012-N16-237	Blood	61	<=4	>16	<=1		<=1	<=2	>2
VIII	2012-N16-245	Other	49	<=4	<=2	<=1		<=1	<=2	0.25
VIII	2012-N17-002	Urine	90	8	4	<=1		<=1	4	<=0.06
VIII	2012-N17-005	Urine	6	<=4	>16	<=1		<=1	<=2	<=0.06
VIII	2012-N17-006	Urine	48	<=4	>16	<=1		<=1	4	<=0.06
VIII	2012-N17-007	Urine	38	<=4	>16	<=1		<=1	<=2	<=0.06
VIII	2012-N17-008	Urine	80	<=4	<=2	<=1		<=1	<=2	0.25
VIII	2012-N17-009	Urine	44	<=4	<=2	<=1		<=1	<=2	<=0.06
VIII	2012-N17-013	Urine	24	<=4	<=2	<=1		<=1	<=2	<=0.06
VIII	2012-N17-018	Urine	84	<=4	<=2	<=1		<=1	<=2	<=0.06
VIII	2012-N17-019	Urine	31	<=4	>16	8		4	>16	>2
VIII	2012-N17-020	Urine	70	<=4	<=2	<=1		<=1	<=2	<=0.06
VIII	2012-N17-021	Urine	2	<=4	<=2	<=1		<=1	<=2	<=0.06
VIII	2012-N17-022	Urine	0	<=4	>16	<=1		<=1	4	>2
VIII	2012-N17-024	Urine	72	<=4	>16	>16		4	>16	<=0.06
VIII	2012-N17-025	Urine	53	<=4	>16	<=1		<=1	<=2	0.12
VIII	2012-N17-027	Urine	37	<=4	>16	<=1		<=1	4	<=0.06
VIII	2012-N17-031	Urine	66	<=4	>16	<=1		<=1	4	<=0.06
VIII	2012-N17-032	Urine	78	8	>16	<=1		<=1	8	0.5
VIII	2012-N17-033	Urine	72	<=4	<=2	<=1		<=1	<=2	<=0.06
VIII	2012-N17-041	Urine	63	<=4	>16	<=1		<=1	16	>2
VIII	2012-N17-042	Urine	60	<=4	>16	<=1		<=1	4	<=0.06
VIII	2012-N17-044	Urine	40	<=4	<=2	<=1		<=1	<=2	>2
VIII	2012-N17-045	Urine	55	8	4	<=1		<=1	4	>2
VIII	2012-N17-046	Urine	64	<=4	4	<=1		<=1	<=2	<=0.06
VIII	2012-N17-048	Urine	80	<=4	>16	4		<=1	>16	>2
VIII	2012-N17-049	Urine	79	<=4	>16	>16		8	>16	>2
VIII	2012-C08-004	Other	82	<=4	>16	<=1		<=1	4	0.25
VIII	2012-C08-006	Urine	59	<=4	<=2	<=1		<=1	<=2	0.25
VIII	2012-C08-007	Urine	40	<=4	<=2	<=1		<=1	4	<=0.06
VIII	2012-C08-009	Urine	72	<=4	>16	<=1		<=1	8	>2
VIII	2012-C08-011	Urine	46	<=4	>16	<=1		<=1	4	<=0.06
VIII	2012-C08-016	Blood	68	<=4	<=2	<=1		<=1	<=2	<=0.06
VIII	2012-C08-017	Urine	51	<=4	4	<=1		<=1	<=2	>2
VIII	2012-C08-023	Blood	56	<=4	>16	<=1		<=1	8	>2
VIII	2012-C08-024	Urine	24	<=4	<=2	<=1		<=1	<=2	<=0.06
VIII	2012-C08-031	Other	61	<=4	>16	<=1		<=1	4	<=0.06
VIII	2012-C08-035	Blood	76	<=4	>16	<=1		<=1	4	<=0.06
VIII	2012-C08-040	Blood	62	<=4	>16	<=1		<=1	<=2	<=0.06
VIII	2012-C08-046	Other	75	<=4	>16	<=1		<=1	8	0.25
VIII	2012-C08-192	Urine	7	<=4	<=2	<=1		<=1	<=2	0.12
VIII	2012-C08-202	Blood	60	<=4	>16	8		8	>16	>2
VIII	2012-C08-212	Blood	48	<=4	>16	<=1		<=1	8	>2
VIII	2012-C08-214	Blood	63	<=4	>16	>16		>16	>16	0.5
VIII	2012-C08-227	Blood	89	<=4	>16	8		4	>16	>2
VIII	2012-C08-243	Blood	72	<=4	>16	<=1		<=1	4	<=0.06
VIII	2012-S11-002	Urine	61	<=4	>16	16		4	>16	0.12
VIII	2012-S11-003	Urine	72	<=4	>16	<=1		<=1	4	<=0.06
VIII	2012-S11-004	Urine	89	<=4	4	<=1		<=1	<=2	0.25
VIII	2012-S11-005	Urine	35	<=4	<=2	<=1		<=1	<=2	0.25
VIII	2012-S11-007	Urine	41	<=4	>16	<=1		<=1	>16	<=0.06
VIII	2012-S11-008	Urine	23	<=4	<=2	<=1		<=1	<=2	<=0.06
VIII	2012-S11-009	Blood	82	<=4	>16	<=1		<=1	4	<=0.06
VIII	2012-S11-011	Urine	73	<=4	>16	<=1		<=1	<=2	0.25
VIII	2012-S11-013	Urine	47	8	>16	<=1		<=1	8	<=0.06
VIII	2012-S11-014	Urine	85	<=4	>16	8		2	>16	>2
VIII	2012-S11-017	Urine	47	<=4	4	<=1		<=1	<=2	0.25
VIII	2012-S11-019	Urine	77	<=4	>16	<=1		<=1	4	0.25

VIII	2012-S11-020	Urine	57	<=4	4	<=1		<=1	4	<=0.06
VIII	2012-S11-021	Urine	36	<=4	>16	<=1		<=1	4	>2
VIII	2012-S11-028	Urine	88	<=4	>16	<=1		<=1	4	<=0.06
VIII	2012-S11-029	Urine	88	<=4	>16	<=1		<=1	8	<=0.06
VIII	2012-S11-030	Urine	74	<=4	>16	8		16	>16	>2
VIII	2012-S11-031	Urine	86	<=4	>16	8		16	>16	>2
VIII	2012-S11-033	Urine	69	<=4	<=2	<=1		<=1	<=2	<=0.06
VIII	2012-S11-036	Urine	74	<=4	>16	<=1		<=1	<=2	>2
VIII	2012-S11-039	Urine	76	<=4	>16	>16		8	>16	>2
VIII	2012-S11-046	Urine	86	<=4	>16	<=1		<=1	<=2	<=0.06
VIII	2012-S11-049	Blood	74	<=4	>16	<=1		<=1	>16	<=0.06
VIII	2012-S11-203	Blood	83	8	>16	<=1		<=1	4	>2
VIII	2012-S11-206	Blood	81	<=4	>16	<=1		<=1	4	0.12
VIII	2012-S11-215	Blood	74	<=4	<=2	<=1		<=1	<=2	<=0.06
VIII	2012-S11-222	Blood	86	<=4	<=2	<=1		<=1	<=2	<=0.06
VIII	2012-S11-223	Blood	82	<=4	>16	<=1		<=1	8	<=0.06
VIII	2012-S11-224	Blood	41	<=4	<=2	<=1		<=1	<=2	<=0.06
VIII	2012-S11-226	Blood	48	<=4	>16	4		8	>16	<=0.06
VIII	2012-S11-231	Blood	84	<=4	>16	<=1		<=1	4	0.25
VIII	2012-S11-233	Blood	48	<=4	>16	>16		8	>16	>2
VIII	2012-S11-234	Blood	62	<=4	>16	<=1		<=1	<=2	>2
VIII	2012-S11-235	Blood	84	<=4	<=2	<=1		<=1	<=2	>2
VIII	2012-S11-238	Blood	75	<=4	>16	<=1		<=1	4	<=0.06
VIII	2012-S11-239	Blood	64	<=4	>16	<=1		<=1	4	<=0.06
VIII	2012-S11-245	Blood	53	<=4	4	<=1		<=1	<=2	<=0.06
VIII	2012-S11-246	Blood	80	<=4	>16	>16		16	>16	>2
VIII	2012-N23-001	Urine	31	<=4	>16	<=1		<=1	4	0.12
VIII	2012-N23-003	Urine	50	<=4	<=2	<=1		<=1	<=2	<=0.06
VIII	2012-N23-004	Urine	35	<=4	>16	<=1		<=1	4	<=0.06
VIII	2012-N23-005	Urine	85	<=4	4	<=1		<=1	4	>2
VIII	2012-N23-006	Urine	90	<=4	<=2	<=1		<=1	<=2	<=0.06
VIII	2012-N23-007	Urine	53	<=4	4	<=1		<=1	<=2	<=0.06
VIII	2012-N23-018	Urine	51	<=4	>16	<=1		<=1	8	<=0.06
VIII	2012-N23-019	Urine	43	<=4	>16	<=1		<=1	8	<=0.06
VIII	2012-N23-020	Urine	46	<=4	4	<=1		<=1	<=2	<=0.06
VIII	2012-N23-021	Urine	71	<=4	4	<=1		<=1	<=2	>2
VIII	2012-N23-022	Urine	74	<=4	>16	<=1		<=1	4	>2
VIII	2012-N23-024	Urine	25	<=4	4	<=1		<=1	<=2	<=0.06
VIII	2012-N23-027	Urine	57	<=4	>16	<=1		<=1	<=2	<=0.06
VIII	2012-N23-029	Urine	59	<=4	<=2	<=1		<=1	<=2	<=0.06
VIII	2012-N23-030	Urine	88	<=4	>16	<=1		<=1	8	0.25
VIII	2012-N23-031	Urine	68	<=4	4	<=1		<=1	<=2	<=0.06
VIII	2012-N23-032	Urine	89	<=4	>16	<=1		<=1	4	<=0.06
VIII	2012-N23-033	Urine	79	<=4	4	<=1		<=1	<=2	>2
VIII	2012-N23-034	Urine	78	<=4	<=2	<=1		<=1	<=2	<=0.06
VIII	2012-N23-036	Urine	69	<=4	>16	<=1		<=1	<=2	0.25
VIII	2012-N23-037	Urine	78	<=4	4	<=1		<=1	<=2	<=0.06
VIII	2012-N23-039	Urine	69	<=4	4	<=1		<=1	<=2	<=0.06
VIII	2012-N23-041	Urine	80	<=4	<=2	<=1		<=1	<=2	<=0.06
VIII	2012-N23-042	Urine	6	<=4	>16	8		>16	>16	<=0.06
VIII	2012-N23-043	Urine	68	<=4	>16	<=1		<=1	4	<=0.06
VIII	2012-N23-044	Urine	60	<=4	>16	<=1		<=1	>16	>2
VIII	2012-N23-045	Urine	27	<=4	>16	<=1		<=1	8	0.25
VIII	2012-N23-046	Urine	8	<=4	4	<=1		<=1	<=2	0.12
VIII	2012-N23-047	Urine	73	<=4	>16	<=1		<=1	<=2	>2
VIII	2012-N23-048	Urine	97	<=4	<=2	<=1		<=1	<=2	<=0.06
VIII	2012-N23-219	Blood	22	<=4	>16	<=1		<=1	4	<=0.06
VIII	2012-N23-243	Blood	64	<=4	>16	<=1		<=1	4	<=0.06
VIII	2012-N23-250	Blood	76	<=4	4	<=1		<=1	<=2	<=0.06
VIII	2012-N21-013	Urine	81	<=4	>16	<=1		<=1	4	1
VIII	2012-N21-014	Urine	95	<=4	>16	>16		>16	>16	>2

VIII	2012-N21-015	Urine	25	<=4	>16	>16	16	>16	>2
VIII	2012-N21-017	Urine	83	<=4	>16	>16	16	>16	<=0.06
VIII	2012-N21-021	Urine	80	<=4	>16	<=1	<=1	4	1
VIII	2012-N21-022	Urine	21	<=4	>16	<=1	<=1	8	<=0.06
VIII	2012-N21-029	Urine	96	<=4	>16	<=1	<=1	4	<=0.06
VIII	2012-N21-032	Urine	71	8	>16	<=1	<=1	4	>2
VIII	2012-N21-033	Urine	1	<=4	>16	<=1	<=1	4	<=0.06
VIII	2012-N21-034	Urine	46	<=4	>16	<=1	<=1	4	<=0.06
VIII	2012-N21-036	Urine	57	<=4	4	<=1	<=1	<=2	<=0.06
VIII	2012-N21-037	Urine	28	<=4	4	<=1	<=1	<=2	<=0.06
VIII	2012-N21-042	Urine	36	<=4	4	<=1	<=1	<=2	<=0.06
VIII	2012-N21-046	Urine	0	<=4	>16	<=1	<=1	<=2	<=0.06
VIII	2012-N21-047	Urine	57	8	<=2	<=1	<=1	<=2	<=0.06
VIII	2012-N21-048	Other	73	<=4	>16	>16	>16	>16	>2
VIII	2012-N21-189	Blood	0	<=4	4	<=1	<=1	<=2	0.12
VIII	2012-N21-216	Blood	72	8	>16	>16	>16	>16	<=0.06
VIII	2012-N21-218	Blood	50	<=4	>16	4	>16	>16	0.5
VIII	2012-N21-219	Blood	48	<=4	>16	<=1	<=1	4	<=0.06
VIII	2012-N21-221	Blood	76	16	>16	2	<=1	>16	>2
VIII	2012-N21-223	Blood	77	<=4	>16	<=1	<=1	4	<=0.06
VIII	2012-N21-224	Blood	70	<=4	>16	<=1	<=1	<=2	0.25
VIII	2012-N21-225	Blood	80	<=4	>16	<=1	<=1	4	<=0.06
VIII	2012-N21-229	Blood	57	<=4	>16	<=1	<=1	<=2	<=0.06
VIII	2012-N21-230	Blood	85	<=4	>16	<=1	<=1	4	>2
VIII	2012-N21-235	Blood	39	8	4	<=1	<=1	<=2	<=0.06
VIII	2012-N21-236	Blood	56	<=4	>16	<=1	<=1	8	<=0.06
VIII	2012-N21-238	Blood	78	<=4	>16	<=1	<=1	4	<=0.06
VIII	2012-N21-239	Blood	62	<=4	>16	8	16	>16	>2
VIII	2012-N21-240	Blood	84	<=4	>16	<=1	<=1	8	<=0.06
VIII	2012-N21-241	Blood	27	<=4	<=2	<=1	<=1	4	0.25
VIII	2012-N21-243	Blood	65	<=4	<=2	<=1	<=1	<=2	<=0.06
VIII	2012-N21-245	Blood	91	<=4	>16	<=1	<=1	4	<=0.06
VIII	2012-N21-248	Blood	56	<=4	>16	8	16	>16	>2
VIII	2012-N21-249	Blood	81	<=4	>16	<=1	<=1	8	<=0.06
VIII	2012-C09-001	Blood	38	<=4	<=2	<=1	<=1	<=2	<=0.06
VIII	2012-C09-003	Blood	58	<=4	>16	<=1	<=1	4	<=0.06
VIII	2012-C09-004	Blood	87	<=4	>16	16	>16	>16	>2
VIII	2012-C09-005	Blood	81	<=4	<=2	<=1	<=1	<=2	<=0.06
VIII	2012-C09-012	Urine	58	<=4	>16	<=1	<=1	<=2	0.25
VIII	2012-C09-013	Urine	56	8	<=2	<=1	<=1	<=2	<=0.06
VIII	2012-C09-014	Urine	49	<=4	>16	<=1	<=1	<=2	<=0.06
VIII	2012-C09-018	Urine	81	<=4	>16	<=1	<=1	16	>2
VIII	2012-C09-024	Urine	80	8	>16	>16	>16	>16	>2
VIII	2012-C09-025	Urine	85	<=4	>16	<=1	<=1	<=2	0.25
VIII	2012-C09-027	Urine	70	<=4	>16	<=1	<=1	16	>2
VIII	2012-C09-028	Urine	52	<=4	4	<=1	<=1	<=2	<=0.06
VIII	2012-C09-034	Urine	66	<=4	4	<=1	<=1	<=2	0.25
VIII	2012-C09-037	Urine	79	<=4	<=2	<=1	<=1	<=2	<=0.06
VIII	2012-C09-039	Urine	1	<=4	>16	<=1	<=1	4	<=0.06
VIII	2012-C09-040	Other	84	<=4	>16	>16	8	>16	>2
VIII	2012-C09-046	Urine	74	<=4	4	<=1	2	<=2	<=0.06
VIII	2012-C09-047	Urine	77	<=4	4	<=1	<=1	4	>2
VIII	2012-C09-049	Other	19	<=4	>16	<=1	<=1	<=2	>2
VIII	2012-C09-203	Other	34	8	>16	<=1	<=1	<=2	<=0.06
VIII	2012-C09-206	Blood	76	<=4	<=2	<=1	<=1	<=2	<=0.06
VIII	2012-C09-222	Blood	65	<=4	>16	<=1	<=1	4	1
VIII	2012-C09-235	Blood	81	<=4	>16	<=1	<=1	4	2
VIII	2012-C09-240	Blood	77	<=4	>16	<=1	<=1	<=2	<=0.06
VIII	2012-C09-245	Blood	81	<=4	>16	8	2	>16	>2
VIII	2012-C09-248	Other	70	<=4	<=2	<=1	<=1	<=2	<=0.06
VIII	2012-C09-249	Blood	73	<=4	>16	<=1	<=1	<=2	0.25

VIII	2012-C09-250	Blood	56	<=4	4	<=1		<=1	<=2	<=0.06
VIII	2012-C10-004	Urine	44	<=4	<=2	<=1		<=1	4	<=0.06
VIII	2012-C10-005	Other	21	<=4	>16	<=1		<=1	<=2	<=0.06
VIII	2012-C10-007	Urine	50	<=4	<=2	<=1		<=1	<=2	<=0.06
VIII	2012-C10-010	Urine	69	<=4	<=2	<=1		<=1	<=2	0.25
VIII	2012-C10-011	Urine	76	<=4	<=2	<=1		<=1	<=2	<=0.06
VIII	2012-C10-012	Urine	22	<=4	<=2	<=1		<=1	<=2	<=0.06
VIII	2012-C10-013	Urine	80	<=4	>16	>16		>16	>16	>2
VIII	2012-C10-022	Urine	54	<=4	4	<=1		<=1	<=2	<=0.06
VIII	2012-C10-023	Urine	82	<=4	>16	<=1		<=1	4	>2
VIII	2012-C10-024	Blood	87	<=4	4	<=1		<=1	<=2	<=0.06
VIII	2012-C10-027	Urine	93	<=4	>16	2		2	>16	<=0.06
VIII	2012-C10-031	Urine	88	<=4	>16	<=1		<=1	4	>2
VIII	2012-C10-032	Urine	88	<=4	>16	<=1		<=1	8	>2
VIII	2012-C10-034	Urine	61	<=4	>16	<=1		<=1	8	<=0.06
VIII	2012-C10-035	Urine	83	<=4	>16	<=1		<=1	>16	>2
VIII	2012-C10-046	Urine	73	<=4	>16	<=1		<=1	<=2	<=0.06
VIII	2012-C10-047	Urine	71	<=4	>16	16		>16	>16	>2
VIII	2012-C10-048	Urine	73	<=4	4	<=1		<=1	<=2	<=0.06
VIII	2012-C10-189	Urine	7	<=4	<=2	<=1		<=1	<=2	<=0.06
VIII	2012-C10-200	Urine	2	<=4	>16	<=1		<=1	4	0.25
VIII	2012-C10-201	Blood	85	<=4	>16	<=1		<=1	<=2	<=0.06
VIII	2012-C10-207	Blood	59	<=4	>16	<=1		<=1	8	<=0.06
VIII	2012-C10-208	Blood	47	<=4	>16	<=1		<=1	8	<=0.06
VIII	2012-C10-212	Blood	84	<=4	>16	>16		16	>16	>2
VIII	2012-C10-213	Blood	56	<=4	>16	<=1		<=1	4	<=0.06
VIII	2012-C10-214	Blood	68	<=4	>16	<=1		<=1	<=2	<=0.06
VIII	2012-C10-215	Blood	58	<=4	>16	<=1		<=1	>16	<=0.06
VIII	2012-C10-219	Blood	19	<=4	>16	<=1		<=1	<=2	<=0.06
VIII	2012-C10-222	Blood	15	<=4	>16	<=1		<=1	4	0.25
VIII	2012-C10-223	Blood	60	<=4	>16	<=1		<=1	4	<=0.06
VIII	2012-C10-238	Blood	78	<=4	>16	<=1		<=1	16	<=0.06
VIII	2012-C10-243	Blood	71	<=4	>16	<=1		<=1	4	<=0.06
VIII	2012-C10-244	Blood	82	<=4	>16	<=1		<=1	<=2	<=0.06
VIII	2012-C10-245	Blood	77	<=4	>16	<=1		<=1	8	>2
VIII	2012-C10-246	Blood	69	<=4	>16	<=1		<=1	4	2
VIII	2012-E04-002	Blood	91	<=4	>16	<=1		<=1	<=2	0.12
VIII	2012-E04-009	Blood	68	<=4	>16	<=1		<=1	16	>2
VIII	2012-E04-013	Blood	66	<=4	>16	4		>16	>16	<=0.06
VIII	2012-E04-020	Blood	74	<=4	>16	<=1		<=1	>16	>2
VIII	2012-E04-031	Blood	76	<=4	4	<=1		<=1	<=2	<=0.06
VIII	2012-E04-052	Blood	84	<=4	>16	<=1		<=1	8	<=0.06
VIII	2012-E04-067	Blood	75	<=4	>16	<=1		<=1	4	<=0.06
VIII	2012-E04-068	Blood	54	<=4	>16	<=1		<=1	8	>2
VIII	2012-E04-088	Blood	0	<=4	4	<=1		<=1	<=2	<=0.06
VIII	2012-E04-090	Blood	75	<=4	>16	<=1		<=1	4	<=0.06
VIII	2012-E04-100	Blood	75	<=4	>16	>16		4	>16	0.25
VIII	2012-E04-131	Blood	72	<=4	>16	>16		>16	>16	>2
VIII	2012-E04-136	Blood	56	<=4	>16	<=1		<=1	4	<=0.06
VIII	2012-E04-150	Blood	98	<=4	>16	<=1		<=1	4	<=0.06
VIII	2012-E04-171	Blood	71	<=4	<=2	<=1		<=1	<=2	0.12
VIII	2012-E04-173	Blood	21	<=4	>16	<=1		<=1	4	<=0.06
VIII	2012-E04-181	Blood	58	<=4	>16	8		16	>16	<=0.06
VIII	2012-E04-194	Blood	46	<=4	4	<=1		<=1	4	<=0.06
VIII	2012-E04-206	Blood	56	<=4	>16	<=1		<=1	<=2	<=0.06
VIII	2012-E04-209	Blood	77	<=4	<=2	<=1		<=1	<=2	>2
VIII	2012-E04-224	Blood	72	<=4	>16	<=1		<=1	4	0.25
VIII	2012-E04-231	Blood	72	<=4	>16	<=1		<=1	4	<=0.06
VIII	2012-E04-232	Blood	78	<=4	>16	2		<=1	>16	>2
VIII	2012-E04-246	Blood	55	<=4	4	<=1		<=1	<=2	<=0.06
VIII	2012-E04-279	Blood	89	<=4	>16	<=1		<=1	16	<=0.06

VIII	2012-E04-283	Blood	75	<=4	>16	16		>16	>16	>2
VIII	2012-E04-285	Blood	66	<=4	>16	<=1		<=1	4	0.25
VIII	2012-E04-287	Blood	57	<=4	>16	<=1		<=1	16	<=0.06
VIII	2012-E04-292	Blood	24	<=4	>16	<=1		<=1	4	0.12
VIII	2012-E04-303	Blood	58	<=4	>16	<=1		<=1	8	0.25
VIII	2012-E04-308	Blood	85	<=4	>16	8		16	>16	<=0.06
VIII	2012-E04-314	Blood	63	<=4	>16	8		2	>16	<=0.06
VIII	2012-E04-315	Blood	63	<=4	<=2	<=1		<=1	<=2	<=0.06
VIII	2012-E04-340	Blood	61	<=4	>16	<=1		<=1	<=2	0.12
VIII	2012-E04-341	Blood	81	<=4	>16	<=1		<=1	16	<=0.06

EPT_M	FEP_M	FOS_M	FOX_M	FRX_M	FTX_M	FUR_M	GEN_M	IMP_M	PIP_M	SXT_M
	<=0.5		<=2	4	<=0.5	<=32	1	<=0.25	<=4	<=0.5
	<=0.5		8	16	4	<=32	>8	<=0.25	>64	>2
	<=0.5		>16	>16	8	<=32	1	<=0.25	>64	>2
	<=0.5		<=2	4	<=0.5	<=32	>8	<=0.25	>64	>2
	<=0.5		8	8	<=0.5	64	<=0.5	<=0.25	<=4	<=0.5
	<=0.5		8	8	<=0.5	<=32	1	<=0.25	>64	>2
	<=0.5		<=2	2	<=0.5	<=32	>8	<=0.25	>64	>2
	<=0.5		8	8	<=0.5	<=32	<=0.5	<=0.25	<=4	<=0.5
	<=0.5		<=2	8	<=0.5	<=32	<=0.5	<=0.25	>64	<=0.5
	<=0.5		4	4	<=0.5	<=32	<=0.5	<=0.25	<=4	>2
	<=0.5		<=2	4	<=0.5	<=32	<=0.5	<=0.25	>64	>2
	<=0.5		<=2	2	<=0.5	<=32	<=0.5	<=0.25	<=4	<=0.5
	<=0.5		4	4	<=0.5	<=32	<=0.5	<=0.25	32	<=0.5
	<=0.5		<=2	2	<=0.5	<=32	<=0.5	<=0.25	>64	>2
	<=0.5		<=2	4	<=0.5	<=32	<=0.5	<=0.25	>64	>2
	<=0.5		4	4	<=0.5	<=32	<=0.5	<=0.25	>64	>2
	<=0.5		4	4	<=0.5	>64	>8	<=0.25	32	>2
	<=0.5		4	2	<=0.5	<=32	<=0.5	<=0.25	>64	<=0.5
	<=0.5		>16	>16	<=0.5	<=32	4	0.5	>64	>2
	<=0.5		<=2	2	<=0.5	<=32	<=0.5	<=0.25	>64	>2
	<=0.5		<=2	2	<=0.5	<=32	1	<=0.25	>64	<=0.5
	<=0.5		8	4	<=0.5	<=32	1	<=0.25	<=4	<=0.5
	<=0.5		4	4	<=0.5	<=32	<=0.5	<=0.25	<=4	<=0.5
	<=0.5		4	4	<=0.5	<=32	1	<=0.25	>64	<=0.5
	<=0.5		>16	>16	8	<=32	<=0.5	0.5	>64	>2
	<=0.5		<=2	2	<=0.5	<=32	<=0.5	<=0.25	<=4	<=0.5
	<=0.5		4	8	<=0.5	<=32	<=0.5	<=0.25	64	>2
	<=0.5		4	4	<=0.5	<=32	<=0.5	<=0.25	>64	>2
	<=0.5		8	4	<=0.5	<=32	1	<=0.25	<=4	<=0.5
	<=0.5		4	4	<=0.5	<=32	>8	<=0.25	>64	>2
	<=0.5		<=2	4	<=0.5	<=32	<=0.5	<=0.25	>64	<=0.5
	<=0.5		<=2	2	<=0.5	<=32	<=0.5	<=0.25	<=4	<=0.5
	<=0.5		<=2	4	<=0.5	<=32	<=0.5	<=0.25	<=4	<=0.5
	<=0.5		4	8	<=0.5	<=32	1	<=0.25	>64	>2
	<=0.5		<=2	8	<=0.5	<=32	<=0.5	<=0.25	>64	<=0.5
	<=0.5		<=2	4	<=0.5	<=32	<=0.5	<=0.25	>64	>2
	<=0.5		<=2	2	<=0.5	<=32	<=0.5	<=0.25	>64	<=0.5
	<=0.5		4	4	<=0.5	<=32	>8	<=0.25	>64	>2
	<=0.5		8	4	<=0.5	<=32	1	<=0.25	<=4	>2
	<=0.5		4	8	<=0.5	64	1	<=0.25	<=4	<=0.5
	<=0.5		4	4	<=0.5	64	1	<=0.25	<=4	<=0.5
	<=0.5		16	8	<=0.5	<=32	<=0.5	<=0.25	<=4	1
	<=0.5		<=2	4	<=0.5	<=32	<=0.5	<=0.25	>64	<=0.5
	16		>16	>16	>32	<=32	>8	<=0.25	>64	>2
	<=0.5		8	4	<=0.5	<=32	1	<=0.25	>64	>2
	<=0.5		4	4	<=0.5	64	>8	<=0.25	>64	>2
	<=0.5		4	2	<=0.5	<=32	>8	<=0.25	>64	>2
	<=0.5		8	8	<=0.5	<=32	<=0.5	<=0.25	<=4	<=0.5
	<=0.5		4	4	<=0.5	<=32	1	<=0.25	<=4	>2
	<=0.5		4	4	<=0.5	<=32	<=0.5	<=0.25	<=4	<=0.5
	<=0.5		8	16	<=0.5	64	1	<=0.25	>64	>2
	<=0.5		4	4	<=0.5	<=32	<=0.5	<=0.25	>64	>2
	<=0.5		<=2	4	<=0.5	<=32	<=0.5	<=0.25	<=4	<=0.5
	<=0.5		<=2	4	<=0.5	<=32	<=0.5	<=0.25	>64	>2
	<=0.5		4	4	<=0.5	<=32	8	<=0.25	64	>2

<=0.5	>16	>16	8	<=32	>8	0.5	>64	>2
<=0.5	<=2	4	<=0.5	>64	<=0.5	<=0.25	>64	>2
<=0.5	8	4	<=0.5	<=32	>8	<=0.25	>64	>2
<=0.5	8	8	<=0.5	<=32	>8	<=0.25	64	>2
<=0.5	<=2	4	<=0.5	<=32	1	<=0.25	64	>2
<=0.5	8	4	<=0.5	<=32	1	<=0.25	64	>2
<=0.5	<=2	4	<=0.5	<=32	>8	<=0.25	16	<=0.5
<=0.5	8	4	<=0.5	<=32	>8	<=0.25	>64	>2
<=0.5	4	4	<=0.5	<=32	<=0.5	<=0.25	8	>2
<=0.5	4	4	<=0.5	>64	<=0.5	<=0.25	>64	>2
<=0.5	8	8	<=0.5	<=32	>8	<=0.25	64	<=0.5
<=0.5	4	4	<=0.5	<=32	<=0.5	<=0.25	64	>2
<=0.5	4	4	<=0.5	<=32	>8	<=0.25	>64	>2
<=0.5	4	4	<=0.5	<=32	1	<=0.25	<=4	<=0.5
<=0.5	4	4	<=0.5	<=32	<=0.5	<=0.25	32	>2
<=0.5	<=2	2	<=0.5	<=32	<=0.5	<=0.25	32	>2
<=0.5	8	8	<=0.5	<=32	<=0.5	<=0.25	>64	>2
<=0.5	4	4	<=0.5	<=32	1	<=0.25	<=4	<=0.5
<=0.5	4	4	<=0.5	<=32	>8	<=0.25	64	>2
<=0.5	4	4	<=0.5	<=32	<=0.5	<=0.25	<=4	<=0.5
<=0.5	<=2	4	<=0.5	<=32	<=0.5	<=0.25	64	>2
<=0.5	4	4	<=0.5	<=32	>8	<=0.25	>64	>2
<=0.5	4	2	<=0.5	<=32	4	<=0.25	<=4	<=0.5
<=0.5	4	4	<=0.5	<=32	1	<=0.25	<=4	<=0.5
<=0.5	4	2	<=0.5	<=32	<=0.5	<=0.25	<=4	1
<=0.5	4	8	<=0.5	<=32	<=0.5	<=0.25	<=4	<=0.5
<=0.5	4	4	<=0.5	<=32	<=0.5	<=0.25	>64	>2
<=0.5	<=2	2	<=0.5	<=32	4	<=0.25	64	>2
<=0.5	>16	>16	8	<=32	<=0.5	<=0.25	>64	>2
<=0.5	<=2	2	<=0.5	<=32	<=0.5	<=0.25	>64	>2
<=0.5	8	4	<=0.5	>64	1	<=0.25	<=4	<=0.5
<=0.5	<=2	4	<=0.5	<=32	1	<=0.25	<=4	<=0.5
<=0.5	4	4	<=0.5	<=32	>8	<=0.25	>64	>2
<=0.5	<=2	2	<=0.5	64	<=0.5	<=0.25	>64	<=0.5
<=0.5	4	8	<=0.5	<=32	>8	<=0.25	32	>2
<=0.5	8	8	<=0.5	<=32	<=0.5	<=0.25	32	<=0.5
<=0.5	4	4	<=0.5	<=32	1	<=0.25	<=4	<=0.5
<=0.5	4	4	<=0.5	<=32	<=0.5	<=0.25	<=4	<=0.5
<=0.5	4	4	<=0.5	<=32	<=0.5	<=0.25	32	<=0.5
<=0.5	<=2	4	<=0.5	<=32	<=0.5	<=0.25	>64	>2
<=0.5	8	8	<=0.5	<=32	<=0.5	<=0.25	<=4	<=0.5
<=0.5	4	4	<=0.5	<=32	1	<=0.25	>64	>2
<=0.5	4	4	<=0.5	<=32	>8	<=0.25	>64	>2
<=0.5	4	4	<=0.5	<=32	<=0.5	<=0.25	64	>2
<=0.5	4	8	<=0.5	<=32	1	<=0.25	64	<=0.5
<=0.5	4	4	<=0.5	<=32	<=0.5	<=0.25	<=4	<=0.5
<=0.5	<=2	4	<=0.5	<=32	1	<=0.25	<=4	<=0.5
<=0.5	<=2	4	<=0.5	<=32	<=0.5	<=0.25	<=4	<=0.5
<=0.5	4	4	<=0.5	<=32	<=0.5	<=0.25	>64	>2
2	8	>16	>32	<=32	>8	<=0.25	>64	>2
<=0.5	<=2	2	<=0.5	<=32	<=0.5	<=0.25	>64	>2
<=0.5	<=2	2	<=0.5	>64	>8	<=0.25	<=4	1
<=0.5	16	8	<=0.5	<=32	4	<=0.25	>64	>2
<=0.5	4	4	<=0.5	<=32	<=0.5	<=0.25	<=4	<=0.5
<=0.5	<=2	4	<=0.5	<=32	<=0.5	<=0.25	>64	<=0.5
<=0.5	4	4	<=0.5	<=32	<=0.5	<=0.25	>64	>2
<=0.5	<=2	2	<=0.5	<=32	<=0.5	<=0.25	<=4	<=0.5
<=0.5	4	4	<=0.5	<=32	<=0.5	<=0.25	<=4	<=0.5
<=0.5	16	8	<=0.5	64	>8	<=0.25	64	>2
<=0.5	>16	>16	16	<=32	1	<=0.25	64	<=0.5
<=0.5	4	4	<=0.5	<=32	1	<=0.25	<=4	<=0.5

	<=0.5	4	4	<=0.5	<=32	<=0.5	<=0.25	64	>2
	<=0.5	<=2	2	<=0.5	<=32	<=0.5	<=0.25	>64	<=0.5
	<=0.5	4	4	<=0.5	<=32	>8	<=0.25	>64	>2
	<=0.5	<=2	4	<=0.5	<=32	1	<=0.25	>64	>2
	<=0.5	16	8	<=0.5	<=32	<=0.5	<=0.25	<=4	<=0.5
	>16	16	>16	>32	<=32	>8	<=0.25	>64	>2
	4	4	>16	16	<=32	8	<=0.25	>64	>2
	<=0.5	4	4	<=0.5	<=32	<=0.5	<=0.25	>64	<=0.5
>4	2	>16	>16	32	>64	>8	4	>64	>2
	1	>16	>16	32	>64	>8	0.5	>64	>2
	<=0.5	8	4	<=0.5	<=32	<=0.5	<=0.25	>64	<=0.5
	<=0.5	4	4	<=0.5	<=32	<=0.5	<=0.25	>64	<=0.5
	<=0.5	<=2	4	<=0.5	<=32	<=0.5	<=0.25	>64	>2
	<=0.5	8	8	<=0.5	<=32	<=0.5	<=0.25	>64	>2
	<=0.5	<=2	4	<=0.5	<=32	<=0.5	<=0.25	<=4	>2
	<=0.5	4	4	<=0.5	<=32	2	<=0.25	<=4	<=0.5
	<=0.5	<=2	<=1	<=0.5	<=32	>8	<=0.25	>64	>2
	<=0.5	<=2	2	<=0.5	<=32	<=0.5	<=0.25	>64	>2
	<=0.5	<=2	2	<=0.5	<=32	<=0.5	<=0.25	>64	>2
	<=0.5	4	4	<=0.5	<=32	2	<=0.25	<=4	<=0.5
	<=0.5	<=2	2	<=0.5	<=32	2	<=0.25	<=4	<=0.5
	<=0.5	4	8	<=0.5	<=32	1	<=0.25	>64	>2
	<=0.5	4	8	<=0.5	<=32	>8	<=0.25	>64	>2
	>16	16	>16	>32	64	>8	<=0.25	>64	>2
	<=0.5	4	4	<=0.5	<=32	1	<=0.25	16	<=0.5
	<=0.5	<=2	4	<=0.5	<=32	1	<=0.25	>64	>2
	<=0.5	<=2	2	<=0.5	<=32	>8	<=0.25	>64	>2
	<=0.5	<=2	4	<=0.5	<=32	<=0.5	<=0.25	>64	>2
	<=0.5	<=2	2	<=0.5	<=32	<=0.5	<=0.25	>64	>2
	<=0.5	4	4	<=0.5	<=32	>8	<=0.25	>64	>2
	<=0.5	8	16	<=0.5	<=32	>8	<=0.25	>64	>2
	<=0.5	16	16	<=0.5	>64	>8	<=0.25	<=4	>2
	<=0.5	>16	>16	8	<=32	>8	<=0.25	>64	>2
	<=0.5	<=2	<=1	<=0.5	<=32	<=0.5	<=0.25	<=4	<=0.5
	<=0.5	8	8	<=0.5	<=32	<=0.5	<=0.25	<=4	<=0.5
	<=0.5	8	8	<=0.5	<=32	1	<=0.25	>64	>2
	<=0.5	4	4	<=0.5	<=32	<=0.5	<=0.25	<=4	<=0.5
	<=0.5	4	4	<=0.5	<=32	<=0.5	<=0.25	<=4	<=0.5
	<=0.5	<=2	4	<=0.5	<=32	<=0.5	<=0.25	>64	<=0.5
	<=0.5	<=2	2	<=0.5	<=32	1	<=0.25	>64	<=0.5
	<=0.5	4	4	<=0.5	<=32	1	<=0.25	<=4	<=0.5
	<=0.5	<=2	4	<=0.5	<=32	<=0.5	<=0.25	64	>2
	<=0.5	8	8	<=0.5	<=32	<=0.5	<=0.25	<=4	<=0.5
	<=0.5	<=2	8	<=0.5	<=32	<=0.5	<=0.25	64	>2
	<=0.5	4	4	<=0.5	<=32	1	<=0.25	16	>2
	<=0.5	4	8	<=0.5	<=32	1	<=0.25	>64	>2
	<=0.5	4	4	<=0.5	<=32	<=0.5	<=0.25	>64	<=0.5
	<=0.5	<=2	8	<=0.5	<=32	<=0.5	<=0.25	64	<=0.5
	<=0.5	8	4	<=0.5	<=32	<=0.5	<=0.25	64	>2
	<=0.5	<=2	<=1	<=0.5	<=32	<=0.5	<=0.25	<=4	<=0.5
	<=0.5	4	4	<=0.5	<=32	1	<=0.25	>64	>2
	2	4	>16	16	<=32	>8	1	>64	>2
	<=0.5	8	8	<=0.5	<=32	4	<=0.25	>64	>2
	<=0.5	16	16	<=0.5	<=32	<=0.5	<=0.25	8	<=0.5
	<=0.5	4	4	<=0.5	<=32	<=0.5	<=0.25	<=4	>2
	<=0.5	8	4	<=0.5	<=32	1	<=0.25	>64	>2
	<=0.5	4	4	<=0.5	<=32	<=0.5	<=0.25	<=4	<=0.5
	<=0.5	<=2	4	<=0.5	>64	<=0.5	<=0.25	>64	>2
	<=0.5	<=2	4	<=0.5	<=32	1	<=0.25	>64	>2
	<=0.5	<=2	4	<=0.5	<=32	<=0.5	<=0.25	>64	<=0.5
	1	16	>16	8	64	>8	<=0.25	64	>2

<=0.5	4	4	<=0.5	<=32	1	<=0.25	<=4	<=0.5
<=0.5	4	4	<=0.5	<=32	<=0.5	<=0.25	<=4	<=0.5
<=0.5	4	8	<=0.5	<=32	<=0.5	<=0.25	64	>2
<=0.5	4	4	<=0.5	<=32	<=0.5	<=0.25	<=4	<=0.5
<=0.5	<=2	<=1	<=0.5	<=32	<=0.5	<=0.25	<=4	<=0.5
<=0.5	<=2	4	<=0.5	<=32	>8	<=0.25	>64	>2
<=0.5	4	4	<=0.5	<=32	>8	<=0.25	<=4	<=0.5
<=0.5	4	4	<=0.5	<=32	<=0.5	<=0.25	>64	>2
<=0.5	4	8	<=0.5	64	8	<=0.25	<=4	<=0.5
<=0.5	8	8	<=0.5	64	<=0.5	<=0.25	>64	>2
<=0.5	<=2	<=1	<=0.5	<=32	<=0.5	<=0.25	32	>2
<=0.5	<=2	2	<=0.5	<=32	<=0.5	<=0.25	>64	>2
<=0.5	<=2	4	<=0.5	<=32	<=0.5	<=0.25	<=4	<=0.5
<=0.5	<=2	2	<=0.5	<=32	>8	<=0.25	<=4	<=0.5
<=0.5	4	8	<=0.5	<=32	>8	<=0.25	32	>2
<=0.5	8	8	<=0.5	>64	>8	<=0.25	<=4	>2
<=0.5	4	4	<=0.5	<=32	1	<=0.25	<=4	<=0.5
<=0.5	<=2	2	<=0.5	<=32	<=0.5	<=0.25	>64	>2
<=0.5	<=2	8	<=0.5	<=32	<=0.5	<=0.25	>64	>2
<=0.5	<=2	2	<=0.5	<=32	1	<=0.25	>64	<=0.5
<=0.5	4	4	<=0.5	<=32	>8	<=0.25	>64	<=0.5
<=0.5	>16	>16	8	<=32	>8	<=0.25	>64	>2
<=0.5	<=2	4	<=0.5	<=32	<=0.5	<=0.25	>64	>2
<=0.5	<=2	8	<=0.5	<=32	1	<=0.25	>64	>2
<=0.5	<=2	4	<=0.5	<=32	<=0.5	<=0.25	>64	>2
<=0.5	4	16	<=0.5	<=32	1	<=0.25	>64	>2
<=0.5	<=2	<=1	<=0.5	<=32	1	<=0.25	<=4	<=0.5
<=0.5	>16	>16	4	<=32	4	<=0.25	16	<=0.5
<=0.5	16	4	<=0.5	<=32	2	<=0.25	<=4	<=0.5
<=0.5	8	8	<=0.5	<=32	1	<=0.25	<=4	<=0.5
<=0.5	4	4	<=0.5	<=32	>8	<=0.25	>64	>2
<=0.5	8	8	<=0.5	<=32	>8	<=0.25	>64	<=0.5
<=0.5	4	8	<=0.5	<=32	4	0.5	64	>2
<=0.5	<=2	4	<=0.5	<=32	<=0.5	<=0.25	<=4	<=0.5
<=0.5	4	4	<=0.5	64	>8	<=0.25	>64	>2
<=0.5	4	4	<=0.5	<=32	1	<=0.25	>64	>2
<=0.5	<=2	2	<=0.5	<=32	<=0.5	<=0.25	>64	<=0.5
<=0.5	<=2	2	<=0.5	<=32	<=0.5	<=0.25	>64	>2
<=0.5	<=2	4	<=0.5	<=32	1	<=0.25	>64	<=0.5
<=0.5	>16	>16	8	<=32	>8	<=0.25	>64	>2
<=0.5	4	8	<=0.5	<=32	8	<=0.25	>64	>2
<=0.5	<=2	4	<=0.5	<=32	>8	<=0.25	>64	>2
<=0.5	4	4	<=0.5	<=32	1	<=0.25	>64	>2
<=0.5	>16	16	4	<=32	>8	<=0.25	>64	>2
<=0.5	8	8	<=0.5	<=32	>8	<=0.25	>64	<=0.5
<=0.5	8	4	<=0.5	<=32	1	<=0.25	>64	>2
<=0.5	<=2	4	<=0.5	<=32	<=0.5	<=0.25	<=4	<=0.5
<=0.5	<=2	2	<=0.5	<=32	1	<=0.25	>64	>2
<=0.5	<=2	4	<=0.5	<=32	>8	<=0.25	>64	>2
<=0.5	4	8	<=0.5	<=32	<=0.5	<=0.25	>64	>2
8	4	>16	>32	<=32	>8	<=0.25	>64	>2
<=0.5	4	4	<=0.5	<=32	1	<=0.25	>64	>2
<=0.5	4	4	<=0.5	<=32	>8	<=0.25	>64	>2
<=0.5	<=2	2	<=0.5	<=32	1	<=0.25	>64	>2
<=0.5	16	16	<=0.5	>64	>8	<=0.25	>64	>2
<=0.5	<=2	2	<=0.5	<=32	<=0.5	0.5	<=4	<=0.5
<=0.5	8	8	<=0.5	<=32	>8	<=0.25	>64	>2
<=0.5	4	8	<=0.5	<=32	<=0.5	<=0.25	8	<=0.5
<=0.5	<=2	8	<=0.5	<=32	<=0.5	<=0.25	<=4	<=0.5
<=0.5	<=2	2	<=0.5	<=32	<=0.5	<=0.25	>64	<=0.5
<=0.5	<=2	4	<=0.5	<=32	<=0.5	<=0.25	>64	>2

<=0.5	8	4	<=0.5	<=32	>8	<=0.25	>64	>2
<=0.5	4	4	<=0.5	<=32	<=0.5	<=0.25	>64	>2
<=0.5	<=2	4	<=0.5	<=32	1	<=0.25	>64	>2
<=0.5	4	4	<=0.5	<=32	4	<=0.25	>64	>2
<=0.5	8	4	<=0.5	<=32	<=0.5	<=0.25	<=4	<=0.5
1	>16	>16	32	<=32	<=0.5	1	>64	>2
<=0.5	<=2	4	<=0.5	<=32	<=0.5	<=0.25	>64	<=0.5
<=0.5	<=2	4	<=0.5	<=32	1	<=0.25	<=4	<=0.5
<=0.5	8	4	<=0.5	<=32	2	<=0.25	>64	<=0.5
<=0.5	<=2	4	<=0.5	<=32	<=0.5	<=0.25	<=4	1
<=0.5	<=2	4	<=0.5	<=32	<=0.5	<=0.25	16	<=0.5
<=0.5	8	4	<=0.5	<=32	<=0.5	<=0.25	>64	<=0.5
<=0.5	<=2	8	<=0.5	<=32	<=0.5	<=0.25	<=4	<=0.5
<=0.5	8	4	<=0.5	<=32	>8	<=0.25	>64	>2
<=0.5	4	4	<=0.5	<=32	<=0.5	<=0.25	>64	<=0.5
<=0.5	4	4	<=0.5	<=32	<=0.5	<=0.25	>64	<=0.5
<=0.5	<=2	4	<=0.5	<=32	>8	<=0.25	>64	>2
<=0.5	4	4	<=0.5	<=32	1	<=0.25	>64	>2
<=0.5	<=2	<=1	<=0.5	<=32	1	<=0.25	<=4	<=0.5
<=0.5	16	8	<=0.5	>64	1	<=0.25	<=4	<=0.5
<=0.5	16	8	<=0.5	<=32	>8	<=0.25	>64	>2
<=0.5	4	4	<=0.5	<=32	<=0.5	<=0.25	64	<=0.5
<=0.5	8	4	<=0.5	<=32	<=0.5	<=0.25	<=4	<=0.5
<=0.5	4	4	<=0.5	<=32	2	<=0.25	<=4	<=0.5
<=0.5	<=2	2	<=0.5	<=32	<=0.5	<=0.25	>64	<=0.5
<=0.5	<=2	4	<=0.5	<=32	<=0.5	<=0.25	>64	>2
<=0.5	<=2	<=1	<=0.5	<=32	<=0.5	<=0.25	<=4	<=0.5
<=0.5	4	2	<=0.5	<=32	<=0.5	1	>64	1
<=0.5	8	4	<=0.5	<=32	<=0.5	<=0.25	>64	>2
<=0.5	<=2	2	<=0.5	<=32	>8	<=0.25	>64	>2
<=0.5	<=2	4	<=0.5	<=32	<=0.5	<=0.25	<=4	<=0.5
<=0.5	<=2	4	<=0.5	<=32	<=0.5	<=0.25	<=4	<=0.5
<=0.5	16	8	<=0.5	<=32	1	<=0.25	64	<=0.5
<=0.5	8	8	<=0.5	<=32	>8	<=0.25	>64	>2
<=0.5	<=2	4	<=0.5	<=32	8	<=0.25	>64	>2
<=0.5	4	4	<=0.5	<=32	1	<=0.25	<=4	<=0.5
<=0.5	<=2	<=1	<=0.5	<=32	<=0.5	<=0.25	>64	<=0.5
<=0.5	4	4	<=0.5	<=32	<=0.5	<=0.25	<=4	<=0.5
<=0.5	<=2	2	<=0.5	<=32	<=0.5	<=0.25	>64	<=0.5
<=0.5	8	8	<=0.5	<=32	>8	<=0.25	>64	>2
1	8	>16	8	<=32	1	<=0.25	>64	<=0.5
<=0.5	4	8	<=0.5	<=32	>8	<=0.25	>64	>2
<=0.5	4	4	<=0.5	<=32	<=0.5	<=0.25	>64	>2
<=0.5	4	8	<=0.5	<=32	1	<=0.25	<=4	>2
<=0.5	8	4	<=0.5	<=32	<=0.5	<=0.25	>64	<=0.5
<=0.5	4	4	<=0.5	<=32	<=0.5	<=0.25	>64	>2
<=0.5	<=2	<=1	<=0.5	<=32	>8	<=0.25	>64	>2
<=0.5	4	4	<=0.5	<=32	1	<=0.25	>64	>2
<=0.5	4	4	<=0.5	<=32	<=0.5	<=0.25	>64	<=0.5
<=0.5	<=2	4	<=0.5	<=32	<=0.5	<=0.25	<=4	<=0.5
<=0.5	<=2	4	<=0.5	<=32	<=0.5	<=0.25	>64	<=0.5
<=0.5	<=2	4	<=0.5	<=32	<=0.5	<=0.25	32	>2
<=0.5	<=2	4	<=0.5	<=32	<=0.5	<=0.25	64	<=0.5
<=0.5	<=2	4	<=0.5	<=32	<=0.5	<=0.25	>64	<=0.5
<=0.5	<=2	2	<=0.5	<=32	<=0.5	<=0.25	>64	>2
2	4	>16	16	<=32	8	<=0.25	>64	>2
8	4	>16	>32	<=32	<=0.5	<=0.25	>64	>2
2	8	>16	16	<=32	8	<=0.25	>64	>2
<=0.5	4	2	<=0.5	<=32	1	<=0.25	<=4	<=0.5
<=0.5	8	2	<=0.5	<=32	<=0.5	<=0.25	<=4	<=0.5
<=0.5	<=2	2	<=0.5	<=32	>8	<=0.25	>64	>2

<=0.5	<=2	4	<=0.5	<=32	<=0.5	<=0.25	>64	<=0.5
<=0.5	4	4	<=0.5	64	<=0.5	<=0.25	>64	>2
<=0.5	<=2	4	<=0.5	<=32	>8	<=0.25	>64	>2
<=0.5	8	4	<=0.5	<=32	<=0.5	<=0.25	>64	<=0.5
<=0.5	<=2	2	<=0.5	<=32	>8	<=0.25	>64	<=0.5
<=0.5	<=2	8	<=0.5	>64	2	1	32	>2
1	>16	>16	32	64	>8	<=0.25	>64	>2
<=0.5	4	2	<=0.5	<=32	>8	<=0.25	>64	>2
<=0.5	4	4	<=0.5	<=32	>8	<=0.25	>64	>2
<=0.5	<=2	2	<=0.5	<=32	<=0.5	<=0.25	<=4	<=0.5
<=0.5	>16	16	4	<=32	8	0.5	>64	>2
<=0.5	>16	>16	16	<=32	>8	0.5	>64	>2
<=0.5	<=2	2	<=0.5	<=32	1	<=0.25	<=4	<=0.5
<=0.5	4	8	<=0.5	<=32	<=0.5	<=0.25	<=4	>2
<=0.5	4	4	<=0.5	<=32	<=0.5	<=0.25	<=4	<=0.5
<=0.5	<=2	4	<=0.5	<=32	1	<=0.25	<=4	<=0.5
<=0.5	4	4	<=0.5	<=32	8	<=0.25	>64	>2
<=0.5	4	8	<=0.5	<=32	<=0.5	<=0.25	>64	>2
<=0.5	<=2	2	<=0.5	<=32	1	0.5	<=4	<=0.5
<=0.5	4	4	<=0.5	<=32	<=0.5	<=0.25	>64	>2
<=0.5	<=2	4	<=0.5	<=32	<=0.5	<=0.25	>64	>2
<=0.5	4	4	<=0.5	<=32	>8	<=0.25	>64	>2
<=0.5	4	4	<=0.5	<=32	<=0.5	<=0.25	<=4	<=0.5
>16	8	>16	>32	<=32	>8	<=0.25	>64	>2
<=0.5	<=2	2	<=0.5	<=32	1	<=0.25	64	>2
<=0.5	8	8	<=0.5	>64	>8	<=0.25	>64	>2
<=0.5	<=2	4	<=0.5	<=32	<=0.5	<=0.25	>64	<=0.5
<=0.5	<=2	2	<=0.5	<=32	1	<=0.25	<=4	<=0.5
<=0.5	4	2	<=0.5	<=32	<=0.5	<=0.25	>64	<=0.5
<=0.5	4	4	<=0.5	<=32	<=0.5	<=0.25	<=4	<=0.5
<=0.5	<=2	<=1	<=0.5	<=32	>8	<=0.25	>64	>2
<=0.5	4	4	<=0.5	<=32	<=0.5	<=0.25	>64	<=0.5
<=0.5	8	8	<=0.5	<=32	1	<=0.25	>64	>2
2	4	4	<=0.5	<=32	>8	<=0.25	16	<=0.5
<=0.5	<=2	4	<=0.5	<=32	8	<=0.25	>64	>2
<=0.5	<=2	2	<=0.5	<=32	<=0.5	<=0.25	<=4	<=0.5
<=0.5	8	8	<=0.5	64	<=0.5	<=0.25	>64	>2
<=0.5	4	4	<=0.5	64	<=0.5	<=0.25	>64	>2
<=0.5	<=2	4	<=0.5	<=32	1	<=0.25	<=4	<=0.5
<=0.5	<=2	2	<=0.5	<=32	<=0.5	<=0.25	<=4	<=0.5
<=0.5	>16	>16	1	<=32	<=0.5	<=0.25	>64	>2
<=0.5	4	2	<=0.5	<=32	1	<=0.25	<=4	<=0.5
<=0.5	4	2	<=0.5	<=32	1	<=0.25	>64	<=0.5
<=0.5	<=2	4	<=0.5	<=32	<=0.5	<=0.25	>64	<=0.5
<=0.5	4	4	<=0.5	<=32	>8	<=0.25	>64	>2
<=0.5	4	4	<=0.5	<=32	<=0.5	<=0.25	<=4	<=0.5
<=0.5	4	8	<=0.5	<=32	<=0.5	<=0.25	>64	>2
<=0.5	4	4	<=0.5	<=32	<=0.5	<=0.25	<=4	<=0.5
<=0.5	<=2	2	<=0.5	<=32	<=0.5	<=0.25	64	>2
<=0.5	8	4	<=0.5	<=32	<=0.5	<=0.25	>64	>2
<=0.5	4	8	<=0.5	<=32	1	0.5	>64	>2
<=0.5	<=2	2	<=0.5	<=32	<=0.5	<=0.25	<=4	<=0.5
<=0.5	8	8	<=0.5	<=32	1	<=0.25	>64	>2
1	>16	16	4	<=32	>8	<=0.25	>64	>2
<=0.5	4	4	<=0.5	<=32	1	<=0.25	>64	>2
<=0.5	8	4	<=0.5	<=32	>8	<=0.25	>64	>2
<=0.5	4	4	<=0.5	<=32	1	<=0.25	<=4	<=0.5
<=0.5	4	4	<=0.5	<=32	>8	<=0.25	>64	>2
<=0.5	<=2	2	<=0.5	<=32	<=0.5	<=0.25	64	<=0.5
<=0.5	<=2	4	<=0.5	<=32	<=0.5	<=0.25	>64	<=0.5
<=0.5	<=2	4	<=0.5	<=32	>8	<=0.25	>64	>2

<=0.5		<=2	4	<=0.5	<=32	>8	<=0.25	>64	>2
<=0.5		<=2	4	<=0.5	<=32	<=0.5	<=0.25	64	>2
<=0.5		<=2	4	<=0.5	<=32	<=0.5	<=0.25	32	<=0.5
<=0.5		<=2	8	<=0.5	<=32	<=0.5	<=0.25	>64	>2
<=0.5		4	4	<=0.5	<=32	<=0.5	<=0.25	>64	>2
<=0.5		<=2	2	<=0.5	<=32	<=0.5	<=0.25	32	>2
<=0.5		4	8	<=0.5	64	1	<=0.25	>64	<=0.5
<=0.5		<=2	2	<=0.5	<=32	<=0.5	<=0.25	<=4	<=0.5
<=0.5		<=2	2	<=0.5	<=32	<=0.5	<=0.25	<=4	>2
<=0.5		<=2	2	<=0.5	<=32	<=0.5	<=0.25	<=4	<=0.5
<=0.5		4	4	<=0.5	<=32	8	<=0.25	>64	>2
<=0.5		<=2	4	<=0.5	<=32	<=0.5	<=0.25	<=4	>2
<=0.5		8	8	<=0.5	<=32	<=0.5	<=0.25	>64	>2
<=0.5		4	4	<=0.5	<=32	<=0.5	<=0.25	<=4	>2
<=0.5		<=2	2	<=0.5	<=32	1	<=0.25	64	>2
4		8	>16	>32	<=32	>8	<=0.25	>64	>2
<=0.5		4	4	<=0.5	<=32	1	<=0.25	>64	>2
<=0.5		8	4	<=0.5	<=32	1	<=0.25	>64	<=0.5
<=0.5		<=2	4	<=0.5	<=32	1	<=0.25	>64	>2
<=0.5		4	4	<=0.5	<=32	1	<=0.25	>64	>2
<=0.5		<=2	2	<=0.5	<=32	<=0.5	<=0.25	>64	>2
<=0.5		<=2	2	<=0.5	<=32	>8	<=0.25	64	>2
<=0.5		<=2	4	<=0.5	<=32	8	<=0.25	>64	>2
<=0.5		4	4	<=0.5	<=32	<=0.5	<=0.25	<=4	<=0.5
<=0.5		<=2	4	<=0.5	<=32	1	<=0.25	64	>2
<=0.5		8	8	<=0.5	<=32	2	<=0.25	<=4	>2
<=0.5		<=2	4	<=0.5	<=32	<=0.5	<=0.25	>64	<=0.5
<=0.5		4	4	<=0.5	<=32	1	<=0.25	<=4	>2
2		16	>16	>32	<=32	>8	<=0.25	>64	>2
<=0.5		<=2	4	<=0.5	<=32	<=0.5	<=0.25	>64	<=0.5
<=0.5		4	4	<=0.5	<=32	<=0.5	<=0.25	<=4	<=0.5
<=0.5		<=2	4	<=0.5	<=32	<=0.5	<=0.25	>64	>2
<=0.5		<=2	4	<=0.5	<=32	<=0.5	<=0.25	>64	<=0.5
<=0.5		4	4	<=0.5	<=32	2	<=0.25	64	<=0.5
<=0.5		<=2	4	<=0.5	<=32	<=0.5	<=0.25	>64	<=0.5
<=0.5		4	4	<=0.5	<=32	>8	<=0.25	>64	>2
<=0.5		8	8	<=0.5	64	1	<=0.25	8	>2
<=0.5		8	4	<=0.5	<=32	>8	<=0.25	>64	<=0.5
<=0.5		8	4	<=0.5	<=32	1	<=0.25	>64	>2
<=0.5		4	4	<=0.5	<=32	4	<=0.25	<=4	>2
<=0.5		4	4	<=0.5	<=32	1	<=0.25	>64	>2
<=0.5		4	4	<=0.5	<=32	1	<=0.25	>64	<=0.5
<=0.5		4	4	<=0.5	<=32	>8	<=0.25	>64	>2
<=0.5		8	8	<=0.5	<=32	<=0.5	<=0.25	>64	>2
<=0.5		<=2	4	<=0.5	<=32	<=0.5	<=0.25	<=4	<=0.5
<=0.5		8	8	<=0.5	<=32	>8	<=0.25	>64	>2
<=0.5		8	8	<=0.5	<=32	>8	<=0.25	>64	>2
<=0.5		<=2	8	<=0.5	<=32	1	<=0.25	<=4	<=0.5
<=0.5		8	8	<=0.5	<=32	>8	<=0.25	>64	>2
<=0.5		4	4	<=0.5	<=32	1	<=0.25	>64	<=0.5
<=0.5		8	16	<=0.5	<=32	>8	<=0.25	>64	>2
<=0.5		8	4	<=0.5	<=32	>8	<=0.25	>64	>2
<=0.5		4	2	<=0.5	<=32	<=0.5	0.5	<=4	<=0.5
<=0.5		4	4	<=0.5	<=32	1	<=0.25	<=4	<=0.5
<=0.5		4	2	<=0.5	<=32	<=0.5	<=0.25	<=4	<=0.5
<=0.5		4	4	<=0.5	<=32	<=0.5	<=0.25	<=4	<=0.5
<=0.5		8	4	<=0.5	<=32	2	<=0.25	<=4	<=0.5
<=0.5		<=2	4	<=0.5	<=32	1	<=0.25	<=4	<=0.5
<=0.5		<=2	2	<=0.5	<=32	<=0.5	<=0.25	<=4	<=0.5
<=0.5		<=2	2	<=0.5	<=32	>8	<=0.25	>64	>2
<=0.5		<=2	4	<=0.5	64	>8	<=0.25	>64	>2

<=0.5	4	4	<=0.5	>64	1	<=0.25	>64	>2
<=0.5	8	8	<=0.5	<=32	>8	<=0.25	>64	>2
<=0.5	8	8	<=0.5	<=32	<=0.5	<=0.25	>64	>2
<=0.5	4	4	<=0.5	<=32	1	<=0.25	<=4	<=0.5
<=0.5	<=2	4	<=0.5	<=32	1	<=0.25	>64	>2
<=0.5	<=2	4	<=0.5	<=32	<=0.5	<=0.25	>64	<=0.5
<=0.5	<=2	8	<=0.5	<=32	1	<=0.25	>64	<=0.5
<=0.5	<=2	8	<=0.5	<=32	<=0.5	<=0.25	>64	>2
<=0.5	16	8	<=0.5	<=32	<=0.5	<=0.25	<=4	<=0.5
<=0.5	4	8	<=0.5	<=32	<=0.5	<=0.25	>64	<=0.5
<=0.5	<=2	2	<=0.5	<=32	2	<=0.25	<=4	<=0.5
<=0.5	<=2	4	<=0.5	<=32	1	<=0.25	>64	<=0.5
<=0.5	4	4	<=0.5	<=32	1	<=0.25	<=4	<=0.5
<=0.5	4	4	<=0.5	64	2	<=0.25	>64	>2
<=0.5	8	8	<=0.5	<=32	1	<=0.25	<=4	<=0.5
<=0.5	4	4	<=0.5	<=32	>8	<=0.25	>64	>2
<=0.5	4	4	<=0.5	<=32	2	0.5	>64	<=0.5
<=0.5	>16	>16	1	<=32	4	<=0.25	>64	>2
<=0.5	4	8	<=0.5	<=32	1	<=0.25	<=4	>2
<=0.5	4	8	<=0.5	<=32	<=0.5	<=0.25	>64	>2
<=0.5	8	4	<=0.5	<=32	1	<=0.25	>64	>2
1	>16	>16	>32	<=32	>8	0.5	>64	>2
<=0.5	<=2	4	<=0.5	<=32	<=0.5	<=0.25	<=4	<=0.5
<=0.5	<=2	4	<=0.5	<=32	1	<=0.25	>64	<=0.5
<=0.5	>16	>16	2	<=32	<=0.5	0.5	64	>2
<=0.5	8	8	<=0.5	<=32	1	<=0.25	>64	<=0.5
<=0.5	8	8	<=0.5	<=32	1	<=0.25	>64	<=0.5
8	>16	>16	>32	64	1	1	>64	>2
<=0.5	4	4	<=0.5	<=32	<=0.5	<=0.25	>64	>2
<=0.5	4	4	<=0.5	<=32	>8	<=0.25	>64	>2
<=0.5	4	4	<=0.5	<=32	1	<=0.25	8	>2
<=0.5	<=2	<=1	<=0.5	<=32	<=0.5	<=0.25	>64	<=0.5
<=0.5	8	4	<=0.5	<=32	8	<=0.25	>64	<=0.5
<=0.5	>16	>16	32	<=32	>8	1	32	>2
<=0.5	16	16	2	<=32	1	<=0.25	32	<=0.5
<=0.5	4	4	<=0.5	<=32	2	<=0.25	>64	>2
<=0.5	4	8	<=0.5	<=32	8	<=0.25	>64	>2
<=0.5	4	8	<=0.5	<=32	<=0.5	<=0.25	>64	>2
<=0.5	8	8	<=0.5	<=32	1	0.5	<=4	<=0.5
<=0.5	4	4	<=0.5	<=32	<=0.5	<=0.25	<=4	<=0.5
<=0.5	<=2	8	<=0.5	<=32	8	0.5	>64	<=0.5
>16	>16	>16	>32	<=32	>8	1	>64	>2
<=0.5	8	16	<=0.5	<=32	2	<=0.25	>64	>2
<=0.5	16	16	1	>64	4	<=0.25	>64	>2
<=0.5	8	8	<=0.5	<=32	2	<=0.25	>64	>2
>16	>16	>16	>32	<=32	>8	1	>64	>2
<=0.5	4	8	<=0.5	<=32	>8	0.5	>64	>2
<=0.5	8	8	<=0.5	<=32	1	<=0.25	<=4	<=0.5
<=0.5	8	8	<=0.5	<=32	1	<=0.25	>64	>2
<=0.5	4	4	<=0.5	<=32	1	<=0.25	>64	<=0.5
<=0.5	4	8	<=0.5	<=32	<=0.5	<=0.25	8	>2
<=0.5	4	8	<=0.5	<=32	<=0.5	<=0.25	>64	>2
16	4	>16	>32	<=32	>8	<=0.25	>64	<=0.5
<=0.5	16	4	<=0.5	<=32	>8	<=0.25	>64	>2
<=0.5	8	4	<=0.5	<=32	<=0.5	<=0.25	>64	>2
<=0.5	4	4	<=0.5	<=32	1	<=0.25	<=4	<=0.5
<=0.5	4	4	<=0.5	<=32	1	<=0.25	<=4	<=0.5
<=0.5	4	4	<=0.5	<=32	8	<=0.25	>64	>2
<=0.5	<=2	2	<=0.5	<=32	1	<=0.25	>64	>2
<=0.5	4	4	<=0.5	<=32	>8	<=0.25	>64	>2
<=0.5	8	8	<=0.5	<=32	<=0.5	<=0.25	<=4	>2

<=0.5	>16	>16	16	64	>8	<=0.25	>64	>2
<=0.5	4	4	<=0.5	<=32	2	<=0.25	>64	>2
<=0.5	4	8	<=0.5	<=32	1	<=0.25	32	<=0.5
<=0.5	4	4	<=0.5	<=32	<=0.5	<=0.25	>64	<=0.5
<=0.5	>16	>16	16	<=32	>8	0.5	>64	>2
<=0.5	8	8	<=0.5	>64	>8	1	>64	>2
<=0.5	4	4	<=0.5	<=32	<=0.5	<=0.25	<=4	<=0.5
<=0.5	8	8	<=0.5	<=32	>8	<=0.25	>64	>2
<=0.5	4	4	<=0.5	<=32	<=0.5	<=0.25	<=4	<=0.5
<=0.5	8	>16	<=0.5	<=32	2	<=0.25	64	>2
<=0.5	<=2	4	<=0.5	<=32	1	<=0.25	<=4	<=0.5
<=0.5	<=2	4	<=0.5	<=32	<=0.5	<=0.25	<=4	<=0.5
<=0.5	4	4	<=0.5	<=32	1	<=0.25	<=4	<=0.5
<=0.5	<=2	4	<=0.5	<=32	1	<=0.25	>64	>2
<=0.5	4	4	<=0.5	<=32	8	<=0.25	>64	>2
<=0.5	<=2	4	<=0.5	<=32	>8	<=0.25	>64	>2
<=0.5	<=2	4	<=0.5	<=32	<=0.5	<=0.25	<=4	<=0.5
<=0.5	4	8	<=0.5	<=32	1	0.5	>64	<=0.5
<=0.5	8	4	<=0.5	<=32	8	<=0.25	<=4	<=0.5
>16	8	>16	>32	64	>8	<=0.25	>64	>2
<=0.5	4	4	<=0.5	<=32	1	<=0.25	>64	<=0.5
<=0.5	<=2	2	<=0.5	<=32	1	<=0.25	>64	<=0.5
16	8	>16	>32	<=32	>8	<=0.25	>64	>2
<=0.5	<=2	2	<=0.5	<=32	<=0.5	<=0.25	<=4	<=0.5
<=0.5	4	4	<=0.5	<=32	<=0.5	<=0.25	<=4	<=0.5
<=0.5	<=2	4	<=0.5	<=32	1	<=0.25	>64	>2
<=0.5	4	4	<=0.5	<=32	2	<=0.25	>64	<=0.5
<=0.5	4	4	<=0.5	<=32	<=0.5	<=0.25	<=4	>2
<=0.5	8	8	<=0.5	<=32	1	<=0.25	<=4	<=0.5
<=0.5	8	16	<=0.5	<=32	1	<=0.25	>64	<=0.5
<=0.5	4	4	<=0.5	<=32	>8	<=0.25	>64	>2
<=0.5	4	8	<=0.5	<=32	<=0.5	<=0.25	>64	<=0.5
<=0.5	4	4	<=0.5	<=32	1	<=0.25	<=4	<=0.5
<=0.5	>16	>16	<=0.5	<=32	<=0.5	<=0.25	>64	>2
<=0.5	16	16	4	<=32	2	0.5	32	<=0.5
<=0.5	<=2	2	<=0.5	<=32	1	<=0.25	<=4	<=0.5
<=0.5	4	4	<=0.5	<=32	<=0.5	<=0.25	>64	>2
<=0.5	<=2	4	<=0.5	<=32	8	<=0.25	>64	>2
<=0.5	<=2	4	<=0.5	<=32	1	<=0.25	>64	>2
<=0.5	4	8	<=0.5	<=32	<=0.5	<=0.25	<=4	<=0.5
<=0.5	4	4	<=0.5	<=32	8	<=0.25	>64	>2
<=0.5	4	4	<=0.5	<=32	<=0.5	<=0.25	>64	>2
<=0.5	4	4	<=0.5	>64	<=0.5	<=0.25	>64	>2
<=0.5	>16	16	<=0.5	<=32	2	<=0.25	<=4	>2
<=0.5	4	4	<=0.5	<=32	1	<=0.25	>64	<=0.5
<=0.5	4	4	<=0.5	<=32	>8	<=0.25	>64	>2
<=0.5	8	8	<=0.5	64	>8	<=0.25	>64	>2
<=0.5	<=2	<=1	<=0.5	<=32	<=0.5	<=0.25	<=4	<=0.5
<=0.5	4	4	<=0.5	64	1	<=0.25	>64	>2
<=0.5	8	8	<=0.5	<=32	>8	<=0.25	>64	>2
<=0.5	<=2	4	<=0.5	<=32	1	<=0.25	<=4	<=0.5
<=0.5	<=2	4	<=0.5	<=32	1	<=0.25	>64	>2
<=0.5	4	4	<=0.5	<=32	>8	<=0.25	>64	>2
<=0.5	<=2	8	<=0.5	<=32	1	<=0.25	>64	>2
<=0.5	<=2	4	<=0.5	<=32	<=0.5	<=0.25	>64	>2
<=0.5	8	8	<=0.5	<=32	1	<=0.25	<=4	<=0.5
<=0.5	<=2	2	<=0.5	<=32	<=0.5	<=0.25	<=4	<=0.5
<=0.5	4	4	<=0.5	<=32	1	<=0.25	<=4	<=0.5
<=0.5	<=2	2	<=0.5	<=32	<=0.5	<=0.25	<=4	<=0.5
<=0.5	4	4	<=0.5	<=32	1	<=0.25	>64	<=0.5
<=0.5	<=2	4	<=0.5	<=32	<=0.5	<=0.25	>64	>2

	<=0.5	4	8	<=0.5	<=32	>8	<=0.25	>64	>2
	<=0.5	4	4	<=0.5	<=32	>8	<=0.25	>64	>2
	<=0.5	<=2	4	<=0.5	<=32	<=0.5	<=0.25	>64	>2
	<=0.5	<=2	4	<=0.5	<=32	<=0.5	<=0.25	>64	>2
	<=0.5	4	8	<=0.5	<=32	<=0.5	<=0.25	>64	>2
	<=0.5	>16	16	<=0.5	<=32	>8	<=0.25	>64	>2
	<=0.5	4	8	<=0.5	<=32	2	<=0.25	>64	<=0.5
	<=0.5	8	8	<=0.5	<=32	>8	<=0.25	>64	>2
	<=0.5	4	4	<=0.5	<=32	1	<=0.25	<=4	<=0.5
	<=0.5	4	4	<=0.5	<=32	<=0.5	<=0.25	>64	<=0.5
	<=0.5	8	4	<=0.5	<=32	>8	<=0.25	>64	>2
	<=0.5	4	8	<=0.5	<=32	>8	<=0.25	>64	>2
	<=0.5	16	8	<=0.5	64	>8	<=0.25	>64	>2
	<=0.5	4	4	<=0.5	<=32	<=0.5	<=0.25	>64	>2
	<=0.5	8	8	<=0.5	<=32	>8	<=0.25	>64	>2
	<=0.5	>16	>16	16	64	8	<=0.25	32	<=0.5
	<=0.5	<=2	2	<=0.5	<=32	<=0.5	<=0.25	>64	<=0.5
	<=0.5	4	4	<=0.5	<=32	1	<=0.25	<=4	<=0.5
	<=0.5	4	4	<=0.5	<=32	1	<=0.25	>64	>2
	<=0.5	<=2	2	<=0.5	64	<=0.5	<=0.25	>64	>2
	<=0.5	4	2	<=0.5	<=32	2	<=0.25	<=4	<=0.5
	<=0.5	<=2	4	<=0.5	<=32	<=0.5	<=0.25	<=4	<=0.5
	8	16	>16	>32	<=32	<=0.5	<=0.25	>64	>2
	<=0.5	4	4	<=0.5	<=32	1	<=0.25	>64	<=0.5
	<=0.5	4	4	<=0.5	<=32	1	<=0.25	>64	<=0.5
	<=0.5	4	4	<=0.5	<=32	<=0.5	<=0.25	>64	<=0.5
	<=0.5	4	2	<=0.5	<=32	>8	<=0.25	>64	>2
	1	>16	>16	32	<=32	>8	0.5	>64	>2
	<=0.5	<=2	2	<=0.5	<=32	1	<=0.25	>64	<=0.5
	<=0.5	<=2	2	<=0.5	<=32	8	<=0.25	>64	>2
	<=0.5	8	16	<=0.5	>64	<=0.5	<=0.25	>64	>2
	<=0.5	4	8	<=0.5	<=32	<=0.5	<=0.25	>64	>2
	<=0.5	<=2	4	<=0.5	<=32	>8	<=0.25	<=4	>2
	<=0.5	4	4	<=0.5	<=32	1	<=0.25	>64	>2
	<=0.5	4	4	<=0.5	<=32	<=0.5	<=0.25	<=4	<=0.5
	<=0.5	4	4	<=0.5	<=32	<=0.5	<=0.25	>64	<=0.5
	<=0.5	<=2	4	<=0.5	<=32	<=0.5	<=0.25	<=4	<=0.5
	<=0.5	4	4	<=0.5	<=32	<=0.5	<=0.25	<=4	<=0.5
	<=0.5	>16	8	<=0.5	>64	4	<=0.25	32	<=0.5
	<=0.5	4	4	<=0.5	<=32	<=0.5	<=0.25	<=4	<=0.5
	<=0.5	4	8	<=0.5	<=32	>8	<=0.25	>64	>2
	<=0.5	8	8	<=0.5	<=32	>8	<=0.25	>64	>2
	<=0.5	4	4	<=0.5	<=32	<=0.5	<=0.25	>64	<=0.5
	<=0.5	4	8	<=0.5	<=32	<=0.5	<=0.25	>64	>2
	<=0.5	8	8	<=0.5	<=32	1	<=0.25	<=4	<=0.5
	<=0.5	4	4	<=0.5	<=32	2	<=0.25	>64	<=0.5
	<=0.5	8	4	<=0.5	>64	<=0.5	<=0.25	>64	>2
	<=0.5	>16	>16	8	64	1	<=0.25	>64	<=0.5
	<=0.5	4	16	<=0.5	<=32	1	<=0.25	64	>2
	<=0.5	8	4	<=0.5	<=32	1	<=0.25	>64	>2
	<=0.5	4	8	<=0.5	<=32	1	<=0.25	<=4	>2
	<=0.5	<=2	4	<=0.5	<=32	1	<=0.25	<=4	<=0.5
	<=0.5	4	2	<=0.5	<=32	1	<=0.25	>64	>2
	<=0.5	8	8	<=0.5	>64	2	<=0.25	<=4	>2
	<=0.5	>16	16	<=0.5	64	2	<=0.25	<=4	<=0.5
<=0.25	<=0.5	4	4	<=0.5	<=32	1	<=0.25	>64	<=0.5
	<=0.5	8	8	<=0.5	<=32	1	<=0.25	<=4	>2
	<=0.5	8	4	<=0.5	64	>8	<=0.25	64	>2
	<=0.5	8	8	<=0.5	<=32	>8	<=0.25	>64	>2
	<=0.5	<=2	4	<=0.5	<=32	8	<=0.25	>64	<=0.5
	<=0.5	8	4	<=0.5	<=32	>8	<=0.25	>64	>2

<=0.5	8	4	<=0.5	<=32	<=0.5	<=0.25	>64	<=0.5
<=0.5	4	2	<=0.5	<=32	>8	<=0.25	>64	>2
<=0.5	4	4	<=0.5	64	<=0.5	<=0.25	>64	>2
<=0.5	<=2	4	<=0.5	<=32	<=0.5	<=0.25	>64	<=0.5
<=0.5	4	4	<=0.5	<=32	1	<=0.25	<=4	<=0.5
<=0.5	4	4	<=0.5	<=32	2	0.5	>64	<=0.5
<=0.5	<=2	<=1	<=0.5	<=32	1	<=0.25	>64	>2
<=0.5	4	4	<=0.5	<=32	1	<=0.25	<=4	<=0.5
<=0.5	<=2	4	<=0.5	<=32	<=0.5	<=0.25	>64	<=0.5
<=0.5	<=2	2	<=0.5	<=32	<=0.5	<=0.25	<=4	<=0.5
<=0.5	<=2	4	<=0.5	<=32	<=0.5	<=0.25	>64	<=0.5
<=0.5	4	4	<=0.5	<=32	<=0.5	<=0.25	>64	<=0.5
<=0.5	<=2	2	<=0.5	<=32	<=0.5	<=0.25	>64	<=0.5
<=0.5	8	8	<=0.5	<=32	<=0.5	<=0.25	<=4	>2
<=0.5	<=2	2	<=0.5	<=32	<=0.5	<=0.25	>64	<=0.5
<=0.5	4	4	<=0.5	<=32	<=0.5	<=0.25	>64	<=0.5
<=0.5	4	4	<=0.5	<=32	1	<=0.25	<=4	<=0.5
<=0.5	4	4	<=0.5	<=32	<=0.5	<=0.25	>64	>2
<=0.5	<=2	4	<=0.5	<=32	<=0.5	<=0.25	<=4	<=0.5
<=0.5	4	4	<=0.5	<=32	<=0.5	<=0.25	>64	<=0.5
4	>16	>16	16	<=32	8	<=0.25	>64	>2
<=0.5	<=2	4	<=0.5	<=32	<=0.5	<=0.25	>64	>2
<=0.5	8	4	<=0.5	<=32	<=0.5	<=0.25	>64	>2
<=0.5	4	8	<=0.5	<=32	<=0.5	<=0.25	<=4	<=0.5
<=0.5	<=2	2	<=0.5	<=32	>8	<=0.25	<=4	>2
<=0.5	4	4	<=0.5	<=32	>8	<=0.25	>64	<=0.5
<=0.5	<=2	4	<=0.5	<=32	<=0.5	<=0.25	<=4	<=0.5
<=0.5	>16	>16	4	<=32	>8	<=0.25	>64	>2
<=0.5	4	2	<=0.5	<=32	<=0.5	<=0.25	<=4	<=0.5
<=0.5	<=2	4	<=0.5	<=32	<=0.5	<=0.25	>64	<=0.5
<=0.5	8	4	<=0.5	<=32	1	<=0.25	>64	>2
<=0.5	4	4	<=0.5	<=32	1	<=0.25	<=4	<=0.5
<=0.5	16	8	<=0.5	<=32	>8	<=0.25	>64	>2
<=0.5	8	4	<=0.5	<=32	8	<=0.25	64	>2
<=0.5	16	8	<=0.5	<=32	2	0.5	32	<=0.5
<=0.5	<=2	4	<=0.5	<=32	1	<=0.25	>64	>2
<=0.5	<=2	<=1	<=0.5	<=32	<=0.5	<=0.25	<=4	<=0.5
<=0.5	<=2	<=1	<=0.5	<=32	1	0.5	<=4	<=0.5
<=0.5	4	4	<=0.5	<=32	>8	<=0.25	>64	>2
<=0.5	16	16	<=0.5	<=32	>8	<=0.25	>64	>2
<=0.5	4	8	<=0.5	<=32	1	<=0.25	>64	<=0.5
<=0.5	>16	>16	8	<=32	>8	<=0.25	>64	>2
<=0.5	4	4	<=0.5	<=32	<=0.5	<=0.25	>64	>2
<=0.5	>16	>16	8	<=32	1	<=0.25	>64	>2
<=0.5	4	8	<=0.5	64	1	<=0.25	<=4	<=0.5
<=0.5	4	4	<=0.5	<=32	>8	<=0.25	64	<=0.5
<=0.5	4	4	<=0.5	<=32	<=0.5	<=0.25	<=4	<=0.5
<=0.5	<=2	4	<=0.5	<=32	<=0.5	<=0.25	<=4	<=0.5
<=0.5	4	4	<=0.5	<=32	<=0.5	<=0.25	<=4	<=0.5
<=0.5	<=2	4	<=0.5	<=32	>8	<=0.25	>64	>2
<=0.5	4	4	<=0.5	<=32	1	<=0.25	>64	>2
<=0.5	4	8	<=0.5	<=32	>8	<=0.25	>64	>2
<=0.5	<=2	<=1	<=0.5	<=32	1	<=0.25	<=4	<=0.5
<=0.5	<=2	8	<=0.5	<=32	2	<=0.25	>64	>2
<=0.5	4	4	<=0.5	<=32	<=0.5	<=0.25	<=4	>2
<=0.5	4	4	<=0.5	<=32	1	<=0.25	<=4	>2
<=0.5	4	4	<=0.5	<=32	<=0.5	<=0.25	<=4	>2
<=0.5	8	4	<=0.5	<=32	>8	<=0.25	>64	>2
<=0.5	4	4	<=0.5	<=32	>8	<=0.25	>64	<=0.5
<=0.5	4	4	<=0.5	<=32	<=0.5	<=0.25	<=4	<=0.5
4	8	>16	>32	<=32	>8	<=0.25	>64	>2

<=0.5	4	4	<=0.5	<=32	1	<=0.25	<=4	<=0.5
4	>16	>16	>32	<=32	>8	<=0.25	>64	>2
<=0.5	>16	>16	16	<=32	>8	0.5	>64	>2
<=0.5	4	4	<=0.5	<=32	<=0.5	<=0.25	>64	<=0.5
<=0.5	8	16	<=0.5	<=32	<=0.5	<=0.25	<=4	>2
<=0.5	8	8	<=0.5	<=32	>8	<=0.25	>64	>2
<=0.5	8	8	<=0.5	<=32	2	<=0.25	>64	<=0.5
<=0.5	>16	>16	8	<=32	>8	0.5	>64	>2
>16	8	>16	>32	<=32	>8	<=0.25	>64	>2
<=0.5	4	4	<=0.5	<=32	>8	<=0.25	>64	>2
>16	8	>16	>32	<=32	>8	<=0.25	>64	>2
<=0.5	<=2	4	<=0.5	<=32	1	<=0.25	<=4	<=0.5
<=0.5	<=2	<=1	<=0.5	<=32	<=0.5	<=0.25	64	<=0.5
<=0.5	4	4	<=0.5	<=32	<=0.5	<=0.25	>64	>2
<=0.5	<=2	8	<=0.5	<=32	2	<=0.25	8	<=0.5
<=0.5	4	4	<=0.5	<=32	<=0.5	<=0.25	>64	<=0.5
<=0.5	<=2	2	<=0.5	<=32	8	<=0.25	>64	<=0.5
<=0.5	<=2	4	<=0.5	<=32	<=0.5	<=0.25	>64	<=0.5
<=0.5	4	4	<=0.5	<=32	>8	<=0.25	>64	>2
2	>16	>16	16	<=32	8	<=0.25	>64	<=0.5
<=0.5	<=2	2	<=0.5	<=32	<=0.5	<=0.25	<=4	<=0.5
<=0.5	4	4	<=0.5	<=32	8	0.5	>64	>2
<=0.5	4	8	<=0.5	<=32	>8	<=0.25	>64	>2
<=0.5	4	4	<=0.5	<=32	4	<=0.25	>64	>2
<=0.5	<=2	4	<=0.5	<=32	1	<=0.25	<=4	<=0.5
<=0.5	<=2	4	<=0.5	<=32	<=0.5	<=0.25	>64	>2
<=0.5	<=2	2	<=0.5	<=32	1	<=0.25	>64	>2
<=0.5	<=2	4	<=0.5	<=32	<=0.5	<=0.25	<=4	<=0.5
<=0.5	4	4	<=0.5	<=32	<=0.5	<=0.25	64	>2
<=0.5	8	8	<=0.5	<=32	>8	<=0.25	>64	<=0.5
<=0.5	4	4	<=0.5	<=32	<=0.5	<=0.25	>64	>2
<=0.5	4	4	<=0.5	<=32	1	<=0.25	<=4	<=0.5
<=0.5	<=2	2	<=0.5	<=32	>8	<=0.25	>64	>2
<=0.5	4	8	<=0.5	<=32	<=0.5	<=0.25	<=4	<=0.5
<=0.5	4	4	<=0.5	64	<=0.5	<=0.25	>64	>2
4	>16	>16	32	<=32	>8	0.5	>64	>2
<=0.5	8	8	<=0.5	<=32	>8	<=0.25	>64	>2
<=0.5	4	4	<=0.5	<=32	1	<=0.25	<=4	<=0.5
<=0.5	8	4	<=0.5	<=32	>8	<=0.25	>64	>2
<=0.5	<=2	4	<=0.5	<=32	1	<=0.25	<=4	<=0.5
<=0.5	4	4	<=0.5	<=32	8	<=0.25	64	>2
<=0.5	8	8	<=0.5	<=32	<=0.5	<=0.25	8	>2
<=0.5	4	4	<=0.5	<=32	<=0.5	<=0.25	<=4	<=0.5
<=0.5	<=2	2	<=0.5	<=32	1	<=0.25	<=4	<=0.5
<=0.5	<=2	4	<=0.5	<=32	<=0.5	<=0.25	<=4	<=0.5
<=0.5	8	4	<=0.5	<=32	<=0.5	<=0.25	64	>2
<=0.5	<=2	2	<=0.5	<=32	1	<=0.25	>64	<=0.5
<=0.5	4	8	<=0.5	<=32	<=0.5	<=0.25	<=4	<=0.5
<=0.5	<=2	2	<=0.5	<=32	1	<=0.25	>64	>2
<=0.5	4	2	<=0.5	<=32	1	<=0.25	>64	>2
<=0.5	<=2	4	<=0.5	<=32	<=0.5	<=0.25	>64	<=0.5
<=0.5	8	8	<=0.5	<=32	<=0.5	<=0.25	<=4	<=0.5
<=0.5	<=2	2	<=0.5	<=32	<=0.5	<=0.25	>64	>2
<=0.5	4	4	<=0.5	<=32	1	<=0.25	<=4	<=0.5
<=0.5	<=2	4	<=0.5	<=32	>8	<=0.25	>64	>2
<=0.5	<=2	2	<=0.5	<=32	1	<=0.25	>64	>2
<=0.5	4	8	<=0.5	<=32	1	<=0.25	<=4	<=0.5
<=0.5	4	4	<=0.5	64	1	<=0.25	>64	<=0.5
<=0.5	4	8	<=0.5	<=32	<=0.5	<=0.25	>64	<=0.5
<=0.5	16	16	<=0.5	<=32	>8	<=0.25	>64	>2
<=0.5	4	8	<=0.5	<=32	1	<=0.25	<=4	<=0.5

<=0.5	4	4	<=0.5	<=32	1	<=0.25	>64	>2
<=0.5	4	4	<=0.5	<=32	<=0.5	<=0.25	<=4	<=0.5
<=0.5	<=2	<=1	<=0.5	<=32	4	0.5	<=4	>2
<=0.5	8	8	<=0.5	<=32	<=0.5	<=0.25	>64	>2
<=0.5	8	8	<=0.5	<=32	<=0.5	<=0.25	>64	>2
<=0.5	<=2	4	<=0.5	<=32	1	<=0.25	>64	<=0.5
<=0.5	4	4	<=0.5	64	1	<=0.25	>64	>2
<=0.5	4	4	<=0.5	<=32	1	<=0.25	>64	<=0.5
<=0.5	<=2	4	<=0.5	<=32	2	<=0.25	>64	>2
<=0.5	8	4	<=0.5	<=32	1	<=0.25	>64	>2
<=0.5	8	8	<=0.5	<=32	<=0.5	<=0.25	<=4	>2
<=0.5	4	4	<=0.5	<=32	<=0.5	<=0.25	>64	>2
<=0.5	4	4	<=0.5	<=32	1	<=0.25	>64	>2
<=0.5	<=2	4	<=0.5	<=32	<=0.5	<=0.25	>64	<=0.5
<=0.5	4	4	<=0.5	<=32	2	<=0.25	<=4	<=0.5
<=0.5	<=2	2	<=0.5	<=32	1	<=0.25	>64	>2
<=0.5	4	4	<=0.5	<=32	<=0.5	<=0.25	<=4	<=0.5
<=0.5	8	4	<=0.5	<=32	1	<=0.25	>64	>2
<=0.5	8	4	<=0.5	<=32	<=0.5	<=0.25	>64	>2
<=0.5	4	4	<=0.5	<=32	<=0.5	<=0.25	>64	>2
<=0.5	4	16	<=0.5	<=32	>8	<=0.25	>64	>2
>16	16	>16	>32	<=32	>8	<=0.25	>64	>2
<=0.5	<=2	4	<=0.5	<=32	<=0.5	<=0.25	>64	>2
<=0.5	4	4	<=0.5	<=32	2	<=0.25	>64	>2
<=0.5	4	4	<=0.5	<=32	1	<=0.25	>64	>2
1	>16	>16	8	<=32	1	<=0.25	>64	>2
<=0.5	<=2	<=1	<=0.5	<=32	<=0.5	<=0.25	<=4	<=0.5
<=0.5	<=2	4	<=0.5	<=32	<=0.5	<=0.25	>64	>2
<=0.5	4	8	<=0.5	<=32	<=0.5	<=0.25	>64	>2
<=0.5	<=2	2	<=0.5	<=32	1	<=0.25	<=4	<=0.5
<=0.5	8	8	<=0.5	<=32	2	<=0.25	>64	<=0.5
>16	16	>16	>32	<=32	>8	<=0.25	>64	>2
<=0.5	4	4	<=0.5	<=32	<=0.5	<=0.25	<=4	<=0.5
<=0.5	4	4	<=0.5	<=32	<=0.5	<=0.25	>64	>2
<=0.5	16	16	<=0.5	<=32	>8	<=0.25	>64	>2
<=0.5	4	4	<=0.5	<=32	<=0.5	<=0.25	>64	>2
<=0.5	<=2	4	<=0.5	<=32	1	<=0.25	64	>2
<=0.5	8	8	<=0.5	<=32	8	<=0.25	>64	>2
1	4	8	<=0.5	<=32	1	0.5	<=4	<=0.5
<=0.5	<=2	2	<=0.5	>64	<=0.5	<=0.25	>64	>2
<=0.5	4	16	<=0.5	<=32	>8	<=0.25	>64	<=0.5
<=0.5	4	4	<=0.5	<=32	1	<=0.25	<=4	<=0.5
<=0.5	4	4	<=0.5	<=32	8	<=0.25	<=4	<=0.5
<=0.5	8	4	<=0.5	<=32	1	<=0.25	<=4	1
<=0.5	8	4	<=0.5	<=32	<=0.5	<=0.25	>64	>2
<=0.5	8	8	<=0.5	<=32	<=0.5	<=0.25	<=4	>2
<=0.5	<=2	4	<=0.5	<=32	<=0.5	<=0.25	>64	>2
<=0.5	4	4	<=0.5	64	<=0.5	<=0.25	>64	>2
<=0.5	<=2	2	<=0.5	<=32	1	<=0.25	>64	>2
<=0.5	8	8	<=0.5	<=32	1	<=0.25	>64	>2
<=0.5	4	4	<=0.5	<=32	1	<=0.25	>64	>2
<=0.5	4	8	<=0.5	<=32	<=0.5	<=0.25	<=4	<=0.5
<=0.5	8	8	<=0.5	<=32	1	<=0.25	>64	>2
<=0.5	4	4	<=0.5	<=32	<=0.5	<=0.25	>64	>2
<=0.5	4	4	<=0.5	<=32	<=0.5	<=0.25	>64	>2
<=0.5	<=2	2	<=0.5	<=32	1	<=0.25	<=4	<=0.5
<=0.5	>16	>16	8	<=32	>8	<=0.25	>64	>2
<=0.5	8	8	<=0.5	<=32	1	<=0.25	>64	>2
<=0.5	<=2	2	<=0.5	<=32	<=0.5	<=0.25	<=4	<=0.5
<=0.5	8	8	<=0.5	<=32	1	<=0.25	>64	<=0.5
<=0.5	<=2	2	<=0.5	<=32	1	<=0.25	<=4	<=0.5

<=0.5	4	4	<=0.5	<=32	1	<=0.25	>64	<=0.5
<=0.5	4	2	<=0.5	64	1	<=0.25	<=4	<=0.5
<=0.5	<=2	4	<=0.5	<=32	<=0.5	<=0.25	>64	<=0.5
<=0.5	<=2	4	<=0.5	<=32	1	<=0.25	>64	<=0.5
<=0.5	<=2	2	<=0.5	<=32	>8	<=0.25	>64	>2
<=0.5	8	4	<=0.5	<=32	<=0.5	<=0.25	>64	>2
<=0.5	8	2	<=0.5	<=32	<=0.5	<=0.25	>64	<=0.5
<=0.5	<=2	4	<=0.5	<=32	<=0.5	<=0.25	>64	>2
<=0.5	>16	>16	16	<=32	>8	<=0.25	32	<=0.5
<=0.5	4	8	<=0.5	<=32	>8	<=0.25	>64	>2
<=0.5	<=2	4	<=0.5	<=32	<=0.5	<=0.25	<=4	<=0.5
<=0.5	4	4	<=0.5	<=32	1	<=0.25	<=4	<=0.5
<=0.5	8	8	<=0.5	<=32	8	<=0.25	>64	>2
<=0.5	8	4	<=0.5	<=32	>8	<=0.25	>64	>2
<=0.5	>16	>16	8	<=32	>8	<=0.25	>64	>2
1	>16	>16	<=0.5	64	4	<=0.25	64	>2
<=0.5	8	4	<=0.5	<=32	>8	<=0.25	>64	>2
<=0.5	<=2	4	<=0.5	<=32	1	<=0.25	>64	<=0.5
>16	>16	>16	>32	<=32	>8	<=0.25	>64	>2
<=0.5	<=2	4	<=0.5	<=32	1	<=0.25	>64	>2
<=0.5	8	8	<=0.5	<=32	2	<=0.25	>64	<=0.5
<=0.5	4	4	<=0.5	<=32	1	<=0.25	64	<=0.5
<=0.5	<=2	8	<=0.5	<=32	1	0.5	>64	<=0.5
<=0.5	<=2	2	<=0.5	<=32	1	<=0.25	>64	>2
<=0.5	4	8	<=0.5	<=32	1	<=0.25	>64	<=0.5
<=0.5	4	4	<=0.5	<=32	8	<=0.25	>64	>2
<=0.5	<=2	8	<=0.5	<=32	<=0.5	<=0.25	<=4	<=0.5
<=0.5	>16	>16	16	<=32	>8	0.5	>64	>2
<=0.5	<=2	2	<=0.5	<=32	2	<=0.25	<=4	<=0.5
<=0.5	4	4	<=0.5	<=32	2	<=0.25	>64	<=0.5
>16	>16	>16	>32	<=32	1	0.5	>64	>2
<=0.5	>16	>16	8	<=32	>8	0.5	>64	>2
<=0.5	<=2	4	<=0.5	<=32	1	<=0.25	>64	>2
<=0.5	4	8	<=0.5	<=32	<=0.5	<=0.25	<=4	<=0.5
<=0.5	8	8	<=0.5	<=32	>8	0.5	>64	>2
<=0.5	4	4	<=0.5	<=32	1	<=0.25	>64	>2
<=0.5	4	4	<=0.5	<=32	<=0.5	<=0.25	>64	<=0.5
<=0.5	<=2	4	<=0.5	<=32	>8	<=0.25	>64	<=0.5
<=0.5	4	4	<=0.5	<=32	>8	<=0.25	>64	>2
<=0.5	4	4	<=0.5	<=32	1	<=0.25	>64	<=0.5
<=0.5	<=2	16	<=0.5	<=32	<=0.5	<=0.25	>64	<=0.5
<=0.5	<=2	4	<=0.5	<=32	>8	<=0.25	>64	<=0.5
<=0.5	<=2	2	<=0.5	<=32	1	<=0.25	>64	<=0.5
<=0.5	8	2	<=0.5	<=32	1	<=0.25	<=4	<=0.5
<=0.5	8	4	<=0.5	<=32	2	<=0.25	>64	<=0.5
<=0.5	4	4	<=0.5	<=32	>8	<=0.25	>64	>2
<=0.5	4	4	<=0.5	<=32	<=0.5	<=0.25	>64	<=0.5
<=0.5	4	4	<=0.5	<=32	<=0.5	<=0.25	>64	>2
<=0.5	8	4	<=0.5	<=32	<=0.5	<=0.25	<=4	<=0.5
<=0.5	4	4	<=0.5	<=32	4	<=0.25	<=4	>2
<=0.5	<=2	2	<=0.5	64	>8	<=0.25	>64	>2
<=0.5	4	4	<=0.5	<=32	<=0.5	<=0.25	<=4	<=0.5
<=0.5	4	4	<=0.5	<=32	2	<=0.25	<=4	>2
<=0.5	4	4	<=0.5	<=32	8	<=0.25	>64	<=0.5
<=0.5	4	2	<=0.5	<=32	<=0.5	<=0.25	>64	<=0.5
<=0.5	4	4	<=0.5	<=32	>8	<=0.25	>64	>2
<=0.5	<=2	2	<=0.5	<=32	<=0.5	0.5	<=4	<=0.5
<=0.5	4	8	<=0.5	<=32	4	<=0.25	<=4	<=0.5
<=0.5	4	8	<=0.5	<=32	1	<=0.25	>64	<=0.5
<=0.5	4	8	<=0.5	<=32	>8	<=0.25	>64	>2
<=0.5	8	8	<=0.5	<=32	<=0.5	<=0.25	<=4	<=0.5

<=0.5	8	4	<=0.5	<=32	2	<=0.25	>64	>2
<=0.5	>16	>16	32	<=32	>8	<=0.25	>64	<=0.5
<=0.5	4	4	<=0.5	<=32	>8	<=0.25	>64	>2
>16	8	>16	>32	<=32	1	<=0.25	>64	<=0.5
<=0.5	8	16	<=0.5	<=32	2	<=0.25	>64	>2
<=0.5	>16	>16	16	>64	>8	<=0.25	64	>2
<=0.5	4	4	<=0.5	<=32	>8	<=0.25	>64	>2
<=0.5	4	4	<=0.5	<=32	1	<=0.25	<=4	<=0.5
<=0.5	8	8	<=0.5	<=32	2	<=0.25	>64	<=0.5
<=0.5	16	8	<=0.5	<=32	1	<=0.25	32	>2
<=0.5	8	8	<=0.5	<=32	1	<=0.25	>64	>2
<=0.5	8	8	<=0.5	<=32	4	<=0.25	>64	>2
<=0.5	4	4	<=0.5	<=32	1	<=0.25	<=4	>2
<=0.5	<=2	4	<=0.5	<=32	<=0.5	<=0.25	>64	>2
<=0.5	8	4	<=0.5	<=32	>8	<=0.25	>64	>2
<=0.5	8	>16	4	<=32	>8	<=0.25	>64	>2
<=0.5	4	4	1	<=32	2	<=0.25	<=4	<=0.5
<=0.5	4	4	<=0.5	<=32	1	<=0.25	<=4	>2
<=0.5	8	4	<=0.5	<=32	<=0.5	<=0.25	>64	>2
<=0.5	4	4	<=0.5	<=32	2	<=0.25	>64	<=0.5
<=0.5	4	2	<=0.5	64	<=0.5	<=0.25	<=4	<=0.5
<=0.5	4	2	<=0.5	>64	1	<=0.25	64	>2
<=0.5	4	8	<=0.5	<=32	<=0.5	<=0.25	>64	>2
1	>16	>16	32	<=32	>8	0.5	>64	>2
<=0.5	4	4	<=0.5	<=32	1	<=0.25	<=4	>2
<=0.5	4	4	<=0.5	<=32	2	<=0.25	<=4	<=0.5
<=0.5	<=2	2	<=0.5	<=32	1	<=0.25	<=4	<=0.5
1	>16	>16	16	>64	>8	0.5	>64	>2
<=0.5	>16	>16	8	<=32	>8	<=0.25	>64	>2
<=0.5	4	8	<=0.5	<=32	>8	<=0.25	>64	>2
<=0.5	8	8	<=0.5	<=32	>8	<=0.25	>64	>2
<=0.5	4	4	<=0.5	<=32	2	<=0.25	<=4	<=0.5
<=0.5	4	2	<=0.5	<=32	1	<=0.25	<=4	<=0.5
<=0.5	<=2	4	<=0.5	<=32	>8	<=0.25	>64	>2
<=0.5	4	4	<=0.5	<=32	<=0.5	<=0.25	<=4	<=0.5
<=0.5	8	4	<=0.5	<=32	<=0.5	<=0.25	<=4	<=0.5
<=0.5	4	4	<=0.5	<=32	>8	<=0.25	>64	<=0.5
<=0.5	4	4	<=0.5	<=32	1	<=0.25	<=4	>2
<=0.5	<=2	4	<=0.5	<=32	1	<=0.25	16	>2
<=0.5	4	4	<=0.5	<=32	<=0.5	<=0.25	64	<=0.5
<=0.5	<=2	8	<=0.5	<=32	>8	<=0.25	>64	<=0.5
<=0.5	<=2	2	<=0.5	<=32	<=0.5	<=0.25	64	>2
<=0.5	4	4	<=0.5	<=32	8	<=0.25	8	1
<=0.5	4	4	<=0.5	<=32	1	<=0.25	>64	<=0.5
<=0.5	<=2	2	<=0.5	<=32	1	<=0.25	>64	<=0.5
<=0.5	4	8	<=0.5	<=32	>8	<=0.25	64	<=0.5
<=0.5	4	8	<=0.5	<=32	2	<=0.25	>64	>2
<=0.5	4	8	<=0.5	<=32	>8	<=0.25	>64	>2
<=0.5	4	8	<=0.5	<=32	<=0.5	<=0.25	>64	>2
<=0.5	4	4	<=0.5	<=32	1	<=0.25	<=4	<=0.5
<=0.5	<=2	4	<=0.5	<=32	<=0.5	<=0.25	64	<=0.5
<=0.5	4	4	<=0.5	<=32	>8	<=0.25	64	>2
<=0.5	4	8	<=0.5	<=32	>8	<=0.25	64	>2
<=0.5	<=2	4	<=0.5	<=32	8	<=0.25	>64	>2
<=0.5	4	8	<=0.5	<=32	>8	<=0.25	64	>2
1	8	16	<=0.5	<=32	<=0.5	<=0.25	>64	<=0.5
<=0.5	<=2	4	<=0.5	<=32	<=0.5	<=0.25	>64	<=0.5
<=0.5	4	4	<=0.5	<=32	<=0.5	<=0.25	<=4	<=0.5
1	4	16	<=0.5	<=32	<=0.5	<=0.25	64	<=0.5
<=0.5	<=2	4	<=0.5	<=32	1	0.5	>64	>2
<=0.5	8	8	<=0.5	64	>8	<=0.25	>64	>2

<=0.5	<=2	8	<=0.5	<=32	1	<=0.25	64	<=0.5
<=0.5	<=2	4	<=0.5	<=32	>8	<=0.25	>64	>2
<=0.5	4	4	<=0.5	<=32	>8	<=0.25	>64	>2
<=0.5	<=2	<=1	<=0.5	<=32	<=0.5	<=0.25	<=4	<=0.5
<=0.5	<=2	8	<=0.5	<=32	<=0.5	<=0.25	>64	>2
<=0.5	<=2	2	<=0.5	<=32	1	<=0.25	>64	>2
<=0.5	<=2	2	<=0.5	<=32	<=0.5	<=0.25	<=4	<=0.5
<=0.5	8	8	<=0.5	<=32	1	<=0.25	>64	<=0.5
<=0.5	4	4	<=0.5	<=32	<=0.5	<=0.25	<=4	<=0.5
<=0.5	4	4	<=0.5	<=32	<=0.5	<=0.25	<=4	<=0.5
<=0.5	4	4	<=0.5	<=32	<=0.5	<=0.25	>64	>2
<=0.5	4	8	<=0.5	>64	<=0.5	<=0.25	>64	>2
<=0.5	<=2	8	<=0.5	<=32	2	<=0.25	>64	>2
<=0.5	<=2	4	<=0.5	<=32	2	<=0.25	>64	>2
<=0.5	8	8	<=0.5	<=32	<=0.5	<=0.25	<=4	>2
<=0.5	8	8	<=0.5	<=32	>8	<=0.25	>64	>2
<=0.5	<=2	16	<=0.5	<=32	8	<=0.25	>64	1
<=0.5	4	4	<=0.5	<=32	<=0.5	<=0.25	>64	>2
<=0.5	<=2	4	<=0.5	<=32	1	<=0.25	<=4	<=0.5
<=0.5	4	4	<=0.5	<=32	<=0.5	<=0.25	<=4	<=0.5
<=0.5	>16	>16	16	<=32	>8	<=0.25	>64	>2
<=0.5	4	4	<=0.5	<=32	<=0.5	<=0.25	<=4	<=0.5
<=0.5	4	4	<=0.5	<=32	<=0.5	<=0.25	>64	<=0.5
<=0.5	4	4	<=0.5	<=32	>8	<=0.25	<=4	>2
<=0.5	4	4	<=0.5	<=32	>8	<=0.25	>64	>2
<=0.5	>16	>16	8	<=32	1	<=0.25	>64	>2
<=0.5	>16	>16	8	<=32	>8	<=0.25	>64	<=0.5
<=0.5	4	4	<=0.5	<=32	1	<=0.25	>64	<=0.5
<=0.5	4	2	<=0.5	<=32	<=0.5	<=0.25	>64	<=0.5
<=0.5	<=2	4	<=0.5	<=32	1	<=0.25	<=4	<=0.5
<=0.5	4	2	<=0.5	<=32	<=0.5	<=0.25	>64	<=0.5
<=0.5	<=2	2	<=0.5	<=32	<=0.5	<=0.25	>64	>2
<=0.5	>16	>16	16	<=32	>8	<=0.25	>64	>2
<=0.5	<=2	4	<=0.5	<=32	8	<=0.25	>64	>2
<=0.5	<=2	4	<=0.5	<=32	<=0.5	<=0.25	>64	<=0.5
<=0.5	4	4	<=0.5	<=32	2	<=0.25	>64	<=0.5
<=0.5	<=2	2	<=0.5	<=32	<=0.5	<=0.25	>64	>2
<=0.5	<=2	<=1	<=0.5	<=32	<=0.5	<=0.25	>64	>2
<=0.5	<=2	4	<=0.5	<=32	<=0.5	<=0.25	>64	<=0.5
<=0.5	8	4	<=0.5	<=32	<=0.5	<=0.25	>64	<=0.5
<=0.5	4	2	<=0.5	<=32	<=0.5	<=0.25	>64	>2
<=0.5	<=2	2	<=0.5	<=32	<=0.5	<=0.25	<=4	<=0.5
<=0.5	<=2	4	<=0.5	<=32	<=0.5	<=0.25	<=4	<=0.5
<=0.5	>16	>16	16	<=32	2	<=0.25	>64	>2
<=0.5	<=2	4	<=0.5	<=32	<=0.5	<=0.25	>64	<=0.5
<=0.5	16	16	<=0.5	<=32	>8	<=0.25	>64	>2
<=0.5	<=2	4	<=0.5	<=32	<=0.5	<=0.25	<=4	>2
<=0.5	<=2	8	<=0.5	<=32	>8	<=0.25	>64	>2
<=0.5	4	4	<=0.5	>64	8	<=0.25	>64	>2
<=0.5	<=2	4	<=0.5	<=32	1	<=0.25	>64	>2
<=0.5	<=2	8	<=0.5	<=32	1	<=0.25	<=4	>2
<=0.5	<=2	2	<=0.5	<=32	<=0.5	<=0.25	>64	>2
<=0.5	4	4	<=0.5	<=32	<=0.5	<=0.25	<=4	<=0.5
<=0.5	4	4	<=0.5	<=32	<=0.5	<=0.25	>64	<=0.5
<=0.5	>16	>16	32	<=32	>8	<=0.25	>64	<=0.5
<=0.5	4	4	<=0.5	<=32	1	<=0.25	>64	<=0.5
<=0.5	<=2	4	<=0.5	<=32	<=0.5	<=0.25	<=4	<=0.5
<=0.5	4	8	<=0.5	<=32	1	<=0.25	>64	>2
<=0.5	8	8	<=0.5	<=32	1	<=0.25	>64	<=0.5
<=0.5	<=2	2	<=0.5	<=32	1	<=0.25	>64	>2
<=0.5	4	4	<=0.5	<=32	<=0.5	<=0.25	<=4	<=0.5

<=0.5	8	4	<=0.5	<=32	>8	<=0.25	>64	<=0.5
<=0.5	4	8	<=0.5	<=32	4	<=0.25	>64	>2
<=0.5	4	2	<=0.5	<=32	1	<=0.25	>64	<=0.5
<=0.5	4	4	<=0.5	<=32	1	<=0.25	<=4	<=0.5
<=0.5	8	8	<=0.5	<=32	1	<=0.25	>64	<=0.5
<=0.5	4	4	<=0.5	<=32	4	<=0.25	<=4	<=0.5
<=0.5	<=2	4	<=0.5	<=32	<=0.5	<=0.25	<=4	<=0.5
<=0.5	8	4	<=0.5	<=32	>8	<=0.25	>64	>2
<=0.5	<=2	<=1	<=0.5	<=32	4	<=0.25	<=4	<=0.5
<=0.5	<=2	2	<=0.5	<=32	>8	<=0.25	64	>4
<=0.5	4	4	<=0.5	<=32	1	<=0.25	>64	<=0.5
<=0.5	4	4	<=0.5	<=32	<=0.5	<=0.25	>64	>4
<=0.5	4	4	<=0.5	<=32	1	<=0.25	<=4	<=0.5
<=0.5	4	4	<=0.5	<=32	>8	<=0.25	>64	>4
<=0.5	<=2	4	<=0.5	<=32	<=0.5	<=0.25	>64	>4
<=0.5	<=2	2	<=0.5	<=32	1	<=0.25	>64	<=0.5
<=0.5	<=2	8	<=0.5	<=32	<=0.5	<=0.25	>64	>4
<=0.5	<=2	4	<=0.5	<=32	1	<=0.25	>64	<=0.5
<=0.5	<=2	<=1	<=0.5	<=32	1	<=0.25	<=4	<=0.5
<=0.5	4	4	<=0.5	<=32	1	<=0.25	>64	>4
<=0.5	4	4	<=0.5	<=32	1	<=0.25	>64	<=0.5
<=0.5	4	4	<=0.5	<=32	>8	<=0.25	>64	>4
<=0.5	>16	>16	8	<=32	4	<=0.25	>64	<=0.5
<=0.5	<=2	4	<=0.5	<=32	2	<=0.25	>64	<=0.5
<=0.5	4	4	<=0.5	<=32	1	<=0.25	>64	>4
<=0.5	<=2	4	<=0.5	<=32	<=0.5	<=0.25	<=4	<=0.5
<=0.5	4	2	<=0.5	<=32	1	<=0.25	>64	>4
<=0.5	4	4	<=0.5	<=32	1	<=0.25	>64	<=0.5
<=0.5	4	8	<=0.5	<=32	1	<=0.25	>64	<=0.5
<=0.5	4	4	<=0.5	<=32	1	<=0.25	<=4	<=0.5
<=0.5	4	4	<=0.5	<=32	2	<=0.25	>64	>4
<=0.5	4	4	<=0.5	<=32	>8	<=0.25	<=4	>4
<=0.5	4	2	<=0.5	<=32	<=0.5	<=0.25	<=4	<=0.5
<=0.5	8	2	<=0.5	<=32	1	<=0.25	<=4	<=0.5
<=0.5	4	4	<=0.5	<=32	>8	<=0.25	64	>2
<=0.5	8	8	<=0.5	<=32	1	<=0.25	>64	>2
<=0.5	<=2	2	<=0.5	<=32	<=0.5	<=0.25	>64	>2
<=0.5	8	8	<=0.5	<=32	<=0.5	<=0.25	>64	<=0.5
<=0.5	4	8	<=0.5	<=32	<=0.5	<=0.25	>64	>2
<=0.5	<=2	2	<=0.5	<=32	<=0.5	<=0.25	>64	<=0.5
<=0.5	<=2	4	<=0.5	<=32	1	<=0.25	>64	>2
<=0.5	8	8	<=0.5	<=32	<=0.5	<=0.25	64	>2
<=0.5	4	4	<=0.5	<=32	<=0.5	<=0.25	>64	>2
<=0.5	4	4	1	<=32	<=0.5	<=0.25	>64	<=0.5
<=0.5	4	4	<=0.5	<=32	<=0.5	<=0.25	<=4	<=0.5
<=0.5	>16	>16	8	<=32	<=0.5	<=0.25	32	<=0.5
<=0.5	8	8	<=0.5	<=32	<=0.5	<=0.25	>64	>2
<=0.5	<=2	2	<=0.5	<=32	1	<=0.25	<=4	<=0.5
<=0.5	4	8	<=0.5	<=32	1	<=0.25	>64	>2
<=0.5	<=2	4	<=0.5	<=32	<=0.5	<=0.25	<=4	<=0.5
<=0.5	4	4	<=0.5	<=32	>8	<=0.25	>64	>2
<=0.5	4	2	<=0.5	<=32	<=0.5	<=0.25	>64	<=0.5
<=0.5	4	4	<=0.5	<=32	>8	<=0.25	>64	>2
<=0.5	4	8	<=0.5	<=32	>8	<=0.25	>64	>2
<=0.5	4	4	<=0.5	<=32	<=0.5	<=0.25	>64	<=0.5
<=0.5	8	8	<=0.5	<=32	<=0.5	<=0.25	<=4	<=0.5
<=0.5	8	8	<=0.5	>64	1	<=0.25	>64	>2
<=0.5	4	4	<=0.5	<=32	1	<=0.25	32	>2
<=0.5	8	4	<=0.5	<=32	<=0.5	<=0.25	>64	>2
<=0.5	<=2	<=1	<=0.5	<=32	<=0.5	<=0.25	<=4	<=0.5
<=0.5	4	4	<=0.5	<=32	>8	<=0.25	>64	>2

<=0.5	4	8	<=0.5	<=32	1	<=0.25	>64	<=0.5
<=0.5	4	8	<=0.5	<=32	1	<=0.25	>64	<=0.5
<=0.5	<=2	4	<=0.5	<=32	1	<=0.25	<=4	<=0.5
<=0.5	<=2	4	<=0.5	<=32	1	<=0.25	<=4	<=0.5
<=0.5	<=2	<=1	<=0.5	<=32	<=0.5	<=0.25	<=4	<=0.5
<=0.5	<=2	4	<=0.5	<=32	1	<=0.25	>64	>4
<=0.5	4	4	<=0.5	<=32	>8	<=0.25	>64	<=0.5
<=0.5	<=2	8	<=0.5	<=32	<=0.5	<=0.25	<=4	<=0.5
<=0.5	4	4	<=0.5	<=32	2	<=0.25	<=4	<=0.5
<=0.5	4	2	<=0.5	<=32	2	<=0.25	<=4	<=0.5
<=0.5	<=2	4	<=0.5	<=32	1	<=0.25	<=4	<=0.5
<=0.5	4	4	<=0.5	<=32	2	<=0.25	>64	>4
<=0.5	<=2	4	<=0.5	<=32	<=0.5	0.5	<=4	>4
<=0.5	4	4	<=0.5	<=32	<=0.5	<=0.25	>64	>4
<=0.5	8	4	<=0.5	<=32	>8	<=0.25	32	>4
<=0.5	4	4	<=0.5	<=32	2	<=0.25	>64	<=0.5
<=0.5	4	8	<=0.5	<=32	>8	<=0.25	>64	>4
<=0.5	4	4	<=0.5	<=32	<=0.5	<=0.25	>64	>4
<=0.5	<=2	8	<=0.5	<=32	1	<=0.25	<=4	<=0.5
<=0.5	4	8	<=0.5	<=32	>8	<=0.25	>64	>4
<=0.5	8	8	<=0.5	<=32	1	<=0.25	<=4	>4
<=0.5	4	4	<=0.5	<=32	1	<=0.25	<=4	<=0.5
<=0.5	4	4	<=0.5	<=32	1	<=0.25	<=4	>4
<=0.5	4	8	<=0.5	<=32	1	<=0.25	>64	<=0.5
<=0.5	<=2	4	<=0.5	<=32	1	<=0.25	>64	<=0.5
<=0.5	8	4	<=0.5	<=32	1	<=0.25	>64	<=0.5
<=0.5	4	4	<=0.5	<=32	<=0.5	<=0.25	<=4	<=0.5
<=0.5	8	8	<=0.5	<=32	<=0.5	<=0.25	>64	>2
<=0.5	>16	>16	32	<=32	>8	<=0.25	>64	>2
<=0.5	4	4	<=0.5	<=32	2	<=0.25	>64	<=0.5
<=0.5	<=2	16	<=0.5	<=32	2	0.5	>64	<=0.5
<=0.5	8	8	<=0.5	<=32	<=0.5	<=0.25	>64	>2
<=0.5	16	16	<=0.5	<=32	1	<=0.25	<=4	>2
<=0.5	4	4	<=0.5	<=32	4	<=0.25	>64	>2
<=0.5	4	8	<=0.5	<=32	<=0.5	<=0.25	>64	>2
<=0.5	4	2	<=0.5	<=32	<=0.5	<=0.25	>64	<=0.5
<=0.5	4	<=1	<=0.5	<=32	<=0.5	<=0.25	64	<=0.5
<=0.5	<=2	4	<=0.5	<=32	8	<=0.25	>64	>4
<=0.5	4	8	<=0.5	<=32	2	<=0.25	>64	>2
>16	8	>16	>32	<=32	1	<=0.25	>64	<=0.5
<=0.5	>16	>16	8	<=32	>8	<=0.25	>64	>2
<=0.5	4	4	<=0.5	<=32	1	<=0.25	<=4	<=0.5
<=0.5	<=2	8	<=0.5	<=32	<=0.5	<=0.25	<=4	>2
<=0.5	<=2	4	<=0.5	<=32	>8	<=0.25	<=4	>2
<=0.5	4	4	<=0.5	<=32	<=0.5	<=0.25	<=4	<=0.5
<=0.5	<=2	2	<=0.5	<=32	>8	<=0.25	>64	>2
<=0.5	8	4	1	<=32	1	<=0.25	>64	<=0.5
<=0.5	<=2	4	<=0.5	<=32	1	<=0.25	>64	<=0.5
<=0.5	4	8	<=0.5	<=32	1	<=0.25	>64	<=0.5
<=0.5	4	4	<=0.5	<=32	8	<=0.25	>64	>2
<=0.5	<=2	2	<=0.5	<=32	<=0.5	<=0.25	<=4	<=0.5
<=0.5	4	4	<=0.5	<=32	4	<=0.25	<=4	<=0.5
1	>16	>16	32	64	>8	0.5	>64	>2
<=0.5	8	16	<=0.5	<=32	>8	<=0.25	>64	>2
<=0.5	4	4	<=0.5	<=32	<=0.5	<=0.25	>64	<=0.5
<=0.5	<=2	8	<=0.5	<=32	2	<=0.25	>64	>2
2	>16	>16	>32	<=32	>8	1	>64	>2
<=0.5	8	4	<=0.5	<=32	>8	<=0.25	>64	>2
<=0.5	<=2	2	<=0.5	<=32	1	<=0.25	>64	<=0.5
<=0.5	4	8	<=0.5	<=32	>8	<=0.25	>64	>2
<=0.5	4	8	<=0.5	<=32	<=0.5	<=0.25	>64	>2

<=0.5	<=2	4	<=0.5	<=32	1	<=0.25	<=4	<=0.5
<=0.5	4	2	<=0.5	<=32	<=0.5	<=0.25	<=4	<=0.5
<=0.5	<=2	2	<=0.5	<=32	1	<=0.25	>64	>2
<=0.5	8	4	<=0.5	<=32	1	<=0.25	<=4	>4
<=0.5	<=2	8	<=0.5	<=32	1	<=0.25	<=4	<=0.5
<=0.5	4	8	<=0.5	<=32	>8	<=0.25	>64	>2
<=0.5	<=2	2	<=0.5	<=32	1	0.5	<=4	<=0.5
<=0.5	<=2	4	<=0.5	<=32	1	<=0.25	<=4	<=0.5
<=0.5	<=2	8	<=0.5	<=32	1	<=0.25	<=4	>2
<=0.5	<=2	4	<=0.5	<=32	<=0.5	<=0.25	64	>2
<=0.5	4	4	<=0.5	<=32	2	<=0.25	>64	>4
<=0.5	8	4	<=0.5	<=32	2	<=0.25	>64	<=0.5
<=0.5	16	8	<=0.5	<=32	<=0.5	<=0.25	<=4	<=0.5
<=0.5	<=2	<=1	<=0.5	<=32	1	<=0.25	<=4	<=0.5
<=0.5	<=2	2	<=0.5	<=32	<=0.5	<=0.25	<=4	<=0.5
<=0.5	>16	>16	8	<=32	>8	<=0.25	>64	>4
<=0.5	8	8	<=0.5	<=32	>8	<=0.25	>64	>4
<=0.5	16	8	<=0.5	<=32	2	<=0.25	>64	1
<=0.5	>16	>16	16	>64	>8	<=0.25	>64	>4
<=0.5	4	4	<=0.5	<=32	1	<=0.25	>64	>4
<=0.5	4	4	<=0.5	<=32	1	<=0.25	>64	>4
<=0.5	4	4	<=0.5	<=32	2	<=0.25	<=4	<=0.5
<=0.5	16	16	<=0.5	64	>8	<=0.25	>64	>4
<=0.5	4	4	<=0.5	<=32	>8	<=0.25	>64	>4
<=0.5	<=2	4	<=0.5	<=32	2	<=0.25	<=4	<=0.5
<=0.5	<=2	4	<=0.5	<=32	1	<=0.25	>64	>4
<=0.5	8	8	<=0.5	<=32	<=0.5	<=0.25	<=4	<=0.5
<=0.5	8	4	<=0.5	<=32	>8	<=0.25	>64	>4
<=0.5	8	8	<=0.5	<=32	1	<=0.25	<=4	<=0.5
<=0.5	<=2	4	<=0.5	<=32	1	<=0.25	>64	>2
<=0.5	8	4	<=0.5	<=32	1	<=0.25	<=4	<=0.5
<=0.5	4	8	<=0.5	<=32	>8	<=0.25	>64	>2
<=0.5	<=2	2	<=0.5	<=32	1	<=0.25	>64	<=0.5
<=0.5	4	8	<=0.5	<=32	>8	<=0.25	>64	<=0.5
<=0.5	4	4	<=0.5	<=32	<=0.5	<=0.25	64	>2
<=0.5	16	8	<=0.5	<=32	>8	<=0.25	>64	2
<=0.5	<=2	4	<=0.5	<=32	1	<=0.25	>64	<=0.5
<=0.5	<=2	4	<=0.5	<=32	1	<=0.25	>64	<=0.5
<=0.5	<=2	4	<=0.5	<=32	<=0.5	<=0.25	64	>2
<=0.5	<=2	4	<=0.5	<=32	>8	<=0.25	>64	>2
<=0.5	4	8	<=0.5	<=32	<=0.5	<=0.25	64	<=0.5
<=0.5	<=2	2	<=0.5	<=32	<=0.5	<=0.25	64	>2
<=0.5	<=2	2	<=0.5	<=32	1	<=0.25	<=4	<=0.5
<=0.5	<=2	2	<=0.5	<=32	<=0.5	<=0.25	>64	>2
<=0.5	<=2	4	<=0.5	<=32	<=0.5	<=0.25	<=4	<=0.5
<=0.5	4	4	<=0.5	<=32	<=0.5	<=0.25	<=4	<=0.5
<=0.5	8	8	<=0.5	<=32	1	<=0.25	>64	>2
<=0.5	4	4	<=0.5	<=32	>8	<=0.25	>64	>2
<=0.5	8	4	<=0.5	<=32	<=0.5	<=0.25	>64	1
<=0.5	>16	>16	16	<=32	<=0.5	<=0.25	32	<=0.5
<=0.5	4	4	<=0.5	<=32	<=0.5	<=0.25	<=4	<=0.5
<=0.5	<=2	4	<=0.5	<=32	<=0.5	<=0.25	>64	<=0.5
<=0.5	<=2	4	<=0.5	<=32	1	<=0.25	<=4	<=0.5
<=0.5	<=2	4	<=0.5	<=32	2	<=0.25	>64	>2
<=0.5	<=2	2	<=0.5	<=32	<=0.5	<=0.25	>64	>2
<=0.5	4	8	<=0.5	<=32	1	<=0.25	>64	<=0.5
<=0.5	4	4	<=0.5	<=32	1	<=0.25	<=4	<=0.5
<=0.5	>16	>16	1	>64	<=0.5	<=0.25	8	>2
<=0.5	8	8	<=0.5	<=32	1	<=0.25	<=4	<=0.5
<=0.5	4	4	<=0.5	<=32	<=0.5	<=0.25	<=4	<=0.5
<=0.5	8	4	<=0.5	<=32	>8	<=0.25	>64	<=0.5

	<=0.5		<=2	4	<=0.5	<=32	<=0.5	<=0.25	>64	<=0.5
	<=0.5		<=2	4	<=0.5	<=32	1	<=0.25	<=4	>2
	<=0.5		16	4	<=0.5	<=32	>8	<=0.25	64	>2
	<=0.5		4	4	<=0.5	<=32	1	<=0.25	<=4	>2
	<=0.5		<=2	2	<=0.5	<=32	1	<=0.25	>64	<=0.5
	<=0.5		4	4	<=0.5	<=32	2	<=0.25	>64	<=0.5
	<=0.5		<=2	4	<=0.5	<=32	<=0.5	<=0.25	>64	>2
	<=0.5		4	4	<=0.5	<=32	1	<=0.25	>64	<=0.5
	<=0.5		4	4	<=0.5	<=32	1	<=0.25	<=4	>2
	<=0.5		8	4	<=0.5	<=32	1	<=0.25	<=4	<=0.5
	<=0.5		8	4	<=0.5	<=32	2	<=0.25	<=4	<=0.5
	<=0.5		4	4	<=0.5	<=32	<=0.5	<=0.25	<=4	<=0.5
	<=0.5		4	8	<=0.5	<=32	1	<=0.25	>64	<=0.5
	<=0.5		4	4	<=0.5	<=32	1	<=0.25	<=4	<=0.5
	<=0.5		<=2	4	<=0.5	<=32	1	<=0.25	<=4	<=0.5
	<=0.5		<=2	4	<=0.5	<=32	1	<=0.25	<=4	<=0.5
	<=0.5		8	4	<=0.5	<=32	>8	<=0.25	>64	>2
	<=0.5		8	4	<=0.5	<=32	1	<=0.25	64	>2
	<=0.5		4	4	<=0.5	<=32	<=0.5	<=0.25	>64	<=0.5
	8		4	>16	2	<=32	1	<=0.25	>64	>2
	<=0.5		4	4	<=0.5	<=32	1	<=0.25	<=4	<=0.5
	<=0.5		<=2	8	<=0.5	<=32	<=0.5	<=0.25	64	>2
	>16		>16	>16	>32	<=32	>8	<=0.25	>64	<=0.5
	<=0.5		4	8	<=0.5	<=32	1	<=0.25	<=4	<=0.5
	<=0.5		8	4	<=0.5	<=32	1	<=0.25	>64	<=0.5
	<=0.5		<=2	2	<=0.5	<=32	<=0.5	<=0.25	>64	>2
	4		8	>16	>32	<=32	>8	<=0.25	>64	>2
	<=0.5		<=2	4	<=0.5	<=32	<=0.5	<=0.25	<=4	<=0.5
	<=0.5		4	2	<=0.5	<=32	<=0.5	<=0.25	16	>2
	<=0.5		<=2	16	<=0.5	<=32	<=0.5	<=0.25	64	<=0.5
	<=0.5		8	4	<=0.5	<=32	>8	<=0.25	>64	>2
	<=0.5		<=2	4	<=0.5	<=32	<=0.5	<=0.25	>64	<=0.5
	<=0.5		<=2	4	<=0.5	<=32	1	<=0.25	64	<=0.5
	<=0.5		4	4	<=0.5	<=32	2	<=0.25	<=4	<=0.5
	<=0.5		4	4	<=0.5	<=32	>8	<=0.25	>64	>2
<=0.25	<=0.5		8	8	<=0.5	<=32	4	<=0.25	16	<=0.25
	<=0.5		<=2	4	<=0.5	<=32	<=0.5	<=0.25	64	>2
	<=0.5		<=2	4	<=0.5	<=32	<=0.5	<=0.25	<=4	<=0.5
	<=0.5		4	4	<=0.5	<=32	1	<=0.25	>64	>2
	<=0.5		<=2	4	<=0.5	<=32	1	<=0.25	<=4	<=0.5
	<=0.5		8	8	<=0.5	<=32	>8	<=0.25	64	<=0.5
	<=0.5		4	4	<=0.5	<=32	8	<=0.25	>64	>2
	<=0.5		8	8	<=0.5	<=32	>8	<=0.25	>64	>2
	<=0.5		4	4	<=0.5	<=32	>8	<=0.25	>64	>2
	<=0.5		8	4	<=0.5	<=32	2	<=0.25	8	<=0.5
	<=0.5		<=2	2	<=0.5	<=32	<=0.5	<=0.25	>64	>4
	<=0.5		<=2	8	<=0.5	<=32	<=0.5	<=0.25	<=4	<=0.5
	2		>16	>16	>32	64	>8	0.5	>64	>4
	<=0.5		<=2	4	<=0.5	<=32	2	<=0.25	<=4	<=0.5
	<=0.5		8	8	<=0.5	<=32	>8	<=0.25	>64	>4
	<=0.5		4	4	<=0.5	<=32	1	<=0.25	<=4	<=0.5
	<=0.5		<=2	8	<=0.5	<=32	>8	<=0.25	>64	>4
	<=0.5		8	8	<=0.5	<=32	<=0.5	<=0.25	>64	>4
	<=0.5		4	4	<=0.5	<=32	<=0.5	<=0.25	<=4	>4
	<=0.5		4	4	<=0.5	<=32	<=0.5	<=0.25	<=4	<=0.5
	<=0.5		<=2	2	<=0.5	<=32	>8	<=0.25	>64	>4
	<=0.5		4	4	<=0.5	<=32	2	<=0.25	32	>4
	<=0.5		4	4	<=0.5	<=32	1	<=0.25	>64	<=0.5
	<=0.5		16	16	4	<=32	<=0.5	<=0.25	>64	>4
	<=0.5		8	8	<=0.5	<=32	>8	<=0.25	64	>4
	4		>16	>16	>32	<=32	<=0.5	1	>64	<=0.5

4	16	>16	32	<=32	>8	<=0.25	>64	<=0.5
>16	>16	>16	>32	<=32	>8	1	>64	>2
<=0.5	4	4	<=0.5	<=32	2	<=0.25	<=4	<=0.5
4	>16	>16	>32	<=32	>8	<=0.25	>64	>2
<=0.5	>16	>16	8	<=32	<=0.5	0.5	32	>2
<=0.5	<=2	4	<=0.5	<=32	>8	<=0.25	>64	<=0.5
<=0.5	4	4	<=0.5	<=32	<=0.5	<=0.25	>64	<=0.5
<=0.5	>16	>16	8	64	8	<=0.25	>64	>2
<=0.5	4	8	2	<=32	<=0.5	<=0.25	<=4	>2
<=0.5	4	4	<=0.5	<=32	<=0.5	<=0.25	>64	<=0.5
<=0.5	16	16	4	<=32	>8	<=0.25	>64	>2
<=0.5	<=2	8	<=0.5	<=32	1	<=0.25	>64	>2
<=0.5	4	4	<=0.5	<=32	>8	<=0.25	>64	>2
<=0.5	4	4	<=0.5	<=32	<=0.5	<=0.25	32	<=0.5
<=0.5	4	4	<=0.5	<=32	<=0.5	<=0.25	>64	>2
>16	>16	>16	>32	<=32	>8	0.5	>64	>2
>16	>16	>16	>32	<=32	>8	<=0.25	>64	>2
1	4	>16	<=0.5	<=32	>8	<=0.25	64	>2
<=0.5	<=2	2	<=0.5	<=32	1	<=0.25	>64	<=0.5
<=0.5	<=2	2	<=0.5	<=32	1	<=0.25	<=4	<=0.5
<=0.5	<=2	2	<=0.5	<=32	<=0.5	<=0.25	<=4	<=0.5
<=0.5	4	8	<=0.5	<=32	>8	<=0.25	>64	>2
<=0.5	8	4	<=0.5	<=32	<=0.5	<=0.25	>64	>2
16	4	>16	>32	<=32	>8	<=0.25	>64	>2
<=0.5	<=2	2	<=0.5	<=32	1	<=0.25	64	>2
<=0.5	4	4	<=0.5	<=32	1	<=0.25	<=4	<=0.5
<=0.5	4	4	<=0.5	<=32	<=0.5	<=0.25	>64	>4
<=0.5	8	4	<=0.5	<=32	>8	<=0.25	>64	>4
<=0.5	>16	>16	4	<=32	1	<=0.25	>64	<=0.5
2	>16	>16	>32	>64	>8	0.5	>64	>4
<=0.5	4	4	<=0.5	<=32	>8	<=0.25	>64	>4
<=0.5	16	8	<=0.5	<=32	1	<=0.25	64	>4
<=0.5	4	2	<=0.5	<=32	<=0.5	<=0.25	>64	<=0.5
<=0.5	4	4	<=0.5	<=32	2	<=0.25	64	<=0.5
<=0.5	4	16	<=0.5	<=32	1	<=0.25	>64	>4
<=0.5	4	4	<=0.5	<=32	2	<=0.25	<=4	>4
<=0.5	4	8	<=0.5	<=32	1	<=0.25	<=4	<=0.5
<=0.5	>16	>16	1	<=32	>8	<=0.25	64	<=0.5
<=0.5	<=2	4	<=0.5	<=32	>8	<=0.25	<=4	>4
<=0.5	<=2	2	<=0.5	<=32	<=0.5	<=0.25	<=4	<=0.5
<=0.5	>16	>16	32	<=32	>8	<=0.25	>64	>4
<=0.5	8	4	<=0.5	<=32	1	<=0.25	<=4	1
<=0.5	>16	>16	16	<=32	2	<=0.25	>64	>4
<=0.5	>16	>16	16	<=32	>8	<=0.25	>64	<=0.5
<=0.5	<=2	4	<=0.5	<=32	1	<=0.25	>64	>4
<=0.5	4	4	<=0.5	<=32	>8	<=0.25	<=4	>4
<=0.5	4	4	<=0.5	<=32	2	<=0.25	<=4	<=0.5
<=0.5	8	8	<=0.5	<=32	1	<=0.25	>64	<=0.5
<=0.5	4	4	<=0.5	<=32	1	<=0.25	>64	>4
<=0.5	16	8	<=0.5	<=32	1	<=0.25	>64	<=0.5
<=0.5	4	4	<=0.5	<=32	1	<=0.25	>64	<=0.5
<=0.5	8	4	<=0.5	<=32	>8	<=0.25	>64	>4
<=0.5	4	4	<=0.5	<=32	1	<=0.25	<=4	<=0.5
<=0.5	16	8	<=0.5	<=32	1	0.5	<=4	<=0.5
<=0.5	8	8	<=0.5	<=32	1	<=0.25	<=4	<=0.5
<=0.5	4	4	<=0.5	<=32	<=0.5	<=0.25	<=4	<=0.5
<=0.5	8	8	<=0.5	64	1	<=0.25	32	<=0.5
<=0.5	4	4	<=0.5	<=32	1	<=0.25	>64	>4
<=0.5	8	8	<=0.5	<=32	>8	<=0.25	>64	<=0.5
<=0.5	4	4	<=0.5	<=32	1	<=0.25	<=4	<=0.5
<=0.5	4	4	<=0.5	<=32	<=0.5	<=0.25	<=4	<=0.5

<=0.5	4	4	<=0.5	<=32	1	<=0.25	>64	>4
<=0.5	4	8	<=0.5	<=32	1	<=0.25	<=4	<=0.5
>16	16	>16	>32	<=32	>8	0.5	>64	>4
<=0.5	4	8	<=0.5	<=32	1	<=0.25	<=4	<=0.5
<=0.5	>16	>16	16	<=32	>8	<=0.25	>64	>4
<=0.5	<=2	4	<=0.5	<=32	1	<=0.25	>64	>4
<=0.5	8	4	<=0.5	<=32	1	<=0.25	>64	>4
<=0.5	8	8	<=0.5	<=32	1	<=0.25	<=4	<=0.5
<=0.5	<=2	4	<=0.5	<=32	4	<=0.25	>64	<=0.5
<=0.5	8	4	<=0.5	<=32	1	<=0.25	>64	>4
<=0.5	4	4	<=0.5	<=32	1	<=0.25	<=4	<=0.5
<=0.5	<=2	2	<=0.5	<=32	<=0.5	<=0.25	>64	>4
<=0.5	4	4	<=0.5	<=32	1	<=0.25	>64	>4
<=0.5	<=2	4	<=0.5	<=32	<=0.5	<=0.25	<=4	<=0.5
<=0.5	4	8	<=0.5	<=32	2	<=0.25	>64	>4
<=0.5	8	8	<=0.5	<=32	>8	<=0.25	>64	>4
<=0.5	<=2	4	<=0.5	<=32	1	<=0.25	>64	<=0.5
<=0.5	>16	>16	32	<=32	>8	0.5	>64	>2
<=0.5	4	4	<=0.5	<=32	>8	<=0.25	>64	<=0.5
<=0.5	>16	>16	8	64	<=0.5	<=0.25	>64	>2
<=0.5	<=2	4	<=0.5	64	2	<=0.25	>64	<=0.5
<=0.5	4	4	<=0.5	>64	<=0.5	<=0.25	>64	<=0.5
<=0.5	>16	16	<=0.5	<=32	>8	0.5	64	>2
<=0.5	4	4	<=0.5	<=32	1	<=0.25	>64	<=0.5
<=0.5	>16	>16	<=0.5	<=32	1	<=0.25	>64	<=0.5
<=0.5	>16	16	4	<=32	1	<=0.25	32	<=0.5
<=0.5	8	4	<=0.5	<=32	2	<=0.25	>64	<=0.5
<=0.5	4	8	<=0.5	<=32	1	<=0.25	<=4	<=0.5
<=0.5	<=2	2	<=0.5	<=32	1	<=0.25	<=4	<=0.5
<=0.5	>16	>16	16	<=32	1	<=0.25	>64	>4
<=0.5	8	>16	<=0.5	<=32	2	<=0.25	>64	>4
<=0.5	4	4	<=0.5	<=32	1	<=0.25	<=4	<=0.5
<=0.5	<=2	2	<=0.5	<=32	<=0.5	<=0.25	>64	>4
<=0.5	8	16	<=0.5	<=32	>8	<=0.25	>64	>4
<=0.5	4	2	<=0.5	<=32	8	<=0.25	>64	>4
<=0.5	<=2	4	<=0.5	64	1	<=0.25	<=4	>4
<=0.5	4	4	<=0.5	<=32	1	<=0.25	<=4	<=0.5
<=0.5	<=2	4	<=0.5	<=32	1	<=0.25	<=4	<=0.5
>16	>16	>16	>32	<=32	>8	<=0.25	>64	>4
<=0.5	4	4	<=0.5	<=32	<=0.5	<=0.25	>64	>4
<=0.5	8	4	<=0.5	<=32	1	<=0.25	>64	>4
<=0.5	<=2	8	<=0.5	<=32	8	<=0.25	>64	>4
8	8	>16	>32	<=32	>8	<=0.25	>64	>4
<=0.5	<=2	4	<=0.5	<=32	<=0.5	<=0.25	<=4	>4
<=0.5	4	4	1	<=32	>8	<=0.25	>64	>4
<=0.5	<=2	4	<=0.5	<=32	<=0.5	<=0.25	<=4	<=0.5
<=0.5	<=2	4	<=0.5	<=32	<=0.5	<=0.25	<=4	<=0.5
<=0.5	4	4	<=0.5	<=32	1	<=0.25	>64	>4
<=0.5	4	4	<=0.5	<=32	<=0.5	<=0.25	>64	<=0.5
<=0.5	4	4	<=0.5	<=32	>8	<=0.25	>64	>4
<=0.5	4	4	<=0.5	<=32	<=0.5	<=0.25	>64	>4
<=0.5	<=2	4	<=0.5	<=32	<=0.5	<=0.25	>64	>4
1	>16	>16	8	<=32	1	<=0.25	>64	<=0.5
1	16	16	<=0.5	<=32	>8	<=0.25	>64	>4
<=0.5	4	2	<=0.5	<=32	<=0.5	<=0.25	<=4	>4
<=0.5	<=2	4	<=0.5	<=32	<=0.5	<=0.25	>64	>4
<=0.5	8	8	<=0.5	<=32	2	<=0.25	>64	>4
<=0.5	8	4	<=0.5	<=32	1	<=0.25	>64	<=0.5
<=0.5	8	8	<=0.5	64	>8	<=0.25	>64	>4
<=0.5	4	8	<=0.5	<=32	2	<=0.25	>64	>4
<=0.5	4	4	<=0.5	<=32	1	<=0.25	<=4	<=0.5

<=0.5	4	8	<=0.5	<=32	4	<=0.25	>64	<=0.5
<=0.5	4	4	<=0.5	<=32	8	<=0.25	>64	>4
<=0.5	4	4	<=0.5	<=32	8	<=0.25	>64	>4
<=0.5	4	4	<=0.5	<=32	1	<=0.25	>64	<=0.5
<=0.5	4	4	<=0.5	<=32	<=0.5	<=0.25	>64	>4
>16	4	>16	>32	<=32	>8	<=0.25	>64	>4
<=0.5	4	8	<=0.5	<=32	1	<=0.25	64	<=0.5
<=0.5	4	4	<=0.5	<=32	<=0.5	<=0.25	>64	<=0.5
<=0.5	<=2	4	<=0.5	<=32	<=0.5	<=0.25	>64	>2
<=0.5	4	2	<=0.5	<=32	<=0.5	<=0.25	<=4	<=0.5
<=0.5	4	4	<=0.5	<=32	>8	<=0.25	>64	>4
<=0.5	4	8	<=0.5	<=32	>8	<=0.25	>64	>4
<=0.5	<=2	4	<=0.5	<=32	4	<=0.25	>64	<=0.5
<=0.5	4	4	<=0.5	<=32	<=0.5	<=0.25	>64	<=0.5
<=0.5	4	4	<=0.5	<=32	1	<=0.25	<=4	<=0.5
<=0.5	<=2	2	<=0.5	<=32	<=0.5	<=0.25	<=4	<=0.5
<=0.5	4	4	<=0.5	<=32	>8	<=0.25	>64	<=0.5
8	>16	>16	>32	<=32	>8	<=0.25	>64	>4
<=0.5	8	8	<=0.5	<=32	<=0.5	<=0.25	>64	>4
<=0.5	4	2	<=0.5	<=32	<=0.5	<=0.25	>64	>4
<=0.5	8	4	<=0.5	<=32	1	<=0.25	>64	<=0.5
<=0.5	<=2	4	<=0.5	<=32	1	<=0.25	>64	>4
<=0.5	>16	>16	>32	<=32	>8	0.5	>64	<=0.5
<=0.5	4	4	2	<=32	2	<=0.25	>64	<=0.5
<=0.5	4	2	<=0.5	<=32	1	<=0.25	>64	>4
<=0.5	8	8	<=0.5	<=32	1	0.5	<=4	<=0.5
<=0.5	<=2	2	<=0.5	<=32	1	<=0.25	<=4	<=0.5
<=0.5	4	4	<=0.5	<=32	1	<=0.25	<=4	<=0.5
<=0.5	4	4	<=0.5	<=32	2	<=0.25	<=4	>4
<=0.5	>16	>16	8	<=32	<=0.5	<=0.25	>64	>4
<=0.5	<=2	4	<=0.5	<=32	<=0.5	<=0.25	>64	>4
<=0.5	4	8	<=0.5	<=32	1	<=0.25	<=4	<=0.5
<=0.5	4	4	<=0.5	<=32	<=0.5	<=0.25	<=4	<=0.5
<=0.5	8	4	<=0.5	<=32	<=0.5	<=0.25	>64	>4
<=0.5	4	4	<=0.5	<=32	<=0.5	<=0.25	>64	<=0.5
<=0.5	4	4	<=0.5	<=32	<=0.5	<=0.25	>64	>4
<=0.5	4	4	<=0.5	<=32	1	<=0.25	32	>2
<=0.5	8	8	<=0.5	<=32	<=0.5	<=0.25	>64	>2
<=0.5	8	16	<=0.5	<=32	2	0.5	>64	>2
<=0.5	>16	8	<=0.5	<=32	2	<=0.25	>64	<=0.5
<=0.5	4	8	<=0.5	<=32	<=0.5	<=0.25	8	<=0.5
<=0.5	4	8	<=0.5	<=32	1	<=0.25	>64	>2
<=0.5	<=2	4	<=0.5	<=32	1	<=0.25	<=4	<=0.5
1	>16	>16	16	<=32	>8	<=0.25	>64	>2
<=0.5	>16	>16	8	64	1	0.5	>64	>2
<=0.5	<=2	2	<=0.5	<=32	1	1	>64	>2
<=0.5	4	16	<=0.5	<=32	1	<=0.25	>64	<=0.5
<=0.5	8	8	<=0.5	<=32	<=0.5	<=0.25	64	<=0.5
<=0.5	16	16	<=0.5	<=32	>8	<=0.25	>64	<=0.5
<=0.5	4	4	<=0.5	<=32	2	<=0.25	<=4	<=0.5
<=0.5	4	4	<=0.5	<=32	>8	<=0.25	>64	>2
<=0.5	4	4	<=0.5	<=32	1	<=0.25	>64	>2
<=0.5	8	8	<=0.5	<=32	>8	<=0.25	>64	<=0.5
<=0.5	<=2	<=1	<=0.5	<=32	1	<=0.25	<=4	<=0.5
<=0.5	8	4	<=0.5	<=32	>8	<=0.25	>64	>2
<=0.5	4	4	<=0.5	<=32	2	<=0.25	<=4	<=0.5
<=0.5	4	4	<=0.5	<=32	<=0.5	<=0.25	>64	>2
<=0.5	16	4	<=0.5	<=32	>8	<=0.25	>64	<=0.5
<=0.5	8	8	<=0.5	<=32	2	<=0.25	>64	<=0.5
<=0.5	8	8	<=0.5	<=32	>8	<=0.25	>64	>2
<=0.5	4	8	<=0.5	<=32	1	<=0.25	>64	<=0.5

<=0.5	8	16	<=0.5	<=32	8	<=0.25	>64	>2
<=0.5	4	4	<=0.5	<=32	1	<=0.25	>64	<=0.5
4	<=2	>16	>32	<=32	<=0.5	<=0.25	>64	>2
<=0.5	8	8	<=0.5	<=32	<=0.5	<=0.25	>64	<=0.5
<=0.5	4	4	<=0.5	<=32	>8	<=0.25	>64	>2
<=0.5	8	8	<=0.5	64	1	<=0.25	>64	>2
<=0.5	<=2	2	<=0.5	<=32	1	<=0.25	<=4	<=0.5
<=0.5	<=2	8	<=0.5	<=32	<=0.5	<=0.25	>64	<=0.5
<=0.5	16	16	<=0.5	>64	<=0.5	<=0.25	<=4	<=0.5
<=0.5	4	8	<=0.5	<=32	1	<=0.25	>64	>2
<=0.5	4	4	<=0.5	<=32	>8	<=0.25	>64	>2
<=0.5	4	4	<=0.5	<=32	1	<=0.25	<=4	<=0.5
<=0.5	8	8	<=0.5	<=32	<=0.5	<=0.25	>64	<=0.5
<=0.5	4	8	<=0.5	<=32	>8	<=0.25	>64	<=0.5
<=0.5	8	8	<=0.5	<=32	2	<=0.25	<=4	<=0.5
<=0.5	4	4	<=0.5		<=0.5	<=0.25	>64	>4
<=0.5	>16	>16	16		>8	<=0.25	>64	>4
<=0.5	8	4	<=0.5		>8	<=0.25	>64	>4
>16?	>16	>16	>32		1	1	32	<=0.5
<=0.5	<=2	2	<=0.5		<=0.5	0.5	>64	<=0.5
<=0.5	<=2	4	<=0.5		1	<=0.25	<=4	>4
<=0.5	8	8	<=0.5		8	<=0.25	>64	>4
<=0.5	4	2	<=0.5		1	<=0.25	<=4	<=0.5
<=0.5	<=2	8	<=0.5		>8	<=0.25	>64	>4
2	<=2	>16	>32		2	1	>64	<=0.5
<=0.5	<=2	4	<=0.5		1	<=0.25	<=4	<=0.5
<=0.5	4	4	<=0.5		<=0.5	<=0.25	<=4	<=0.5
<=0.5	8	4	<=0.5		1	<=0.25	<=4	<=0.5
<=0.5	4	4	<=0.5		1	<=0.25	<=4	<=0.5
<=0.5	<=2	2	<=0.5		2	<=0.25	>64	<=0.5
<=0.5	4	8	<=0.5		1	<=0.25	>64	>4
<=0.5	<=2	4	<=0.5		1	<=0.25	<=4	<=0.5
<=0.5	4	4	<=0.5		1	<=0.25	<=4	<=0.5
<=0.5	<=2	4	<=0.5		1	<=0.25	<=4	<=0.5
<=0.5	4	2	<=0.5		1	0.5	<=4	<=0.5
<=0.5	8	8	<=0.5		1	<=0.25	>64	>4
<=0.5	<=2	4	<=0.5		1	<=0.25	<=4	<=0.5
16	8	>16	>32		<=0.5	<=0.25	>64	>4
<=0.5	<=2	4	<=0.5		1	<=0.25	<=4	<=0.5
4	8	>16	>32		>8	<=0.25	>64	>4
<=0.5	4	4	<=0.5		1	<=0.25	<=4	<=0.5
>16	16	>16	>32		<=0.5	0.5	>64	>4
<=0.5	8	4	<=0.5		2	0.5	>64	>4
<=0.5	8	8	<=0.5		>8	<=0.25	>64	>4
<=0.5	4	4	<=0.5		1	<=0.25	>64	<=0.5
<=0.5	8	4	<=0.5		2	<=0.25	<=4	>4
<=0.5	16	16	<=0.5		4	0.5	>64	<=0.5
8	16	>16	>32		2	0.5	>64	>4
<=0.5	>16	>16	8		>8	<=0.25	64	>4
<=0.5	>16	16	8		2	<=0.25	>64	>4
2	4	>16	>32		>8	<=0.25	>64	<=0.5
16	>16	>16	>32		1	1	>64	>4
<=0.5	4	4	<=0.5		1	<=0.25	<=4	<=0.5
<=0.5	4	4	<=0.5		1	<=0.25	<=4	>4
<=0.5	4	4	<=0.5		1	<=0.25	<=4	<=0.5
<=0.5	4	4	<=0.5		1	<=0.25	<=4	<=0.5
<=0.5	4	4	<=0.5		1	<=0.25	<=4	<=0.5
<=0.5	4	4	<=0.5		2	<=0.25	<=4	>4
<=0.5	<=2	4	<=0.5		<=0.5	<=0.25	>64	<=0.5
<=0.5	<=2	4	<=0.5		>8	<=0.25	>64	>4
<=0.5	4	2	<=0.5		<=0.5	<=0.25	<=4	<=0.5

<=0.5	8	8	<=0.5	<=0.5	<=0.25	>64	>4
<=0.5	<=2	2	<=0.5	1	<=0.25	>64	>4
<=0.5	4	8	<=0.5	<=0.5	<=0.25	<=4	<=0.5
<=0.5	4	4	<=0.5	1	<=0.25	<=4	<=0.5
4	>16	>16	>32	>8	<=0.25	>64	<=0.5
<=0.5	<=2	2	<=0.5	>8	0.5	>64	>4
<=0.5	<=2	4	<=0.5	<=0.5	<=0.25	64	>4
1	>16	>16	16	>8	0.5	>64	<=0.5
<=0.5	<=2	2	<=0.5	<=0.5	<=0.25	<=4	<=0.5
<=0.5	4	4	<=0.5	1	<=0.25	>64	>4
<=0.5	<=2	8	<=0.5	1	<=0.25	<=4	<=0.5
<=0.5	8	8	<=0.5	1	<=0.25	>64	<=0.5
<=0.5	>16	>16	2	>8	<=0.25	>64	>4
<=0.5	<=2	4	<=0.5	1	<=0.25	>64	>4
<=0.5	8	4	<=0.5	1	<=0.25	<=4	<=0.5
<=0.5	>16	>16	8	>8	0.5	>64	<=0.5
4	<=2	>16	32	>8	<=0.25	>64	>4
<=0.5	4	4	<=0.5	8	<=0.25	>64	>4
<=0.5	8	8	<=0.5	1	<=0.25	>64	<=0.5
<=0.5	4	4	<=0.5	>8	<=0.25	>64	>4
<=0.5	16	8	<=0.5	2	<=0.25	>64	>4
<=0.5	<=2	2	<=0.5	<=0.5	<=0.25	<=4	<=0.5
<=0.5	>16	>16	16	2	0.5	32	>4
<=0.5	8	16	<=0.5	>8	<=0.25	>64	4
<=0.5	<=2	4	<=0.5	<=0.5	<=0.25	<=4	>4
<=0.5	8	8	<=0.5	1	<=0.25	>64	>4
<=0.5	<=2	2	<=0.5	1	<=0.25	<=4	<=0.5
<=0.5	<=2	<=1	<=0.5	1	<=0.25	<=4	<=0.5
<=0.5	4	8	<=0.5	1	<=0.25	>64	<=0.5
<=0.5	8	8	<=0.5	8	<=0.25	>64	4
<=0.5	4	4	<=0.5	1	<=0.25	<=4	<=0.5
<=0.5	4	4	<=0.5	1	<=0.25	>64	<=0.5
<=0.5	4	8	<=0.5	1	<=0.25	>64	>4
<=0.5	8	4	<=0.5	>8	<=0.25	>64	>4
<=0.5	16	16	<=0.5	<=0.5	<=0.25	>64	>4
<=0.5	>16	>16	8	>8	<=0.25	>64	>4
<=0.5	<=2	4	<=0.5	<=0.5	<=0.25	>64	<=0.5
<=0.5	8	4	<=0.5	2	<=0.25	<=4	<=0.5
<=0.5	<=2	4	<=0.5	1	<=0.25	>64	<=0.5
<=0.5	<=2	8	<=0.5	1	<=0.25	>64	<=0.5
<=0.5	16	4	<=0.5	>8	<=0.25	>64	>4
<=0.5	4	2	<=0.5	1	<=0.25	>64	>4
<=0.5	4	8	<=0.5	<=0.5	<=0.25	<=4	<=0.5
<=0.5	16	8	2	<=0.5	0.5	64	>4
<=0.5	<=2	2	<=0.5	<=0.5	<=0.25	>64	<=0.5
2	>16	>16	>32	4	0.5	>64	>4
<=0.5	4	4	<=0.5	1	<=0.25	>64	<=0.5
2	>16	>16	>32	>8	0.5	>64	>4
>16	4	>16	>32	>8	<=0.25	>64	<=0.5
<=0.5	8	8	<=0.5	2	<=0.25	>64	>4
<=0.5	8	16	<=0.5	2	<=0.25	>64	>4
<=0.5	8	4	<=0.5	1	<=0.25	<=4	<=0.5
<=0.5	<=2	<=1	<=0.5	<=0.5	<=0.25	<=4	>4
<=0.5	4	4	<=0.5	>8	<=0.25	>64	>4
<=0.5	4	4	<=0.5	>8	<=0.25	>64	>4
<=0.5	4	4	<=0.5	1	<=0.25	<=4	<=0.5
<=0.5	<=2	2	<=0.5	>8	<=0.25	>64	<=0.5
<=0.5	<=2	2	<=0.5	<=0.5	<=0.25	<=4	<=0.5
<=0.5	<=2	4	<=0.5	<=0.5	<=0.25	<=4	<=0.5
<=0.5	4	4	<=0.5	1	<=0.25	>64	<=0.5
<=0.5	4	4	<=0.5	1	<=0.25	>64	>4

	<=0.5	4	4	<=0.5	1	<=0.25	>64	<=0.5
	<=0.5	>16	>16	32	4	<=0.25	32	<=0.5
	<=0.5	16	8	<=0.5	>8	<=0.25	64	>4
	<=0.5	<=2	2	<=0.5	1	<=0.25	<=4	<=0.5
	<=0.5	>16	16	<=0.5	1	<=0.25	<=4	<=0.5
	<=0.5	8	8	<=0.5	1	<=0.25	>64	<=0.5
	<=0.5	8	4	<=0.5	1	<=0.25	>64	<=0.5
<=0.25	>16	>16	>16	>32	>8	<=0.25	>64	<=0.25
	16	4	>16	>32	2	<=0.25	>64	<=0.5
	<=0.5	4	4	<=0.5	1	<=0.25	<=4	<=0.5
	<=0.5	16	16	<=0.5	>8	<=0.25	>64	>4
	<=0.5	8	8	<=0.5	1	<=0.25	<=4	<=0.5
	<=0.5	8	8	<=0.5	<=0.5	<=0.25	>64	>4
	<=0.5	4	4	<=0.5	1	<=0.25	<=4	<=0.5
	<=0.5	<=2	4	<=0.5	<=0.5	<=0.25	>64	>4
	<=0.5	8	4	<=0.5	1	<=0.25	>64	>4
	<=0.5	>16	>16	8	1	<=0.25	>64	>4
<=0.25	<=0.5	8	16	<=0.5	1	1	<=4	<=0.5
	<=0.5	16	>16	<=0.5	1	<=0.25	8	<=0.5
	<=0.5	>16	>16	8	>8	<=0.25	64	>4
	<=0.5	4	8	<=0.5	1	<=0.25	>64	>4
	<=0.5	<=2	2	<=0.5	1	<=0.25	64	>4
	<=0.5	<=2	2	<=0.5	<=0.5	<=0.25	>64	>4
	<=0.5	16	16	<=0.5	>8	<=0.25	>64	>4
	<=0.5	4	4	<=0.5	>8	0.5	>64	>4
	<=0.5	>16	>16	8	>8	<=0.25	>64	>4
	<=0.5	8	8	<=0.5	1	<=0.25	<=4	>4
	<=0.5	4	8	<=0.5	>8	<=0.25	>64	>4
	<=0.5	>16	>16	8	1	<=0.25	>64	>4
	<=0.5	<=2	2	<=0.5	<=0.5	<=0.25	>64	<=0.5
	<=0.5	<=2	4	<=0.5	1	<=0.25	>64	<=0.5
	4	>16	>16	>32	>8	0.5	>64	>4
	<=0.5	4	8	<=0.5	>8	<=0.25	>64	>4
	>16	>16	>16	>32	>8	0.5	>64	>4
	>16	8	>16	>32	>8	<=0.25	>64	>4
	>16	16	>16	>32	1	<=0.25	>64	>4
	<=0.5	8	8	<=0.5	1	<=0.25	>64	>4
	<=0.5	8	8	<=0.5	>8	<=0.25	>64	>4
	<=0.5	<=2	2	<=0.5	1	0.5	>64	>4
	<=0.5	4	4	<=0.5	2	<=0.25	>64	>4
	<=0.5	<=2	4	<=0.5	<=0.5	<=0.25	>64	<=0.5
	<=0.5	4	4	<=0.5	2	<=0.25	<=4	<=0.5
	<=0.5	>16	>16	16	4	<=0.25	>64	>4
	<=0.5	4	4	<=0.5	1	<=0.25	<=4	<=0.5
	<=0.5	8	8	<=0.5	>8	<=0.25	>64	>4
	<=0.5	4	4	<=0.5	1	<=0.25	>64	<=0.5
	<=0.5	8	8	<=0.5	1	<=0.25	<=4	<=0.5
	<=0.5	4	8	<=0.5	<=0.5	<=0.25	>64	>4
	<=0.5	>16	8	<=0.5	>8	<=0.25	>64	>4
	<=0.5	4	8	<=0.5	1	<=0.25	<=4	<=0.5
	<=0.5	>16	16	4	<=0.5	<=0.25	>64	>4
	<=0.5	<=2	4	<=0.5	2	<=0.25	>64	<=0.5
	<=0.5	<=2	4	<=0.5	1	<=0.25	>64	>4
	<=0.5	<=2	2	<=0.5	1	<=0.25	>64	<=0.5
	<=0.5	8	8	<=0.5	1	<=0.25	<=4	<=0.5
	<=0.5	<=2	8	<=0.5	2	<=0.25	64	<=0.5
	<=0.5	4	8	<=0.5	1	<=0.25	<=4	<=0.5
	<=0.5	4	2	<=0.5	<=0.5	<=0.25	<=4	<=0.5
	<=0.5	4	4	<=0.5	>8	<=0.25	>64	>4
	<=0.5	<=2	4	<=0.5	1	<=0.25	<=4	<=0.5
	<=0.5	<=2	4	<=0.5	<=0.5	<=0.25	>64	>4

	<=0.5	<=2	4	<=0.5	<=0.5	<=0.25	<=4	<=0.5
	<=0.5	16	16	<=0.5	>8	<=0.25	<=4	<=0.5
	<=0.5	4	4	<=0.5	<=0.5	<=0.25	<=4	<=0.5
	<=0.5	<=2	<=1	<=0.5	<=0.5	<=0.25	>64	>4
	<=0.5	<=2	4	<=0.5	<=0.5	<=0.25	>64	>4
	<=0.5	4	2	<=0.5	1	<=0.25	64	>4
	<=0.5	4	4	<=0.5	>8	<=0.25	>64	>4
	<=0.5	<=2	2	<=0.5	1	<=0.25	>64	>4
	<=0.5	<=2	2	<=0.5	1	<=0.25	>64	<=0.5
	<=0.5	8	8	<=0.5	>8	<=0.25	>64	>4
	<=0.5	8	4	<=0.5	>8	<=0.25	>64	>4
	4	8	>16	>32	<=0.5	<=0.25	>64	>4
	<=0.5	4	4	<=0.5	>8	<=0.25	<=4	>4
	<=0.5	4	8	<=0.5	>8	<=0.25	>64	>4
	<=0.5	8	4	<=0.5	2	<=0.25	<=4	>4
	<=0.5	4	4	<=0.5	<=0.5	<=0.25	<=4	<=0.5
	<=0.5	4	4	<=0.5	1	<=0.25	>64	<=0.5
	<=0.5	4	8	<=0.5	1	<=0.25	<=4	<=0.5
	<=0.5	<=2	<=1	<=0.5	<=0.5	<=0.25	<=4	<=0.5
	<=0.5	<=2	4	<=0.5	1	<=0.25	>64	>4
	<=0.5	<=2	4	<=0.5	1	<=0.25	<=4	<=0.5
	<=0.5	4	4	<=0.5	<=0.5	<=0.25	<=4	<=0.5
	>16	>16	>16	>32	>8	<=0.25	>64	>4
	<=0.5	4	2	<=0.5	<=0.5	<=0.25	<=4	<=0.5
	<=0.5	8	8	<=0.5	1	<=0.25	>64	>4
	<=0.5	8	16	<=0.5	1	<=0.25	<=4	<=0.5
	<=0.5	4	4	<=0.5	1	<=0.25	<=4	<=0.5
	<=0.5	4	4	<=0.5	<=0.5	<=0.25	<=4	<=0.5
	<=0.5	4	4	<=0.5	2	<=0.25	>64	>4
	<=0.5	<=2	2	<=0.5	<=0.5	<=0.25	<=4	<=0.5
	<=0.5	8	8	<=0.5	1	<=0.25	>64	>4
	<=0.5	<=2	4	<=0.5	<=0.5	<=0.25	>64	<=0.5
	<=0.5	4	8	<=0.5	>8	<=0.25	>64	>4
	<=0.5	>16	>16	4	>8	<=0.25	>64	>4
	<=0.5	4	4	<=0.5	<=0.5	<=0.25	>64	>4
	<=0.5	4	4	<=0.5	1	<=0.25	>64	<=0.5
	<=0.5	8	8	<=0.5	>8	<=0.25	>64	>4
	8	>16	>16	16	>8	1	>64	>4
	<=0.5	8	8	<=0.5	>8	<=0.25	>64	>4
	<=0.5	8	8	<=0.5	>8	<=0.25	>64	>4
	<=0.5	<=2	4	<=0.5	1	<=0.25	>64	>4
	<=0.5	8	8	<=0.5	>8	<=0.25	64	>4
	<=0.5	4	4	<=0.5	1	<=0.25	>64	>4
	<=0.5	8	4	<=0.5	1	<=0.25	<=4	<=0.5
4	4	>16	>16	>32	>8	2	>64	>4
	<=0.5	8	8	<=0.5	>8	<=0.25	>64	<=0.5
	<=0.5	>16	>16	4	>8	<=0.25	64	>4
	<=0.5	4	4	<=0.5	1	<=0.25	>64	<=0.5
	<=0.5	>16	>16	8	1	0.5	>64	>4
	<=0.5	4	8	<=0.5	>8	0.5	>64	>4
	<=0.5	4	2	<=0.5	<=0.5	<=0.25	>64	>4
	>16	>16	>16	>32	>8	<=0.25	>64	>4
	<=0.5	4	2	<=0.5	1	<=0.25	>64	<=0.5
	<=0.5	4	2	<=0.5	>8	<=0.25	>64	>4
	<=0.5	<=2	4	<=0.5	2	<=0.25	>64	<=0.5
	<=0.5	4	8	<=0.5	1	<=0.25	<=4	<=0.5
	<=0.5	8	8	<=0.5	<=0.5	<=0.25	<=4	2
	<=0.5	8	8	<=0.5	1	<=0.25	>64	>4
	<=0.5	8	4	<=0.5	<=0.5	<=0.25	<=4	<=0.5
	<=0.5	<=2	4	<=0.5	1	<=0.25	>64	>4
	<=0.5	8	8	<=0.5	<=0.5	<=0.25	>64	>4

	<=0.5	4	4	<=0.5	1	1	>64	<=0.5
	<=0.5	4	8	<=0.5	1	<=0.25	>64	>4
4	2	>16	>16	>32	>8	1	64	>4
	<=0.5	8	4	<=0.5	>8	<=0.25	>64	>4
	<=0.5	8	4	<=0.5	1	<=0.25	<=4	<=0.5
	<=0.5	>16	>16	<=0.5	>8	0.5	>64	>4
	<=0.5	8	4	<=0.5	1	<=0.25	<=4	<=0.5
	<=0.5	<=2	2	<=0.5	<=0.5	<=0.25	>64	<=0.5
	<=0.5	4	4	<=0.5	<=0.5	<=0.25	>64	>4
	<=0.5	8	4	<=0.5	<=0.5	<=0.25	>64	<=0.5
	<=0.5	>16	8	<=0.5	2	<=0.25	>64	>4
	<=0.5	8	4	<=0.5	4	<=0.25	>64	>4
	2	>16	>16	32	>8	<=0.25	>64	>4
	4	>16	>16	>32	>8	0.5	>64	>4
	<=0.5	8	4	<=0.5	1	<=0.25	>64	<=0.5
	1	>16	>16	>32	1	0.5	>64	>4
	<=0.5	>16	>16	32	>8	0.5	>64	>4
	<=0.5	4	2	<=0.5	1	<=0.25	<=4	<=0.5
	<=0.5	4	4	<=0.5	<=0.5	<=0.25	<=4	<=0.5
	<=0.5	4	4	<=0.5	<=0.5	<=0.25	>64	>4
	>16	16	>16	>32	>8	<=0.25	>64	>4
	<=0.5	>16	>16	16	>8	<=0.25	>64	>4
	<=0.5	4	4	<=0.5	>8	<=0.25	>64	>4
	<=0.5	<=2	4	<=0.5	1	<=0.25	<=4	<=0.5
	<=0.5	4	4	<=0.5	<=0.5	<=0.25	<=4	<=0.5
	<=0.5	8	8	<=0.5	2	<=0.25	>64	<=0.5
	<=0.5	8	4	<=0.5	>8	<=0.25	>64	<=0.5
	<=0.5	8	8	<=0.5	1	<=0.25	<=4	<=0.5
	<=0.5	<=2	8	<=0.5	>8	<=0.25	>64	<=0.5
	<=0.5	8	4	<=0.5	<=0.5	<=0.25	>64	>4
	<=0.5	4	4	<=0.5	>8	<=0.25	>64	>4
	<=0.5	4	4	<=0.5	1	<=0.25	<=4	<=0.5
	<=0.5	4	4	<=0.5	>8	<=0.25	>64	>4
	<=0.5	<=2	2	<=0.5	<=0.5	<=0.25	<=4	<=0.5
	<=0.5	8	8	<=0.5	<=0.5	<=0.25	>64	<=0.5
	16	8	>16	>32	1	0.5	>64	<=0.5
	<=0.5	<=2	4	<=0.5	1	<=0.25	>64	<=0.5
	<=0.5	<=2	8	<=0.5	<=0.5	0.5	<=4	<=0.5
	<=0.5	4	4	<=0.5	<=0.5	<=0.25	>64	<=0.5
	<=0.5	4	8	<=0.5	<=0.5	<=0.25	<=4	<=0.5
	<=0.5	4	4	<=0.5	1	<=0.25	<=4	<=0.5
	<=0.5	<=2	4	<=0.5	1	<=0.25	>64	>4
	<=0.5	<=2	4	<=0.5	<=0.5	<=0.25	>64	<=0.5
	<=0.5	4	4	<=0.5	1	<=0.25	<=4	<=0.5
	<=0.5	<=2	4	<=0.5	1	<=0.25	>64	<=0.5
	<=0.5	<=2	8	<=0.5	>8	<=0.25	>64	>4
	<=0.5	8	8	<=0.5	>8	<=0.25	>64	>4
	<=0.5	4	4	<=0.5	<=0.5	<=0.25	64	>4
	2	>16	>16	>32	<=0.5	0.5	>64	>4
	<=0.5	4	2	<=0.5	<=0.5	<=0.25	>64	<=0.5
	<=0.5	<=2	2	<=0.5	>8	<=0.25	<=4	>4
	<=0.5	<=2	2	<=0.5	2	<=0.25	>64	>4
	<=0.5	4	4	<=0.5	<=0.5	<=0.25	<=4	<=0.5
	<=0.5	4	4	<=0.5	<=0.5	<=0.25	>64	>4
	<=0.5	4	4	<=0.5	1	<=0.25	<=4	<=0.5
	<=0.5	8	8	<=0.5	1	<=0.25	>64	4
	<=0.5	4	4	<=0.5	<=0.5	<=0.25	<=4	<=0.5
	<=0.5	16	16	<=0.5	>8	<=0.25	>64	>4
	<=0.5	<=2	2	<=0.5	1	<=0.25	<=4	<=0.5
	<=0.5	8	8	<=0.5	1	<=0.25	>64	>4
	<=0.5	>16	>16	2	<=0.5	<=0.25	64	<=0.5

<=0.5	4	2	<=0.5	1	<=0.25	>64	<=0.5
<=0.5	4	4	<=0.5	<=0.5	<=0.25	<=4	<=0.5
<=0.5	4	4	<=0.5	>8	<=0.25	>64	>4
<=0.5	<=2	<=1	<=0.5	>8	<=0.25	>64	2
<=0.5	<=2	4	<=0.5	<=0.5	<=0.25	>64	>4
<=0.5	<=2	4	<=0.5	<=0.5	<=0.25	>64	<=0.5
<=0.5	<=2	4	<=0.5	1	<=0.25	64	<=0.5
<=0.5	<=2	2	<=0.5	1	<=0.25	<=4	<=0.5
<=0.5	4	4	<=0.5	<=0.5	<=0.25	>64	>4
<=0.5	4	4	<=0.5	1	<=0.25	>64	>4
<=0.5	<=2	4	<=0.5	2	<=0.25	<=4	<=0.5
<=0.5	4	2	<=0.5	>8	<=0.25	>64	<=0.5
<=0.5	<=2	2	<=0.5	<=0.5	<=0.25	>64	>4
<=0.5	4	4	<=0.5	1	<=0.25	<=4	<=0.5
<=0.5	4	4	<=0.5	2	<=0.25	<=4	<=0.5
<=0.5	4	4	<=0.5	2	<=0.25	>64	<=0.5
<=0.5	4	4	<=0.5	1	<=0.25	>64	>4
<=0.5	4	4	<=0.5	<=0.5	<=0.25	>64	>4
<=0.5	4	4	<=0.5	<=0.5	<=0.25	>64	<=0.5
1	>16	>16	32	>8	<=0.25	>64	>4
<=0.5	4	4	<=0.5	<=0.5	<=0.25	<=4	<=0.5
<=0.5	8	8	<=0.5	1	<=0.25	>64	>4
<=0.5	4	4	<=0.5	2	<=0.25	>64	>4
<=0.5	8	4	<=0.5	<=0.5	<=0.25	<=4	<=0.5
<=0.5	<=2	2	<=0.5	1	<=0.25	<=4	<=0.5
<=0.5	4	4	<=0.5	<=0.5	<=0.25	64	>4
<=0.5	<=2	2	<=0.5	<=0.5	<=0.25	>64	<=0.5
<=0.5	4	2	<=0.5	>8	<=0.25	>64	>4
>16	>16	>16	>32	>8	0.5	>64	>4
<=0.5	4	2	<=0.5	<=0.5	<=0.25	<=4	<=0.5
<=0.5	8	4	<=0.5	1	<=0.25	>64	>4
<=0.5	4	4	<=0.5	>8	<=0.25	>64	<=0.5
<=0.5	8	4	<=0.5	>8	<=0.25	>64	>4
<=0.5	<=2	4	<=0.5	1	<=0.25	>64	>4
4	>16	>16	>32	>8	0.5	>64	>4
<=0.5	8	4	<=0.5	8	<=0.25	64	>4
<=0.5	4	4	<=0.5	<=0.5	<=0.25	>64	<=0.5
<=0.5	<=2	8	<=0.5	1	<=0.25	<=4	<=0.5
>16	8	>16	>32	>8	<=0.25	>64	>4
<=0.5	<=2	4	<=0.5	>8	0.5	>64	>4
<=0.5	8	16	<=0.5	2	0.5	>64	>4
<=0.5	4	8	<=0.5	<=0.5	<=0.25	>64	>4
1	<=2	8	<=0.5	1	<=0.25	64	>4
<=0.5	4	4	<=0.5	>8	<=0.25	>64	>4
<=0.5	4	2	<=0.5	1	<=0.25	>64	>4
<=0.5	4	2	<=0.5	1	<=0.25	<=4	<=0.5
<=0.5	<=2	2	<=0.5	2	<=0.25	>64	>4
<=0.5	4	2	<=0.5	1	<=0.25	>64	>4
<=0.5	8	4	<=0.5	<=0.5	<=0.25	<=4	<=0.5
<=0.5	<=2	4	<=0.5	<=0.5	<=0.25	>64	<=0.5
<=0.5	4	4	<=0.5	>8	<=0.25	>64	>4
<=0.5	8	8	<=0.5	2	<=0.25	64	>4
<=0.5	4	4	<=0.5	1	<=0.25	<=4	<=0.5
<=0.5	<=2	2	<=0.5	<=0.5	<=0.25	<=4	<=0.5
<=0.5	8	4	<=0.5	>8	<=0.25	>64	>4
<=0.5	8	8	<=0.5	>8	<=0.25	>64	<=0.5
<=0.5	4	4	<=0.5	1	<=0.25	>64	<=0.5
<=0.5	>16	>16	16	1	<=0.25	>64	>4
<=0.5	4	4	<=0.5	>8	<=0.25	>64	>4
<=0.5	<=2	2	<=0.5	1	<=0.25	>64	<=0.5
<=0.5	8	8	<=0.5	<=0.5	<=0.25	>64	>4

>16	>16	>16	>32	>8	<=0.25	>64	>4
4	>16	>16	>32	>8	0.5	>64	>4
<=0.5	8	8	<=0.5	>8	<=0.25	>64	>4
<=0.5	4	4	<=0.5	2	<=0.25	>64	<=0.5
<=0.5	4	4	<=0.5	<=0.5	<=0.25	64	>4
<=0.5	<=2	2	<=0.5	>8	<=0.25	>64	>4
<=0.5	4	4	<=0.5	1	<=0.25	64	>4
<=0.5	8	4	<=0.5	1	<=0.25	<=4	<=0.5
<=0.5	<=2	4	<=0.5	>8	<=0.25	>64	>4
<=0.5	<=2	4	<=0.5	<=0.5	<=0.25	<=4	<=0.5
<=0.5	<=2	4	<=0.5	1	<=0.25	>64	<=0.5
<=0.5	<=2	2	<=0.5	1	<=0.25	>64	<=0.5
8	8	>16	>32	1	<=0.25	>64	>4
<=0.5	8	16	1	<=0.5	<=0.25	16	<=0.5
<=0.5	<=2	2	<=0.5	1	<=0.25	<=4	<=0.5
<=0.5	4	4	<=0.5	>8	<=0.25	>64	>4
<=0.5	4	<=1	<=0.5	1	<=0.25	64	<=0.5
<=0.5	8	8	<=0.5	2	<=0.25	>64	<=0.5
<=0.5	4	4	<=0.5	<=0.5	<=0.25	<=4	>4
<=0.5	4	4	<=0.5	>8	<=0.25	>64	>4
<=0.5	<=2	2	<=0.5	2	<=0.25	<=4	<=0.5
<=0.5	>16	>16	8	>8	<=0.25	>64	>4
<=0.5	<=2	<=1	<=0.5	<=0.5	<=0.25	>64	<=0.5
1	>16	>16	16	>8	<=0.25	>64	>4
<=0.5	4	4	<=0.5	<=0.5	<=0.25	<=4	<=0.5
<=0.5	4	4	<=0.5	1	<=0.25	32	>4
<=0.5	16	16	<=0.5	2	<=0.25	8	<=0.5
<=0.5	4	2	<=0.5	1	<=0.25	>64	>4
<=0.5	4	8	<=0.5	<=0.5	<=0.25	<=4	<=0.5
<=0.5	8	8	<=0.5	>8	<=0.25	>64	>4
<=0.5	4	2	<=0.5	<=0.5	<=0.25	<=4	<=0.5
<=0.5	<=2	4	<=0.5	1	<=0.25	<=4	<=0.5
<=0.5	4	2	<=0.5	1	<=0.25	>64	<=0.5
8	>16	>16	>32	>8	<=0.25	>64	>4
<=0.5	4	4	<=0.5	<=0.5	<=0.25	<=4	<=0.5
8	>16	>16	>32	>8	<=0.25	>64	>4
<=0.5	<=2	2	<=0.5	<=0.5	<=0.25	64	>4
<=0.5	<=2	2	<=0.5	1	<=0.25	<=4	<=0.5
<=0.5	>16	>16	16	>8	0.5	32	1
<=0.5	<=2	4	<=0.5	1	<=0.25	>64	<=0.5
<=0.5	4	4	<=0.5	1	<=0.25	<=4	<=0.5
<=0.5	4	8	<=0.5	1	<=0.25	<=4	<=0.5
<=0.5	<=2	2	<=0.5	<=0.5	<=0.25	<=4	<=0.5
<=0.5	8	2	<=0.5	<=0.5	<=0.25	>64	>4
<=0.5	8	4	<=0.5	<=0.5	<=0.25	>64	<=0.5
<=0.5	4	2	<=0.5	<=0.5	<=0.25	>64	>4
<=0.5	4	4	<=0.5	<=0.5	<=0.25	64	>4
<=0.5	<=2	4	<=0.5	1	<=0.25	<=4	<=0.5
<=0.5	<=2	2	<=0.5	<=0.5	0.5	>64	>4
1	4	2	<=0.5	1	<=0.25	<=4	<=0.5
<=0.5	<=2	4	<=0.5	1	<=0.25	>64	>4
16	4	>16	>32	<=0.5	<=0.25	>64	>4
<=0.5	4	4	<=0.5	1	<=0.25	>64	>4
<=0.5	<=2	2	<=0.5	<=0.5	<=0.25	>64	>4
<=0.5	>16	16	1	>8	<=0.25	>64	>4
<=0.5	>16	>16	>32	>8	<=0.25	64	>4
<=0.5	4	4	<=0.5	1	<=0.25	<=4	<=0.5
<=0.5	<=2	2	<=0.5	<=0.5	<=0.25	<=4	<=0.5
<=0.5	<=2	4	<=0.5	2	<=0.25	<=4	>4
<=0.5	4	4	<=0.5	>8	<=0.25	>64	<=0.5
<=0.5	16	16	<=0.5	1	<=0.25	<=4	<=0.5

<=0.5	4	4	<=0.5	2	<=0.25	>64	<=0.5
<=0.5	4	4	<=0.5	<=0.5	<=0.25	>64	>4
<=0.5	<=2	4	<=0.5	<=0.5	<=0.25	>64	>4
<=0.5	<=2	4	<=0.5	1	<=0.25	<=4	2
<=0.5	4	8	<=0.5	1	0.5	<=4	<=0.5
<=0.5	<=2	2	<=0.5	>8	<=0.25	>64	<=0.5
<=0.5	<=2	4	<=0.5	<=0.5	<=0.25	>64	>4
<=0.5	8	4	<=0.5	2	<=0.25	>64	>4
<=0.5	4	4	<=0.5	1	<=0.25	<=4	<=0.5
<=0.5	>16	>16	8	1	1	64	1
>16	8	>16	>32	1	<=0.25	>64	2
<=0.5	<=2	2	<=0.5	1	<=0.25	<=4	<=0.5
<=0.5	<=2	4	<=0.5	1	<=0.25	>64	<=0.5
<=0.5	8	8	<=0.5	1	<=0.25	>64	>4
<=0.5	<=2	4	<=0.5	1	<=0.25	>64	<=0.5
<=0.5	>16	>16	32	>8	<=0.25	>64	>4
<=0.5	4	2	<=0.5	1	<=0.25	<=4	<=0.5
<=0.5	4	4	<=0.5	1	<=0.25	>64	>4
<=0.5	<=2	8	<=0.5	1	<=0.25	>64	>4
<=0.5	4	8	<=0.5	1	<=0.25	<=4	>4
<=0.5	4	4	<=0.5	<=0.5	<=0.25	<=4	<=0.5
<=0.5	8	4	<=0.5	<=0.5	<=0.25	<=4	<=0.5
<=0.5	<=2	2	<=0.5	<=0.5	<=0.25	<=4	<=0.5
<=0.5	4	4	<=0.5	1	<=0.25	>64	>4
<=0.5	<=2	2	<=0.5	1	<=0.25	<=4	<=0.5
<=0.5	<=2	4	<=0.5	<=0.5	<=0.25	<=4	2
<=0.5	>16	>16	8	2	<=0.25	>64	>4
<=0.5	>16	8	<=0.5	1	<=0.25	<=4	<=0.5
>16	16	>16	>32	>8	<=0.25	>64	>4
<=0.5	4	4	<=0.5	>8	<=0.25	>64	>4
<=0.5	<=2	4	<=0.5	<=0.5	<=0.25	>64	>4
<=0.5	>16	>16	16	>8	<=0.25	>64	>4
<=0.5	<=2	2	<=0.5	<=0.5	<=0.25	<=4	<=0.5
<=0.5	4	2	<=0.5	1	<=0.25	>64	>4
<=0.5	<=2	4	<=0.5	<=0.5	<=0.25	>64	<=0.5
<=0.5	<=2	2	<=0.5	1	<=0.25	>64	>4
<=0.5	8	>16	<=0.5	<=0.5	<=0.25	>64	<=0.5
<=0.5	>16	>16	4	>8	<=0.25	>64	>4
<=0.5	4	4	<=0.5	<=0.5	<=0.25	>64	<=0.5
<=0.5	4	8	<=0.5	1	<=0.25	>64	>4
<=0.5	4	4	<=0.5	2	<=0.25	>64	<=0.5
<=0.5	>16	>16	32	>8	<=0.25	>64	>4
<=0.5	4	4	<=0.5	1	<=0.25	>64	>4
<=0.5	>16	>16	8	>8	0.5	>64	>4
<=0.5	4	4	<=0.5	<=0.5	<=0.25	<=4	<=0.5
<=0.5	4	4	<=0.5	<=0.5	<=0.25	<=4	>4
<=0.5	<=2	2	<=0.5	<=0.5	<=0.25	>64	<=0.5
<=0.5	<=2	2	<=0.5	<=0.5	<=0.25	<=4	<=0.5
<=0.5	4	4	<=0.5	<=0.5	<=0.25	64	>4
<=0.5	4	4	<=0.5	1	<=0.25	<=4	<=0.5
<=0.5	<=2	<=1	<=0.5	1	<=0.25	<=4	<=0.5
<=0.5	<=2	4	<=0.5	<=0.5	<=0.25	>64	<=0.5
<=0.5	4	4	<=0.5	1	<=0.25	>64	<=0.5
<=0.5	<=2	2	<=0.5	<=0.5	<=0.25	<=4	<=0.5
<=0.5	<=2	2	<=0.5	1	<=0.25	<=4	<=0.5
<=0.5	4	4	<=0.5	2	<=0.25	>64	>4
<=0.5	4	4	<=0.5	1	<=0.25	>64	>4
<=0.5	<=2	4	<=0.5	<=0.5	<=0.25	>64	>4
<=0.5	4	2	<=0.5	1	<=0.25	>64	<=0.5
<=0.5	<=2	4	<=0.5	1	<=0.25	<=4	<=0.5
<=0.5	8	4	<=0.5	1	<=0.25	<=4	<=0.5

16		>16	>16	>32		>8	<=0.25	>64	>4
<=0.5		<=2	4	<=0.5		1	<=0.25	>64	<=0.5
<=0.5		4	4	<=0.5		1	<=0.25	<=4	>4
<=0.5		4	4	<=0.5		1	<=0.25	<=4	<=0.5
<=0.5		8	4	<=0.5		<=0.5	<=0.25	>64	>4
<=0.5		4	4	<=0.5		1	<=0.25	>64	<=0.5
<=0.5		4	4	<=0.5		1	<=0.25	>64	<=0.5
<=0.5		4	4	<=0.5		>8	<=0.25	>64	>4
<=0.5		4	4	<=0.5		1	<=0.25	>64	>4
<=0.5		<=2	4	<=0.5		<=0.5	<=0.25	<=4	<=0.5
<=0.5		<=2	2	<=0.5		1	<=0.25	<=4	>4
<=0.5		4	8	<=0.5		1	<=0.25	>64	<=0.5
<=0.5		8	8	<=0.5		>8	<=0.25	>64	>4
<=0.5		8	8	<=0.5		1	<=0.25	>64	>4
<=0.5		4	8	<=0.5		1	<=0.25	>64	>4
<=0.5		4	4	<=0.5		<=0.5	<=0.25	<=4	>4
2		>16	>16	>32		1	0.5	>64	>4
<=0.5		4	4	<=0.5		1	<=0.25	>64	<=0.5
<=0.5		>16	>16	32		>8	<=0.25	>64	>4
<=0.5		4	4	<=0.5		1	<=0.25	>64	>4
<=0.5		<=2	2	<=0.5		1	<=0.25	>64	<=0.5
<=0.5		<=2	4	<=0.5		<=0.5	<=0.25	<=4	<=0.5
<=0.5		<=2	4	<=0.5		<=0.5	<=0.25	<=4	<=0.5
<=0.5		8	8	<=0.5		>8	<=0.25	>64	>4
<=0.5		<=2	2	<=0.5		<=0.5	<=0.25	>64	>4
<=0.5		<=2	2	<=0.5		<=0.5	<=0.25	>64	>4
<=0.5		<=2	2	<=0.5		<=0.5	<=0.25	>64	>4
<=0.5		4	4	<=0.5		1	<=0.25	>64	>4
<=0.5		<=2	4	<=0.5		>8	<=0.25	<=4	>4
<=0.5		<=2	4	<=0.5		>8	<=0.25	>64	>4
<=0.5		4	4	<=0.5		1	<=0.25	>64	<=0.5
<=0.5		<=2	4	<=0.5		<=0.5	<=0.25	<=4	<=0.5
<=0.5		4	8	<=0.5		>8	<=0.25	>64	>4
<=0.5		>16	>16	8		>8	<=0.25	>64	>4
<=0.5		4	4	<=0.5		<=0.5	<=0.25	<=4	<=0.5
<=0.5		4	4	<=0.5		1	<=0.25	>64	>4
<=0.5		4	8	<=0.5		>8	<=0.25	>64	>4
<=0.5		4	4	<=0.5		>8	<=0.25	>64	<=0.5
<=0.5		>16	>16	8		<=0.5	<=0.25	>64	<=0.5
<=0.5		8	8	<=0.5		8	<=0.25	>64	>4
<=0.5		4	4	<=0.5		1	<=0.25	>64	>4
<=0.5		4	2	<=0.5		1	<=0.25	>64	>4
<=0.5		<=2	2	<=0.5		1	<=0.25	>64	<=0.5
<=0.5		<=2	4	<=0.5		1	<=0.25	>64	>4
<=0.5		4	4	<=0.5		1	<=0.25	64	>4
<=0.5		8	4	<=0.5		1	<=0.25	>64	>4
<=0.5		4	4	<=0.5		1	<=0.25	>64	<=0.5
<=0.5		<=2	4	<=0.5		>8	<=0.25	>64	>4
<=0.5		16	8	<=0.5		>8	<=0.25	>64	<=0.5
<=0.5		8	4	<=0.5		1	<=0.25	<=4	<=0.5
<=0.5		4	4	<=0.5		>8	<=0.25	<=4	>4
<=0.5		4	4	<=0.5		1	<=0.25	>64	<=0.5
<=0.5		8	8	<=0.5		1	0.5	>64	<=0.5
<=0.5		4	4	<=0.5		1	<=0.25	<=4	<=0.5
<=0.5		4	4	<=0.5		1	<=0.25	32	>4
<=0.5		4	8	<=0.5		1	<=0.25	<=4	<=0.5
<=0.5		8	8	<=0.5		>8	<=0.25	>64	<=0.5
<=0.5		4	8	<=0.5		>8	<=0.25	>64	>4
<=0.5		4	4	<=0.5		1	<=0.25	>64	>4
<=0.5		4	4	<=0.5		1	<=0.25	<=4	<=0.5
2		>16	>16	16		>8	0.5	>64	>4

	<=0.5	8	4	<=0.5	1	<=0.25	>64	>4
	<=0.5	4	2	<=0.5	>8	<=0.25	>64	>4
	<=0.5	<=2	2	<=0.5	1	1	>64	>4
	<=0.5	4	4	<=0.5	1	<=0.25	<=4	<=0.5
	<=0.5	<=2	4	<=0.5	2	<=0.25	<=4	<=0.5
	<=0.5	>16	>16	8	<=0.5	<=0.25	>64	<=0.5
	<=0.5	4	4	<=0.5	1	<=0.25	>64	>4
	<=0.5	4	4	<=0.5	<=0.5	<=0.25	>64	>4
	>16	16	>16	>32	>8	<=0.25	>64	>4
	1	>16	>16	32	>8	0.5	>64	>4
	<=0.5	<=2	2	<=0.5	<=0.5	<=0.25	>64	>4
	<=0.5	4	8	<=0.5	1	<=0.25	>64	>4
	<=0.5	4	8	<=0.5	1	<=0.25	>64	<=0.5
	<=0.5	>16	>16	8	2	0.5	64	<=0.5
0.5	>16	>16	>16	>32	>8	2	>64	>4
	>16	16	>16	>32	1	<=0.25	>64	<=0.5
	<=0.5	4	4	<=0.5	1	<=0.25	<=4	<=0.5
	<=0.5	<=2	2	<=0.5	1	<=0.25	>64	<=0.5
	4	>16	>16	32	>8	<=0.25	>64	>4
	<=0.5	4	4	<=0.5	8	<=0.25	>64	<=0.5
	<=0.5	16	8	<=0.5	1	<=0.25	<=4	<=0.5
	<=0.5	4	8	<=0.5	1	<=0.25	>64	>4
	<=0.5	16	4	<=0.5	>8	<=0.25	>64	>4
	<=0.5	>16	>16	4	>8	<=0.25	>64	<=0.5
0.5	<=0.5	>16	>16	16	8	1	>64	>4
	<=0.5	8	16	<=0.5	>8	<=0.25	>64	>4
	<=0.5	<=2	2	<=0.5	1	<=0.25	>64	>4
	<=0.5	4	2	<=0.5	1	<=0.25	<=4	<=0.5
	<=0.5	4	8	<=0.5	2	<=0.25	>64	<=0.5
	<=0.5	4	8	<=0.5	1	<=0.25	>64	<=0.5
	<=0.5	<=2	2	<=0.5	>8	<=0.25	>64	>4
	<=0.5	4	2	<=0.5	1	<=0.25	>64	<=0.5
	<=0.5	8	4	<=0.5	1	<=0.25	<=4	<=0.5
	>16	>16	>16	>32	>8	0.5	>64	>4
	<=0.5	8	8	<=0.5	1	<=0.25	>64	<=0.5
	<=0.5	<=2	2	<=0.5	<=0.5	<=0.25	<=4	<=0.5
	<=0.5	4	4	<=0.5	<=0.5	<=0.25	>64	<=0.5
	<=0.5	4	8	<=0.5	1	<=0.25	<=4	<=0.5
	<=0.5	4	4	<=0.5	>8	<=0.25	>64	>4
	<=0.5	4	4	<=0.5	>8	<=0.25	>64	>4
	<=0.5	8	8	<=0.5	>8	<=0.25	>64	>4
	<=0.5	<=2	4	<=0.5	<=0.5	<=0.25	<=4	<=0.5
	<=0.5	4	8	<=0.5	2	<=0.25	<=4	<=0.5
	<=0.5	8	4	<=0.5	1	<=0.25	>64	>4
	<=0.5	<=2	4	<=0.5	1	<=0.25	<=4	<=0.5
	<=0.5	4	4	<=0.5	<=0.5	<=0.25	<=4	<=0.5
	<=0.5	8	8	<=0.5	<=0.5	<=0.25	>64	<=0.5
	<=0.5	4	8	<=0.5	1	<=0.25	<=4	<=0.5
	<=0.5	4	4	<=0.5	1	<=0.25	<=4	<=0.5
	<=0.5	4	8	<=0.5	>8	<=0.25	>64	<=0.5
	<=0.5	8	4	<=0.5	2	<=0.25	<=4	<=0.5
	<=0.5	<=2	4	<=0.5	1	<=0.25	>64	>4
	<=0.5	4	4	<=0.5	1	<=0.25	<=4	<=0.5
	1	>16	>16	32	<=0.5	0.5	>64	>4
	<=0.5	4	8	<=0.5	<=0.5	<=0.25	<=4	<=0.5
	<=0.5	8	16	<=0.5	1	<=0.25	>64	>4
	<=0.5	4	4	<=0.5	1	<=0.25	<=4	<=0.5
	<=0.5	8	8	<=0.5	1	0.5	>64	<=0.5
	<=0.5	<=2	2	<=0.5	1	<=0.25	<=4	<=0.5
	<=0.5	4	4	<=0.5	1	<=0.25	<=4	<=0.5
	16	4	>16	>32	>8	<=0.25	>64	<=0.5

<=0.5	<=2	2	<=0.5	1	<=0.25	>64	>4
<=0.5	>16	>16	32	>8	<=0.25	>64	>4
<=0.5	4	8	<=0.5	<=0.5	<=0.25	>64	>4
<=0.5	8	8	<=0.5	2	<=0.25	<=4	<=0.5
<=0.5	<=2	<=1	<=0.5	1	<=0.25	<=4	<=0.5
<=0.5	>16	>16	32	>8	<=0.25	>64	<=0.5
<=0.5	8	8	<=0.5	1	<=0.25	>64	>4
<=0.5	4	4	<=0.5	1	<=0.25	>64	<=0.5
>16	16	>16	>32	>8	<=0.25	>64	<=0.5
<=0.5	8	4	<=0.5	1	<=0.25	>64	<=0.5
<=0.5	4	4	<=0.5	8	<=0.25	<=4	>4
<=0.5	4	8	<=0.5	1	<=0.25	<=4	<=0.5
<=0.5	8	8	<=0.5	4	<=0.25	<=4	<=0.5
<=0.5	4	4	<=0.5	2	<=0.25	>64	<=0.5
<=0.5	<=2	2	<=0.5	<=0.5	<=0.25	8	>4
<=0.5	<=2	4	<=0.5	1	<=0.25	64	>4
<=0.5	8	8	<=0.5	1	<=0.25	>64	>4
<=1	<=4	4	<=1	<=1	<=0.25		<=0.5
<=1	<=4	4	<=1	>8	<=0.25		>2
>16	16	>16	>32	2	<=0.25		<=0.5
<=1	<=4	4	<=1	<=1	<=0.25		<=0.5
<=1	<=4	4	<=1	<=1	<=0.25		<=0.5
<=1	<=4	4	<=1	2	<=0.25		<=0.5
<=1	<=4	8	<=1	<=1	<=0.25		>2
<=1	>16	>16	8	>8	<=0.25		>2
<=1	<=4	4	<=1	<=1	<=0.25		<=0.5
<=1	<=4	4	<=1	<=1	<=0.25		>2
16	<=4	>16	>32	<=1	<=0.25		>2
<=1	>16	8	<=1	<=1	0.5		>2
<=1	>16	>16	16	4	<=0.25		>2
<=1	8	8	<=1	<=1	<=0.25		>2
<=1	8	8	<=1	2	<=0.25		<=0.5
<=1	<=4	4	<=1	<=1	<=0.25		<=0.5
<=1	8	8	<=1	>8	<=0.25		>2
<=1	<=4	8	<=1	<=1	<=0.5	>64	>2
<=1	<=4	4	<=1	<=1	<=0.5	>64	>2
<=1	<=4	8	<=1	<=1	<=0.5	>64	>2
<=1	<=4	8	<=1	<=1	<=0.5	>64	>2
<=1	<=4	4	<=1	<=1	<=0.5	64	<=0.5
<=1	<=4	4	<=1	<=1	<=0.5	>64	>2
<=1	>16	>16	8	>8	<=0.5	>64	>4
<=1	<=4	8	<=1	4	<=0.5	<=4	<=0.5
<=1	<=4	8	<=1	<=1	<=0.5	<=4	<=0.5
<=1	<=4	4	<=1	<=1	<=0.5	<=4	<=0.5
<=1	<=4	4	<=1	<=1	<=0.5	<=4	1
>16	8	>16	>32	4	<=0.5	>64	>4
<=1	<=4	8	<=1	<=1	<=0.5	>64	<=0.5
<=1	<=4	<=2	<=1	<=1	<=0.25		<=0.5
<=1	<=4	4	<=1	<=1	<=0.25		<=0.5
<=1	<=4	4	<=1	>8	<=0.25		>2
<=1	<=4	8	<=1	<=1	<=0.25		>2
<=1	8	8	<=1	8	<=0.25		>2
4	<=4	>16	>32	<=1	<=0.5	>64	>4
<=1	8	8	<=1	<=1	<=0.5	>64	>2
<=1	<=4	4	<=1	>8	<=0.5	>64	>2
<=1	<=4	8	<=1	<=1	<=0.5	>64	<=0.5
<=1	<=4	4	<=1	<=1	<=0.25		>2
<=1	<=4	4	<=1	<=1	<=0.25		<=0.5
<=1	<=4	4	<=1	<=1	<=0.25		>2
<=1	<=4	4	<=1	<=1	<=0.25		>2
<=1	<=4	4	<=1	<=1	<=0.25		<=0.5

<=1	>16	>16	8	>8	<=0.5	>64	>4
<=1	<=4	4	<=1	<=1	<=0.25		<=0.5
<=1	<=4	4	<=1	<=1	<=0.25		>2
2	16	>16	>32	>8	<=0.25		2
<=1	<=4	4	<=1	<=1	<=0.25		<=0.5
<=1	<=4	8	<=1	>8	<=0.25		>2
<=1	<=4	4	<=1	>8	<=0.25		>2
2	>16	>16	>32	>8	1	>64	>4
<=1	<=4	4	<=1	<=1	<=0.5	<=4	<=0.5
<=1	<=4	4	<=1	<=1	<=0.5	<=4	<=0.5
<=1	<=4	4	<=1	<=1	<=0.5	>64	<=0.5
<=1	8	8	<=1	>8	<=0.5	>64	>2
<=1	<=4	4	<=1	<=1	<=0.5	>64	<=0.5
<=1	>16	>16	16	<=1	<=0.25		<=0.5
<=1	8	8	<=1	<=1	<=0.25		>2
<=1	<=4	4	<=1	<=1	<=0.25		<=0.5
<=1	>16	>16	8	<=1	<=0.25		>2
<=1	<=4	4	<=1	<=1	<=0.25		<=0.5
>16	<=4	>16	>32	>8	<=0.25		>2
<=1	<=4	<=2	<=1	<=1	<=0.25		<=0.5
<=1	<=4	4	<=1	<=1	<=0.25		>2
<=1	>16	>16	8	<=1	<=0.25		<=0.5
<=1	>16	16	4	<=1	<=0.25		>2
<=1	<=4	<=2	<=1	<=1	<=0.25		>2
<=1	<=4	<=2	<=1	<=1	<=0.25		<=0.5
<=1	<=4	4	<=1	<=1	<=0.25		<=0.5
<=1	8	4	<=1	<=1	<=0.25		>2
4	>16	>16	16	>8	<=0.25		<=0.5
<=1	8	4	<=1	<=1	<=0.5	32	>2
<=1	<=4	4	<=1	<=1	<=0.5	>64	>2
<=1	<=4	4	<=1	<=1	<=0.5	<=4	<=0.5
<=1	<=4	4	<=1	2	<=0.5	>64	>2
<=1	8	4	<=1	<=1	<=0.25		>2
<=1	<=4	4	<=1	<=1	<=0.25		<=0.5
<=1	<=4	4	<=1	>8	<=0.5	>64	>2
<=1	<=4	4	<=1	<=1	<=0.5	<=4	<=0.5
<=1	8	4	<=1	<=1	<=0.5	<=4	<=0.5
<=1	>16	>16	<=1	>8	<=0.5	>64	<=0.5
<=1	<=4	4	<=1	<=1	<=0.5	<=4	<=0.5
<=1	<=4	4	<=1	<=1	<=0.5	<=4	<=0.5
<=1	<=4	4	<=1	<=1	<=0.25		<=0.5
<=1	8	8	<=1	>8	<=0.5	>64	>2
<=1	<=4	4	<=1	<=1	<=0.5	<=4	<=0.5
<=1	8	8	<=1	<=1	<=0.5	>64	>2
<=1	<=4	8	<=1	<=1	<=0.5	>64	>2
<=1	<=4	4	<=1	<=1	<=0.5	>64	<=0.5
<=1	<=4	4	<=1	<=1	<=0.25		<=0.5
>16	8	>16	>32	<=1	<=0.25		>2
<=1	<=4	<=2	<=1	<=1	<=0.25		<=0.5
<=1	8	4	<=1	<=1	<=0.25		>2
<=1	<=4	4	<=1	>8	<=0.25		>2
<=1	<=4	4	<=1	<=1	<=0.25		>2
<=1	8	4	<=1	<=1	<=0.25		<=0.5
<=1	<=4	4	<=1	<=1	<=0.5	64	>2
<=1	<=4	4	<=1	<=1	<=0.25		<=0.5
<=1	<=4	<=2	<=1	<=1	<=0.25		<=0.5
<=1	<=4	4	<=1	<=1	<=0.25		>2
8	<=4	>16	32	<=1	<=0.5	>64	>4
<=1	<=4	8	<=1	<=1	<=0.5	<=4	<=0.5
<=1	<=4	4	<=1	<=1	<=0.5	<=4	<=0.5
<=1	<=4	8	<=1	<=1	<=0.5	>64	>2

<=1	8	4	<=1	<=1	<=0.25		<=0.5
<=1	<=4	4	<=1	<=1	<=0.25		<=0.5
>16	>16	>16	>32	>8	0.5		>2
>16	>16	>16	>32	>8	<=0.25		>2
<=1	8	4	<=1	<=1	<=0.25		<=0.5
<=1	<=4	4	<=1	<=1	<=0.25		>2
<=1	16	4	<=1	<=1	<=0.25		<=0.5
<=1	<=4	8	<=1	2	<=0.5	>64	>2
<=1	>16	>16	8	>8	<=0.5	>64	>4
<=1	<=4	<=2	<=1	<=1	<=0.25		>2
<=1	<=4	4	<=1	<=1	<=0.25		<=0.5
<=1	<=4	8	<=1	<=1	<=0.25		<=0.5
<=1	<=4	4	<=1	<=1	<=0.25		<=0.5
<=1	<=4	8	<=1	<=1	<=0.25		<=0.5
>16	>16	>16	>32	>8	<=0.5	>64	<=0.5
<=1	>16	>16	16	<=1	<=0.25		>2
<=1	<=4	<=2	<=1	4	<=0.25		>2
<=1	<=4	8	<=1	2	<=0.25		<=0.5
>16	8	>16	>32	>8	<=0.5	>64	>4
<=1	>16	>16	8	<=1	<=0.25		>2
<=1	<=4	4	<=1	<=1	<=0.25		>2
<=1	<=4	4	<=1	<=1	<=0.25		<=0.5
<=1	<=4	4	<=1	<=1	<=0.25		>2
<=1	<=4	4	<=1	>8	<=0.25		>2
<=1	<=4	8	<=1	<=1	<=0.5	>64	>2
>16	>16	>16	>32	>8	<=0.5	>64	<=0.5
<=1	<=4	8	<=1	<=1	<=0.5	8	<=0.5
2	>16	>16	>32	<=1	<=0.25		>2
<=1	8	4	<=1	<=1	<=0.25		>2
<=1	>16	>16	8	>8	<=0.25		<=0.5
<=1	<=4	4	<=1	>8	<=0.25		>2
<=1	<=4	4	<=1	<=1	<=0.25		>2
<=1	<=4	4	<=1	<=1	<=0.25		>2
<=1	<=4	<=2	<=1	<=1	<=0.25		>2
8	<=4	>16	>32	>8	<=0.25		>2
<=1	>16	>16	8	<=1	<=0.25		>2
<=1	8	4	<=1	<=1	<=0.25		>2
<=1	<=4	<=2	<=1	<=1	<=0.25		<=0.5
<=1	8	4	<=1	<=1	<=0.25		>2
<=1	<=4	4	<=1	<=1	<=0.25		<=0.5
<=1	<=4	4	<=1	<=1	<=0.25		<=0.5
<=1	>16	>16	32	>8	<=0.25		>2
<=1	<=4	4	<=1	<=1	<=0.25		>2
<=1	<=4	<=2	<=1	<=1	<=0.25		<=0.5
<=1	<=4	<=2	<=1	>8	<=0.25		1
<=1	<=4	4	<=1	<=1	<=0.25		<=0.5
2	>16	>16	32	>8	<=0.25		>2
<=1	<=4	8	<=1	<=1	<=0.25		>2
<=1	<=4	4	<=1	<=1	<=0.25		<=0.5
<=1	<=4	4	<=1	<=1	<=0.5	<=4	<=0.5
<=1	16	16	<=1	<=1	<=0.5	<=4	<=0.5
<=1	<=4	<=2	<=1	<=1	<=0.5	>64	<=0.5
<=1	<=4	4	<=1	<=1	<=0.5	<=4	<=0.5
<=1	<=4	<=2	<=1	<=1	<=0.5	<=4	<=0.5
>16	>16	>16	>32	>8	<=0.5	>64	<=0.5
>16	>16	>16	>32	8	<=0.25		>2
<=1	<=4	4	<=1	<=1	<=0.5	>64	>2
<=1	<=4	8	<=1	<=1	<=0.5	<=4	>2
<=1	<=4	4	<=1	>8	<=0.5	32	>2
>16	<=4	>16	>32	<=1	<=0.25		<=0.5
<=1	<=4	4	<=1	<=1	<=0.5	>64	>2

>16	8	>16	>32	<=1	<=0.25		>2
<=1	>16	>16	16	>8	<=0.25		<=0.5
<=1	>16	16	4	>8	<=0.25		>2
<=1	<=4	4	<=1	<=1	<=0.5	<=4	<=0.5
<=1	<=4	8	<=1	>8	<=0.5	>64	>2
<=1	<=4	8	<=1	<=1	<=0.5	<=4	<=0.5
<=1	>16	>16	16	<=1	<=0.5	>64	>2
2	>16	>16	8	>8	<=0.5	>64	>4
<=1	>16	>16	8	<=1	<=0.5	32	<=0.5
<=1	8	8	<=1	>8	<=0.5	>64	<=0.5
<=1	>16	16	4	>8	<=0.5	>64	>2
<=1	<=4	4	<=1	<=1	<=0.5	>64	>2
<=1	16	8	<=1	>8	<=0.5	>64	>2
<=1	>16	>16	16	8	1	>64	>2
<=1	<=4	4	<=1	>8	<=0.5	>64	>2
<=1	<=4	<=2	<=1	<=1	<=0.25		<=0.5
<=1	<=4	4	<=1	<=1	<=0.25		<=0.5
<=1	<=4	4	<=1	<=1	<=0.25		<=0.5
<=1	<=4	4	<=1	<=1	<=0.25		<=0.5
<=1	<=4	4	<=1	4	<=0.25		<=0.5
<=1	<=4	4	<=1	>8	<=0.25		>2
<=1	<=4	4	<=1	<=1	<=0.5	>64	<=0.5
<=1	<=4	4	<=1	>8	<=0.25		>2
<=1	<=4	4	<=1	2	<=0.25		<=0.5
8	8	>16	>32	>8	<=0.25		>2
<=1	<=4	4	<=1	>8	<=0.25		>2
<=1	<=4	4	<=1	<=1	<=0.25		<=0.5
<=1	<=4	4	<=1	<=1	<=0.25		>2
<=1	<=4	4	<=1	<=1	<=0.25		<=0.5
<=1	<=4	4	<=1	<=1	<=0.25		<=0.5
<=1	<=4	8	<=1	<=1	<=0.25	>64	>4
<=1	<=4	4	<=1	<=1	<=0.25		>2
<=1	<=4	4	<=1	<=1	<=0.25		<=0.5
<=1	8	16	<=1	>8	1	>64	>4
<=1	<=4	4	<=1	<=1	<=0.25		<=0.5
<=1	<=4	4	<=1	<=1	<=0.25		>2
<=1	<=4	8	<=1	<=1	<=0.25		<=0.5
<=1	<=4	4	<=1	<=1	<=0.25		<=0.5
<=1	<=4	4	<=1	>8	<=0.25		>2
<=1	<=4	4	<=1	<=1	<=0.25		<=0.5
<=1	8	4	<=1	<=1	<=0.5	<=4	<=0.5
<=1	8	4	<=1	<=1	<=0.5	<=4	<=0.5
<=1	<=4	4	<=1	<=1	<=0.5	<=4	<=0.5
<=1	8	4	<=1	<=1	<=0.25		<=0.5
<=1	<=4	4	<=1	<=1	<=0.25		<=0.5
<=1	8	<=2	<=1	<=1	<=0.25		<=0.5
<=1	16	8	<=1	<=1	0.5		>2
<=1	<=4	4	<=1	<=1	<=0.25		>2
<=1	<=4	<=2	<=1	8	<=0.25		<=0.5
<=1	>16	>16	8	>8	<=0.25		>2
<=1	<=4	<=2	<=1	<=1	<=0.25		>2
<=1	<=4	<=2	<=1	<=1	<=0.25		<=0.5
<=1	<=4	8	<=1	<=1	<=0.25		>2
<=1	<=4	<=2	<=1	<=1	<=0.25		<=0.5
<=1	<=4	4	<=1	<=1	<=0.25		<=0.5
<=1	>16	>16	8	<=1	<=0.25		<=0.5
<=1	<=4	8	<=1	<=1	<=0.25		>2
<=1	8	8	<=1	4	<=0.25		>2
>16	<=4	>16	>32	<=1	<=0.25		>2
<=1	<=4	4	<=1	<=1	<=0.25		<=0.5
<=1	8	8	<=1	<=1	<=0.5	<=4	>2

<=1	8	8	<=1	>8	1	32	>4
<=1	<=4	<=2	<=1	<=1	<=0.5	>64	<=0.5
<=1	<=4	8	<=1	<=1	<=0.5	<=4	<=0.5
<=1	<=4	<=2	<=1	<=1	<=0.5	>64	<=0.5
<=1	<=4	4	<=1	>8	<=0.5	>64	>2
<=1	8	4	<=1	<=1	<=0.25		>2
<=1	<=4	4	<=1	<=1	<=0.25		<=0.5
<=1	>16	16	<=1	<=1	<=0.25		<=0.5
<=1	16	8	<=1	<=1	<=0.25		<=0.5
<=1	<=4	4	<=1	<=1	<=0.25		>2
<=1	>16	16	<=1	<=1	<=0.25		<=0.5
<=1	<=4	4	<=1	<=1	<=0.25		<=0.5
>16	8	>16	>32	>8	<=0.25		>2
<=1	<=4	<=2	<=1	<=1	<=0.25		<=0.5
<=1	<=4	4	<=1	4	<=0.25		>2
<=1	<=4	4	<=1	<=1	<=0.5	>64	>2
<=1	>16	>16	8	>8	<=0.25		>2
<=1	<=4	4	<=1	<=1	<=0.5	<=4	<=0.5
16	>16	>16	>32	>8	<=0.5	>64	>4
<=1	<=4	4	<=1	<=1	<=0.25		<=0.5
<=1	<=4	4	<=1	<=1	<=0.5	>64	>2
<=1	<=4	4	<=1	<=1	<=0.5	<=4	<=0.5
<=1	<=4	4	<=1	>8	<=0.25		>2
<=1	>16	>16	16	>8	<=0.25		>2
<=1	<=4	<=2	<=1	<=1	<=0.25		<=0.5
<=1	<=4	4	<=1	<=1	<=0.25		<=0.5
<=1	<=4	<=2	<=1	<=1	<=0.5	64	>2
<=1	>16	16	<=1	>8	<=0.5	>64	>2
<=1	<=4	4	<=1	<=1	<=0.5	<=4	<=0.5
<=1	8	8	<=1	<=1	<=0.5	<=4	<=0.5
<=1	<=4	4	<=1	<=1	<=0.5	>64	>2
<=1	<=4	8	<=1	<=1	<=0.5	>64	<=0.5
<=1	<=4	4	<=1	<=1	<=0.5	<=4	<=0.5
<=1	<=4	8	<=1	<=1	<=0.5	>64	<=0.5
<=1	<=4	16	<=1	<=1	<=0.5	<=4	>2
<=1	<=4	4	<=1	<=1	<=0.5	>64	>2
<=1	<=4	8	<=1	2	<=0.25		<=0.5
<=1	16	8	<=1	>8	<=0.25		>2
<=1	<=4	<=2	<=1	2	<=0.25		<=0.5
8	<=4	>16	>32	<=1	<=0.25		<=0.5
<=1	>16	16	<=1	<=1	1	<=4	<=0.5
2	>16	>16	>32	<=1	<=0.25		<=0.5
<=1	<=4	4	<=1	>8	<=0.25		>2
<=1	<=4	4	<=1	<=1	<=0.25		<=0.5
<=1	<=4	4	<=1	<=1	<=0.25		<=0.5
<=1	>16	>16	<=1	<=1	<=0.25		<=0.5
>16	8	>16	>32	<=1	<=0.25		<=0.5
<=1	<=4	4	<=1	<=1	<=0.25		>2
<=1	>16	>16	8	<=1	<=0.25		>2
<=1	<=4	<=2	<=1	>8	<=0.25		>2
<=1	<=4	4	<=1	<=1	<=0.5	<=4	<=0.5
<=1	>16	>16	>32	4	0.5		<=0.5
>16	>16	>16	>32	>8	0.5		>2
<=1	8	4	<=1	<=1	<=0.25		<=0.5
<=1	<=4	4	<=1	>8	<=0.5	>64	>2
<=1	>16	>16	8	>8	<=0.5	32	<=0.5
>16	>16	>16	>32	>8	<=0.5	>64	>4
>16	>16	>16	>32	>8	1	>64	>4
<=1	<=4	4	<=1	<=1	<=0.25		>2
<=1	<=4	4	<=1	<=1	<=0.25		>2
<=1	<=4	4	<=1	<=1	<=0.25		>2

<=1		<=4	8	<=1		<=1	<=0.25		<=0.5
<=1		<=4	4	<=1		<=1	<=0.25		>2
<=1		<=4	4	<=1		<=1	<=0.25		<=0.5
<=1		8	8	<=1		<=1	<=0.25		>2
<=1		<=4	4	<=1		>8	<=0.25		>2
<=1		<=4	4	<=1		<=1	<=0.25		<=0.5
<=1		<=4	4	<=1		<=1	<=0.25		<=0.5
<=1		<=4	4	<=1		<=1	<=0.25		<=0.5
<=1		<=4	<=2	<=1		<=1	<=0.25		>2
<=1		<=4	4	<=1		<=1	<=0.25		>2
<=1		<=4	4	<=1		<=1	<=0.25		<=0.5
<=1		8	4	<=1		<=1	<=0.25		>2
<=1		8	4	<=1		>8	<=0.25		>2
<=1		8	8	<=1		2	<=0.25		<=0.5
<=1		8	8	<=1		<=1	<=0.25		<=0.5
<=1		<=4	<=2	<=1		<=1	<=0.25		>2
>16		16	>16	>32		>8	<=0.25		>2
<=1		<=4	4	<=1		>8	<=0.25		<=0.5
<=1		<=4	4	<=1		<=1	<=0.25		>2
<=1		<=4	8	<=1		<=1	<=0.5	<=4	<=0.5
<=1		<=4	4	<=1		<=1	<=0.5	>64	>2
<=1		<=4	4	<=1		8	<=0.5	>64	>2
<=1		<=4	8	<=1		<=1	<=0.5	>64	>2
<=1		16	8	<=1		<=1	<=0.5	<=4	<=0.5
<=1		<=4	4	<=1		<=1	<=0.25		<=0.5
<=1		<=4	4	<=1		<=1	<=0.5	<=4	>2
<=1		<=4	4	<=1		>8	<=0.25		<=0.5
<=1		8	4	<=1		<=1	<=0.25		<=0.5
<=1		<=4	4	<=1		<=1	<=0.25		>2
<=1		<=4	4	<=1		<=1	<=0.25		<=0.5
<=1		<=4	8	<=1		2	<=0.25		<=0.5
<=1		>16	>16	16		>8	<=0.25		>2
<=1		16	8	<=1		4	<=0.25		>2
<=1		>16	>16	32		<=1	<=0.25		>2
<=1		16	8	<=1		<=1	<=0.25		>2
<=1		<=4	4	<=1		<=1	<=0.25		>2
<=1		16	16	<=1		<=1	<=0.25		>2
<=1		<=4	<=2	<=1		<=1	<=0.25		<=0.5
<=1		<=4	<=2	<=1		<=1	<=0.25		>2
<=1		<=4	16	<=1		2	0.5		<=0.5
<=1		<=4	4	<=1		>8	<=0.25		>2
<=1		8	4	<=1		>8	<=0.25		>2
<=1		8	4	<=1		>8	<=0.25		>2
<=1		<=4	4	<=1		>8	<=0.25		<=0.5
<=1		<=4	4	<=1		<=1	<=0.25		<=0.5
<=1		<=4	4	<=1		>8	<=0.25		>2
<=1		<=4	4	<=1		<=1	<=0.5	>64	>2
>16		16	>16	>32		<=1	<=0.25		<=0.5
<=1		>16	>16	32		>8	<=0.25		>2
<=1		>16	>16	8		>8	<=0.25		>2
<=1		<=4	4	<=1		4	<=0.25		<=0.5
<=1		<=4	<=2	<=1		2	<=0.25		<=0.5
<=1		<=4	<=2	<=1		<=1	<=0.5	<=4	<=0.5
<=1		<=4	<=2	<=1		>8	<=0.5	<=4	<=0.5
<=1		>16	>16	32		<=1	<=0.5	>64	>4
<=1		>16	>16	8		>8	<=0.5	>64	>4
<=1		<=4	4	<=1		<=1	<=0.25		>2
<=1		<=4	4	<=1		<=1	<=0.25		>2
<=1		<=4	4	<=1		<=1	<=0.25		<=0.5
<=1		<=4	8	<=1		<=1	<=0.5	<=4	<=0.5
<=1		>16	16	8		2	<=0.5	>64	>4

<=1	<=4	16	<=1	<=1	<=0.5	>64	<=0.5
<=1	<=4	8	<=1	2	<=0.5	>64	<=0.5
<=1	<=4	4	<=1	<=1	<=0.25		<=0.5
<=1	<=4	<=2	<=1	<=1	<=0.5	<=4	<=0.5
<=1	<=4	4	<=1	<=1	<=0.5	<=4	<=0.5
<=1	<=4	4	<=1	<=1	<=0.5	<=4	>2
<=1	<=4	4	<=1	<=1	<=0.5	<=4	<=0.5
<=1	<=4	<=2	<=1	<=1	<=0.5	>64	<=0.5
<=1	<=4	4	<=1	<=1	<=0.5	>64	>2
<=1	<=4	4	<=1	<=1	<=0.5	>64	<=0.5
<=1	8	4	<=1	>8	<=0.5	>64	>2
<=1	<=4	4	<=1	<=1	<=0.5	>64	<=0.5
<=1	>16	>16	16	<=1	<=0.5	64	<=0.5
<=1	>16	>16	32	>8	<=0.5	>64	>4
<=1	<=4	4	<=1	<=1	<=0.5	<=4	<=0.5
<=1	<=4	8	<=1	<=1	<=0.5	>64	<=0.5
<=1	<=4	<=2	<=1	<=1	<=0.5	<=4	<=0.5
<=1	<=4	<=2	<=1	<=1	<=0.5	<=4	<=0.5
<=1	>16	>16	16	2	<=0.25		>2
<=1	<=4	4	<=1	<=1	<=0.25		<=0.5
<=1	<=4	4	<=1	<=1	<=0.25		<=0.5
<=1	<=4	<=2	<=1	<=1	<=0.25		<=0.5
<=1	<=4	4	<=1	<=1	<=0.25		<=0.5
<=1	<=4	<=2	<=1	<=1	<=0.25		<=0.5
16	>16	>16	>32	>8	<=0.25		<=0.5
<=1	8	4	<=1	<=1	<=0.25		<=0.5
<=1	<=4	4	<=1	<=1	<=0.25		<=0.5
<=1	<=4	8	<=1	<=1	1	>64	<=0.5
<=1	16	8	<=1	>8	<=0.25		>2
<=1	<=4	4	<=1	<=1	<=0.5	>64	<=0.5
<=1	<=4	8	<=1	<=1	<=0.5	>64	>4
<=1	8	4	<=1	<=1	<=0.25		1
<=1	8	4	<=1	2	<=0.5	>64	>2
<=1	<=4	16	<=1	2	<=0.5	<=4	<=0.5
>16	8	>16	>32	>8	<=0.5	>64	<=0.5
<=1	>16	>16	8	<=1	<=0.5	32	<=0.5
<=1	>16	16	<=1	>8	<=0.5	>64	>2
<=1	<=4	4	<=1	4	<=0.5	>64	<=0.5
<=1	<=4	4	<=1	<=1	<=0.25		<=0.5
<=1	<=4	4	<=1	>8	<=0.25		>2
<=1	16	16	<=1	<=1	<=0.25		>2
<=1	>16	>16	16	>8	<=0.25		>2
>16	16	>16	>32	>8	<=0.25		>2
<=1	<=4	4	<=1	<=1	<=0.25		<=0.5
8	>16	>16	>32	>8	<=0.25		<=0.5
<=1	<=4	8	<=1	>8	<=0.25		>2
<=1	<=4	4	<=1	2	<=0.25		>2
<=1	<=4	4	<=1	<=1	<=0.25		>2
<=1	>16	>16	16	<=1	<=0.25		<=0.5
<=1	16	>16	32	<=1	<=0.25		>2
<=1	<=4	<=2	<=1	<=1	<=0.25		<=0.5
<=1	>16	8	2	>8	<=0.25		1
<=1	<=4	4	<=1	<=1	<=0.25		<=0.5
<=1	8	4	<=1	<=1	<=0.25		>2
<=1	<=4	4	<=1	<=1	<=0.25		<=0.5
<=1	8	4	<=1	<=1	<=0.25		<=0.5
<=1	<=4	4	<=1	<=1	<=0.25		>2
<=1	8	4	<=1	<=1	<=0.25		<=0.5
>16	<=4	>16	>32	<=1	<=0.25		>2
>16	>16	>16	>32	>8	<=0.25		>2
<=1	>16	>16	8	>8	<=0.25		>2

<=1	>16	>16	8	<=1	<=0.25		<=0.5
<=1	<=4	4	<=1	<=1	<=0.25		<=0.5
<=1	8	8	<=1	<=1	<=0.25		<=0.5
<=1	16	16	<=1	>8	<=0.25		>2
<=1	<=4	4	<=1	<=1	<=0.25		>2
<=1	<=4	4	<=1	<=1	<=0.25		<=0.5
<=1	<=4	4	<=1	<=1	<=0.25		>2
<=1	8	4	<=1	<=1	<=0.25		<=0.5
<=1	<=4	4	<=1	<=1	<=0.25		<=0.5
<=1	8	8	<=1	<=1	<=0.25		<=0.5
2	8	>16	32	<=1	<=0.25		<=0.5
<=1	<=4	4	<=1	<=1	<=0.25		<=0.5
<=1	8	8	<=1	>8	<=0.25		>2
<=1	<=4	4	<=1	>8	<=0.25		>2
>16	<=4	>16	>32	>8	<=0.5	>64	>4
<=1	<=4	4	<=1	<=1	<=0.25		<=0.5
<=1	<=4	4	<=1	<=1	<=0.5	>64	<=0.5
<=1	<=4	4	<=1	<=1	<=0.5	<=4	<=0.5
<=1	<=4	4	<=1	<=1	<=0.5	<=4	>2
>16	>16	>16	>32	>8	<=0.5	>64	>4
<=1	<=4	4	<=1	<=1	<=0.5	<=4	>2
<=1	8	4	<=1	<=1	<=0.25		>2
<=1	>16	>16	8	<=1	<=0.5	>64	>4
<=1	8	8	<=1	>8	<=0.25		>2
<=1	<=4	8	<=1	<=1	<=0.5	>64	>2
<=1	8	4	<=1	<=1	<=0.25		<=0.5
<=1	<=4	<=2	<=1	<=1	<=0.25		<=0.5
<=1	16	8	<=1	<=1	<=0.25		>2
<=1	<=4	8	<=1	<=1	<=0.5	>64	<=0.5
<=1	<=4	4	<=1	<=1	<=0.5	<=4	<=0.5
<=1	<=4	4	<=1	<=1	<=0.25		>2
<=1	<=4	<=2	<=1	>8	<=0.25		>2
<=1	<=4	4	<=1	>8	<=0.25		>2
<=1	<=4	4	<=1	>8	<=0.25		>2
>16	8	>16	>32	>8	<=0.25		>2
8	8	>16	>32	>8	<=0.25		<=0.5
<=1	<=4	4	<=1	>8	<=0.25		>2
<=1	<=4	4	<=1	<=1	<=0.5	<=4	<=0.5
<=1	<=4	8	<=1	>8	<=0.5	>64	<=0.5
<=1	<=4	4	<=1	<=1	<=0.5	>64	>2
<=1	<=4	8	<=1	<=1	<=0.5	>64	<=0.5
<=1	<=4	4	<=1	<=1	<=0.5	<=4	<=0.5
<=1	<=4	8	<=1	<=1	<=0.5	>64	>2
<=1	<=4	4	<=1	<=1	<=0.5	<=4	<=0.5
<=1	<=4	4	<=1	<=1	<=0.5	>64	<=0.5
<=1	<=4	4	<=1	<=1	<=0.5	>64	<=0.5
<=1	<=4	4	<=1	<=1	<=0.5	>64	<=0.5
<=1	<=4	4	<=1	<=1	<=0.25		<=0.5
<=1	<=4	8	<=1	8	<=0.5	>64	>2
<=1	<=4	4	<=1	<=1	<=0.5	<=4	<=0.5
>16	16	>16	>32	2	<=0.5	>64	>4
<=1	>16	>16	8	<=1	<=0.5	64	<=0.5
<=1	<=4	8	<=1	<=1	<=0.5	<=4	<=0.5
<=1	>16	>16	16	<=1	<=0.5	>64	<=0.5
<=1	<=4	4	<=1	<=1	<=0.5	<=4	<=0.5
<=1	<=4	<=2	<=1	<=1	<=0.5	<=4	<=0.5
<=1	<=4	<=2	<=1	2	<=0.25		1
<=1	<=4	8	<=1	<=1	<=0.25		<=0.5
<=1	<=4	8	<=1	>8	<=0.25		<=0.5
<=1	<=4	4	<=1	<=1	<=0.25		<=0.5
<=1	<=4	4	<=1	<=1	<=0.25		<=0.5

<=1	<=4	<=2	<=1	<=1	<=0.25		<=0.5
<=1	<=4	4	<=1	>8	<=0.25		>2
<=1	8	4	<=1	<=1	<=0.25		<=0.5
<=1	8	8	<=1	<=1	<=0.25		<=0.5
<=1	8	8	<=1	>8	<=0.25		>2
<=1	8	4	<=1	<=1	<=0.25		<=0.5
<=1	<=4	<=2	<=1	<=1	<=0.25		<=0.5
<=1	8	4	<=1	8	<=0.25		>2
<=1	<=4	4	<=1	<=1	<=0.25		<=0.5
<=1	>16	>16	8	>8	<=0.25		>2
<=1	<=4	4	<=1	8	<=0.25		>2
<=1	<=4	<=2	<=1	<=1	<=0.25		<=0.5
<=1	<=4	4	<=1	<=1	<=0.5	>64	<=0.5
<=1	<=4	4	<=1	<=1	<=0.5	>64	<=0.5
<=1	>16	>16	>32	>8	<=0.5	>64	>4
<=1	<=4	4	<=1	<=1	<=0.5	>64	>2
<=1	<=4	4	<=1	<=1	<=0.5	>64	<=0.5
>16	>16	>16	>32	>8	<=0.5	>64	>4
4	8	>16	>32	2	<=0.5	>64	>4
<=1	<=4	4	<=1	>8	<=0.25		>2
<=1	8	4	<=1	<=1	<=0.25		<=0.5
<=1	<=4	8	<=1	<=1	<=0.25		>2
<=1	>16	>16	>32	<=1	0.5		<=0.5
<=1	8	4	<=1	2	<=0.25		>2
<=1	>16	>16	8	<=1	<=0.25		<=0.5
<=1	<=4	<=2	<=1	<=1	<=0.25		>2
<=1	<=4	4	<=1	<=1	<=0.25		<=0.5
<=1	<=4	4	<=1	<=1	<=0.25		<=0.5
>16	>16	>16	>32	>8	<=0.25		>2
>16	>16	>16	>32	>8	<=0.25		>2
<=1	16	4	<=1	<=1	<=0.25		<=0.5
<=1	<=4	4	<=1	<=1	<=0.25		>2
<=1	<=4	<=2	<=1	<=1	<=0.25		<=0.5
<=1	<=4	<=2	<=1	<=1	<=0.25		<=0.5
<=1	8	8	<=1	2	<=0.25		>2
<=1	<=4	<=2	<=1	<=1	<=0.25		<=0.5
<=1	<=4	<=2	<=1	<=1	<=0.25		<=0.5
<=1	<=4	<=2	<=1	<=1	<=0.25		>2
<=1	<=4	4	<=1	<=1	<=0.25		<=0.5
<=1	<=4	4	<=1	2	<=0.5	<=4	<=0.5
<=1	8	8	<=1	<=1	<=0.25		>2
<=1	<=4	<=2	<=1	<=1	<=0.25		<=0.5
<=1	<=4	4	<=1	<=1	<=0.25		<=0.5
<=1	<=4	4	<=1	<=1	<=0.25		<=0.5
<=1	<=4	8	<=1	<=1	<=0.25		<=0.5
<=1	<=4	4	<=1	<=1	<=0.25		<=0.5
<=1	<=4	4	<=1	<=1	<=0.25		<=0.5
<=1	16	8	<=1	<=1	<=0.25		>2
<=1	<=4	<=2	<=1	>8	<=0.25		<=0.5
<=1	<=4	8	<=1	>8	<=0.25		>2
<=1	<=4	<=2	<=1	8	<=0.25		>2
<=1	<=4	4	<=1	<=1	<=0.25		<=0.5
<=1	<=4	<=2	<=1	<=1	<=0.25		<=0.5
<=1	<=4	4	<=1	<=1	<=0.25		<=0.5
<=1	<=4	4	<=1	<=1	<=0.25		<=0.5
<=1	<=4	8	<=1	<=1	<=0.25		<=0.5
<=1	<=4	4	<=1	8	<=0.25		>2
<=1	<=4	<=2	<=1	<=1	<=0.25		>2
<=1	>16	>16	<=1	<=1	<=0.25		<=0.5
<=1	<=4	4	<=1	<=1	<=0.25		>2
>16	>16	>16	>32	2	<=0.5	>64	>4

<=1		<=4	4	<=1		<=1	<=0.25		<=0.5
<=1		8	4	<=1		<=1	<=0.25		>2
<=1		8	4	<=1		<=1	<=0.25		<=0.5
<=1		<=4	4	<=1		<=1	<=0.25		<=0.5
<=1		<=4	4	<=1		<=1	<=0.25		>2
<=1		<=4	16	<=1		<=1	<=0.25		<=0.5
<=1		8	4	<=1		<=1	<=0.25		>2
<=1		<=4	8	<=1		2	<=0.5	<=4	<=0.5
<=1		<=4	4	<=1		<=1	<=0.5	64	>2
<=1		<=4	4	<=1		>8	<=0.5	>64	>2
<=1		<=4	8	<=1		4	<=0.5	>64	>2
<=1		<=4	<=2	<=1		>8	<=0.5	64	<=0.5
<=1		>16	16	2		<=1	<=0.5	>64	>4
<=1		<=4	4	<=1		<=1	<=0.25		>2
<=1		<=4	4	<=1		<=1	<=0.25		>2
<=1		<=4	8	<=1		<=1	<=0.25		<=0.5
<=1		<=4	16	<=1		<=1	<=0.25		<=0.5
<=1		<=4	4	<=1		>8	<=0.25		>2
<=1		<=4	4	<=1		<=1	<=0.25		<=0.5
<=1		<=4	8	<=1		<=1	<=0.5	<=4	<=0.5
<=1		<=4	4	<=1		4	<=0.25		>2
<=1		16	16	<=1		>8	<=0.25		>2
<=1		8	4	<=1		>8	<=0.25		>2
<=1		16	8	<=1		<=1	<=0.25		<=0.5
<=1		8	4	<=1		>8	<=0.25		>2
<=1		<=4	8	<=1		<=1	<=0.5	>64	<=0.5
<=1		<=4	4	<=1		<=1	<=0.25		<=0.5
<=1		>16	16	4		<=1	<=0.25		<=0.5
<=1		<=4	8	<=1		<=1	<=0.25		>2
>16		8	>16	>32		>8	<=0.25		<=0.5
<=1		<=4	4	<=1		<=1	<=0.25		<=0.5
<=1		<=4	>16	8		<=1	<=0.25		<=0.5
<=1		<=4	4	<=1		<=1	<=0.5	<=4	<=0.5
<=1		<=4	16	32		>8	<=0.5	>64	>2
<=1		<=4	4	<=1		<=1	<=0.5	>64	>2
<=1		<=4	16	<=1		<=1	<=0.5	>64	>2
<=1		<=4	8	<=1		<=1	<=0.5	>64	<=0.5
<=1		<=4	4	<=1		<=1	<=0.5	>64	<=0.5
<=1		<=4	4	<=1		<=1	<=0.5	>64	>2
<=1		<=4	<=2	<=1		<=1	<=0.5	>64	>2
8		>16	>16	32		>8	1	>64	>4
<=1		<=4	4	<=1		<=1	<=0.5	>64	>2
<=1		>16	>16	16		>8	<=0.5	>64	>4
<=1		<=4	<=2	<=1		<=1	<=0.5	<=4	<=0.5
<=1		<=4	4	<=1		<=1	<=0.5	>64	>2
<=1		>16	16	4		<=1	<=0.5	>64	<=0.5
<=1		<=4	<=2	<=1		<=1	1	>64	<=0.5
<=1		>16	>16	8		<=1	<=0.25		>2
<=1		<=4	<=2	<=1		<=1	<=0.25		<=0.5
<=1		<=4	4	<=1		<=1	<=0.25		<=0.5
<=1		<=4	<=2	<=1		<=1	<=0.25		<=0.5
<=1		<=4	8	<=1		<=1	<=0.5	<=4	<=0.5
<=1		<=4	8	<=1		>8	<=0.5	>64	>2
<=1		<=4	4	<=1		<=1	<=0.5	<=4	<=0.5
<=1		<=4	4	<=1		<=1	<=0.5	>64	<=0.5
<=1		<=4	4	<=1		8	<=0.5	8	<=0.5
<=1		<=4	4	<=1		<=1	<=0.5	<=4	<=0.5
<=1		<=4	8	<=1		<=1	<=0.5	<=4	<=0.5
<=1		8	4	<=1		2	<=0.5	>64	>2
<=1		<=4	4	<=1		<=1	<=0.5	>64	>2
<=1		<=4	4	<=1		<=1	<=0.5	<=4	<=0.5

<=1	<=4	4	<=1	<=1	<=0.25		>2
<=1	<=4	8	<=1	<=1	<=0.5	>64	<=0.5
<=1	<=4	4	<=1	<=1	<=0.5	<=4	<=0.5
<=1	<=4	4	<=1	<=1	<=0.5	64	>2
<=1	<=4	4	<=1	<=1	<=0.5	>64	>2
<=1	<=4	8	<=1	<=1	<=0.5	>64	>2
<=1	8	16	<=1	<=1	<=0.5	<=4	<=0.5
<=1	<=4	<=2	<=1	<=1	<=0.5	<=4	<=0.5
<=1	<=4	4	<=1	<=1	<=0.5	>64	>4
<=1	<=4	<=2	<=1	<=1	<=0.5	<=4	<=0.5
<=1	8	16	<=1	>8	<=0.5	>64	<=0.5
<=1	<=4	4	<=1	>8	<=0.25		>2
<=1	16	8	<=1	>8	<=0.25		>2
<=1	<=4	4	<=1	<=1	<=0.25		1
<=1	<=4	<=2	<=1	<=1	<=0.25		<=0.5
<=1	<=4	4	<=1	2	<=0.25		<=0.5
<=1	<=4	4	<=1	<=1	<=0.25		>2
<=1	<=4	<=2	<=1	<=1	<=0.25		>2
<=1	<=4	4	<=1	<=1	<=0.5	<=4	<=0.5
<=1	<=4	4	<=1	<=1	<=0.5	>64	<=0.5
<=1	<=4	<=2	<=1	<=1	<=0.5	<=4	<=0.5
<=1	8	8	<=1	>8	<=0.25		>2
<=1	<=4	4	<=1	>8	<=0.25		>2
<=1	<=4	4	<=1	2	<=0.25		<=0.5
<=1	<=4	4	2	<=1	<=0.25		<=0.5
<=1	<=4	4	<=1	>8	<=0.25		>2
>16	>16	>16	>32	>8	0.5		>2
<=1	<=4	4	<=1	<=1	<=0.25		>2
<=1	<=4	4	<=1	<=1	<=0.25		<=0.5
<=1	<=4	4	<=1	2	<=0.25		<=0.5
<=1	<=4	<=2	<=1	<=1	<=0.5	<=4	<=0.5
<=1	<=4	4	<=1	>8	<=0.5	>64	>2
<=1	<=4	4	<=1	<=1	<=0.5	<=4	<=0.5
<=1	<=4	4	<=1	<=1	<=0.5	>64	<=0.5
<=1	<=4	8	<=1	<=1	<=0.5	>64	>2
<=1	8	4	<=1	<=1	<=0.5	<=4	<=0.5
<=1	<=4	4	<=1	<=1	<=0.25		<=0.5
<=1	<=4	4	<=1	<=1	<=0.25		>2
<=1	<=4	4	<=1	<=1	<=0.25		<=0.5
<=1	8	8	<=1	<=1	<=0.25		<=0.5
<=1	8	4	<=1	>8	<=0.5	>64	>2
8	<=4	>16	>32	<=1	<=0.25		<=0.5
<=1	<=4	4	<=1	<=1	<=0.5	<=4	<=0.5
>16	>16	>16	>32	>8	<=0.25		>2
<=1	<=4	<=2	<=1	<=1	<=0.5	>64	>2
16	>16	>16	>32	>8	<=0.5	>64	>4
<=1	<=4	4	<=1	>8	<=0.25		>2
<=1	<=4	4	<=1	<=1	<=0.25		<=0.5
<=1	<=4	4	<=1	<=1	<=0.25		>2
16	>16	>16	>32	>8	0.5		>2
<=1	<=4	4	<=1	<=1	<=0.25		>2
<=1	<=4	4	<=1	<=1	<=0.25		2
<=1	<=4	4	<=1	<=1	<=0.25		<=0.5
<=1	<=4	4	<=1	<=1	<=0.25		<=0.5
<=1	<=4	<=2	<=1	<=1	<=0.25		<=0.5
<=1	8	4	<=1	<=1	<=0.25		<=0.5
<=1	<=4	4	<=1	>8	<=0.25		>2
<=1	<=4	4	<=1	<=1	<=0.25		>2
<=1	<=4	8	<=1	<=1	<=0.5	<=4	<=0.5
<=1	>16	>16	16	>8	<=0.5	>64	>4
<=1	<=4	4	<=1	<=1	<=0.5	>64	<=0.5

	<=1		<=4	4	<=1		2	<=0.5	<=4	<=0.5
	<=1		<=4	8	<=1		<=1	<=0.5	>64	>2
	>16		8	>16	>32		>8	<=0.5	>64	>4
	<=1		<=4	<=2	<=1		>8	<=0.5	<=4	<=0.5
	<=1		<=4	4	<=1		<=1	<=0.5	>64	>2
	<=1		<=4	4	<=1		<=1	<=0.5	<=4	<=0.5
	<=1		>16	>16	32		<=1	<=0.5	>64	<=0.5
	2		<=4	8	<=1		>8	<=0.5	>64	>2
	<=1		<=4	4	<=1		<=1	<=0.5	>64	<=0.5
	<=1		<=4	4	<=1		<=1	<=0.5	>64	<=0.5
	<=1		<=4	8	<=1		<=1	1	<=4	>2
	<=1		<=4	4	<=1		<=1	<=0.5	<=4	<=0.5
	<=1		<=4	8	<=1		<=1	<=0.5	<=4	<=0.5
	<=1		8	8	<=1		2	<=0.5	<=4	<=0.5
	<=1		<=4	4	<=1		<=1	<=0.5	>64	<=0.5
	<=1		<=4	4	<=1		<=1	<=0.5	>64	>2
	<=1		<=4	8	<=1		<=1	<=0.25		<=0.5
	<=1		8	8	<=1		<=1	<=0.5	>64	<=0.5
	<=1		<=4	4	<=1		<=1	<=0.5	>64	>2
	<=1		<=4	8	<=1		<=1	<=0.5	<=4	<=0.5
<=0.25	16	0.5	8	>16	>32	16	>8	<=0.25		<=0.5
<=0.25	>16	1	16	>16	>32	32	>8	<=0.25		>2
<=0.25	>16	1	<=4	>16	>32	32	2	<=0.25		>2
<=0.25	<=1	0.5	8	8	<=1	32	2	<=0.25		>2
<=0.25	<=1	1	<=4	4	<=1	32	<=1	<=0.25		<=0.5
<=0.25	<=1	0.5	<=4	4	<=1	16	>8	<=0.25		<=0.5
<=0.25	<=1	1	<=4	<=2	<=1	32	<=1	<=0.25		<=0.5
<=0.25	<=1	2	<=4	4	<=1	32	<=1	<=0.25		<=0.5
<=0.25	<=1	0.5	<=4	4	<=1	32	<=1	<=0.25		<=0.5
<=0.25	<=1	2	<=4	4	<=1	32	<=1	<=0.25		>2
<=0.25	<=1	0.5	<=4	<=2	<=1	32	<=1	<=0.25		<=0.5
<=0.25	<=1	1	<=4	4	<=1	32	<=1	<=0.25		<=0.5
0.5	<=1	1	>16	>16	32	32	<=1	<=0.25		>2
<=0.25	<=1	1	<=4	4	<=1	32	<=1	<=0.25		>2
<=0.25	<=1		<=4	<=2	<=1		<=1	<=0.25		<=0.5
<=0.25	<=1	0.5	<=4	4	<=1	32	2	<=0.25		<=0.5
<=0.25	<=1	1	<=4	4	<=1	32	>8	<=0.25		>2
<=0.25	<=1	0.5	<=4	<=2	<=1	32	<=1	<=0.25		>2
<=0.25	<=1	1	<=4	8	<=1	32	<=1	<=0.25		<=0.5
0.5	2	0.5	>16	>16	>32	16	<=1	<=0.25		<=0.5
<=0.25	<=1	0.5	<=4	4	<=1	32	<=1	<=0.25		<=0.5
<=0.25	8	0.5	<=4	>16	>32	16	>8	<=0.25		<=0.5
<=0.25	<=1	1	<=4	<=2	<=1	32	<=1	<=0.25		<=0.5
<=0.25	<=1	4	<=4	8	<=1	32	<=1	<=0.25		<=0.5
<=0.25	<=1	0.5	<=4	8	<=1	32	<=1	<=0.25		>2
<=0.25	<=1	0.5	<=4	8	<=1	16	<=1	<=0.25		>2
<=0.25	<=1	1	<=4	<=2	<=1	8	2	<=0.25		>2
0.5	>16	1	<=4	>16	>32	32	<=1	<=0.25		<=0.5
<=0.25	8	1	<=4	>16	>32	16	>8	<=0.25		<=0.5
<=0.25	16	1	8	>16	>32	16	>8	<=0.25		<=0.5
<=0.25	<=1		<=4	8	<=1		>8	<=0.25		>2
<=0.25	<=1		<=4	4	<=1		<=1	<=0.25		<=0.5
<=0.25	<=1		16	8	<=1		<=1	<=0.25		<=0.5
<=0.25	<=1		<=4	4	<=1		<=1	<=0.25		<=0.5
<=0.25	<=1		<=4	8	<=1		<=1	<=0.25		>2
<=0.25	<=1		>16	>16	16		>8	<=0.25		>2
<=0.25	<=1		>16	>16	<=1		<=1	<=0.25		<=0.5
1	2	4	>16	>16	32	>256	>8	<=0.25		>2
<=0.25	>16	2	8	>16	>32	16	>8	<=0.25		>2
<=0.25	8	0.5	<=4	>16	>32	32	<=1	<=0.25		>2
<=0.25	>16		8	>16	>32		<=1	<=0.25		>2

<=0.25	16	1	<=4	>16	>32	32	>8	<=0.25	>2
<=0.25	4	2	8	>16	>32	64	<=1	<=0.25	>2
<=0.25	>16	2	8	>16	>32	16	<=1	<=0.25	>2
<=0.25	16	1	8	>16	>32	64	>8	<=0.25	<=0.5
<=0.25	>16	0.5	8	>16	>32	32	<=1	<=0.25	<=0.5
0.5	4	<=0.25	>16	>16	>32	32	<=1	0.5	<=0.5
<=0.25	8	0.5	>16	>16	>32	64	>8	<=0.25	>2
<=0.25	16	0.5	8	>16	>32	64	<=1	<=0.25	<=0.5
<=0.25	4	0.5	<=4	>16	32	32	>8	<=0.25	>2
<=0.25	>16	1	16	>16	>32	32	>8	<=0.25	>2
0.5	>16	1	>16	>16	>32	16	>8	0.5	>2
<=0.25	>16		8	>16	>32		>8	<=0.25	>2
<=0.25	<=1	1	<=4	>16	16	32	<=1	<=0.25	>2
<=0.25	>16	0.5	16	>16	>32	32	<=1	<=0.25	<=0.5
<=0.25	>16	1	<=4	>16	>32	32	>8	<=0.25	>2
<=0.25	>16	0.5	>16	>16	>32	16	>8	<=0.25	<=0.5
<=0.25	>16	1	<=4	>16	>32	16	>8	<=0.25	<=0.5
<=0.25	>16	1	8	>16	>32	32	>8	<=0.25	>2
<=0.25	>16	4	8	>16	>32	32	<=1	<=0.25	>2
<=0.25	>16	0.5	<=4	>16	>32	32	>8	<=0.25	>2
<=0.25	8		<=4	>16	>32		<=1	<=0.25	>2
<=0.25	<=1		<=4	4	<=1		<=1	<=0.25	<=0.5
<=0.25	<=1	1	>16	>16	8	32	<=1	<=0.25	<=0.5
<=0.25	<=1	1	<=4	4	<=1	32	<=1	<=0.25	>2
<=0.25	<=1	1	<=4	4	<=1	32	>8	<=0.25	>2
<=0.25	2	4	>16	>16	>32	32	>8	<=0.25	>2
<=0.25	<=1	1	<=4	8	<=1	32	>8	<=0.25	<=0.5
<=0.25	<=1	<=0.25	8	8	<=1	32	<=1	<=0.25	>2
<=0.25	<=1	2	<=4	8	<=1	32	<=1	<=0.25	<=0.5
<=0.25	<=1	0.5	<=4	8	<=1	32	<=1	<=0.25	<=0.5
<=0.25	<=1	1	8	8	<=1	128	>8	<=0.25	>2
<=0.25	<=1	2	<=4	8	<=1	32	<=1	<=0.25	<=0.5
<=0.25	<=1	4	<=4	4	<=1	32	<=1	<=0.25	>2
<=0.25	<=1	0.5	<=4	4	<=1	32	<=1	<=0.25	<=0.5
<=0.25	<=1	1	<=4	<=2	<=1	16	2	<=0.25	<=0.5
<=0.25	<=1		<=4	4	<=1		<=1	<=0.25	<=0.5
<=0.25	>16	2	8	>16	>32	32	>8	<=0.25	>2
<=0.25	>16	128	16	>16	>32	32	>8	<=0.25	<=0.5
<=0.25	<=1	1	8	4	<=1	16	2	<=0.25	<=0.5
<=0.25	<=1	1	8	8	<=1	32	<=1	<=0.25	>2
<=0.25	<=1		<=4	4	<=1		<=1	<=0.25	<=0.5
<=0.25	<=1		<=4	4	<=1		<=1	<=0.25	<=0.5
<=0.25	<=1		<=4	4	<=1		<=1	<=0.25	<=0.5
<=0.25	<=1	0.5	8	8	<=1	16	<=1	<=0.25	>2
<=0.25	<=1	0.5	<=4	8	<=1	32	<=1	<=0.25	>2
<=0.25	<=1		<=4	8	<=1		>8	<=0.25	>2
<=0.25	<=1	2	<=4	8	<=1	32	<=1	<=0.25	>2
<=0.25	2	1	>16	>16	>32	16	<=1	1	>2
<=0.25	<=1	1	8	4	<=1	32	<=1	<=0.25	<=0.5
<=0.25	<=1		<=4	4	<=1		<=1	<=0.25	>2
<=0.25	<=1	0.5	<=4	4	<=1	32	<=1	<=0.25	<=0.5
<=0.25	<=1	2	<=4	4	<=1	32	<=1	<=0.25	1
<=0.25	<=1	1	8	8	<=1	32	<=1	<=0.25	<=0.5
<=0.25	<=1	1	<=4	4	<=1	16	<=1	<=0.25	>2
<=0.25	<=1	1	<=4	4	<=1	32	>8	<=0.25	<=0.5
<=0.25	<=1		<=4	4	<=1		<=1	<=0.25	>2
<=0.25	<=1		<=4	4	<=1		<=1	<=0.25	<=0.5
<=0.25	>16		>16	>16	>32		<=1	<=0.25	>2
<=0.25	<=1	0.5	16	8	<=1	32	<=1	<=0.25	>2
<=0.25	<=1	0.5	<=4	4	<=1	32	<=1	<=0.25	<=0.5
<=0.25	<=1	1	<=4	4	<=1	32	<=1	<=0.25	>2

<=0.25	<=1	0.5	<=4	4	<=1	32	>8	<=0.25	<=0.5
<=0.25	<=1		8	8	<=1		<=1	<=0.25	>2
<=0.25	<=1		<=4	4	<=1		<=1	<=0.25	>2
<=0.25	<=1		8	4	<=1		<=1	<=0.25	<=0.5
<=0.25	<=1		<=4	4	<=1		<=1	<=0.25	<=0.5
1	4		>16	>16	>32		<=1	1	<=0.5
<=0.25	<=1		<=4	4	<=1		<=1	<=0.25	<=0.5
<=0.25	<=1		<=4	4	<=1		<=1	<=0.25	>2
<=0.25	<=1		<=4	<=2	<=1		<=1	<=0.25	<=0.5
<=0.25	<=1		<=4	<=2	<=1		<=1	<=0.25	>2
<=0.25	<=1		<=4	4	<=1		<=1	<=0.25	>2
<=0.25	<=1	1	8	16	<=1	256	<=1	<=0.25	>2
<=0.25	<=1	0.5	<=4	4	<=1	64	<=1	<=0.25	<=0.5
<=0.25	<=1	2	>16	>16	16	32	<=1	<=0.25	>2
<=0.25	<=1	0.5	>16	>16	16	16	>8	<=0.25	1
<=0.25	<=1	0.5	<=4	8	<=1	16	<=1	<=0.25	>2
<=0.25	<=1		8	8	<=1		<=1	<=0.25	<=0.5
<=0.25	<=1		<=4	4	<=1		<=1	<=0.25	>2
<=0.25	<=1		<=4	8	<=1		<=1	<=0.25	>2
<=0.25	>16		8	>16	>32		>8	<=0.25	>2
<=0.25	<=1		16	16	<=1		>8	<=0.25	>2
<=0.25	<=1		<=4	4	<=1		<=1	<=0.25	<=0.5
<=0.25	<=1		>16	>16	16		>8	<=0.25	>2
<=0.25	<=1	0.5	<=4	8	<=1	32	<=1	<=0.25	<=0.5
<=0.25	<=1	2	<=4	4	<=1	16	<=1	<=0.25	>2
<=0.25	<=1	4	>16	>16	8	256	>8	<=0.25	>2
<=0.25	<=1	2	16	16	<=1	32	>8	<=0.25	<=0.5
<=0.25	<=1	1	8	8	<=1	32	<=1	<=0.25	<=0.5
<=0.25	<=1	1	>16	>16	8	128	>8	<=0.25	>2
<=0.25	<=1	1	<=4	8	<=1	32	<=1	<=0.25	<=0.5
<=0.25	<=1	0.5	<=4	4	<=1	32	>8	<=0.25	>2
<=0.25	<=1		<=4	8	<=1		>8	<=0.25	>2
<=0.25	>16	1	8	>16	>32	16	>8	<=0.25	<=0.5
<=0.25	2	0.5	8	>16	>32	16	<=1	<=0.25	>2
<=0.25	<=1		8	8	<=1		<=1	0.5	>2
<=0.25	>16		<=4	>16	>32		>8	<=0.25	>2
<=0.25	<=1		<=4	4	<=1		<=1	<=0.25	>2
<=0.25	<=1		<=4	8	<=1		>8	<=0.25	<=0.5
<=0.25	<=1		8	4	<=1		<=1	<=0.25	<=0.5
<=0.25	>16		16	>16	>32		>8	<=0.25	1
<=0.25	<=1		8	8	<=1		<=1	<=0.25	>2
<=0.25	<=1		8	4	<=1		<=1	<=0.25	<=0.5
<=0.25	<=1		<=4	<=2	<=1		<=1	<=0.25	<=0.5
<=0.25	<=1		16	16	2		<=1	<=0.25	>2
<=0.25	<=1		16	16	<=1		>8	<=0.25	>2
<=0.25	<=1		<=4	8	<=1		<=1	<=0.25	>2
<=0.25	<=1	0.5	<=4	4	<=1	32	>8	<=0.25	>2
<=0.25	<=1	1	<=4	8	<=1	16	<=1	<=0.25	>2
<=0.25	>16	1	8	>16	>32	16	>8	<=0.25	<=0.5
<=0.25	>16	0.5	>16	>16	>32	128	>8	<=0.25	>2
<=0.25	<=1	2	<=4	4	<=1	32	<=1	<=0.25	>2
<=0.25	<=1	128	16	16	<=1	64	<=1	<=0.25	<=0.5
<=0.25	<=1	0.5	<=4	4	<=1	32	>8	<=0.25	<=0.5
<=0.25	<=1	2	>16	>16	16	32	>8	<=0.25	<=0.5
<=0.25	<=1	0.5	<=4	<=2	<=1	32	2	<=0.25	<=0.5
<=0.25	<=1	1	<=4	<=2	<=1	64	<=1	<=0.25	<=0.5
<=0.25	<=1	0.5	<=4	4	<=1	32	2	<=0.25	<=0.5
<=0.25	<=1	1	8	8	<=1	64	<=1	<=0.25	<=0.5
<=0.25	<=1	0.5	>16	>16	<=1	8	<=1	<=0.25	>2
<=0.25	<=1	1	<=4	4	<=1	16	<=1	<=0.25	<=0.5
<=0.25	<=1	0.5	<=4	<=2	<=1	32	<=1	<=0.25	<=0.5

<=0.25	<=1	64	<=4	4	<=1	32	2	<=0.25	<=0.5
<=0.25	<=1	0.5	8	8	<=1	16	<=1	<=0.25	>2
<=0.25	<=1	0.5	8	8	<=1	32	2	<=0.25	<=0.5
<=0.25	>16	2	<=4	>16	>32	16	<=1	<=0.25	>2
<=0.25	<=1	0.5	<=4	4	<=1	32	<=1	<=0.25	<=0.5
<=0.25	<=1		<=4	4	<=1		>8	<=0.25	>2
<=0.25	<=1	0.5	<=4	4	<=1	32	<=1	<=0.25	<=0.5
<=0.25	<=1		<=4	4	<=1		<=1	<=0.25	>2
<=0.25	4		<=4	>16	32		<=1	<=0.25	>2
<=0.25	<=1		<=4	<=2	<=1		<=1	<=0.25	<=0.5
<=0.25	<=1		<=4	4	<=1		<=1	<=0.25	<=0.5
<=0.25	<=1		<=4	8	<=1		<=1	<=0.25	>2
<=0.25	<=1		<=4	4	<=1		<=1	<=0.25	<=0.5
<=0.25	<=1		<=4	4	<=1		<=1	<=0.25	>2
<=0.25	<=1		16	8	<=1		<=1	<=0.25	<=0.5
<=0.25	<=1		<=4	4	<=1		2	<=0.25	>2
<=0.25	<=1		<=4	8	<=1		>8	<=0.25	<=0.5
<=0.25	<=1		<=4	<=2	<=1		<=1	<=0.25	<=0.5
<=0.25	<=1	2	<=4	4	<=1	32	<=1	<=0.25	<=0.5
<=0.25	<=1	0.5	>16	>16	8	16	<=1	<=0.25	<=0.5
<=0.25	<=1	1	>16	>16	32	32	>8	<=0.25	<=0.5
<=0.25	<=1		<=4	8	<=1		<=1	<=0.25	<=0.5
<=0.25	<=1	0.5	<=4	<=2	<=1	32	<=1	<=0.25	>2
<=0.25	<=1	0.5	<=4	4	<=1	32	<=1	<=0.25	<=0.5
<=0.25	<=1		<=4	8	<=1		<=1	<=0.25	1
<=0.25	<=1	1	8	8	<=1	32	4	<=0.25	>2
<=0.25	<=1	32	16	8	<=1	8	2	<=0.25	>2
<=0.25	<=1		<=4	4	<=1		<=1	<=0.25	>2
<=0.25	<=1		8	4	<=1		<=1	<=0.25	<=0.5
<=0.25	<=1		<=4	4	<=1		<=1	<=0.25	<=0.5
<=0.25	<=1		<=4	4	<=1		<=1	<=0.25	<=0.5
<=0.25	<=1		8	8	<=1		<=1	<=0.25	>2
<=0.25	8		16	>16	>32		<=1	<=0.25	>2
<=0.25	<=1		<=4	4	<=1		<=1	<=0.25	<=0.5
<=0.25	<=1		<=4	8	<=1		<=1	<=0.25	<=0.5
<=0.25	<=1		<=4	8	<=1		<=1	<=0.25	<=0.5
<=0.25	<=1		<=4	<=2	<=1		<=1	<=0.25	<=0.5
<=0.25	<=1		>16	>16	8		<=1	<=0.25	<=0.5
<=0.25	<=1	1	<=4	8	<=1	16	<=1	<=0.25	<=0.5
<=0.25	<=1	1	<=4	4	<=1	32	<=1	<=0.25	>2
<=0.25	<=1	1	<=4	<=2	<=1	32	>8	<=0.25	>2
<=0.25	<=1	1	<=4	4	<=1	16	<=1	<=0.25	<=0.5
<=0.25	<=1	0.5	<=4	4	<=1	32	<=1	<=0.25	<=0.5
<=0.25	<=1	1	<=4	4	<=1	16	<=1	<=0.25	>2
<=0.25	<=1	0.5	8	4	<=1	32	<=1	<=0.25	<=0.5
<=0.25	<=1	0.5	<=4	<=2	<=1	32	<=1	<=0.25	<=0.5
<=0.25	<=1	1	<=4	8	<=1	32	>8	<=0.25	<=0.5
<=0.25	<=1	2	<=4	4	<=1	32	<=1	<=0.25	<=0.5
<=0.25	<=1	2	<=4	8	8	32	<=1	<=0.25	<=0.5
<=0.25	<=1	0.5	<=4	4	<=1	32	<=1	<=0.25	<=0.5
<=0.25	<=1	2	<=4	4	<=1	32	<=1	<=0.25	<=0.5
<=0.25	<=1	0.5	<=4	<=2	<=1	32	<=1	<=0.25	<=0.5
<=0.25	<=1	0.5	8	8	<=1	32	<=1	<=0.25	<=0.5
<=0.25	<=1	1	<=4	8	<=1	16	<=1	<=0.25	>2
<=0.25	<=1	0.5	<=4	4	<=1	32	<=1	<=0.25	<=0.5
<=0.25	<=1	0.5	<=4	4	<=1	32	<=1	<=0.25	<=0.5
<=0.25	<=1	2	<=4	4	<=1	32	<=1	<=0.25	<=0.5
<=0.25	<=1	0.5	8	8	<=1	32	4	<=0.25	>2
<=0.25	<=1	1	<=4	4	<=1	32	2	<=0.25	>2
<=0.25	<=1	2	<=4	<=2	<=1	16	<=1	<=0.25	<=0.5
<=0.25	<=1		16	8	<=1		<=1	<=0.25	<=0.5

<=0.25	<=1	0.5	<=4	8	<=1	32	<=1	<=0.25	<=0.5
<=0.25	<=1	2	<=4	8	<=1	32	<=1	<=0.25	<=0.5
<=0.25	<=1	1	<=4	4	<=1	32	<=1	<=0.25	>2
<=0.25	<=1		<=4	<=2	<=1		<=1	<=0.25	<=0.5
<=0.25	<=1		<=4	4	<=1		<=1	<=0.25	>2
<=0.25	<=1		<=4	<=2	<=1		2	<=0.25	<=0.5
<=0.25	<=1		<=4	4	<=1		2	<=0.25	<=0.5
<=0.25	<=1		<=4	4	<=1		>8	<=0.25	>2
0.5	<=1	2	<=4	4	<=1	32	>8	<=0.25	>2
<=0.25	<=1	32	8	8	<=1	32	<=1	<=0.25	<=0.5
<=0.25	8		<=4	>16	>32		<=1	<=0.25	<=0.5
<=0.25	<=1	1	<=4	8	<=1	32	<=1	<=0.25	>2
<=0.25	<=1	0.5	<=4	4	<=1	32	<=1	<=0.25	<=0.5
<=0.25	<=1	1	<=4	8	<=1	32	<=1	<=0.25	<=0.5
<=0.25	<=1	2	<=4	8	<=1	16	2	<=0.25	>2
<=0.25	<=1	0.5	8	8	<=1	32	>8	<=0.25	<=0.5
<=0.25	<=1	0.5	<=4	8	<=1	32	<=1	<=0.25	<=0.5
<=0.25	<=1	1	<=4	4	<=1	32	<=1	<=0.25	>2
<=0.25	<=1	0.5	<=4	4	<=1	32	<=1	<=0.25	<=0.5
<=0.25	>16	1	<=4	>16	>32	16	>8	<=0.25	<=0.5
<=0.25	<=1	0.5	<=4	4	<=1	32	<=1	<=0.25	<=0.5
<=0.25	<=1	1	>16	>16	8	16	<=1	<=0.25	<=0.5
<=0.25	<=1	1	8	8	<=1	32	<=1	<=0.25	<=0.5
<=0.25	<=1	1	<=4	<=2	<=1	32	<=1	<=0.25	<=0.5
<=0.25	<=1	1	8	8	<=1	32	<=1	<=0.25	>2
<=0.25	<=1	0.5	<=4	4	<=1	16	<=1	<=0.25	>2
<=0.25	<=1	1	>16	>16	8	128	<=1	1	>2
<=0.25	<=1	1	<=4	8	<=1	32	<=1	<=0.25	<=0.5
<=0.25	<=1	256	<=4	4	<=1	32	<=1	<=0.25	<=0.5
<=0.25	<=1	2	<=4	4	<=1	32	<=1	<=0.25	<=0.5
<=0.25	<=1	1	8	<=2	<=1	32	<=1	<=0.25	>2
<=0.25	<=1	1	<=4	4	<=1	16	<=1	<=0.25	>2
0.5	>16	2	16	>16	>32	32	>8	<=0.25	<=0.5
<=0.25	<=1	0.5	<=4	4	<=1	32	<=1	<=0.25	<=0.5
<=0.25	<=1	1	<=4	<=2	<=1	32	<=1	<=0.25	<=0.5
<=0.25	<=1		>16	>16	32		<=1	<=0.25	<=0.5
<=0.25	<=1		<=4	8	<=1		<=1	<=0.25	<=0.5
<=0.25	<=1		<=4	4	<=1		>8	<=0.25	<=0.5
<=0.25	<=1		8	8	<=1		2	<=0.25	<=0.5
<=0.25	<=1		<=4	4	<=1		<=1	<=0.25	>2
<=0.25	2		<=4	>16	32		<=1	<=0.25	>2
<=0.25	<=1		<=4	4	<=1		>8	<=0.25	>2
<=0.25	>16		>16	>16	>32		>8	<=0.25	>2
<=0.25	<=1		<=4	8	<=1		<=1	<=0.25	<=0.5
<=0.25	<=1		16	8	<=1		<=1	<=0.25	<=0.5
<=0.25	>16	0.5	8	>16	>32	16	<=1	<=0.25	<=0.5
<=0.25	<=1	1	<=4	<=2	<=1	32	<=1	<=0.25	<=0.5
<=0.25	<=1	0.5	<=4	8	<=1	32	>8	<=0.25	<=0.5
<=0.25	<=1	1	<=4	8	<=1	32	<=1	<=0.25	>2
<=0.25	<=1	0.5	<=4	8	<=1	32	<=1	<=0.25	>2
<=0.25	<=1		<=4	4	<=1		<=1	<=0.25	>2
<=0.25	<=1		<=4	4	8		<=1	<=0.25	<=0.5
<=0.25	<=1	1	<=4	8	<=1	32	<=1	<=0.25	<=0.5
<=0.25	<=1	<=0.25	<=4	4	<=1	32	<=1	<=0.25	>2
<=0.25	<=1	1	<=4	4	<=1	32	<=1	<=0.25	<=0.5
<=0.25	<=1	2	>16	>16	8	64	2	0.5	<=0.5
<=0.25	<=1		<=4	4	<=1		<=1	<=0.25	<=0.5
<=0.25	<=1	0.5	<=4	<=2	<=1	32	<=1	<=0.25	<=0.5
<=0.25	<=1		<=4	4	<=1		<=1	<=0.25	<=0.5
<=0.25	<=1		>16	>16	8		>8	<=0.25	>2
<=0.25	<=1	1	<=4	4	<=1	32	<=1	<=0.25	<=0.5

2	8	0.5	>16	>16	>32	32	>8	0.5	>2
<=0.25	8	0.5	<=4	>16	>32	16	>8	<=0.25	>2
<=0.25	<=1		<=4	8	<=1		<=1	<=0.25	<=0.5
<=0.25	<=1	0.5	>16	16	4	32	<=1	<=0.25	>2
<=0.25	<=1	1	<=4	4	<=1	32	<=1	<=0.25	<=0.5
<=0.25	<=1	0.5	<=4	8	<=1	32	<=1	<=0.25	<=0.5
<=0.25	<=1	8	<=4	8	<=1	128	<=1	<=0.25	>2
<=0.25	<=1	0.5	<=4	8	<=1	32	<=1	<=0.25	>2
<=0.25	<=1	<=0.25	<=4	4	<=1	32	<=1	<=0.25	>2
<=0.25	<=1	2	<=4	4	<=1	32	<=1	<=0.25	<=0.5
<=0.25	<=1	0.5	<=4	4	<=1	32	<=1	<=0.25	<=0.5
<=0.25	<=1		<=4	4	<=1		<=1	<=0.25	<=0.5
<=0.25	<=1		<=4	8	<=1		<=1	<=0.25	<=0.5
<=0.25	<=1		16	8	<=1		<=1	<=0.25	>2
<=0.25	<=1		16	8	<=1		<=1	<=0.25	>2
<=0.25	<=1		<=4	4	<=1		2	<=0.25	<=0.5
<=0.25	<=1		<=4	4	<=1		<=1	<=0.25	<=0.5
<=0.25	<=1		<=4	4	<=1		>8	<=0.25	<=0.5
<=0.25	<=1		<=4	<=2	<=1		<=1	<=0.25	<=0.5
<=0.25	>16		>16	>16	>32		>8	<=0.25	>2
<=0.25	<=1		>16	>16	16		<=1	<=0.25	>2
<=0.25	<=1		<=4	4	<=1		<=1	<=0.25	>2
<=0.25	<=1		<=4	4	<=1		<=1	<=0.25	<=0.5
<=0.25	<=1	0.5	<=4	4	<=1	32	<=1	<=0.25	<=0.5
<=0.25	>16	1	>16	>16	>32	16	>8	<=0.25	>2
<=0.25	<=1	1	<=4	8	<=1	32	>8	<=0.25	<=0.5
<=0.25	<=1	1	8	16	<=1	32	>8	<=0.25	<=0.5
<=0.25	<=1	<=0.25	<=4	4	<=1	32	<=1	<=0.25	<=0.5
<=0.25	<=1		8	8	<=1		<=1	<=0.25	>2
<=0.25	<=1	2	<=4	4	<=1	32	<=1	<=0.25	<=0.5
<=0.25	<=1	0.5	8	8	<=1	16	>8	<=0.25	>2
<=0.25	<=1	16	>16	>16	4	4	<=1	<=0.25	<=0.5
<=0.25	<=1	1	<=4	4	<=1	256	<=1	<=0.25	>2
<=0.25	<=1	0.5	<=4	8	<=1	32	<=1	<=0.25	<=0.5
<=0.25	<=1	<=0.25	<=4	<=2	<=1	32	<=1	<=0.25	<=0.5
<=0.25	>16	16	16	>16	>32	32	2	<=0.25	>2
<=0.25	<=1	2	<=4	4	<=1	32	<=1	<=0.25	<=0.5
<=0.25	<=1	1	<=4	4	<=1	16	<=1	<=0.25	>2
<=0.25	<=1	0.5	<=4	<=2	<=1	32	>8	<=0.25	<=0.5
<=0.25	<=1	1	<=4	<=2	<=1	32	<=1	<=0.25	<=0.5
<=0.25	<=1	0.5	<=4	4	<=1	32	<=1	<=0.25	>2
<=0.25	<=1	1	8	8	<=1	16	<=1	<=0.25	>2
<=0.25	<=1	1	<=4	16	<=1	32	<=1	<=0.25	>2
<=0.25	<=1	1	<=4	4	<=1	32	<=1	<=0.25	>2
<=0.25	4	1	>16	>16	>32	32	>8	<=0.25	>2
<=0.25	<=1	0.5	<=4	4	<=1	16	<=1	<=0.25	>2
<=0.25	<=1	4	>16	>16	16	8	>8	<=0.25	>2
<=0.25	<=1	2	<=4	4	<=1	16	<=1	<=0.25	>2
<=0.25	<=1	1	<=4	4	<=1	32	<=1	<=0.25	>2
<=0.25	<=1		<=4	4	<=1		<=1	<=0.25	<=0.5
<=0.25	<=1	1	<=4	<=2	<=1	16	<=1	<=0.25	>2
<=0.25	<=1	0.5	<=4	4	<=1	32	<=1	<=0.25	<=0.5
<=0.25	>16	2	16	>16	>32	16	2	<=0.25	>2
<=0.25	<=1	2	<=4	<=2	<=1	32	<=1	<=0.25	<=0.5
<=0.25	>16	2	8	>16	>32	32	<=1	<=0.25	<=0.5
<=0.25	8	0.5	<=4	>16	>32	16	<=1	<=0.25	>2
<=0.25	<=1	1	8	8	<=1	32	<=1	<=0.25	<=0.5
<=0.25	<=1		16	4	<=1		>8	<=0.25	>2
<=0.25	<=1	0.5	<=4	4	<=1	32	<=1	<=0.25	<=0.5
<=0.25	<=1	0.5	<=4	8	<=1	32	>8	<=0.25	<=0.5
<=0.25	<=1	1	8	8	<=1	16	>8	<=0.25	>2

<=0.25	<=1	1	<=4	4	<=1	16	<=1	<=0.25	<=0.5
<=0.25	<=1	2	8	8	<=1	32	<=1	<=0.25	>2
<=0.25	<=1	1	<=4	4	<=1	32	<=1	<=0.25	<=0.5
<=0.25	<=1	1	<=4	<=2	<=1	16	<=1	<=0.25	>2
<=0.25	>16	1	<=4	>16	>32	32	<=1	<=0.25	<=0.5
<=0.25	<=1	0.5	<=4	4	<=1	128	>8	<=0.25	>2
<=0.25	<=1	0.5	<=4	<=2	<=1	32	<=1	<=0.25	>2
<=0.25	<=1	2	8	8	<=1	32	<=1	<=0.25	>2
<=0.25	>16	2	16	>16	>32	32	>8	<=0.25	<=0.5
<=0.25	<=1		8	16	<=1		>8	<=0.25	<=0.5
<=0.25	<=1	1	8	8	<=1	128	<=1	<=0.25	<=0.5
<=0.25	<=1		8	8	<=1		<=1	<=0.25	<=0.5
<=0.25	<=1	0.5	<=4	4	<=1	32	>8	<=0.25	>2
<=0.25	<=1	0.5	8	4	<=1	32	>8	<=0.25	>2
<=0.25	<=1	2	<=4	8	<=1	16	<=1	<=0.25	>2
<=0.25	<=1	8	<=4	4	<=1	16	<=1	<=0.25	>2
<=0.25	<=1		8	8	<=1		<=1	<=0.25	<=0.5
<=0.25	<=1		8	8	<=1		<=1	<=0.25	<=0.5
<=0.25	<=1		<=4	4	<=1		<=1	<=0.25	<=0.5
<=0.25	<=1		8	8	<=1		<=1	<=0.25	1
<=0.25	<=1		<=4	4	<=1		<=1	<=0.25	<=0.5
<=0.25	<=1		<=4	<=2	<=1		<=1	<=0.25	<=0.5
<=0.25	<=1		<=4	4	<=1		<=1	<=0.25	>2
<=0.25	<=1		<=4	4	<=1		<=1	<=0.25	<=0.5
<=0.25	<=1		<=4	4	<=1		<=1	<=0.25	>2
<=0.25	<=1		<=4	4	<=1		<=1	<=0.25	<=0.5
<=0.25	<=1		<=4	<=2	<=1		<=1	<=0.25	>2
<=0.25	<=1		<=4	4	<=1		<=1	<=0.25	<=0.5
<=0.25	<=1		<=4	4	<=1		<=1	<=0.25	>2
<=0.25	<=1		>16	>16	8		>8	<=0.25	>2
<=0.25	<=1	1	<=4	4	<=1	32	<=1	<=0.25	>2
<=0.25	<=1		<=4	4	<=1		8	<=0.25	>2
<=0.25	<=1	2	<=4	4	<=1	16	<=1	<=0.25	>2
<=0.25	<=1	2	>16	>16	8	16	<=1	<=0.25	<=0.5
<=0.25	<=1	2	>16	>16	8	32	<=1	<=0.25	<=0.5
<=0.25	<=1	1	<=4	<=2	<=1	16	<=1	<=0.25	<=0.5
<=0.25	<=1		>16	>16	16		<=1	<=0.25	>2
<=0.25	<=1	1	<=4	4	<=1	32	<=1	<=0.25	<=0.5
<=0.25	<=1		<=4	8	<=1		<=1	<=0.25	>2
<=0.25	<=1	1	<=4	8	<=1	16	<=1	<=0.25	>2
<=0.25	<=1	1	<=4	4	<=1	32	>8	<=0.25	>2
<=0.25	<=1	0.5	<=4	4	<=1	32	<=1	<=0.25	>2
<=0.25	<=1	2	<=4	8	<=1	16	<=1	<=0.25	>2
<=0.25	<=1	0.5	8	4	<=1	32	<=1	<=0.25	<=0.5
<=0.25	<=1	1	<=4	<=2	<=1	16	>8	<=0.25	<=0.5
<=0.25	<=1		<=4	8	<=1		<=1	<=0.25	<=0.5
<=0.25	<=1		8	8	<=1		<=1	<=0.25	>2
<=0.25	<=1		<=4	4	<=1		<=1	<=0.25	>2
<=0.25	>16		16	>16	>32		<=1	<=0.25	>2
<=0.25	<=1		<=4	8	<=1		>8	<=0.25	>2
<=0.25	2		>16	>16	16		>8	<=0.25	<=0.5
<=0.25	<=1		<=4	4	<=1		<=1	<=0.25	<=0.5
<=0.25	<=1		<=4	8	<=1		>8	<=0.25	>2
<=0.25	<=1		<=4	4	<=1		2	<=0.25	<=0.5
<=0.25	<=1		<=4	<=2	<=1		<=1	<=0.25	<=0.5
<=0.25	<=1		<=4	4	<=1		<=1	<=0.25	<=0.5
<=0.25	<=1		8	8	<=1		>8	<=0.25	>2
<=0.25	<=1	<=0.25	8	<=2	<=1	16	4	<=0.25	>2
<=0.25	<=1	1	<=4	4	<=1	16	<=1	<=0.25	>2
<=0.25	<=1		<=4	4	<=1		<=1	<=0.25	<=0.5
<=0.25	<=1	0.5	8	8	<=1	32	8	<=0.25	>2

<=0.25	<=1	0.5	<=4	4	<=1	32	<=1	<=0.25	>2
<=0.25	<=1	8	<=4	<=2	<=1	64	>8	<=0.25	>2
<=0.25	<=1	1	<=4	4	<=1	32	<=1	<=0.25	<=0.5
<=0.25	<=1		<=4	8	<=1		<=1	<=0.25	>2
<=0.25	<=1	1	<=4	4	<=1	4	<=1	<=0.25	<=0.5
<=0.25	<=1	2	<=4	4	<=1	16	>8	<=0.25	<=0.5
<=0.25	<=1		<=4	4	<=1		<=1	<=0.25	<=0.5
<=0.25	<=1		<=4	4	<=1		>8	<=0.25	<=0.5
<=0.25	<=1		8	4	<=1		<=1	<=0.25	<=0.5
2	>16	1	>16	>16	>32	16	<=1	<=0.25	>2
<=0.25	>16	<=0.25	<=4	>16	>32	16	<=1	<=0.25	>2
<=0.25	<=1	0.5	<=4	8	<=1	16	<=1	<=0.25	<=0.5
<=0.25	<=1	1	8	8	<=1	64	<=1	<=0.25	>2
<=0.25	<=1		<=4	8	<=1		>8	<=0.25	>2
<=0.25	<=1		<=4	4	<=1		<=1	<=0.25	>2
<=0.25	<=1	1	<=4	4	<=1	16	<=1	<=0.25	<=0.5
<=0.25	<=1		8	4	<=1		<=1	<=0.25	>2
<=0.25	<=1		<=4	4	<=1		<=1	<=0.25	<=0.5
<=0.25	>16		16	>16	>32		>8	<=0.25	<=0.5
<=0.25	<=1		<=4	8	<=1		>8	<=0.25	>2
<=0.25	<=1		<=4	4	<=1		2	<=0.25	<=0.5
<=0.25	>16		>16	>16	>32		>8	<=0.25	>2
<=0.25	<=1		8	4	<=1		<=1	<=0.25	>2
<=0.25	<=1		<=4	4	<=1		<=1	<=0.25	>2
<=0.25	<=1		<=4	4	<=1		<=1	<=0.25	<=0.5
<=0.25	>16		16	>16	>32		>8	<=0.25	>2
<=0.25	<=1		<=4	4	<=1		<=1	<=0.25	<=0.5
<=0.25	<=1	0.5	<=4	<=2	<=1	32	<=1	<=0.25	<=0.5
<=0.25	<=1	0.5	<=4	8	<=1	32	<=1	<=0.25	>2
<=0.25	<=1		<=4	4	<=1		<=1	<=0.25	<=0.5
<=0.25	<=1	<=0.25	<=4	<=2	<=1	32	<=1	<=0.25	<=0.5
<=0.25	<=1	0.5	<=4	8	<=1	16	<=1	<=0.25	>2
<=0.25	<=1	0.5	<=4	<=2	<=1	16	>8	0.5	<=0.5
<=0.25	<=1	1	<=4	8	<=1	32	>8	<=0.25	>2
<=0.25	<=1	1	<=4	<=2	<=1	32	<=1	<=0.25	<=0.5
<=0.25	<=1	1	>16	>16	8	64	<=1	<=0.25	>2
<=0.25	<=1	0.5	8	8	<=1	16	<=1	<=0.25	<=0.5
<=0.25	<=1	1	<=4	<=2	<=1	16	<=1	<=0.25	>2
<=0.25	<=1	1	<=4	4	<=1	16	<=1	<=0.25	>2
<=0.25	<=1	1	<=4	4	<=1	32	<=1	<=0.25	<=0.5
<=0.25	<=1	0.5	>16	16	<=1	32	<=1	<=0.25	<=0.5
<=0.25	2	1	>16	>16	>32	8	8	<=0.25	>2
<=0.25	<=1	1	8	4	<=1	16	<=1	<=0.25	<=0.5
<=0.25	<=1	16	8	4	<=1	16	<=1	<=0.25	<=0.5
<=0.25	<=1	1	<=4	8	<=1	16	>8	<=0.25	>2
<=0.25	4	2	<=4	>16	>32	32	<=1	<=0.25	>2
<=0.25	<=1	0.5	>16	>16	16	16	2	<=0.25	<=0.5
<=0.25	<=1	0.5	<=4	4	<=1	32	<=1	<=0.25	>2
<=0.25	<=1	4	<=4	8	<=1	32	<=1	<=0.25	<=0.5
<=0.25	<=1		<=4	4	<=1		<=1	<=0.25	<=0.5
<=0.25	<=1		<=4	4	<=1		<=1	<=0.25	<=0.5
<=0.25	<=1		>16	>16	32		>8	<=0.25	>2
<=0.25	<=1	1	<=4	<=2	<=1	32	<=1	<=0.25	<=0.5
<=0.25	<=1		>16	16	<=1		>8	<=0.25	>2
<=0.25	<=1	0.5	>16	8	<=1	16	>8	<=0.25	<=0.5
<=0.25	<=1	1	8	4	<=1	16	>8	<=0.25	>2
<=0.25	<=1	8	>16	16	<=1	64	<=1	<=0.25	1
<=0.25	8		8	>16	>32		<=1	<=0.25	>2
<=0.25	<=1		<=4	4	<=1		<=1	<=0.25	<=0.5
<=0.25	8	1	8	>16	>32	16	>8	<=0.25	>2
<=0.25	<=1	0.5	<=4	4	<=1	16	>8	<=0.25	<=0.5

<=0.25	<=1	2	>16	>16	32	64	<=1	<=0.25		<=0.5
<=0.25	<=1		8	8	<=1		<=1	<=0.25		<=0.5
<=0.25	<=1	1	<=4	4	<=1	16	<=1	<=0.25		<=0.5
<=0.25	<=1	1	<=4	4	<=1	32	<=1	<=0.25		>2
<=0.25	<=1	2	<=4	4	<=1	32	<=1	<=0.25		<=0.5
<=0.25	<=1		<=4	<=2	<=1		<=1	<=0.25		>2
<=0.25	<=1	64	<=4	8	<=1	64	<=1	<=0.25		>2
<=0.25	<=1	0.5	<=4	4	<=1	32	<=1	<=0.25		>2
<=0.25	<=1	1	<=4	4	<=1	32	<=1	<=0.25		<=0.5
<=0.25	>16		<=4	>16	>32		>8	<=0.25		>2
<=0.25	>16		8	>16	>32		>8	<=0.25		>2
<=0.25	<=1		>16	>16	8		>8	<=0.25		>2
<=0.25	<=1		8	4	<=1		>8	<=0.25		>2
<=0.25	4		>16	>16	<=1		<=1	<=0.25		>2
<=0.25	>16		8	>16	>32		>8	<=0.25		<=0.5
<=0.25	8		<=4	>16	>32		>8	<=0.25		>2
<=0.25	<=1		<=4	4	<=1		>8	<=0.25		>2
<=0.25	<=1		<=4	4	<=1		<=1	<=0.25		>2
<=0.25	<=1		<=4	4	<=1		<=1	<=0.25		<=0.5
<=0.25	<=1		<=4	4	<=1		<=1	<=0.25		>2
<=0.25	4		8	>16	>32		>8	<=0.25		>2
<=0.25	>16		<=4	>16	>32		<=1	<=0.25		<=0.5
<=0.25	>16		<=4	>16	>32		>8	<=0.25		>2
<=0.25	<=1		8	4	<=1		<=1	<=0.25		>2
<=0.25	<=1	1	<=4	<=2	<=1	32	<=1	<=0.25		<=0.5
<=0.25	<=1	0.5	<=4	4	<=1	32	<=1	<=0.25		>2
<=0.25	<=1	0.5	<=4	<=2	<=1	16	<=1	<=0.25		<=0.5
<=0.25	<=1		<=4	<=2	<=1		<=1	<=0.25		>2
<=0.25	<=1	1	>16	>16	4	256	>8	<=0.25		>2
<=0.25	<=1	1	<=4	<=2	<=1	32	<=1	<=0.25		>2
<=0.25	<=1	4	8	4	<=1	16	>8	<=0.25		>2
<=0.25	<=1	0.5	16	8	<=1	64	<=1	<=0.25		>2
<=0.25	>16		<=4	>16	>32		>8	<=0.25		>2
<=0.25	>16		>16	>16	>32		>8	<=0.25		>2
<=0.25	<=1		<=4	4	<=1		<=1	<=0.25		<=0.5
<=0.25	>16		<=4	>16	>32		>8	<=0.25		>2
<=0.25	<=1	4	<=4	<=2	<=1	32	<=1	<=0.25		<=0.5
<=0.25	<=1	1	<=4	4	<=1	16	<=1	<=0.25		>2
<=0.25	<=1	0.5	<=4	4	<=1	16	<=1	<=0.25		<=0.5
<=0.25	<=1	0.5	<=4	4	<=1	32	>8	<=0.25		<=0.5
<=0.25	<=1	0.5	<=4	4	<=1	32	<=1	<=0.25		<=0.5
<=0.25	<=1	1	16	8	<=1	32	<=1	<=0.25		<=0.5
<=0.25	<=1	0.5	<=4	4	<=1	32	<=1	<=0.25		<=0.5
<=0.25	<=1	0.5	8	8	<=1	32	<=1	<=0.25		<=0.5
<=0.25	16	1	8	>16	>32	16	<=1	<=0.25		<=0.5
<=0.25	<=1		8	8	<=1		>8	<=0.25		<=0.5
<=0.25	<=1	1	<=4	4	<=1	16	<=1	<=0.25		>2
<=0.25	<=1	1	8	8	<=1	32	>8	0.5		>2
<=0.25	2	4	>16	>16	32	16	>8	<=0.25		>2
<=0.25	<=1	1	<=4	4	<=1	32	<=1	<=0.25		<=0.5
<=0.25	<=1	0.5	<=4	4	<=1	16	>8	<=0.25		<=0.5
<=0.25	<=1	4	8	8	<=1	128	>8	<=0.25		>2
<=0.25	<=1	0.5	8	8	<=1	32	<=1	<=0.25		<=0.5
<=0.25	<=1	0.5	<=4	4	<=1	32	<=1	<=0.25		<=0.5
<=0.25	<=1	32	<=4	4	<=1	32	<=1	<=0.25		<=0.5
<=0.25	<=1	0.5	<=4	4	<=1	32	<=1	<=0.25		<=0.5
<=0.25	<=1	1	<=4	<=2	<=1	32	<=1	<=0.25		<=0.5
<=0.25	2		8	>16	16		2	<=0.25		>2
<=0.25	<=1		<=4	4	<=1		<=1	<=0.25		>2
<=0.25	<=1		<=4	4	<=1		<=1	<=0.25		>2
<=0.25	<=1		8	8	<=1		<=1	<=0.25		>2

<=0.25	<=1		8	4	<=1		2	<=0.25	<=0.5
<=0.25	<=1		<=4	4	<=1		<=1	<=0.25	<=0.5
<=0.25	<=1		<=4	8	<=1		>8	<=0.25	<=0.5
<=0.25	<=1		<=4	<=2	<=1		>8	<=0.25	>2
<=0.25	<=1		<=4	4	<=1		<=1	<=0.25	1
<=0.25	<=1	1	8	8	<=1	32	<=1	<=0.25	<=0.5
<=0.25	<=1	0.5	<=4	8	<=1	16	<=1	<=0.25	>2
<=0.25	<=1	0.5	8	8	<=1	32	<=1	<=0.25	>2
<=0.25	<=1	1	8	4	<=1	32	<=1	<=0.25	>2
<=0.25	<=1	0.5	<=4	4	<=1	32	<=1	<=0.25	<=0.5
<=0.25	<=1	0.5	<=4	8	<=1	32	<=1	<=0.25	<=0.5
<=0.25	<=1	0.5	<=4	<=2	<=1	32	<=1	<=0.25	<=0.5
<=0.25	<=1	0.5	<=4	4	<=1	16	2	<=0.25	<=0.5
<=0.25	4	2	16	>16	>32	4	<=1	<=0.25	>2
<=0.25	<=1	1	<=4	8	<=1	16	<=1	<=0.25	<=0.5
<=0.25	<=1	0.5	<=4	<=2	<=1	32	2	<=0.25	<=0.5
<=0.25	<=1	0.5	16	16	<=1	64	2	<=0.25	>2
<=0.25	>16	0.5	<=4	>16	>32	32	>8	<=0.25	>2
<=0.25	<=1	1	<=4	4	<=1	16	<=1	<=0.25	>2
<=0.25	<=1	1	<=4	4	<=1	16	<=1	<=0.25	<=0.5
<=0.25	<=1	1	<=4	4	<=1	16	<=1	<=0.25	>2
<=0.25	<=1	0.5	<=4	4	<=1	32	8	<=0.25	>2
<=0.25	<=1	1	<=4	4	<=1	32	<=1	<=0.25	<=0.5
<=0.25	<=1	0.5	>16	16	<=1	32	>8	<=0.25	>2
<=0.25	<=1	1	<=4	4	<=1	16	<=1	<=0.25	<=0.5
<=0.25	<=1	2	<=4	4	<=1	16	<=1	<=0.25	<=0.5
<=0.25	<=1	0.5	16	8	<=1	32	<=1	<=0.25	<=0.5
<=0.25	<=1	0.5	<=4	8	<=1	32	<=1	<=0.25	<=0.5
<=0.25	4	0.5	8	>16	>32	16	>8	<=0.25	<=0.5
<=0.25	>16	2	8	>16	>32	32	>8	<=0.25	>2
<=0.25	<=1		<=4	8	<=1		2	<=0.25	>2
<=0.25	<=1	1	<=4	4	<=1	16	2	<=0.25	<=0.5
<=0.25	<=1	8	<=4	4	<=1	32	<=1	<=0.25	<=0.5
<=0.25	<=1	8	>16	>16	<=1	64	>8	<=0.25	>2
<=0.25	<=1	2	<=4	8	<=1	32	2	<=0.25	>2
<=0.25	<=1		<=4	8	<=1		<=1	<=0.25	<=0.5
<=0.25	<=1	0.5	<=4	8	<=1	32	<=1	<=0.25	>2
<=0.25	<=1		<=4	4	<=1		<=1	<=0.25	>2
<=0.25	<=1	0.5	<=4	<=2	<=1	32	<=1	<=0.25	<=0.5
<=0.25	<=1		<=4	8	<=1		>8	<=0.25	>2
<=0.25	<=1		<=4	4	<=1		<=1	<=0.25	>2
<=0.25	<=1		<=4	4	<=1		<=1	<=0.25	>2
<=0.25	<=1		16	8	<=1		>8	<=0.25	<=0.5
<=0.25	<=1	1	<=4	<=2	<=1	16	<=1	<=0.25	<=0.5
<=0.25	<=1		>16	>16	8		>8	<=0.25	>2
<=0.25	<=1		<=4	<=2	<=1		<=1	<=0.25	>2
0.5	2		>16	>16	32		<=1	1	>2
<=0.25	4		<=4	>16	>32		<=1	<=0.25	>2
<=0.25	<=1		<=4	8	<=1		4	<=0.25	<=0.5
<=0.25	4	2	<=4	>16	>32	32	>8	<=0.25	<=0.5
<=0.25	<=1	1	<=4	<=2	<=1	32	<=1	<=0.25	>2
<=0.25	<=1	0.5	<=4	8	<=1	32	<=1	<=0.25	<=0.5
<=0.25	<=1	1	<=4	4	<=1	16	<=1	<=0.25	<=0.5
<=0.25	<=1	0.5	<=4	4	<=1	32	<=1	<=0.25	<=0.5
<=0.25	<=1	1	<=4	4	<=1	16	<=1	<=0.25	<=0.5
<=0.25	<=1		<=4	8	<=1		<=1	<=0.25	>2
<=0.25	<=1	2	<=4	<=2	<=1	32	<=1	<=0.25	>2
<=0.25	<=1	1	8	8	<=1	32	>8	<=0.25	>2
<=0.25	16	4	<=4	>16	>32	16	>8	<=0.25	<=0.5
<=0.25	<=1	0.5	<=4	4	<=1	32	<=1	<=0.25	<=0.5
<=0.25	<=1	0.5	<=4	4	<=1	32	<=1	<=0.25	>2

<=0.25	<=1	1	16	4	<=1	32	<=1	<=0.25	<=0.5
<=0.25	<=1	0.5	<=4	4	<=1	32	>8	<=0.25	>2
<=0.25	<=1	<=0.25	<=4	<=2	<=1	16	<=1	<=0.25	>2
<=0.25	<=1	0.5	<=4	4	<=1	32	<=1	<=0.25	<=0.5
<=0.25	<=1	0.5	>16	>16	8	32	>8	<=0.25	>2
<=0.25	<=1	0.5	>16	>16	8	16	>8	<=0.25	>2
<=0.25	<=1	0.5	<=4	8	<=1	32	<=1	<=0.25	<=0.5
<=0.25	<=1	0.5	<=4	4	<=1	32	<=1	<=0.25	>2
<=0.25	>16	1	8	>16	>32	32	>8	<=0.25	>2
<=0.25	<=1	1	<=4	4	<=1	32	4	<=0.25	<=0.5
<=0.25	<=1		16	>16	<=1		8	<=0.25	>2
<=0.25	<=1		<=4	4	<=1		<=1	<=0.25	>2
<=0.25	<=1		<=4	<=2	<=1		>8	<=0.25	<=0.5
<=0.25	<=1		<=4	<=2	<=1		<=1	<=0.25	<=0.5
<=0.25	<=1		<=4	4	<=1		<=1	<=0.25	<=0.5
<=0.25	<=1		<=4	8	<=1		<=1	<=0.25	<=0.5
<=0.25	<=1		<=4	<=2	<=1		<=1	<=0.25	<=0.5
<=0.25	<=1		>16	16	8		<=1	<=0.25	<=0.5
<=0.25	<=1		8	8	<=1		>8	<=0.25	<=0.5
<=0.25	8		<=4	>16	>32		<=1	<=0.25	>2
<=0.25	<=1		<=4	4	<=1		8	<=0.25	<=0.5
<=0.25	<=1		<=4	4	<=1		<=1	<=0.25	<=0.5
<=0.25	<=1		<=4	4	<=1		<=1	<=0.25	>2
<=0.25	<=1		<=4	4	<=1		<=1	<=0.25	>2
<=0.25	<=1		<=4	8	<=1		<=1	<=0.25	<=0.5
<=0.25	>16		16	>16	>32		<=1	<=0.25	>2
<=0.25	<=1	1	<=4	4	<=1	32	>8	<=0.25	>2
<=0.25	<=1	1	<=4	4	<=1	16	<=1	<=0.25	<=0.5
<=0.25	<=1	0.5	<=4	8	<=1	32	<=1	<=0.25	<=0.5
<=0.25	<=1	1	16	16	<=1	32	<=1	<=0.25	>2
<=0.25	<=1	2	<=4	4	<=1	32	<=1	<=0.25	<=0.5
<=0.25	<=1	2	<=4	4	<=1	32	<=1	<=0.25	<=0.5
<=0.25	<=1	0.5	<=4	4	<=1	32	<=1	<=0.25	>2
<=0.25	<=1	64	<=4	4	<=1	32	<=1	<=0.25	<=0.5
<=0.25	<=1	8	<=4	8	<=1	32	<=1	<=0.25	<=0.5
<=0.25	<=1	0.5	8	4	<=1	32	<=1	<=0.25	<=0.5
<=0.25	<=1	2	<=4	4	<=1	32	>8	<=0.25	>2
<=0.25	<=1	0.5	<=4	4	<=1	32	<=1	<=0.25	<=0.5
<=0.25	<=1	1	<=4	4	<=1	32	8	<=0.25	<=0.5
<=0.25	<=1	1	8	4	<=1	16	<=1	<=0.25	>2
<=0.25	<=1	1	<=4	4	<=1	16	<=1	<=0.25	>2
<=0.25	<=1	0.5	<=4	<=2	<=1	32	<=1	<=0.25	<=0.5
<=0.25	<=1	0.5	<=4	8	<=1	32	<=1	<=0.25	<=0.5
<=0.25	<=1	1	<=4	4	<=1	32	<=1	<=0.25	<=0.5
<=0.25	<=1	1	<=4	4	<=1	32	<=1	<=0.25	<=0.5
<=0.25	<=1	1	<=4	4	<=1	32	<=1	<=0.25	>2
<=0.25	<=1	0.5	8	4	<=1	32	<=1	<=0.25	<=0.5
<=0.25	<=1	1	8	4	<=1	16	<=1	<=0.25	<=0.5
<=0.25	<=1	1	<=4	<=2	<=1	8	<=1	<=0.25	<=0.5
<=0.25	<=1	1	>16	>16	16	32	8	<=0.25	>2
<=0.25	<=1	1	<=4	4	<=1	32	<=1	<=0.25	<=0.5
<=0.25	<=1	0.5	<=4	4	<=1	32	>8	<=0.25	>2
<=0.25	<=1	0.5	<=4	4	<=1	16	>8	<=0.25	>2
<=0.25	<=1	2	<=4	4	<=1	32	<=1	<=0.25	>2
<=0.25	<=1	0.5	<=4	<=2	<=1	32	2	<=0.25	<=0.5
<=0.25	<=1	1	<=4	<=2	<=1	16	<=1	<=0.25	<=0.5
<=0.25	<=1		<=4	8	<=1		>8	<=0.25	>2
<=0.25	<=1		<=4	4	<=1		<=1	<=0.25	<=0.5
<=0.25	<=1		8	4	<=1		<=1	<=0.25	<=0.5
<=0.25	<=1	1	8	8	<=1	16	>8	<=0.25	>2
2	>16	2	>16	>16	>32	32	>8	<=0.25	>2

<=0.25	>16	16	16	>16	>32	16	>8	<=0.25	>2
<=0.25	>16	0.5	>16	>16	>32	128	<=1	<=0.25	>2
<=0.25	<=1	1	8	8	<=1	32	>8	<=0.25	>2
<=0.25	<=1	1	16	8	<=1	32	<=1	<=0.25	<=0.5
<=0.25	<=1	1	<=4	4	<=1	64	<=1	<=0.25	>2
<=0.25	<=1	1	8	8	<=1	32	>8	<=0.25	>2
<=0.25	<=1	1	<=4	4	<=1	32	<=1	<=0.25	<=0.5
<=0.25	<=1	0.5	<=4	8	<=1	32	<=1	<=0.25	>2
<=0.25	<=1	0.5	<=4	8	<=1	32	<=1	<=0.25	<=0.5
<=0.25	<=1	0.5	<=4	4	<=1	32	<=1	<=0.25	<=0.5
<=0.25	<=1	1	8	8	<=1	32	<=1	<=0.25	<=0.5
<=0.25	<=1	1	<=4	4	<=1	32	<=1	<=0.25	>2
<=0.25	<=1	0.5	<=4	<=2	<=1	32	<=1	<=0.25	<=0.5
4	16		>16	>16	>32		<=1	4	<=0.5
<=0.25	<=1		<=4	4	<=1		<=1	<=0.25	<=0.5
<=0.25	>16		8	>16	>32		<=1	<=0.25	<=0.5
<=0.25	<=1		>16	16	8		<=1	<=0.25	>2
<=0.25	<=1		<=4	8	<=1		<=1	<=0.25	>2
<=0.25	<=1		<=4	>16	8		<=1	<=0.25	>2
<=0.25	<=1		8	8	<=1		<=1	<=0.25	>2
<=0.25	<=1		<=4	4	<=1		<=1	<=0.25	>2
<=0.25	<=1		<=4	4	<=1		<=1	<=0.25	>2
<=0.25	<=1		<=4	8	<=1		<=1	<=0.25	>2
<=0.25	<=1		16	16	<=1		4	<=0.25	1
<=0.25	<=1		<=4	8	<=1		<=1	<=0.25	<=0.5
<=0.25	<=1		<=4	8	<=1		4	<=0.25	>2
<=0.25	<=1		<=4	4	<=1		<=1	<=0.25	>2
<=0.25	<=1		>16	>16	8		<=1	<=0.25	<=0.5
<=0.25	<=1		<=4	8	<=1		>8	<=0.25	>2
<=0.25	<=1		<=4	4	<=1		2	<=0.25	>2
<=0.25	<=1		<=4	4	<=1		<=1	<=0.25	<=0.5
<=0.25	<=1		<=4	8	<=1		>8	<=0.25	>2
<=0.25	<=1		>16	>16	4		>8	<=0.25	>2
<=0.25	<=1		16	16	<=1		<=1	<=0.25	>2
<=0.25	<=1		<=4	<=2	<=1		<=1	<=0.25	<=0.5
<=0.25	<=1		<=4	4	<=1		<=1	<=0.25	<=0.5
<=0.25	<=1		>16	>16	16		>8	<=0.25	>2
<=0.25	<=1		8	16	<=1		<=1	<=0.25	<=0.5
<=0.25	<=1	0.5	<=4	<=2	<=1	16	4	<=0.25	>2
<=0.25	<=1	<=0.25	<=4	4	<=1	32	2	<=0.25	<=0.5
<=0.25	<=1	2	<=4	4	<=1	32	<=1	<=0.25	>2
<=0.25	<=1	0.5	<=4	4	<=1	16	<=1	<=0.25	<=0.5
<=0.25	>16	0.5	>16	>16	>32	32	<=1	<=0.25	>2
<=0.25	<=1	0.5	<=4	<=2	<=1	32	>8	<=0.25	>2
<=0.25	<=1	4	8	8	<=1	64	<=1	<=0.25	<=0.5
<=0.25	<=1	1	<=4	16	<=1	32	<=1	<=0.25	<=0.5
<=0.25	<=1	1	<=4	4	<=1	16	<=1	<=0.25	<=0.5
<=0.25	<=1	0.5	<=4	<=2	<=1	32	<=1	<=0.25	<=0.5
<=0.25	<=1	1	<=4	4	<=1	32	<=1	<=0.25	<=0.5
<=0.25	>16		8	>16	>32		>8	<=0.25	>2
<=0.25	<=1	0.5	<=4	8	<=1	16	<=1	<=0.25	<=0.5
<=0.25	<=1	0.5	8	8	<=1	32	>8	<=0.25	>2
<=0.25	<=1		<=4	4	<=1		<=1	<=0.25	<=0.5
<=0.25	<=1		8	4	<=1		<=1	<=0.25	<=0.5
<=0.25	<=1		<=4	4	<=1		<=1	<=0.25	<=0.5
<=0.25	<=1		<=4	8	<=1		8	<=0.25	>2
<=0.25	<=1		<=4	4	<=1		>8	<=0.25	>2
<=0.25	<=1		<=4	4	<=1		<=1	<=0.25	>2
<=0.25	8		<=4	>16	>32		>8	<=0.25	<=0.5
<=0.25	<=1		<=4	4	<=1		<=1	<=0.25	<=0.5
<=0.25	<=1		<=4	4	<=1		<=1	<=0.25	>2

<=0.25	<=1		8	4	<=1		<=1	<=0.25	<=0.5
<=0.25	<=1	1	<=4	4	<=1	32	<=1	<=0.25	<=0.5
<=0.25	<=1		<=4	4	<=1		<=1	<=0.25	<=0.5
<=0.25	<=1	1	<=4	4	<=1	128	<=1	<=0.25	<=0.5
<=0.25	<=1	8	<=4	4	<=1	32	>8	<=0.25	>2
<=0.25	<=1	1	<=4	<=2	<=1	32	<=1	<=0.25	<=0.5
<=0.25	<=1	0.5	<=4	4	<=1	32	<=1	<=0.25	<=0.5
<=0.25	<=1	2	>16	>16	32	16	>8	<=0.25	>2
<=0.25	<=1	0.5	<=4	8	<=1	32	<=1	<=0.25	<=0.5
<=0.25	<=1	2	<=4	4	<=1	<=4	<=1	<=0.25	<=0.5
<=0.25	<=1		<=4	4	<=1		<=1	<=0.25	<=0.5
<=0.25	<=1	1	8	8	<=1	16	<=1	<=0.25	<=0.5
<=0.25	<=1	0.5	<=4	4	<=1	32	<=1	<=0.25	>2
<=0.25	<=1	1	16	8	<=1	16	<=1	<=0.25	<=0.5
<=0.25	<=1	0.5	8	8	<=1	16	<=1	<=0.25	<=0.5
<=0.25	<=1	1	8	8	<=1	128	2	<=0.25	>2
<=0.25	<=1	2	<=4	4	<=1	32	<=1	<=0.25	>2
<=0.25	<=1	1	>16	>16	32	32	>8	<=0.25	>2
<=0.25	<=1	1	<=4	4	<=1	32	<=1	<=0.25	1
<=0.25	<=1	1	<=4	<=2	<=1	16	<=1	<=0.25	>2
<=0.25	<=1	0.5	<=4	4	<=1	32	>8	<=0.25	>2
<=0.25	<=1		<=4	4	<=1		<=1	<=0.25	>2
<=0.25	<=1		<=4	4	<=1		<=1	<=0.25	<=0.5
<=0.25	<=1		<=4	4	<=1		<=1	<=0.25	<=0.5
<=0.25	>16		8	>16	>32		>8	<=0.25	>2
<=0.25	<=1		<=4	8	<=1		<=1	<=0.25	<=0.5
<=0.25	<=1		<=4	4	<=1		<=1	<=0.25	<=0.5
<=0.25	<=1		<=4	4	<=1		<=1	<=0.25	<=0.5
<=0.25	<=1		<=4	8	<=1		<=1	<=0.25	>2
<=0.25	<=1		<=4	4	<=1		2	<=0.25	<=0.5
<=0.25	<=1		<=4	4	<=1		<=1	<=0.25	<=0.5
<=0.25	<=1		<=4	8	<=1		2	<=0.25	<=0.5
<=0.25	<=1		<=4	8	<=1		<=1	<=0.25	>2
<=0.25	<=1		<=4	<=2	<=1		2	<=0.25	<=0.5
<=0.25	<=1		16	8	<=1		>8	<=0.25	>2
<=0.25	<=1		<=4	<=2	<=1		>8	<=0.25	>2
<=0.25	<=1		<=4	4	<=1		<=1	<=0.25	>2
<=0.25	<=1		<=4	4	<=1		>8	<=0.25	>2
<=0.25	<=1		>16	>16	8		>8	<=0.25	>2
<=0.25	<=1		8	8	<=1		>8	<=0.25	>2
<=0.25	<=1		<=4	4	<=1		<=1	<=0.25	<=0.5
<=0.25	<=1		<=4	4	<=1		<=1	<=0.25	>2
<=0.25	<=1		<=4	4	<=1		<=1	<=0.25	>2
<=0.25	<=1		<=4	8	<=1		<=1	<=0.25	>2
<=0.25	<=1		8	4	<=1		<=1	<=0.25	>2
<=0.25	<=1		<=4	8	<=1		<=1	<=0.25	<=0.5
<=0.25	8		<=4	>16	>32		>8	<=0.25	>2
<=0.25	2		>16	>16	>32		>8	0.5	>2
<=0.25	<=1		8	4	<=1		<=1	<=0.25	>2
<=0.25	<=1		<=4	4	<=1		<=1	<=0.25	>2
<=0.25	<=1		<=4	<=2	<=1		>8	<=0.25	>2
<=0.25	<=1		8	4	<=1		>8	<=0.25	>2
<=0.25	<=1		>16	>16	8		<=1	<=0.25	>2
<=0.25	<=1		<=4	8	<=1		<=1	<=0.25	<=0.5
<=0.25	<=1		<=4	4	<=1		<=1	<=0.25	>2
<=0.25	<=1		<=4	<=2	<=1		<=1	<=0.25	>2
<=0.25	<=1		<=4	4	<=1		<=1	<=0.25	>2
<=0.25	<=1		8	8	<=1		<=1	<=0.25	<=0.5
<=0.25	8		<=4	>16	>32		>8	<=0.25	2
<=0.25	<=1		8	4	<=1		<=1	<=0.25	<=0.5
<=0.25	<=1		<=4	4	<=1		<=1	<=0.25	<=0.5

<=0.25	<=1		>16	>16	32		>8	<=0.25		>2
<=0.25	<=1		8	4	<=1		>8	<=0.25		<=0.5
<=0.25	<=1		8	8	<=1		<=1	<=0.25		>2
<=0.25	<=1		<=4	4	<=1		<=1	<=0.25		>2
<=0.25	<=1		<=4	4	<=1		2	<=0.25		<=0.5
<=0.25	<=1		>16	>16	8		<=1	<=0.25		>2
<=0.25	>16		<=4	>16	>32		<=1	<=0.25		<=0.5
<=0.25	<=1		<=4	<=2	<=1		<=1	<=0.25		<=0.5
<=0.25	<=1		<=4	4	<=1		<=1	<=0.25		>2
<=0.25	<=1		<=4	4	<=1		<=1	<=0.25		<=0.5

TCY_M	TGC_M	TZP_M	ESBL Phenotypic Confirmatory test	ESBL PCR result	AmpC PCR result -	ESBL/AmpC PCR Status
<=1						
>8			Pos	SHV	Neg	Pos/Neg
>8			Neg	Neg	CMY	Neg/Pos
>8						
>8						
2						
>8						
2						
>8						
>8						
>8						
<=1						
>8						
>8						
>8						
2						
>8						
2						
>8						
>8						
2						
2						
>8						
>8						
2			Neg	Neg	CMY	Neg/Pos
2						
>8						
>8						
2						
>8						
>8						
>8						
>8						
>8						
<=1						
>8						
>8						
<=1						
>8						
4						
2						
4						
>8						
>8			Pos	CTX-M, SHV	Neg	Pos/Neg
>8						
>8						
>8						
>8						
4						
2						
>8						
>8						
2						
>8						
>8						

>8	Neg	SHV	CMY, DHA	Pos/Pos
>8				
4				
>8				
<=1				
2				
>8				
2				
>8				
>8				
>8				
>8				
2				
>8				
>8				
>8				
2				
2				
>8				
>8				
>8				
2				
>8				
<=1				
2				
>8				
>8				
>8				
>8	Neg	Neg	CMY	Neg/Pos
<=1				
>8				
<=1				
>8				
>8				
2				
>8				
2				
2				
>8				
>8				
8				
>8				
>8				
>8				
>8				
<=1				
2				
>8				
>8				
4	Pos	CTX-M	Neg	Pos/Neg
>8				
>8				
>8				
>8				
>8				
>8				
2				
2				
>8				
2	Neg	Neg	CMY	Neg/Pos
2				

2				
>8				
4				
>8				
>8				
>8	Pos	CTX-M	Neg	Pos/Neg
>8	Pos	CTX-M	Neg	Pos/Neg
>8				
8	Neg	Neg	CMY	Neg/Pos
8	Neg	Neg	CMY	Neg/Pos
2				
>8				
>8				
>8				
2				
2				
>8				
>8				
>8				
>8				
2				
>8				
>8				
>8	Pos	CTX-M	Neg	Pos/Neg
>8				
>8				
>8				
>8				
>8				
>8				
>8				
>8				
>8	Neg	Neg	CMY	Neg/Pos
>8				
>8				
2				
2				
>8				
>8				
>8				
>8				
>8				
2				
>8				
>8				
2				
>8				
>8				
>8				
2				
>8				
>8	Pos	CTX-M	Neg	Pos/Neg
>8				
>8	Neg	Neg	Neg	Neg/Neg
>8				
>8				
>8				
>8				
>8				
>8				
>8	Neg	CTX-M	Neg	Pos/Neg

>8				
>8				
>8				
2				
>8				
>8				
>8				
>8				
2				
>8				
<=1				
>8				
2				
>8				
>8				
>8				
>8				
>8				
2				
>8				
2				
>8	Neg	Neg	CMY	Neg/Pos
>8				
4				
>8				
>8				
2				
2	Neg	Neg	CMY	Neg/Pos
2				
4				
>8				
8				
>8				
2				
>8				
>8				
>8				
>8				
>8				
>8	Neg	Neg	CMY	Neg/Pos
2				
>8				
>8				
2	Neg	Neg	CMY	Neg/Pos
2				
>8				
2				
2				
2				
>8				
>8	Pos	CTX-M	Neg	Pos/Neg
>8				
>8				
>8				
>8				
2				
>8				
>8				
>8				
>8				
>8				

4				
>8				
>8				
>8				
<=1				
>8	Neg	Neg	CMY	Neg/Pos
2				
>8				
>8				
>8				
>8				
>8				
2				
4				
>8				
>8				
4				
>8				
>8				
4				
>8				
>8				
2				
>8				
2				
<=1				
2				
>8				
>8				
>8				
2				
2				
>8				
>8				
>8				
4				
>8				
4				
2				
>8				
2	Pos	SHV	Neg	Pos/Neg
4				
>8				
>8				
2				
>8				
>8				
>8				
>8				
>8				
4				
>8				
>8				
2				
>8				
>8	Pos	CTX-M	Neg	Pos/Neg
>8	Pos	CTX-M	Neg	Pos/Neg
2	Pos	CTX-M	Neg	Pos/Neg
2				
2				
>8				

2				
2				
2				
4				
>8				
>8				
>8	Neg	Neg	CMY	Neg/Pos
>8				
>8				
2				
>8	Neg	Neg	CMY	Neg/Pos
>8	Neg	Neg	CMY	Neg/Pos
<=1				
>8				
2				
>8				
4				
>8				
2				
2				
>8				
>8				
2				
>8	Pos	CTX-M	Neg	Pos/Neg
>8				
<=1				
>8				
2				
2				
2				
>8				
4				
4				
>8				
>8				
>8				
>8				
>8				
2				
2				
>8	Neg	Neg	Neg	Neg/Neg
>8				
>8				
>8				
>8				
>8				
>8				
4				
>8				
>8				
>8				
>8				
>8				
>8	Pos	SHV	Neg	Pos/Neg
2				
>8				
<=1				
>8				
2				
2				
>8				

>8				
>8				
>8				
>8				
>8				
>8				
>8				
2				
>8				
>8				
>8				
>8				
2				
>8				
>8				
>8	Pos	CTX-M	Neg	Pos/Neg
>8				
>8				
>8				
2				
>8				
>8				
>8				
2				
>8				
4				
>8				
2				
>8	Pos	CTX-M	Neg	Pos/Neg
>8				
2				
>8				
>8				
>8				
2				
>8				
<=1				
>8				
>8				
>8				
>8				
4				
4				
4				
>8				
>8				
>8				
4				
>8				
>8				
>8				
>8				
>8				
<=1				
>8				
>8				
<=1				
4				
8				
>8				
>8				

>8				
>8				
>8				
2				
>8				
<=1				
>8				
>8				
>8				
2				
2				
>8				
<=1				
>8				
4				
>8				
>8				
>8	Neg	Neg	Neg	Neg/Neg
2				
>8				
>8				
>8	Neg	Neg	CMY	Neg/Pos
2				
2				
>8	Neg	Neg	Neg	Neg/Neg
>8				
>8				
>8	Neg	Neg	CMY	Neg/Pos
>8				
>8				
>8				
>8				
>8				
>8	Neg	Neg	DHA	Neg/Pos
>8	Neg	Neg	Neg	Neg/Neg
>8				
>8				
2				
2				
>8				
>8				
>8	Neg	CTX-M	CMY	Pos/Pos
>8				
>8	Neg	Neg	Neg	Neg/Neg
4				
>8	Pos	CTX-M	CMY	Pos/Pos
4				
4				
>8				
<=1				
<=1				
>8				
<=1	Pos	CTX-M	Neg	Pos/Neg
>8				
>8				
2				
<=1				
>8				
<=1				
>8				
2				

>8	Neg	Neg	CMY	Neg/Pos
2				
>8				
>8				
>8	Neg	Neg	CMY	Neg/Pos
2				
>8				
>8				
>8				
>8				
<=1				
4				
4				
2				
>8				
>8				
2				
>8				
2				
>8	Pos	CTX-M	Neg	Pos/Neg
>8				
>8				
>8	Pos	CTX-M	Neg	Pos/Neg
2				
2				
2				
<=1				
>8				
2				
>8				
>8				
>8				
>8				
>8				
2	Neg	Neg	CMY	Neg/Pos
>8				
>8				
>8				
>8				
4				
>8				
>8				
>8				
4				
>8				
>8				
>8				
2				
>8				
>8				
>8				
>8				
>8				
>8				
>8				
>8				
2				
>8				
2				
>8				
>8				

4				
>8				
>8				
>8				
>8				
>8				
>8				
>8				
>8				
>8				
2				
4				
>8				
>8				
>8				
>8	Neg	Neg	CMY	Neg/Pos
2				
2				
>8				
>8				
<=1				
8				
>8	Pos	CTX-M	Neg	Pos/Neg
>8				
>8				
2				
>8				
2	Neg	Neg	CMY	Neg/Pos
2				
>8				
>8				
2				
>8				
4				
>8				
2				
<=1				
2				
>8	Pos	Neg	Neg	Neg/Neg
>8				
>8				
>8				
>8				
2				
2				
>8				
>8				
>8	Neg	Neg	Neg	Neg/Neg
2				
8				
>8				
>8				
>8				
>8				
>8				
2				
>8				
>8				
4				
>8				
>8				

2				
>8				
>8				
>8				
2				
>8				
>8				
<=1				
>8				
>8				
>8				
2				
2				
>8				
>8				
>8				
2				
>8				
<=1				
>8				
>8	Pos	Neg	CMY	Neg/Pos
>8				
>8				
2				
<=1				
4				
2				
>8	Neg	Neg	Neg	Neg/Neg
2				
>8				
2				
2				
>8				
>8				
>8				
>8				
2				
2				
>8				
>8				
>8				
>8	Neg	Neg	CMY	Neg/Pos
>8				
>8	Neg	Neg	CMY	Neg/Pos
<=1				
4				
>8				
2				
2				
>8				
>8				
4				
2				
>8				
>8				
>8				
>8				
>8				
4				
>8				
>8	Pos	CTX-M	Neg	Pos/Neg

<=1				
>8	Pos	CTX-M	CMY	Pos/Pos
>8	Neg	Neg	CMY	Neg/Pos
>8				
4				
>8				
2				
>8	Neg	Neg	CMY	Neg/Pos
>8	Pos	CTX-M	Neg	Pos/Neg
>8				
>8	Pos	CTX-M	Neg	Pos/Neg
2				
<=1				
<=1				
4				
<=1				
>8				
<=1				
>8				
2	Pos	SHV	CMY	Pos/Pos
<=1				
>8				
2				
>8				
2				
>8				
>8				
2				
4				
2				
>8				
2				
>8				
2				
>8				
>8	Neg	CTX-M	CMY	Pos/Pos
>8				
2				
>8				
<=1				
>8				
>8				
>8				
<=1				
2				
>8				
>8				
<=1				
2				
>8				
>8				
2				
>8				
2				
>8				
>8				
<=1				
>8				
2				
4				
4				

>8				
2				
>8				
>8				
4				
>8				
>8				
2				
>8				
>8				
>8				
>8				
4				
<=1				
4				
>8				
2				
>8				
>8				
>8				
>8				
>8	Pos	CTX-M	Neg	Pos/Neg
>8				
>8				
>8				
2	Neg	Neg	CMY	Neg/Pos
2				
>8				
>8				
<=1				
4				
>8	Pos	CTX-M	Neg	Pos/Neg
>8				
2				
>8				
>8				
>8				
>8				
2				
>8				
4				
2				
2				
<=1				
>8				
2				
<=1				
>8				
2				
>8				
>8				
2				
>8				
>8				
>8				
2				
>8	Neg	Neg	CMY	Neg/Pos
<=1				
2				
<=1				
<=1				

>8				
>8				
2				
>8				
2				
2				
2				
>8				
2	Neg	Neg	CMY	Neg/Pos
>8				
<=1				
2				
>8				
>8				
>8	Neg	Neg	CMY	Neg/Pos
>8				
>8				
>8				
>8	Pos	CTX-M	CMY	Pos/Pos
>8				
2				
>8				
>8				
>8				
4				
>8				
2				
>8	Neg	Neg	CMY	Neg/Pos
<=1				
2				
>8	Pos	CTX-M	CMY	Pos/Pos
>8	Neg	Neg	CMY	Neg/Pos
>8				
>8				
2				
2				
>8				
<=1				
>8				
>8				
>8				
<=1				
>8				
2				
2				
>8				
>8				
2				
>8				
2				
>8				
<=1				
>8				
2				
>8				
>8				
2				
2				
>8				
2				
<=1				

>8				
>8	Neg	Neg	CMY	Neg/Pos
>8				
>8	Pos	CTX-M	Neg	Pos/Neg
2				
>8	Neg	Neg	CMY	Neg/Pos
<=1				
2				
>8				
>8				
>8				
>8				
>8				
2				
>8				
>8	Neg	Neg	Neg	Neg/Neg
2				
>8				
>8				
>8				
>8				
>8				
<=1				
8	Neg	Neg	CMY	Neg/Pos
>8				
>8				
2				
>8	Neg	Neg	CMY	Neg/Pos
<=1	Neg	Neg	CMY	Neg/Pos
4				
>8				
4				
2				
>8				
2				
4				
>8				
>8				
>8				
>8				
>8				
>8				
>8				
>8				
<=1				
>8				
>8				
>8				
>8				
<=1				
>8				
>8				
>8				
>8				
>8				
>8				
>8				
<=1				
>8				
2				
>8				

>8				
4				
2				
<=1				
>8				
<=1				
>8				
>8				
2				
2				
>8				
>8				
>8				
>8				
2				
>8				
>8				
<=1				
<=1				
>8				
>8	Neg	Neg	CMY	Neg/Pos
<=1				
>8				
>8				
>8				
>8	Neg	Neg	CMY	Neg/Pos
2	Neg	Neg	CMY	Neg/Pos
>8				
>8				
<=1				
<=1				
>8				
>8	Neg	Neg	CMY	Neg/Pos
>8				
>8				
>8				
>8				
>8				
>8				
>8				
2				
<=1				
<=1				
>8	Neg	Neg	CMY	Neg/Pos
>8				
>8				
2				
>8				
>8				
2				
2				
>8				
>8				
<=1				
2	Neg	Neg	CMY	Neg/Pos
>8				
<=1				
>8				
4				
>8				
2				

>8					
>8					
2					
2					
>8					
2					
2					
>8					
<=1					
>8	<=8				
>8	<=8				
>8	<=8				
2	<=8				
>8	<=8				
>8	<=8				
2	<=8				
4	<=8				
2	<=8				
<=1	<=8				
>8	<=8				
2	16				
2	<=8				
>8	<=8	Neg	Neg	CMY	Neg/Pos
>8	<=8				
>8	<=8				
<=1	<=8				
>8	<=8				
>8	<=8				
>8	<=8				
>8	<=8				
>8	<=8				
>8	<=8				
2	<=8				
2	<=8				
>8					
>8					
2					
>8					
2					
2					
>8					
>8					
>8					
>8		Neg	Neg	Neg	Neg/Neg
2					
2		Neg	Neg	Neg	Neg/Neg
>8					
>8					
2					
<=1					
>8					
>8					
>8					
4					
2					
2					
>8					
>8					
2					
2					
>8					

>8					
2					
>8	<=8				
2	<=8				
2	<=8				
>8	<=8				
2	<=8				
<=1	<=8				
>8	<=8				
>8	<=8				
2	<=8				
>8	<=8				
2	<=8				
>8	<=8				
>8	<=8				
2	<=8				
>8	<=8				
>8	<=8				
>8	<=8				
>8	<=8				
>8	<=8				
2	<=8				
>8	<=8				
>8	<=8				
>8	<=8				
>8					
>8					
>8					
>8		Neg	Neg	CMY	Neg/Pos
2					
>8					
>8					
>8					
>8					
>8					
<=1					
<=1					
>8	<=8				
>8					
>8		Pos	CTX-M	Neg	Pos/Neg
>8		Neg	Neg	CMY	Neg/Pos
<=1					
2					
>8					
2					
>8					
>8					
>8					
2					
<=1					
<=1					
<=1					
>8		Neg	Neg	CMY	Neg/Pos
2					
>8					
2					
4		Neg	Neg	CMY	Neg/Pos
>8					
<=1	<=8				
4					
>8					

2					
2					
>8					
4	<=8				
<=1					
>8					
<=1					
<=1					
<=1					
2					
>8	<=8				
>8	<=8				
2	<=8				
<=1	<=8				
2	<=8				
>8	<=8	Neg	Neg	CMY	Neg/Pos
2	<=8				
>8	<=8				
>8	<=8	Neg	Neg	CMY	Neg/Pos
>8	<=8				
<=1	>64				
4	<=8				
>8	<=8	Neg	Neg	Neg	Neg/Neg
2	<=8				
2	<=8				
2	<=8				
>8	<=8				
>8	<=8				
>8	<=8				
>8					
>8					
2					
>8					
>8					
>8					
>8					
>8					
>8					
>8					
>8					
>8					
<=1					
<=1					
2					
>8					
>8					
>8					
>8					
<=1					
2		Neg	Neg	CMY	Neg/Pos
>8					
>8					
2					
>8					
>8					
2					
2					
>8		Neg	Neg	Neg	Neg/Neg
4					
<=1					
<=1					

>8					
>8					
>8					
>8					
<=1					
>8					
>8					
2					
>8					
2	<=8				
2	<=8				
2					
>8					
<=1					
<=1					
>8					
>8					
>8					
>8					
>8		Neg	Neg	Neg	Neg/Neg
2					
>8					
>8		Pos	CTX-M	Neg	Pos/Neg
2					
2					
>8					
>8		Pos	CTX-M	Neg	Pos/Neg
<=1					
>8					
>8					
>8					
>8					
>8					
>8					
2					
2					
>8					
2					
2					
>8					
>8					
<=1					
>8					
>8					
>8					
>8	<=8				
2	<=8				
2	>64	Neg	Neg	Neg	Neg/Neg
<=1	<=8				
2	<=8				
2	<=8				
4	<=8				
2	>64				
<=1	<=8				
>8	<=8				
>8	<=8				
>8	<=8				
>8	<=8				
>8	<=8	Neg	Neg	CMY	Neg/Pos
>8	<=8	Neg	Neg	Neg	Neg/Neg
<=1		Neg	Neg	CMY	Neg/Pos

>8		Pos	CTX-M	Neg	Pos/Neg
4		Neg	CTX-M	CMY	Pos/Pos
<=1					
>8		Neg	Neg	CMY	Neg/Pos
2		Neg	Neg	CMY, DHA	Neg/Pos
2					
2					
>8		Neg	Neg	CMY	Neg/Pos
>8		Neg	Neg	Neg	Neg/Neg
2					
>8		Neg	Neg	CMY	Neg/Pos
>8					
>8					
>8					
>8					
>8		Pos	CTX-M	CMY	Pos/Pos
>8		Pos	CTX-M	CMY	Pos/Pos
2		Neg	Neg	Neg	Neg/Neg
>8					
2					
<=1					
2					
>8					
>8		Pos	CTX-M	Neg	Pos/Neg
>8					
<=1	<=8				
>8	<=8				
>8	<=8				
>8	<=8	Neg	Neg	CMY	Neg/Pos
>8	>64	Neg	Neg	CMY	Neg/Pos
>8	<=8				
>8	<=8				
>8	<=8				
4	<=8				
>8	<=8				
>8	<=8				
2	<=8				
4	<=8				
>8	<=8				
<=1	<=8				
>8	16	Neg	Neg	CMY	Neg/Pos
>8	<=8				
>8	<=8	Neg	Neg	CMY	Neg/Pos
>8	16	Neg	Neg	CMY	Neg/Pos
>8	<=8				
>8	<=8				
2	<=8				
4	<=8				
>8	<=8				
4	<=8				
2	<=8				
>8	<=8				
>8	<=8				
4	<=8				
2	<=8				
4	<=8				
>8	<=8				
>8	16				
>8	<=8				
>8	<=8				
4	<=8				

>8	<=8				
>8	<=8				
>8	>64	Pos	CTX-M	Neg	Pos/Neg
2	<=8				
4	<=8	Neg	Neg	CMY	Neg/Pos
2	<=8				
>8	<=8				
2	<=8				
>8	<=8				
4	<=8				
2	<=8				
>8	<=8				
2	<=8				
<=1	<=8				
>8	<=8				
4	<=8				
2					
>8		Neg	Neg	CMY	Neg/Pos
4					
>8		Neg	Neg	CMY	Neg/Pos
>8					
>8					
>8					
>8					
>8					
>8		Neg	Neg	CMY	Neg/Pos
>8					
2					
2	<=8				
>8	<=8	Neg	Neg	CMY	Neg/Pos
>8	<=8				
<=1	<=8				
2	<=8				
>8	16				
<=1	<=8				
>8	<=8				
2	<=8				
<=1	<=8				
>8	>64	Pos	CTX-M	CMY	Pos/Pos
>8	<=8				
<=1	<=8				
>8	<=8				
>8	<=8	Pos	CTX-M	Neg	Pos/Neg
2	<=8				
>8	<=8				
<=1	<=8				
2	<=8				
>8	<=8				
>8	<=8				
>8	<=8				
>8	<=8				
>8	<=8				
>8	<=8	Neg	Neg	CMY	Neg/Pos
>8	>64				
<=1	<=8				
>8	<=8				
>8	<=8				
>8	<=8				
>8	<=8				
2	<=8				
>8	<=8				

>8	<=8				
>8	<=8				
>8	<=8				
>8	<=8				
<=1	<=8				
>8	<=8	Pos	CTX-M	Neg	Pos/Neg
>8	<=8				
>8	<=8				
2					
<=1	<=8				
4	<=8				
>8	<=8				
2	<=8				
>8	<=8				
<=1	<=8				
>8	<=8				
>8	<=8				
>8	64	Pos	CTX-M	CMY	Pos/Pos
>8	<=8				
>8	<=8				
>8	<=8				
<=1	32				
2	64	Neg	Neg	CMY	Neg/Pos
<=1	16	Neg	Neg	Neg	Neg/Neg
>8	<=8				
4	<=8				
<=1	<=8				
2	<=8				
>8	<=8				
>8	64	Neg	Neg	Neg	Neg/Neg
>8	>64				
2	<=8				
>8	<=8				
8	<=8				
>8	<=8				
>8	<=8				
>8					
>8					
>8					
>8					
>8					
2					
2					
>8		Neg	Neg	CMY	Neg/Pos
>8		Neg	Neg	CMY	Neg/Pos
2					
>8					
>8					
2					
2					
>8					
>8					
4					
2					
>8					
>8					
2					
2					
>8					
>8					
<=1					

>8					
>8					
>8		Pos	CTX-M	Neg	Pos/Neg
4					
2					
>8					
2					
4					
>8					
>8					
>8					
<=1					
>8					
>8					
>8					
>8	<=8				
4	<=8	Neg	Neg	CMY	Neg/Pos
>8	<=8				
<=1	32	Neg	Neg	Neg	Neg/Neg
<=1	<=8				
>8	<=8				
>8	<=8				
4	<=8				
>8	<=8				
>8	<=8	Pos	CTX-M	Neg	Pos/Neg
2	<=8				
2	<=8				
2	<=8				
>8	<=8				
>8	<=8				
>8	<=8				
2	<=8				
2	<=8				
2	<=8				
2	<=8				
2	<=8				
2	<=8				
>8	<=8	Pos	CTX-M	Neg	Pos/Neg
>8	<=8				
>8	<=8	Pos	CTX-M	Neg	Pos/Neg
2	<=8				
>8	<=8	Pos	CTX-M	Neg	Pos/Neg
>8	<=8				
>8	<=8				
>8	<=8				
2	<=8				
8	<=8				
>8	<=8	Pos	CTX-M	Neg	Pos/Neg
>8	<=8	Pos	Neg	CMY	Neg/Pos
>8	<=8	Neg	Neg	CMY	Neg/Pos
>8	<=8	Pos	CTX-M	Neg	Pos/Neg
>8	32	Pos	CTX-M	CMY	Pos/Pos
<=1	<=8				
2	<=8				
2	<=8				
2	<=8				
2	<=8				
2	<=8				
<=1	<=8				
>8	<=8				
2	<=8				

>8	<=8				
>8	<=8				
2	<=8				
2	<=8				
4	<=8	Neg	CTX-M	CMY	Pos/Pos
>8	<=8				
>8	<=8				
2	<=8	Neg	Neg	CMY	Neg/Pos
<=1	<=8				
2	<=8				
>8	<=8				
2	<=8				
>8	16	Neg	Neg	Neg	Neg/Neg
>8	<=8				
2	<=8				
2	<=8	Neg	Neg	CMY	Neg/Pos
>8	<=8	Pos	CTX-M	Neg	Pos/Neg
>8	<=8				
>8	<=8				
>8	<=8				
>8	<=8				
2	<=8				
>8	<=8	Neg	Neg	CMY	Neg/Pos
4	<=8				
>8	<=8				
>8	<=8				
2	<=8				
2	<=8				
>8	16				
2	<=8				
2	<=8				
>8	<=8				
>8	<=8				
>8	<=8				
2	32				
>8	16	Neg	Neg	CMY	Neg/Pos
>8	<=8				
2	<=8				
2	<=8				
2	<=8				
>8	<=8				
>8	<=8				
2	<=8				
>8	<=8	Neg	Neg	CMY	Neg/Pos
>8	<=8				
>8	>64	Neg	Neg	CMY	Neg/Pos
>8	<=8				
>8	32	Neg	Neg	CMY	Neg/Pos
>8	<=8	Pos	CTX-M	Neg	Pos/Neg
>8	<=8				
2	16				
2	<=8				
2	<=8				
>8	<=8				
4	<=8				
2	<=8				
>8	<=8				
>8	<=8				
2	<=8				
>8	<=8				
>8	<=8				

>8	<=8				
2	<=8	Neg	Neg	CMY	Neg/Pos
4	<=8				
<=1	<=8				
4	<=8				
>8	<=8				
2	<=8				
>8	64	Neg	Neg	CMY	Neg/Pos
>8	<=8	Pos	CTX-M	Neg	Pos/Neg
2	<=8				
>8	16				
2	<=8				
>8	<=8				
4	<=8				
>8	<=8				
>8	<=8				
>8	<=8	Neg	Neg	CMY	Neg/Pos
4	<=8				
>8	<=8				
>8	<=8	Neg	Neg	CMY	Neg/Pos
>8	<=8				
>8	<=8				
>8	<=8				
>8	<=8				
>8	<=8				
>8	<=8	Neg	Neg	CMY	Neg/Pos
2	<=8				
>8	<=8				
>8	<=8	Neg	Neg	CMY	Neg/Pos
>8	<=8				
>8	<=8				
>8	>64	Neg	Neg	CMY	Neg/Pos
4	<=8				
>8	16	Pos	CTX-M	CMY	Pos/Pos
>8	<=8	Pos	CTX-M	Neg	Pos/Neg
>8	<=8	Pos	CTX-M	Neg	Pos/Neg
>8	<=8				
>8	<=8				
>8	<=8				
>8	<=8				
2	<=8				
>8	<=8				
>8	<=8	Neg	Neg	CMY	Neg/Pos
2	<=8				
>8	<=8				
>8	<=8				
4	<=8				
>8	<=8				
>8	<=8				
2	<=8				
>8	<=8	Neg	Neg	CMY	Neg/Pos
>8	<=8				
>8	<=8				
>8	<=8				
>8	<=8				
>8	<=8				
2	<=8				
>8	<=8				
>8	<=8				
2	<=8				
2	<=8				

2	<=8				
4	<=8				
>8	<=8				
>8	<=8				
>8	<=8				
>8	<=8				
>8	<=8				
>8	<=8				
>8	<=8				
>8	<=8				
4	<=8				
>8	<=8	Pos	CTX-M	Neg	Pos/Neg
>8	<=8				
4	<=8				
>8	<=8				
>8	<=8				
>8	<=8				
>8	<=8				
2	<=8				
>8	<=8				
<=1	<=8				
2	<=8				
>8	16	Pos	CTX-M	Neg	Pos/Neg
2	<=8				
2	<=8				
>8	<=8				
2	<=8				
2	<=8				
>8	<=8				
<=1	<=8				
>8	<=8				
>8	<=8				
>8	<=8				
>8	<=8	Neg	Neg	CMY	Neg/Pos
>8	<=8				
>8	<=8				
>8	<=8				
>8	<=8	Neg	SHV	Neg	Pos/Neg
2	<=8				
>8	<=8				
>8	<=8				
>8	<=8				
>8	<=8				
>8	<=8				
2	64	Neg	Neg	CMY	Neg/Pos
2	<=8				
>8	<=8	Neg	Neg	Neg	Neg/Neg
>8	<=8				
>8	<=8	Neg	Neg	CMY	Neg/Pos
>8	<=8				
2	<=8				
4	16	Pos	CTX-M	Neg	Pos/Neg
>8	<=8				
>8	<=8				
2	<=8				
4	<=8				
>8	<=8				
2	<=8				
>8	<=8				
<=1	<=8				
>8	<=8				

<=1	<=8				
>8	<=8				
>8	32	Neg	Neg	CMY	Neg/Pos
>8	<=8				
2	<=8				
>8	<=8	Neg	Neg	DHA	Neg/Pos
2	<=8				
2	<=8				
>8	<=8				
>8	<=8				
>8	<=8				
>8	<=8				
>8	<=8	Neg	Neg	Neg	Neg/Neg
>8	>64	Neg	Neg	CMY	Neg/Pos
>8	<=8				
>8	16	Neg	Neg	Neg	Neg/Neg
2	16	Neg	Neg	CMY	Neg/Pos
2	<=8				
2	<=8				
>8	<=8				
2	>64	Pos	CTX-M	Neg	Pos/Neg
<=1	<=8	Neg	Neg	CMY	Neg/Pos
>8	<=8				
<=1	<=8				
2	<=8				
>8	<=8				
4	<=8				
>8	<=8				
2	<=8				
>8	<=8				
>8	<=8				
2	<=8				
>8	<=8				
>8	<=8				
>8	<=8				
4	<=8	Pos	CTX-M	Neg	Pos/Neg
>8	<=8				
>8	<=8				
>8	<=8				
2	<=8				
2	<=8				
>8	<=8				
>8	<=8				
>8	<=8				
2	<=8				
>8	<=8				
>8	>64				
>8	<=8				
>8	32	Neg	Neg	CMY	Neg/Pos
4	<=8				
>8	<=8				
2	<=8				
>8	<=8				
>8	<=8				
>8	<=8				
>8	<=8				
2	<=8				
>8	<=8				
2	<=8				
>8	<=8				
>8	<=8	Neg	Neg	Neg	Neg/Neg

2	<=8				
2	<=8				
>8	<=8				
>8	<=8				
>8	<=8				
>8	<=8				
>8	<=8				
<=1	<=8				
2	<=8				
>8	<=8				
2	<=8				
>8	<=8				
>8	<=8				
4	<=8				
<=1	<=8				
2	<=8				
2	<=8				
2	<=8				
2	<=8				
>8	32	Neg	Neg	CMY	Neg/Pos
>8	<=8				
>8	<=8				
>8	<=8				
4	<=8				
<=1	<=8				
>8	<=8				
<=1	<=8				
>8	<=8				
2	16	Pos	CTX-M	CMY	Pos/Pos
2	<=8				
>8	<=8				
>8	<=8				
>8	<=8				
>8	<=8				
>8	16	Neg	Neg	CMY	Neg/Pos
>8	<=8				
>8	<=8				
2	<=8				
>8	16	Pos	CTX-M	Neg	Pos/Neg
>8	<=8				
>8	<=8				
>8	<=8				
2	<=8				
>8	<=8				
>8	<=8				
2	<=8				
>8	<=8				
>8	<=8				
>8	<=8				
<=1	<=8				
>8	<=8				
>8	<=8				
4	<=8				
>8	<=8				
>8	<=8				
2	<=8				
>8	<=8				
>8	<=8	Neg	Neg	CMY	Neg/Pos
>8	<=8				
>8	<=8				
>8	<=8				

>8	<=8	Pos	CTX-M	Neg	Pos/Neg
2	>64	Neg	Neg	CMY	Neg/Pos
>8	<=8				
>8	<=8				
>8	<=8				
2	<=8				
>8	<=8				
2	<=8				
>8	<=8				
2	<=8				
2	<=8				
>8	<=8				
>8	<=8	Pos	CTX-M	Neg	Pos/Neg
2	<=8	Neg	Neg	Neg	Neg/Neg
2	<=8				
>8	<=8				
<=1	<=8				
2	<=8				
>8	<=8				
>8	<=8				
2	<=8				
>8	<=8	Neg	Neg	CMY	Neg/Pos
2	<=8				
>8	16	Neg	Neg	CMY	Neg/Pos
>8	<=8				
>8	<=8				
2	<=8				
>8	<=8				
2	<=8				
>8	<=8				
2	<=8				
2	<=8				
>8	<=8				
4	32	Neg	CTX-M	CMY, DHA	Pos/Pos
4	<=8				
8	<=8	Neg	CTX-M	CMY	Pos/Pos
>8	<=8				
2	<=8				
4	<=8	Neg	Neg	CMY, DHA	Neg/Pos
>8	<=8				
2	<=8				
2	<=8				
2	<=8				
>8	<=8				
>8	<=8				
>8	<=8				
>8	<=8				
<=1	<=8				
>8	<=8				
<=1	<=8				
>8	<=8				
>8	<=8	Pos	CTX-M	Neg	Pos/Neg
4	32				
<=1	<=8				
>8	<=8	Neg	Neg	DHA	Neg/Pos
>8	<=8	Neg	Neg	CMY	Neg/Pos
2	<=8				
<=1	<=8				
>8	<=8				
>8	<=8				
4	<=8				

2	<=8				
>8	<=8				
>8	<=8				
2	<=8				
<=1	<=8				
<=1	<=8				
>8	<=8				
>8	<=8				
>8	<=8				
>8	<=8	Neg	Neg	CMY	Neg/Pos
>8	<=8	Pos	CTX-M	Neg	Pos/Neg
2	<=8				
2	<=8				
2	<=8				
>8	<=8				
>8	<=8	Neg	Neg	Neg	Neg/Neg
<=1	<=8				
2	<=8				
2	32				
>8	<=8				
2	<=8				
>8	<=8				
2	<=8				
>8	<=8				
2	<=8				
2	<=8				
>8	<=8	Neg	Neg	CMY	Neg/Pos
8	<=8				
>8	<=8	Pos	CTX-M	Neg	Pos/Neg
>8	<=8				
>8	<=8				
>8	<=8	Neg	Neg	CMY	Neg/Pos
<=1	<=8				
>8	<=8				
<=1	<=8				
2	<=8				
>8	<=8				
>8	<=8	Neg	Neg	CMY	Neg/Pos
2	<=8				
>8	<=8				
2	<=8				
4	32	Neg	Neg	CMY	Neg/Pos
2	<=8				
>8	<=8	Neg	Neg	DHA	Neg/Pos
2	<=8				
>8	<=8				
>8	16				
>8	<=8				
>8	<=8				
>8	<=8				
2	<=8				
2	<=8				
2	<=8				
2	<=8				
2	<=8				
>8	<=8				
>8	<=8				
>8	<=8				
2	<=8				
2	<=8				
>8	<=8				

4	<=8	Pos	CTX-M	Neg	Pos/Neg
2	<=8				
>8	<=8				
<=1	<=8				
>8	<=8				
>8	<=8				
<=1	<=8				
2	<=8				
2	<=8				
4	<=8				
2	<=8				
>8	<=8				
>8	<=8				
>8	<=8				
>8	<=8				
2	<=8				
>8	64	Neg	Neg	CMY	Neg/Pos
>8	<=8				
>8	<=8	Neg	Neg	CMY	Neg/Pos
>8	<=8				
2	<=8				
>8	<=8				
>8	<=8				
>8	<=8				
2	<=8				
2	<=8				
2	<=8				
<=1	<=8				
>8	<=8				
>8	<=8				
2	<=8				
2	<=8				
>8	<=8				
>8	<=8	Neg	Neg	CMY	Neg/Pos
>8	<=8				
>8	<=8				
4	<=8				
2	<=8				
>8	<=8	Neg	Neg	CMY	Neg/Pos
2	<=8				
>8	<=8				
2	<=8				
>8	<=8				
>8	<=8				
2	<=8				
2	16				
2	<=8				
>8	<=8				
4	<=8				
2	<=8				
2	<=8				
4	<=8				
>8	<=8				
2	<=8				
>8	<=8				
2	<=8				
2	<=8				
>8	<=8				
>8	<=8				
2	<=8				
>8	<=8	Pos	Neg	CMY	Neg/Pos

>8	<=8				
>8	<=8				
>8	<=8				
2	<=8				
2	<=8				
<=1	<=8	Neg	Neg	CMY	Neg/Pos
2	<=8				
>8	<=8				
>8	<=8	Pos	CTX-M	Neg	Pos/Neg
>8	<=8	Neg	Neg	CMY	Neg/Pos
>8	<=8				
>8	>64				
>8	<=8				
4	<=8	Neg	Neg	CMY	Neg/Pos
>8	<=8	Neg	CTX-M	CMY	Pos/Pos
8	>64	Pos	CTX-M, SHV	Neg	Pos/Neg
<=1	<=8				
2	<=8				
4	16	Pos	CTX-M	Neg	Pos/Neg
4	<=8				
4	<=8				
2	<=8				
>8	<=8				
4	<=8	Neg	Neg	CMY	Neg/Pos
4	32	Neg	Neg	DHA	Neg/Pos
>8	<=8				
>8	<=8				
2	<=8				
2	<=8				
4	<=8				
>8	<=8				
>8	>64				
>8	<=8				
>8	64	Neg	CTX-M	CMY	Pos/Pos
4	<=8				
2	<=8				
>8	<=8				
4	<=8				
>8	<=8				
>8	<=8				
>8	<=8				
2	<=8				
2	<=8				
>8	<=8				
2	<=8				
2	<=8				
>8	<=8				
2	<=8				
2	<=8				
2	<=8				
4	<=8				
>8	<=8				
>8	<=8				
>8	32	Neg	Neg	CMY	Neg/Pos
<=1	<=8				
>8	<=8				
4	<=8				
>8	<=8				
2	<=8				
>8	<=8				
>8	<=8	Pos	SHV	Neg	Pos/Neg

2		<=8				
>8		16	Neg	Neg	CMY	Neg/Pos
>8		<=8				
>8		<=8				
2		<=8				
>8		<=8	Neg	Neg	CMY	Neg/Pos
>8		<=8				
2		<=8				
>8		<=8	Pos	CTX-M	Neg	Pos/Neg
>8		<=8				
>8		<=8				
>8		<=8				
2		<=8				
>8		<=8				
<=1		<=8				
>8		<=8				
4		<=8				
	<=0.25	<=4				
	<=0.25	<=4				
	<=0.25	<=4	Pos	CTX-M	Neg	Pos/Neg
	<=0.25	<=4				
	<=0.25	<=4				
	<=0.25	<=4				
	<=0.25	<=4				
	<=0.25	<=4	Neg	Neg	CMY	Neg/Pos
	<=0.25	<=4				
	<=0.25	<=4				
	<=0.25	<=4	Pos	CTX-M	Neg	Pos/Neg
	<=0.25	<=4	Neg	Neg	DHA	Neg/Pos
	<=0.25	8	Neg	Neg	CMY	Neg/Pos
	<=0.25	<=4				
	<=0.25	<=4				
	<=0.25	<=4				
	<=0.25	<=4				
	<=0.12	<=4				
	<=0.12	<=4				
	<=0.12	<=4				
	<=0.12	<=4				
	<=0.12	<=4				
	<=0.12	<=4				
	<=0.12	<=4	Neg	Neg	CMY	Neg/Pos
	<=0.12	8				
	<=0.12	<=4				
	<=0.12	<=4				
	<=0.12	<=4				
	<=0.12	<=4	Pos	CTX-M	Neg	Pos/Neg
	<=0.12	<=4				
	<=0.25	<=4				
	<=0.25	<=4				
	<=0.25	<=4				
	<=0.25	<=4				
	<=0.25	<=4				
	<=0.12	<=4	Pos	CTX-M	Neg	Pos/Neg
	<=0.12	<=4				
	0.25	<=4				
	<=0.12	<=4				
	<=0.25	<=4				
	<=0.25	<=4				
	<=0.25	<=4				
	<=0.25	<=4				
	<=0.25	<=4				

<=0.12	<=4	Neg	Neg	CMY	Neg/Pos
<=0.25	<=4				
<=0.25	<=4				
<=0.25	<=4	Pos	CTX-M	Neg	Pos/Neg
<=0.25	<=4				
<=0.25	<=4				
<=0.25	<=4				
0.25	64	Neg	Neg	CMY	Neg/Pos
<=0.12	<=4				
<=0.12	<=4				
<=0.12	<=4				
<=0.12	<=4				
<=0.12	<=4				
<=0.25	<=4	Neg	Neg	CMY	Neg/Pos
<=0.25	<=4				
<=0.25	<=4				
<=0.25	<=4	Neg	Neg	CMY	Neg/Pos
<=0.25	<=4				
<=0.25	<=4	Pos	CTX-M	Neg	Pos/Neg
<=0.25	<=4				
<=0.25	<=4				
<=0.25	<=4	Neg	Neg	CMY	Neg/Pos
<=0.25	<=4	Neg	Neg	CMY	Neg/Pos
<=0.25	<=4				
<=0.25	<=4				
<=0.25	<=4				
<=0.25	<=4				
<=0.25	>64	Neg	Neg	CMY	Neg/Pos
<=0.12	<=4				
<=0.12	<=4				
<=0.12	<=4				
0.5	<=4				
<=0.25	<=4				
<=0.25	<=4				
<=0.12	<=4	Neg	Neg	Neg	Neg/Neg
<=0.12	<=4				
<=0.12	<=4				
<=0.12	8				
<=0.12	<=4				
<=0.12	<=4				
<=0.25	<=4				
<=0.12	<=4				
<=0.12	<=4				
<=0.12	<=4				
<=0.12	<=4				
<=0.12	<=4				
<=0.25	<=4				
<=0.25	<=4	Pos	CTX-M	Neg	Pos/Neg
<=0.25	<=4				
<=0.25	<=4				
<=0.25	<=4				
<=0.25	<=4				
<=0.25	<=4				
<=0.12	<=4				
<=0.25	<=4				
<=0.25	<=4				
<=0.25	<=4				
<=0.12	<=4	Pos	CTX-M	Neg	Pos/Neg
<=0.12	<=4				
<=0.12	<=4				
0.25	<=4				

<=0.25	<=4				
<=0.25	<=4				
<=0.25	32	Pos	CTX-M	CMY	Pos/Pos
<=0.25	16	Pos	CTX-M	CMY	Pos/Pos
<=0.25	<=4				
<=0.25	<=4				
<=0.25	<=4				
<=0.12	<=4				
0.25	8	Neg	Neg	CMY	Neg/Pos
<=0.25	<=4				
<=0.25	<=4				
<=0.25	<=4				
<=0.25	<=4				
<=0.25	<=4				
<=0.12	16	Pos	CTX-M	CMY	Pos/Pos
<=0.25	<=4	Neg	Neg	CMY	Neg/Pos
<=0.25	<=4				
<=0.25	<=4				
<=0.12	8	Pos	CTX-M	Neg	Pos/Neg
<=0.25	<=4	Neg	Neg	CMY	Neg/Pos
<=0.25	<=4				
<=0.25	<=4				
<=0.25	<=4				
<=0.25	<=4				
<=0.12	<=4				
<=0.12	32	Neg	CTX-M	CMY	Pos/Pos
<=0.12	<=4				
<=0.25	32	Neg	Neg	CMY	Neg/Pos
<=0.25	<=4				
<=0.25	<=4	Neg	Neg	CMY	Neg/Pos
<=0.25	<=4				
<=0.25	<=4				
<=0.25	<=4				
<=0.25	<=4				
<=0.25	<=4	Pos	CTX-M	Neg	Pos/Neg
<=0.25	<=4	Neg	Neg	CMY	Neg/Pos
<=0.25	<=4				
0.5	<=4	Neg	Neg	Neg	Neg/Neg
<=0.25	<=4				
<=0.25	<=4				
<=0.25	<=4				
<=0.25	<=4	Neg	Neg	CMY	Neg/Pos
<=0.25	<=4				
<=0.25	<=4				
<=0.25	<=4				
<=0.25	<=4				
<=0.25	8	Pos	CTX-M	CMY	Pos/Pos
<=0.25	<=4				
<=0.25	<=4				
<=0.12	<=4				
<=0.12	<=4				
<=0.12	<=4				
<=0.12	<=4				
<=0.12	<=4				
0.25	<=4	Pos	CTX-M	CMY	Pos/Pos
<=0.25	8	Pos	CTX-M	Neg	Pos/Neg
<=0.12	16				
<=0.12	<=4				
<=0.12	<=4				
<=0.25	<=4	Pos	CTX-M	Neg	Pos/Neg
<=0.12	16				

<=0.25	<=4	Pos	CTX-M	Neg	Pos/Neg
<=0.25	8	Neg	Neg	CMY	Neg/Pos
<=0.25	<=4	Neg	Neg	CMY	Neg/Pos
<=0.12	<=4				
0.25	<=4				
<=0.12	<=4				
<=0.12	8	Neg	Neg	CMY	Neg/Pos
<=0.12	64	Neg	Neg	CMY	Neg/Pos
<=0.12	<=4	Neg	Neg	CMY	Neg/Pos
<=0.12	<=4				
<=0.12	<=4	Neg	Neg	CMY	Neg/Pos
0.25	<=4				
<=0.12	<=4				
<=0.12	8	Neg	Neg	CMY	Neg/Pos
<=0.12	<=4				
<=0.25	<=4				
<=0.25	<=4				
<=0.25	<=4				
<=0.25	<=4				
<=0.25	<=4				
<=0.25	<=4				
<=0.12	<=4				
<=0.25	<=4				
<=0.25	<=4				
<=0.25	<=4	Pos	CTX-M	Neg	Pos/Neg
<=0.25	<=4				
<=0.25	<=4				
<=0.25	<=4				
<=0.25	<=4				
<=0.25	<=4				
<=0.12	<=4				
<=0.25	<=4				
<=0.25	<=4				
0.5	<=4	Neg	Neg	Neg	Neg/Neg
<=0.25	<=4				
<=0.25	<=4				
<=0.25	<=4				
<=0.25	<=4				
<=0.25	<=4	Neg	Neg	Neg	Neg/Neg
<=0.25	<=4				
<=0.12	<=4				
<=0.12	<=4				
<=0.12	<=4				
<=0.25	<=4				
<=0.25	<=4				
<=0.25	<=4				
<=0.25	<=4				
<=0.25	<=4				
<=0.25	<=4				
<=0.25	>64	Neg	Neg	CMY	Neg/Pos
<=0.25	<=4				
<=0.25	<=4				
<=0.25	<=4				
<=0.25	<=4				
<=0.25	<=4				
<=0.25	32	Neg	Neg	CMY	Neg/Pos
<=0.25	<=4				
<=0.25	16				
<=0.25	<=4	Pos	CTX-M	Neg	Pos/Neg
<=0.25	<=4				
<=0.12	<=4				

<=0.12	<=4	Neg	Neg	CMY	Neg/Pos
<=0.12	<=4				
<=0.12	<=4				
<=0.12	<=4				
<=0.12	<=4				
<=0.25	<=4				
<=0.25	<=4				
<=0.25	<=4				
<=0.25	<=4				
<=0.25	<=4				
<=0.25	8	Neg	Neg	Neg	Neg/Neg
<=0.25	<=4				
<=0.25	<=4	Pos	CTX-M	Neg	Pos/Neg
<=0.25	<=4				
<=0.25	<=4				
<=0.12	<=4				
<=0.25	64	Neg	Neg	CMY	Neg/Pos
<=0.12	<=4				
<=0.12	<=4	Pos	CTX-M	CMY	Pos/Pos
<=0.25	<=4				
<=0.12	<=4				
<=0.12	<=4				
<=0.25	<=4				
<=0.25	16	Neg	Neg	Neg	Neg/Neg
<=0.25	<=4				
<=0.25	<=4				
<=0.12	<=4				
<=0.12	<=4	Neg	Neg	DHA	Neg/Pos
<=0.12	<=4				
<=0.12	<=4				
<=0.12	<=4				
<=0.12	<=4				
<=0.12	<=4				
<=0.12	<=4				
<=0.12	<=4				
<=0.12	<=4				
<=0.25	<=4				
<=0.25	<=4				
<=0.25	<=4				
<=0.25	<=4	Pos	CTX-M	Neg	Pos/Neg
0.25	<=4				
<=0.25	32	Neg	Neg	CMY	Neg/Pos
<=0.25	<=4				
<=0.25	<=4				
<=0.25	<=4				
<=0.25	<=4	Neg	Neg	Neg	Neg/Neg
<=0.25	<=4	Pos	CTX-M	Neg	Pos/Neg
<=0.25	<=4				
<=0.25	<=4	Neg	Neg	CMY	Neg/Pos
<=0.25	<=4				
<=0.12	<=4				
<=0.25	32	Neg	Neg	CMY	Neg/Pos
<=0.25	32	Pos	CTX-M	CMY	Pos/Pos
<=0.25	<=4				
<=0.12	<=4				
<=0.12	<=4	Neg	Neg	CMY	Neg/Pos
<=0.12	32	Pos	CTX-M	CMY	Pos/Pos
0.25	64	Pos	CTX-M	CMY	Pos/Pos
<=0.25	<=4				
<=0.25	<=4				
<=0.25	<=4				

<=0.25	<=4				
<=0.25	<=4				
<=0.25	<=4				
<=0.25	<=4				
<=0.25	<=4				
<=0.25	<=4				
<=0.25	<=4				
<=0.25	<=4				
<=0.25	<=4				
<=0.25	<=4				
<=0.25	<=4				
<=0.25	<=4				
<=0.25	<=4				
<=0.25	<=4				
<=0.25	<=4				
<=0.25	<=4				
<=0.25	8	Pos	CTX-M	Neg	Pos/Neg
<=0.25	<=4				
<=0.25	<=4				
<=0.12	<=4				
<=0.12	<=4				
<=0.12	<=4				
<=0.12	<=4				
<=0.12	<=4				
<=0.25	<=4				
<=0.12	<=4				
<=0.25	<=4				
<=0.25	<=4				
<=0.25	<=4				
<=0.25	<=4				
<=0.25	<=4				
<=0.25	<=4	Pos	Neg	CMY	Neg/Pos
<=0.25	<=4				
<=0.25	<=4	Neg	Neg	CMY	Neg/Pos
<=0.25	<=4				
<=0.25	<=4				
<=0.25	<=4				
<=0.25	<=4				
<=0.25	<=4				
<=0.25	<=4				
<=0.25	<=4				
<=0.25	<=4				
<=0.25	<=4				
<=0.25	<=4				
<=0.25	<=4				
<=0.25	<=4				
<=0.12	<=4				
<=0.25	<=4	Pos	CTX-M	Neg	Pos/Neg
<=0.25	8	Neg	Neg	CMY	Neg/Pos
<=0.25	16	Neg	Neg	CMY	Neg/Pos
<=0.25	<=4				
<=0.25	<=4				
<=0.12	<=4				
0.25	<=4				
<=0.12	8	Neg	Neg	CMY	Neg/Pos
<=0.12	<=4	Neg	Neg	CMY	Neg/Pos
<=0.25	<=4				
<=0.25	<=4				
<=0.25	<=4				
<=0.12	<=4				
<=0.12	<=4	Neg	Neg	CMY	Neg/Pos

<=0.12	<=4				
<=0.12	<=4				
<=0.25	<=4				
<=0.12	<=4				
<=0.12	<=4				
<=0.12	<=4				
<=0.12	<=4				
<=0.12	<=4				
<=0.12	<=4				
<=0.12	<=4				
<=0.12	<=4				
<=0.12	<=4				
<=0.12	8	Neg	Neg	CMY	Neg/Pos
<=0.12	8	Neg	Neg	CMY	Neg/Pos
<=0.12	<=4				
<=0.12	<=4				
<=0.12	<=4				
<=0.12	<=4				
<=0.25	8	Neg	Neg	CMY	Neg/Pos
<=0.25	<=4				
<=0.25	<=4				
<=0.25	<=4				
<=0.25	<=4				
<=0.25	<=4				
<=0.25	32	Pos	CTX-M	CMY	Pos/Pos
<=0.25	<=4				
<=0.25	<=4				
<=0.12	<=4				
<=0.25	<=4				
<=0.12	<=4				
0.25	8				
<=0.25	<=4				
<=0.12	<=4				
0.5	<=4				
<=0.12	<=4	Pos	CTX-M	Neg	Pos/Neg
0.25	<=4	Neg	Neg	CMY	Neg/Pos
0.25	<=4				
<=0.12	<=4				
<=0.25	<=4				
<=0.25	<=4				
<=0.25	8				
<=0.25	<=4	Neg	Neg	CMY	Neg/Pos
<=0.25	<=4	Pos	CTX-M	Neg	Pos/Neg
<=0.25	<=4				
<=0.25	16	Neg	CTX-M	CMY	Pos/Pos
<=0.25	8				
<=0.25	<=4				
<=0.25	<=4				
<=0.25	<=4	Neg	Neg	CMY	Neg/Pos
<=0.25	<=4	Neg	Neg	CMY	Neg/Pos
<=0.25	<=4				
<=0.25	<=4	Neg	Neg	DHA	Neg/Pos
<=0.25	<=4				
<=0.25	<=4				
<=0.25	<=4				
<=0.25	<=4				
<=0.25	<=4				
<=0.25	<=4				
<=0.25	<=4	Pos	CTX-M	Neg	Pos/Neg
<=0.25	16	Pos	CTX-M	CMY	Pos/Pos
<=0.25	<=4	Neg	Neg	CMY	Neg/Pos

<=0.25	<=4	Neg	Neg	CMY	Neg/Pos
<=0.25	<=4				
<=0.25	<=4				
0.5	8				
<=0.25	<=4				
<=0.25	<=4				
<=0.25	<=4				
<=0.25	<=4				
<=0.25	<=4				
<=0.25	<=4				
<=0.25	<=4	Pos	CTX-M	Neg	Pos/Neg
<=0.25	<=4				
<=0.25	<=4	Neg	Neg	Neg	Neg/Neg
<=0.25	<=4				
<=0.12	<=4	Pos	CTX-M	Neg	Pos/Neg
<=0.25	<=4				
<=0.12	<=4				
0.25	<=4				
<=0.12	<=4				
0.25	16	Pos	CTX-M	CMY	Pos/Pos
<=0.12	<=4				
<=0.25	<=4				
<=0.12	<=4	Neg	Neg	CMY	Neg/Pos
<=0.25	<=4				
<=0.12	<=4				
<=0.25	<=4				
<=0.25	<=4				
<=0.25	>64				
<=0.12	<=4				
<=0.12	<=4				
<=0.25	<=4				
<=0.25	<=4				
<=0.25	<=4				
<=0.25	<=4				
<=0.25	<=4	Pos	CTX-M	Neg	Pos/Neg
<=0.25	<=4	Pos	CTX-M	Neg	Pos/Neg
<=0.25	<=4				
<=0.12	<=4				
<=0.12	<=4				
<=0.12	<=4				
<=0.12	<=4				
<=0.12	<=4				
<=0.12	<=4				
<=0.12	<=4				
<=0.12	<=4				
<=0.12	<=4				
<=0.12	<=4				
<=0.25	<=4				
<=0.12	<=4				
<=0.12	<=4				
0.25	<=4	Pos	CTX-M	Neg	Pos/Neg
<=0.12	8	Neg	Neg	CMY	Neg/Pos
<=0.12	<=4				
<=0.12	<=4	Neg	Neg	CMY	Neg/Pos
<=0.12	<=4				
<=0.12	<=4				
<=0.25	<=4				
<=0.25	<=4				
<=0.25	<=4				
<=0.25	<=4				
<=0.25	<=4				

<=0.25	<=4				
<=0.25	<=4				
<=0.25	<=4				
<=0.25	<=4				
<=0.25	<=4				
<=0.25	<=4				
<=0.25	<=4				
<=0.25	<=4				
<=0.25	<=4				
<=0.25	<=4	Neg	Neg	CMY	Neg/Pos
<=0.25	<=4				
<=0.25	<=4				
<=0.12	<=4				
<=0.12	<=4				
<=0.12	8	Neg	Neg	CMY	Neg/Pos
<=0.12	<=4				
<=0.12	<=4				
0.25	8	Pos	CTX-M	CMY	Pos/Pos
<=0.12	<=4	Pos	CTX-M	Neg	Pos/Neg
<=0.25	<=4				
<=0.25	<=4				
<=0.25	<=4				
<=0.25	64	Neg	Neg	CMY, DHA	Neg/Pos
<=0.25	<=4				
<=0.25	<=4	Neg	Neg	CMY	Neg/Pos
<=0.25	<=4				
<=0.25	<=4				
<=0.25	<=4				
<=0.25	8	Pos	CTX-M	Neg	Pos/Neg
<=0.25	16	Pos	CTX-M	Neg	Pos/Neg
<=0.25	<=4				
<=0.25	<=4				
<=0.25	<=4				
<=0.25	<=4				
<=0.25	<=4				
<=0.25	<=4				
<=0.25	<=4				
<=0.25	<=4				
<=0.25	<=4				
<=0.12	<=4				
<=0.25	<=4				
<=0.25	<=4				
<=0.25	<=4				
<=0.25	<=4				
<=0.25	<=4				
<=0.25	<=4				
<=0.25	<=4				
<=0.25	<=4				
<=0.25	<=4				
<=0.25	<=4				
<=0.25	<=4				
<=0.25	<=4				
<=0.25	<=4				
<=0.25	<=4				
0.5	8				
<=0.25	<=4				
<=0.25	<=4				
<=0.25	<=4				
<=0.25	<=4				
<=0.25	<=4				
0.25	8	Pos	CTX-M	CMY	Pos/Pos

<=0.25	<=4				
<=0.25	<=4				
<=0.25	<=4				
<=0.25	<=4				
<=0.25	<=4				
<=0.25	<=4				
<=0.25	<=4				
<=0.12	<=4				
<=0.12	<=4				
0.25	<=4				
<=0.12	<=4				
<=0.12	<=4				
<=0.12	<=4	Neg	Neg	DHA	Neg/Pos
<=0.25	<=4				
<=0.25	<=4				
<=0.25	<=4				
<=0.25	<=4				
<=0.25	<=4				
<=0.25	<=4				
<=0.12	<=4				
<=0.25	<=4				
<=0.25	<=4	Neg	Neg	Neg	Neg/Neg
<=0.25	<=4				
<=0.25	<=4				
<=0.25	<=4				
0.25	<=4				
<=0.25	<=4				
<=0.25	<=4	Neg	Neg	CMY	Neg/Pos
<=0.25	<=4				
<=0.25	16	Pos	CTX-M	Neg	Pos/Neg
<=0.25	<=4				
<=0.25	<=4	Pos	CTX-M	Neg	Pos/Neg
<=0.12	<=4				
<=0.12	<=4	Neg	Neg	Neg	Neg/Neg
<=0.12	<=4				
<=0.12	<=4				
<=0.12	<=4				
<=0.12	<=4				
<=0.12	<=4				
<=0.12	<=4				
<=0.12	64	Neg	SHV	CMY, DHA	Pos/Pos
<=0.12	<=4				
0.25	<=4	Neg	Neg	CMY	Neg/Pos
<=0.12	<=4				
<=0.12	<=4				
<=0.12	<=4	Neg	Neg	CMY	Neg/Pos
<=0.12	<=4				
<=0.25	<=4	Neg	Neg	CMY	Neg/Pos
<=0.25	<=4				
<=0.25	<=4				
<=0.25	<=4				
<=0.12	<=4				
<=0.12	<=4				
<=0.12	<=4				
<=0.12	<=4				
<=0.12	<=4				
<=0.12	<=4				
0.25	<=4				
<=0.12	<=4				
<=0.12	<=4				
<=0.12	<=4				

<=0.25	<=4				
<=0.12	<=4				
<=0.12	<=4				
<=0.12	<=4				
<=0.12	<=4				
<=0.12	<=4				
<=0.12	<=4				
<=0.12	<=4				
<=0.12	<=4				
<=0.12	<=4				
<=0.12	<=4				
<=0.25	<=4				
<=0.25	<=4				
<=0.25	<=4				
<=0.25	<=4				
<=0.25	<=4				
<=0.25	<=4				
<=0.25	<=4				
<=0.12	<=4				
<=0.12	<=4				
<=0.12	<=4				
<=0.25	<=4				
<=0.25	<=4				
<=0.25	<=4				
<=0.25	>64	Neg	Neg	Neg	Neg/Neg
<=0.25	<=4				
<=0.25	>64	Neg	CTX-M	CMY	Pos/Pos
<=0.25	<=4				
<=0.25	<=4				
<=0.25	<=4				
<=0.12	<=4				
<=0.12	<=4				
<=0.12	<=4				
<=0.12	<=4				
<=0.12	32				
<=0.12	<=4				
<=0.25	<=4				
<=0.25	<=4				
<=0.25	<=4				
<=0.25	<=4				
<=0.12	<=4				
<=0.25	<=4	Pos	CTX-M	Neg	Pos/Neg
<=0.12	<=4				
<=0.25	16	Pos	CTX-M	CMY	Pos/Pos
<=0.12	<=4				
<=0.12	16	Neg	CTX-M	CMY	Pos/Pos
<=0.25	<=4				
<=0.25	<=4				
<=0.25	<=4				
0.5	>64	Pos	CTX-M, SHV	CMY, DHA	Pos/Pos
<=0.25	<=4				
<=0.25	<=4				
<=0.25	<=4				
<=0.25	<=4				
<=0.25	<=4				
<=0.25	<=4				
<=0.25	<=4				
<=0.25	<=4				
<=0.12	<=4				
0.25	16	Neg	Neg	CMY	Neg/Pos
<=0.12	<=4				

<=0.12	<=4				
<=0.12	<=4				
<=0.12	<=4	Pos	CTX-M	Neg	Pos/Neg
<=0.12	<=4				
<=0.12	<=4				
<=0.12	<=4				
0.25	16	Neg	Neg	CMY	Neg/Pos
<=0.12	<=4				
<=0.12	<=4				
<=0.12	<=4				
<=0.12	<=4				
<=0.12	<=4				
<=0.12	<=4				
<=0.12	<=4				
<=0.12	<=4				
<=0.12	<=4				
<=0.25	<=4				
<=0.12	<=4				
<=0.12	<=4				
<=0.12	<=4				
<=0.25	<=4	Pos	CTX-M	Neg	Pos/Neg
<=0.25	>64	Pos	CTX-M	Neg	Pos/Neg
<=0.25	<=4	Pos	CTX-M	Neg	Pos/Neg
0.5	<=4				
<=0.25	<=4				
<=0.25	<=4				
<=0.25	<=4				
<=0.25	<=4				
<=0.25	<=4				
<=0.25	<=4				
<=0.25	<=4				
<=0.25	<=4				
<=0.25	8	Neg	Neg	CMY	Neg/Pos
<=0.25	<=4				
<=0.25	<=4				
<=0.25	<=4				
<=0.25	<=4				
<=0.25	<=4				
<=0.25	<=4				
<=0.25	>64	Neg	Neg	CMY	Neg/Pos
<=0.25	<=4				
<=0.25	<=4	Pos	CTX-M	Neg	Pos/Neg
<=0.25	<=4				
<=0.25	<=4				
<=0.25	<=4				
<=0.25	<=4				
<=0.25	<=4				
<=0.25	<=4	Pos	CTX-M	Neg	Pos/Neg
<=0.25	<=4	Pos	CTX-M	Neg	Pos/Neg
<=0.25	<=4	Pos	CTX-M	Neg	Pos/Neg
<=0.25	<=4				
<=0.25	<=4				
<=0.25	<=4				
<=0.25	<=4				
<=0.25	<=4				
<=0.25	8	Neg	Neg	CMY	Neg/Pos
<=0.25	<=4	Neg	Neg	Neg	Neg/Neg
<=0.25	64	Neg	Neg	CMY	Neg/Pos
<=0.25	<=4	Pos	CTX-M	Neg	Pos/Neg
<=0.25	<=4	Pos	CTX-M	Neg	Pos/Neg
<=0.25	<=4	Pos	CTX-M	Neg	Pos/Neg

<=0.25	<=4	Pos	CTX-M	Neg	Pos/Neg
<=0.25	<=4	Pos	CTX-M	Neg	Pos/Neg
<=0.25	<=4	Pos	CTX-M	Neg	Pos/Neg
<=0.25	<=4	Pos	CTX-M	Neg	Pos/Neg
<=0.25	<=4	Pos	CTX-M	Neg	Pos/Neg
<=0.25	>64	Neg	Neg	CMY	Neg/Pos
<=0.25	>64	Pos	CTX-M	Neg	Pos/Neg
<=0.25	<=4	Pos	CTX-M	Neg	Pos/Neg
<=0.25	<=4	Pos	CTX-M	Neg	Pos/Neg
<=0.25	<=4	Pos	CTX-M	Neg	Pos/Neg
<=0.25	>64	Pos	CTX-M	CMY	Pos/Pos
<=0.25	<=4	Pos	CTX-M	Neg	Pos/Neg
<=0.25	<=4	Pos	CTX-M	Neg	Pos/Neg
<=0.25	<=4	Pos	CTX-M	Neg	Pos/Neg
<=0.25	<=4	Pos	CTX-M	Neg	Pos/Neg
<=0.25	<=4	Pos	CTX-M	Neg	Pos/Neg
<=0.25	64	Pos	CTX-M	Neg	Pos/Neg
<=0.25	<=4	Pos	CTX-M	Neg	Pos/Neg
<=0.25	>64	Pos	CTX-M	Neg	Pos/Neg
<=0.25	<=4	Pos	CTX-M	Neg	Pos/Neg
<=0.25	<=4	Pos	CTX-M	Neg	Pos/Neg
<=0.25	<=4				
<=0.25	<=4	Neg	Neg	CMY	Neg/Pos
<=0.25	<=4				
<=0.25	<=4				
<=0.25	32	Neg	Neg	CMY	Neg/Pos
<=0.25	<=4				
<=0.25	<=4				
<=0.25	<=4				
<=0.25	<=4				
<=0.25	>64				
<=0.25	<=4				
<=0.25	<=4				
<=0.25	<=4				
<=0.25	<=4				
<=0.25	<=4				
<=0.25	<=4	Pos	CTX-M	Neg	Pos/Neg
<=0.25	<=4	Pos	CTX-M	Neg	Pos/Neg
<=0.25	<=4				
<=0.25	<=4				
<=0.25	<=4				
<=0.25	<=4				
<=0.25	<=4				
<=0.25	<=4				
<=0.25	<=4				
<=0.25	<=4				
<=0.25	<=4				
<=0.25	>64	Neg	Neg	CMY	Neg/Pos
<=0.25	<=4				
<=0.25	<=4				
<=0.25	<=4				
<=0.25	<=4				
<=0.25	<=4				
<=0.25	<=4				
<=0.25	<=4				
<=0.25	<=4				
<=0.25	<=4				
0.5	16	Pos	CTX-M	CMY	Pos/Pos
<=0.25	<=4				
0.5	<=4				
<=0.25	<=4				

<=0.25	<=4				
<=0.25	<=4				
<=0.25	<=4				
<=0.25	<=4				
<=0.25	<=4				
<=0.25	>64	Neg	Neg	CMY	Neg/Pos
<=0.25	<=4				
<=0.25	<=4				
<=0.25	<=4				
<=0.25	<=4				
<=0.25	8				
<=0.25	8				
<=0.25	<=4				
<=0.25	8	Neg	Neg	CMY	Neg/Pos
<=0.25	>64	Neg	Neg	CMY	Neg/Pos
<=0.25	<=4				
<=0.25	<=4				
<=0.25	<=4				
<=0.25	8				
<=0.25	8	Pos	CTX-M	Neg	Pos/Neg
<=0.25	<=4				
<=0.25	<=4				
<=0.25	<=4	Neg	Neg	CMY	Neg/Pos
<=0.25	<=4				
<=0.25	<=4				
<=0.25	32	Neg	Neg	Neg	Neg/Neg
<=0.25	<=4				
<=0.25	<=4				
<=0.25	<=4	Neg	Neg	CMY	Neg/Pos
<=0.25	<=4				
<=0.25	<=4				
<=0.25	<=4				
<=0.25	<=4	Pos	CTX-M	Neg	Pos/Neg
<=0.25	<=4	Pos	CTX-M	Neg	Pos/Neg
<=0.25	<=4				
<=0.25	8	Pos	CTX-M	Neg	Pos/Neg
<=0.25	<=4				
<=0.25	<=4				
<=0.25	<=4				
<=0.25	16	Pos	CTX-M	Neg	Pos/Neg
<=0.25	<=4				
<=0.25	<=4				
<=0.25	<=4				
<=0.25	<=4	Neg	Neg	CMY	Neg/Pos
<=0.25	<=4				
<=0.25	<=4				
<=0.25	<=4				
<=0.25	<=4				
<=0.25	<=4	Pos	CTX-M	Neg	Pos/Neg
<=0.25	16	Pos	CTX-M	Neg	Pos/Neg
<=0.25	<=4				
<=0.25	8				
<=0.25	<=4				
<=0.25	32	Neg	Neg	CMY	Neg/Pos
<=0.25	<=4				
<=0.25	<=4				
<=0.25	<=4				
<=0.25	<=4				
<=0.25	<=4				
<=0.25	<=4				
<=0.25	<=4				

<=0.25	<=4				
<=0.25	<=4				
<=0.25	<=4				
<=0.25	<=4	Pos	CTX-M	Neg	Pos/Neg
<=0.25	<=4				
<=0.25	>64				
<=0.25	<=4				
<=0.25	<=4				
<=0.25	<=4	Pos	CTX-M	Neg	Pos/Neg
<=0.25	<=4				
<=0.25	<=4				
<=0.25	<=4				
<=0.25	<=4				
<=0.25	<=4				
<=0.25	<=4				
<=0.25	<=4				
<=0.25	<=4				
<=0.25	<=4				
<=0.25	<=4				
<=0.25	<=4	Neg	Neg	CMY	Neg/Pos
<=0.25	8	Neg	Neg	CMY	Neg/Pos
<=0.25	<=4				
<=0.25	<=4				
<=0.25	<=4				
<=0.25	<=4				
<=0.25	<=4				
<=0.25	<=4				
<=0.25	<=4				
<=0.25	<=4				
<=0.25	<=4				
<=0.25	<=4				
<=0.25	<=4				
<=0.25	<=4	Pos	CTX-M	Neg	Pos/Neg
<=0.25	<=4				
<=0.25	<=4				
<=0.25	<=4				
<=0.25	<=4				
<=0.25	<=4	Neg	Neg	CMY	Neg/Pos
<=0.25	<=4				
<=0.25	8				
<=0.25	<=4				
<=0.25	<=4				
<=0.25	<=4				
<=0.25	8				
<=0.25	<=4				
<=0.25	<=4				
<=0.25	<=4				
<=0.25	<=4				
<=0.25	<=4	Neg	Neg	Neg	Neg/Neg
<=0.25	<=4				
<=0.25	<=4				
<=0.25	<=4				
<=0.25	<=4				
<=0.25	<=4				
<=0.25	<=4				
<=0.25	<=4				
<=0.25	<=4				
<=0.25	<=4				
<=0.25	<=4				
<=0.25	<=4				
<=0.25	<=4				

<=0.25	<=4				
<=0.25	<=4				
<=0.25	<=4				
<=0.25	<=4				
<=0.25	<=4				
<=0.25	<=4				
<=0.25	<=4				
<=0.25	<=4				
<=0.25	<=4				
<=0.25	<=4				
<=0.25	<=4	Pos	CTX-M	Neg	Pos/Neg
<=0.25	<=4				
<=0.25	<=4				
<=0.25	<=4				
<=0.25	<=4				
<=0.25	<=4				
<=0.25	>64				
<=0.25	<=4				
<=0.25	<=4				
<=0.25	16	Pos	CTX-M	Neg	Pos/Neg
<=0.25	<=4				
<=0.25	<=4	Neg	Neg	CMY	Neg/Pos
<=0.25	<=4				
<=0.25	<=4				
<=0.25	<=4				
<=0.25	<=4				
<=0.25	<=4	Neg	Neg	DHA	Neg/Pos
<=0.25	<=4				
<=0.25	<=4				
<=0.25	<=4				
<=0.25	<=4				
<=0.25	<=4				
<=0.25	>64	Pos	CTX-M	Neg	Pos/Neg
<=0.25	<=4				
<=0.25	<=4				
<=0.25	32	Neg	Neg	CMY	Neg/Pos
<=0.25	<=4				
<=0.25	<=4				
<=0.25	<=4				
<=0.25	<=4				
<=0.25	<=4	Neg	Neg	Neg	Neg/Neg
<=0.25	<=4				
<=0.25	32	Pos	CTX-M	CMY	Pos/Pos
<=0.25	<=4				
<=0.25	<=4				
<=0.25	64	Pos	CTX-M	Neg	Pos/Neg
<=0.25	<=4				
<=0.25	<=4				
<=0.25	<=4				
<=0.25	<=4				
<=0.25	<=4				
<=0.25	<=4	Neg	Neg	Neg	Neg/Neg
<=0.25	<=4				
<=0.25	<=4				
<=0.25	<=4				
0.5	8	Neg	Neg	CMY	Neg/Pos
<=0.25	<=4				
<=0.25	<=4				
<=0.25	<=4				
<=0.25	<=4	Neg	Neg	CMY	Neg/Pos
<=0.25	<=4				

<=0.25	>64	Neg	Neg	CMY	Neg/Pos
<=0.25	<=4	Pos	CTX-M	Neg	Pos/Neg
<=0.25	<=4				
<=0.25	<=4	Neg	Neg	CMY	Neg/Pos
<=0.25	<=4				
<=0.25	<=4				
<=0.25	<=4				
<=0.25	<=4				
<=0.25	<=4				
<=0.25	<=4				
<=0.25	<=4				
<=0.25	<=4				
<=0.25	<=4				
<=0.25	<=4				
<=0.25	<=4				
<=0.25	<=4				
<=0.25	<=4				
<=0.25	<=4				
<=0.25	<=4				
<=0.25	16	Pos	CTX-M	CMY	Pos/Pos
<=0.25	8	Neg	SHV	CMY	Pos/Pos
<=0.25	<=4				
<=0.25	<=4				
<=0.25	<=4				
<=0.25	32	Pos	CTX-M	CMY	Pos/Pos
<=0.25	<=4				
<=0.25	<=4				
<=0.25	<=4				
<=0.25	<=4				
<=0.25	<=4				
<=0.25	<=4				
<=0.25	16	Neg	Neg	Neg	Neg/Neg
<=0.25	<=4				
<=0.25	<=4				
<=0.25	<=4				
<=0.25	<=4	Pos	CTX-M	Neg	Pos/Neg
<=0.25	<=4				
<=0.25	<=4				
<=0.25	<=4				
<=0.25	<=4				
<=0.25	<=4				
<=0.25	<=4				
<=0.25	<=4				
<=0.25	<=4				
<=0.25	>64	Neg	Neg	CMY	Neg/Pos
<=0.25	<=4				
<=0.25	<=4	Neg	Neg	CMY	Neg/Pos
<=0.25	<=4				
<=0.25	<=4				
<=0.25	<=4				
<=0.25	<=4				
<=0.25	<=4				
<=0.25	<=4	Pos	CTX-M	Neg	Pos/Neg
<=0.25	<=4				
<=0.25	<=4	Pos	CTX-M	Neg	Pos/Neg
<=0.25	<=4	Pos	CTX-M	Neg	Pos/Neg
<=0.25	<=4				
<=0.25	<=4				
<=0.25	<=4				
<=0.25	<=4				
<=0.25	8				

<=0.25	<=4				
<=0.25	8				
<=0.25	<=4				
<=0.25	<=4				
<=0.25	<=4	Pos	CTX-M	Neg	Pos/Neg
<=0.25	<=4				
<=0.25	<=4				
<=0.25	<=4				
<=0.25	<=4	Pos	CTX-M	Neg	Pos/Neg
<=0.25	<=4				
<=0.25	<=4				
<=0.25	<=4				
<=0.25	<=4				
<=0.25	<=4				
<=0.25	>64				
<=0.25	<=4				
<=0.25	<=4				
<=0.25	<=4				
<=0.25	<=4				
<=0.25	<=4				
<=0.25	<=4				
<=0.25	<=4				
<=0.25	<=4				
<=0.25	<=4				
<=0.25	<=4				
<=0.25	<=4				
<=0.25	<=4				
<=0.25	<=4				
<=0.25	<=4				
<=0.25	<=4	Neg	Neg	CMY	Neg/Pos
<=0.25	<=4				
<=0.25	<=4				
<=0.25	<=4				
<=0.25	<=4	Neg	Neg	CMY	Neg/Pos
<=0.25	8	Neg	Neg	CMY	Neg/Pos
<=0.25	<=4				
<=0.25	8	Neg	Neg	CMY	Neg/Pos
<=0.25	<=4	Neg	Neg	Neg	Neg/Neg
<=0.25	<=4				
<=0.25	32				
<=0.25	<=4				
<=0.25	<=4				
<=0.25	<=4				
<=0.25	<=4				
<=0.25	<=4				
<=0.25	<=4				
<=0.25	<=4				
<=0.25	<=4				
<=0.25	<=4	Pos	CTX-M	Neg	Pos/Neg
<=0.25	<=4				
<=0.25	8	Neg	Neg	CMY	Neg/Pos
<=0.25	<=4				
<=0.25	<=4				
<=0.25	<=4				
<=0.25	<=4				
<=0.25	<=4				
<=0.25	<=4				
<=0.25	<=4	Neg	Neg	Neg	Neg/Neg
<=0.25	<=4				
<=0.25	<=4				
<=0.25	<=4				

<=0.25	<=4				
<=0.25	<=4				
<=0.25	<=4				
<=0.25	<=4				
<=0.25	<=4				
<=0.25	<=4				
<=0.25	<=4				
<=0.25	<=4				
<=0.25	<=4				
<=0.25	32	Pos	CTX-M	CMY	Pos/Pos
<=0.25	<=4	Pos	CTX-M	Neg	Pos/Neg
<=0.25	<=4				
<=0.25	<=4				
<=0.25	<=4				
<=0.25	<=4				
<=0.25	<=4				
<=0.25	<=4				
<=0.25	<=4				
<=0.25	<=4	Pos	CTX-M	Neg	Pos/Neg
<=0.25	<=4				
<=0.25	<=4				
<=0.25	<=4	Pos	CTX-M	Neg	Pos/Neg
<=0.25	<=4				
<=0.25	<=4				
<=0.25	<=4				
<=0.25	16	Pos	CTX-M	Neg	Pos/Neg
<=0.25	<=4				
<=0.25	<=4				
<=0.25	<=4				
<=0.25	<=4				
<=0.25	<=4				
<=0.25	<=4				
<=0.25	<=4				
<=0.25	<=4				
<=0.25	<=4				
<=0.25	<=4	Neg	Neg	CMY	Neg/Pos
<=0.25	<=4				
<=0.25	<=4				
<=0.25	32				
<=0.25	<=4				
<=0.25	<=4				
<=0.25	16	Neg	Neg	CMY	Neg/Pos
<=0.25	<=4				
<=0.25	<=4				
<=0.25	<=4				
<=0.25	<=4	Neg	Neg	Neg	Neg/Neg
<=0.25	<=4	Neg	Neg	CMY	Neg/Pos
<=0.25	<=4				
<=0.25	<=4				
<=0.25	<=4				
<=0.25	<=4				
<=0.25	8	Neg	Neg	CMY	Neg/Pos
<=0.25	<=4				
<=0.25	<=4	Neg	Neg	DHA	Neg/Pos
<=0.25	8				
<=0.25	<=4				
<=0.25	8				
0.5	<=4	Pos	CTX-M	Neg	Pos/Neg
<=0.25	<=4				
<=0.25	<=4	Pos	CTX-M	Neg	Pos/Neg
<=0.25	<=4				

<=0.25	16	Neg	Neg	CMY	Neg/Pos
<=0.25	<=4				
<=0.25	<=4				
<=0.25	<=4				
<=0.25	<=4				
<=0.25	<=4				
<=0.25	>64				
<=0.25	<=4				
<=0.25	<=4				
<=0.25	<=4	Pos	CTX-M	Neg	Pos/Neg
<=0.25	<=4	Pos	CTX-M	Neg	Pos/Neg
<=0.25	8	Neg	Neg	CMY	Neg/Pos
<=0.25	<=4				
<=0.25	<=4	Neg	Neg	Neg	Neg/Neg
<=0.25	<=4	Pos	CTX-M	Neg	Pos/Neg
<=0.25	<=4	Pos	CTX-M	Neg	Pos/Neg
<=0.25	<=4				
<=0.25	<=4				
<=0.25	<=4				
<=0.25	>64				
<=0.25	<=4	Pos	CTX-M	Neg	Pos/Neg
<=0.25	<=4	Pos	CTX-M	Neg	Pos/Neg
<=0.25	<=4	Pos	CTX-M	Neg	Pos/Neg
<=0.25	<=4				
<=0.25	<=4				
<=0.25	<=4				
<=0.25	<=4				
<=0.25	<=4				
<=0.25	8	Neg	Neg	CMY	Neg/Pos
<=0.25	<=4				
<=0.25	<=4				
<=0.25	<=4				
<=0.25	<=4	Pos	CTX-M	Neg	Pos/Neg
<=0.25	<=4	Pos	CTX-M	Neg	Pos/Neg
<=0.25	<=4				
<=0.25	<=4	Pos	CTX-M	Neg	Pos/Neg
<=0.25	<=4				
<=0.25	<=4				
<=0.25	<=4				
<=0.25	<=4				
<=0.25	<=4				
<=0.25	<=4				
<=0.25	<=4	Neg	Neg	Neg	Neg/Neg
<=0.25	<=4				
<=0.25	<=4	Pos	CTX-M	Neg	Pos/Neg
<=0.25	<=4				
<=0.25	<=4				
<=0.25	<=4				
<=0.25	32	Neg	Neg	CMY	Neg/Pos
<=0.25	<=4				
<=0.25	<=4				
<=0.25	<=4				
<=0.25	<=4				
<=0.25	<=4				
<=0.25	<=4				
<=0.25	<=4				
<=0.25	<=4				
<=0.25	16	Pos	CTX-M	Neg	Pos/Neg
<=0.25	<=4				
<=0.25	<=4				
<=0.25	<=4				

<=0.25	<=4				
<=0.25	<=4				
<=0.25	<=4				
<=0.25	<=4				
<=0.25	<=4				
<=0.25	<=4				
<=0.25	<=4				
<=0.25	<=4				
<=0.25	<=4				
<=0.25	<=4				
<=0.25	<=4				
<=0.25	<=4				
<=0.25	<=4				
<=0.25	<=4	Pos	CTX-M	Neg	Pos/Neg
<=0.25	<=4				
<=0.25	<=4				
<=0.25	<=4				
<=0.25	<=4	Pos	CTX-M	Neg	Pos/Neg
<=0.25	<=4				
<=0.25	<=4				
<=0.25	<=4				
<=0.25	<=4				
<=0.25	<=4				
<=0.25	<=4				
<=0.25	<=4				
<=0.25	<=4				
<=0.25	<=4				
<=0.25	<=4				
<=0.25	<=4	Pos	CTX-M	Neg	Pos/Neg
<=0.25	<=4	Pos	CTX-M	Neg	Pos/Neg
<=0.25	<=4				
<=0.25	<=4				
<=0.25	<=4				
<=0.25	8				
<=0.25	<=4				
<=0.25	<=4				
<=0.25	<=4				
<=0.25	<=4				
<=0.25	<=4				
<=0.25	<=4				
<=0.25	<=4				
<=0.25	<=4				
<=0.25	<=4				
<=0.25	<=4				
<=0.25	<=4	Neg	Neg	CMY	Neg/Pos
<=0.25	<=4				
<=0.25	32	Neg	Neg	CMY	Neg/Pos
0.5	<=4	Pos	CTX-M	Neg	Pos/Neg
<=0.25	<=4				
<=0.25	<=4	Pos	CTX-M	Neg	Pos/Neg
<=0.25	<=4				
<=0.25	<=4				
<=0.25	<=4				
<=0.25	<=4				
<=0.25	<=4				
<=0.25	<=4				
<=0.25	<=4				
<=0.25	<=4				
<=0.25	<=4	Pos	CTX-M	Neg	Pos/Neg
<=0.25	<=4				
<=0.25	<=4				

<=0.25	<=4				
<=0.25	<=4				
<=0.25	<=4				
<=0.25	<=4				
<=0.25	<=4	Neg	Neg	CMY	Neg/Pos
<=0.25	<=4	Neg	Neg	CMY	Neg/Pos
<=0.25	<=4				
<=0.25	<=4				
<=0.25	<=4	Pos	CTX-M	Neg	Pos/Neg
<=0.25	<=4				
<=0.25	<=4				
<=0.25	<=4				
<=0.25	<=4				
<=0.25	<=4				
<=0.25	<=4				
<=0.25	<=4				
<=0.25	<=4				
<=0.25	<=4	Neg	Neg	CMY	Neg/Pos
<=0.25	<=4				
<=0.25	<=4	Pos	CTX-M	Neg	Pos/Neg
<=0.25	<=4				
<=0.25	<=4				
<=0.25	<=4				
<=0.25	<=4				
<=0.25	<=4				
<=0.25	<=4	Pos	CTX-M	Neg	Pos/Neg
<=0.25	<=4				
<=0.25	<=4				
<=0.25	<=4				
0.5	<=4				
<=0.25	<=4				
<=0.25	<=4				
<=0.25	<=4				
<=0.25	<=4				
<=0.25	<=4				
<=0.25	<=4				
<=0.25	<=4				
<=0.25	<=4				
<=0.25	<=4				
<=0.25	<=4				
<=0.25	<=4				
<=0.25	<=4				
<=0.25	<=4				
<=0.25	<=4				
<=0.25	<=4				
<=0.25	<=4				
<=0.25	<=4				
<=0.25	<=4				
<=0.25	<=4				
<=0.25	<=4	Neg	Neg	CMY	Neg/Pos
<=0.25	<=4				
<=0.25	8				
<=0.25	<=4				
<=0.25	<=4				
<=0.25	<=4				
<=0.25	<=4				
<=0.25	<=4				
<=0.25	<=4				
<=0.25	<=4				
<=0.25	<=4				
<=0.25	64	Neg	CTX-M	CMY	Pos/Pos

<=0.25	<=4	Pos	CTX-M	Neg	Pos/Neg
0.5	8	Pos	CTX-M	CMY	Pos/Pos
<=0.25	<=4				
<=0.25	<=4				
<=0.25	<=4				
<=0.25	<=4				
<=0.25	<=4				
<=0.25	<=4				
<=0.25	<=4				
<=0.25	<=4				
<=0.25	<=4				
<=0.25	<=4				
<=0.25	<=4				
<=0.25	>64	Neg	Neg	CMY	Neg/Pos
<=0.25	<=4				
<=0.25	<=4	Pos	CTX-M	Neg	Pos/Neg
<=0.25	8	Neg	Neg	CMY	Neg/Pos
<=0.25	<=4				
<=0.25	8	Pos	CTX-M	Neg	Pos/Neg
<=0.25	<=4				
<=0.25	<=4				
<=0.25	<=4				
<=0.25	<=4				
<=0.25	8				
<=0.25	<=4				
<=0.25	<=4				
<=0.25	<=4				
<=0.25	<=4	Neg	Neg	CMY	Neg/Pos
<=0.25	<=4				
<=0.25	<=4				
<=0.25	<=4				
<=0.25	<=4				
<=0.25	8	Neg	Neg	CMY	Neg/Pos
<=0.25	<=4				
<=0.25	<=4				
<=0.25	<=4				
<=0.25	16	Neg	Neg	CMY	Neg/Pos
<=0.25	<=4				
<=0.25	<=4				
<=0.25	<=4				
<=0.25	<=4				
<=0.25	<=4				
<=0.25	16	Pos	CTX-M	CMY	Pos/Pos
<=0.25	<=4				
<=0.25	<=4				
<=0.25	<=4				
<=0.25	<=4				
<=0.25	<=4				
<=0.25	<=4				
<=0.25	<=4	Pos	CTX-M	Neg	Pos/Neg
<=0.25	<=4	Neg	Neg	Neg	Neg/Neg
<=0.25	<=4				
<=0.25	<=4				
<=0.25	<=4				
<=0.25	<=4				
<=0.25	<=4				
<=0.25	<=4				
<=0.25	<=4				
<=0.25	<=4	Pos	CTX-M	Neg	Pos/Neg
<=0.25	<=4				
<=0.25	<=4				

<=0.25	<=4				
<=0.25	<=4				
<=0.25	<=4				
<=0.25	<=4				
<=0.25	<=4				
<=0.25	<=4				
<=0.25	<=4				
<=0.25	64	Neg	Neg	CMY	Neg/Pos
<=0.25	<=4				
<=0.25	<=4				
<=0.25	<=4				
<=0.25	<=4	Neg	Neg	CMY	Neg/Pos
<=0.25	<=4				
<=0.25	<=4				
<=0.25	<=4				
<=0.25	<=4				
<=0.25	<=4				
<=0.25	8	Neg	Neg	CMY	Neg/Pos
<=0.25	<=4				
<=0.25	<=4				
<=0.25	<=4				
<=0.25	<=4				
<=0.25	<=4				
<=0.25	<=4				
<=0.25	16	Pos	CTX-M	Neg	Pos/Neg
<=0.25	<=4				
<=0.25	8				
<=0.25	<=4				
<=0.25	<=4				
<=0.25	<=4				
<=0.25	<=4				
<=0.25	<=4				
<=0.25	<=4				
<=0.25	<=4				
<=0.25	<=4				
<=0.25	<=4				
<=0.25	<=4				
<=0.25	<=4				
<=0.25	<=4	Neg	Neg	CMY	Neg/Pos
<=0.25	<=4				
<=0.25	<=4				
<=0.25	<=4				
<=0.25	<=4				
<=0.25	<=4				
<=0.25	<=4				
<=0.25	<=4				
<=0.25	<=4	Pos	CTX-M	Neg	Pos/Neg
<=0.25	16	Neg	Neg	CMY	Neg/Pos
<=0.25	<=4				
<=0.25	<=4				
<=0.25	<=4				
<=0.25	<=4				
<=0.25	<=4	Neg	Neg	CMY	Neg/Pos
<=0.25	<=4				
<=0.25	<=4				
<=0.25	<=4				
<=0.25	<=4				
<=0.25	<=4				
<=0.25	<=4	Pos	CTX-M	Neg	Pos/Neg
<=0.25	<=4				
<=0.25	<=4				

<=0.25	8	Neg	Neg	CMY	Neg/Pos
<=0.25	<=4				
<=0.25	<=4				
<=0.25	<=4				
0.5	<=4				
<=0.25	<=4	Neg	Neg	CMY	Neg/Pos
<=0.25	<=4	Pos	CTX-M	Neg	Pos/Neg
<=0.25	<=4				
<=0.25	<=4				
<=0.25	<=4				
